# Divergent Synthesis of Functionalized Indenopyridin-2-ones and 2-Pyridones via Benzyl Group Transfer: Two Cases of Aza-semipinacol-Type Rearrangement

**DOI:** 10.1021/acs.orglett.2c03361

**Published:** 2022-11-11

**Authors:** Jacek G. Sośnicki, Aleksandra Borzyszkowska-Ledwig, Tomasz J. Idzik, Magdalena M. Lubowicz, Gabriela Maciejewska, Łukasz Struk

**Affiliations:** † West Pomeranian University of Technology, Szczecin, Faculty of Chemical Technology and Engineering, Department of Organic and Physical Chemistry, Al. Piastów 42, Szczecin 71-065, Poland; ‡ Wrocław University of Science and Technology, Faculty of Chemistry, Wybrzeże Wyspiańskiego 27, 50-370 Wrocław, Poland

## Abstract

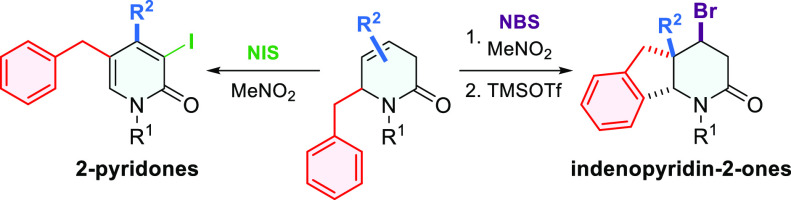

The synthesis of bromo-substituted indeno[1,2-*b*]pyridin-2-ones and 3-iodo-5-benzyl-substituted 2-pyridones, starting from easily available 6-benzyl-3,6-dihydropyridin-2(1*H*)-ones, triggered by NBS and NIS, respectively, is described. In both syntheses, a transfer of a benzyl group from the C6 to C5 lactam position occurred, indicating a novel aza-semipinacol-type rearrangement. Identification of intermediate compounds in both transformations supported the proposed reaction mechanisms. In the process of checking the scope of the method’s application, functionalized indeno[1,2-*b*]pyridin-2-ones and 5-benzyl-2-pyridones were obtained.

Functionalized 2-pyridones [pyridin-2(1*H*)-ones] have attracted considerable attention since they were recognized as key structural units present in a broad spectrum of naturally occurring compounds^1^ and in several active pharmaceuticals.^2^ They also have been applied as valuable precursors of a variety of naturally occurring and naturally inspired bioactive polycyclic piperidines.^3^

Driven by the need to synthesize novel bioactive piperidine-containing polycycles, we explored 2-pyridones as a platform for obtaining benzoquinolizidine^4^ and quinolizidine^5^ derivatives, aryl-substituted indeno[2,1-*b*]pyridones (resembling the core of haouamine^6^), indeno[2,1-*c*]piperidine,^7^ and benzomorphanones.^8^ The latter have been achieved by treatment of easily accessible 6-benzyl-3,6-dihydropyridin-2(1*H*)-one^9^ with *N*-bromosuccinimide (NBS) in wet CH_3_NO_2_ as a solvent and with (PhO)_3_P as a catalyst (Scheme 1). The results revealed that the presence of a substituent at C4 (capable of stabilizing the carbocation) provided a lower amount of benzomorphanone in favor of α,β-unsaturated δ-lactam (Scheme 1).^8^

**Scheme 1:**
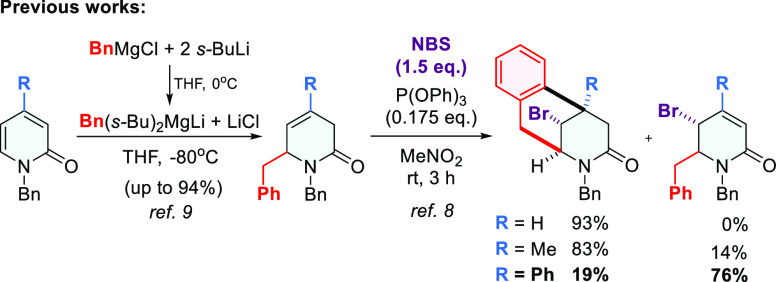
Reaction Product Distribution Found by Treatment of 3,6-Dihydropyridin-2-ones with NBS according to the Type of Substituent R Present at C4, as Described in Previous Work^8^

Preliminary tests carried out under the same reaction conditions for C5-methyl-substituted derivative **2a** showed surprisingly the formation of a novel product **5a** apart from the expected benzomorphanone **4a** (Scheme 2, part I). On the basis of the structural analysis by ^1^H and ^13^C NMR spectroscopy, including the investigation of nuclear Overhauser effects (^1^H,^1^H NOESY) and dihedral angle analysis,^10^ it was found that compound **5a** was a rearranged product, in which the benzyl group was transferred from the C6 to C5 position, forming an all-carbon quaternary center. Both above-mentioned facts are of great importance. First, no such rearrangement has been hitherto observed for lactams, whereas, when it has been observed for other non-lactam systems, no benzyl group transfer has been noted to take place. Literature survey indicated that transformations of this type occurred in the synthesis of cyclobutylimines,^11^ in asymmetric hydrogenation of cyclic N-sulfonyl amino alcohols,^12^ and in the synthesis of indole^13^ and piperidine^14^ ring-containing molecules. A similar 1,2-shift of substituents, promoted by SmI_2_, was also observed for uracils.^15^ Second, although the compounds with all-carbon quaternary stereogenic centers are of unquestionable importance as they occur in naturally and biologically active compounds,^16^ the synthesis of indeno[1,2-*b*]piperidin(on)es^17^ containing this structural motif is still challenging.

**Scheme 2:**
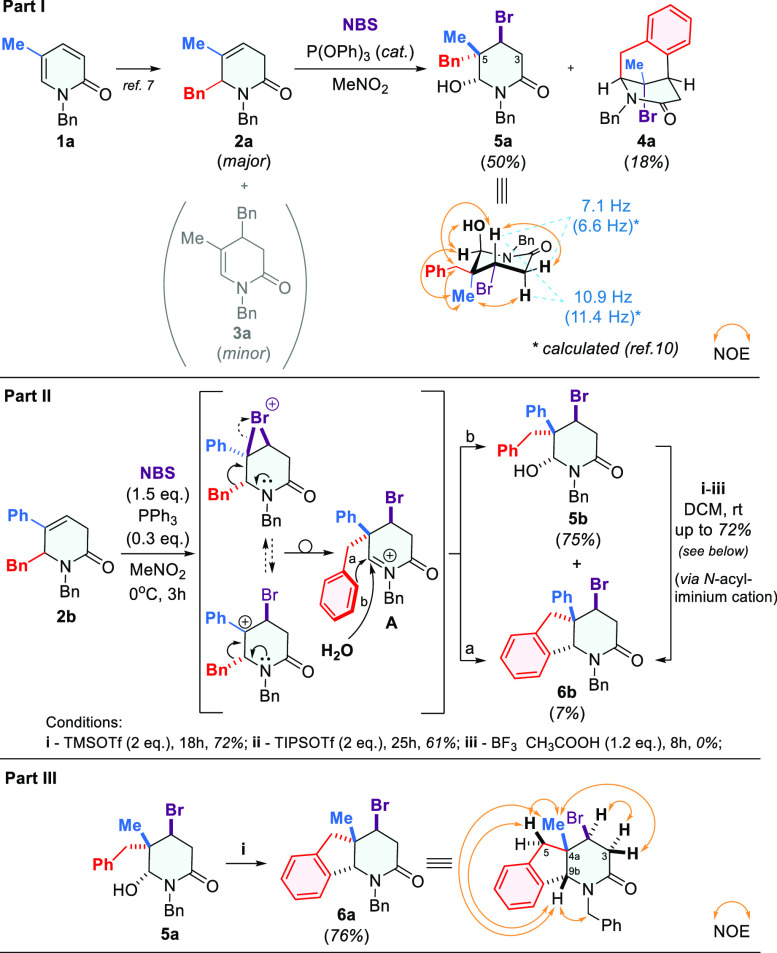
Preliminary Results of the Study

Extension of the initial studies, focused on the novel type of rearrangement, revealed that C5-phenyl-substituted 3,6-dihydro-2-pyridone **2b**, under the same reaction conditions, gave a greater amount of the rearranged product **5b,** together with a small amount of indeno[1,2-*b*]pyridin-2-one **6b**, both possessing a novel quaternary chiral center (Scheme 2, part II). The interesting biological activity of the species built on an indeno[1,2-*b*]pyridine skeleton^18^ (the same as in **6b**) and a new way of its formation (through the aza-semipinacol rearrangement followed by an electrophilic substitution) prompted us to work out the best possible method for their synthesis. We also wanted to check some possibilities of further functionalization, in particular the applicability of *N*-iodosuccinimide (NIS) in these transformations. Herein, we report the results of our effort toward the synthesis of indeno[1,2-*b*]pyridin-2-ones from 6-benzyl-3,6-dihydropyridin-2(1*H*)-ones with the use of NBS and NIS.

Our first tests aimed at optimization of transformations of **2b** toward indeno[1,2-*b*]pyridin-2-ones concentrated on finding a way to avoid **5b** formation. We assumed that the formation of **5b** and **6b** must proceed via *N*-acyliminium ion **A** (Scheme 2, part II) and the presence of a water molecule as a nucleophile could compete with a phenyl ring in the reaction with **A**, resulting in the formation of **6b** in lower yield. Therefore, dry instead of wet CH_3_NO_2_, a temperature of 60 °C, and a reaction time of 3.5 h at the unchanged ratio of NBS (1.5 equiv) and (PhO)_3_P (0.3 equiv) were applied. Unfortunately, changes in these parameters permitted obtaining the expected product **6b** only in a moderate yield of ∼50%, as was quantified by ^1^H NMR using an internal reference standard. Further attempts to change the parameters did not improve the yield of **6b**.

On the other hand, it is known that an *N*-acyliminium ion can be generated from 6-hydroxy lactams using Lewis acid such as BF_3_·OEt_2_ or TMSOTf.^19^ Testing BF_3_·CH_3_COOH, TMSOTf, and additionally TIPSOTf,^4^ we found that TMSOTf was the most effective, as it enabled the conversion of compound **5b** into **6b** in 72% yield as well as **5a** into **6a** in 76% yield (Scheme 2, parts II and III). Finally, due to the observed instability of some 6-hydroxylactams **5** and their efficient formation in wet nitromethane, we decided to target indenopyridines **6** in two reaction steps without isolation of the intermediate hydroxylactams, applying slightly adjusted conditions in both steps. The proposed method allowed us to obtain the desired products **6** in good and moderate yields (Scheme 3). However, some setbacks were also encountered. The substrate with a 2-Cl-benzyl group (**2i**) gave a low yield of the rearranged product **6i** (Scheme 3), while the attempts to transfer bigger groups, such as biphenyl (**2k**) or naphthyl (**2l**), ended in complete failure because these substrates were decomposed, yielding many unidentified products (Scheme 3). Furthermore, experiments performed with C6-substituted lactam **2j** showed that steric effects play a critical and negative role in the rearrangement of this type, since the reactions with this substrate led to an unstable and unidentified product (Scheme 3).

**Scheme 3:**
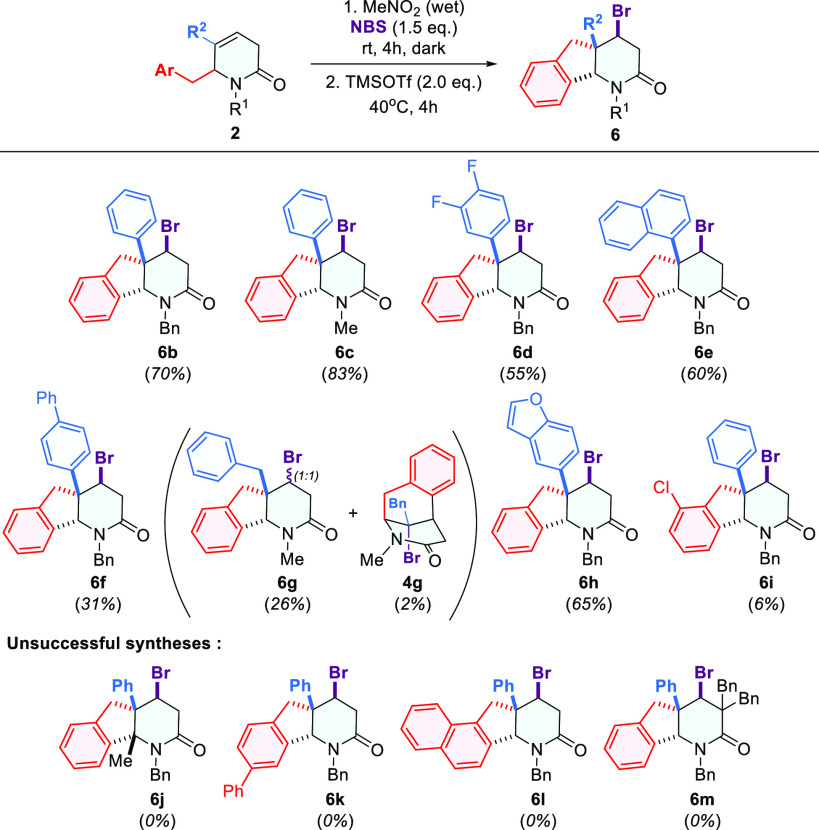
Two-Step Synthesis of 4-Bromo-1,3,4,4a,5,9b-hexahydro-2*H*-indeno[1,2-*b*]pyridin-2-one **6**

As far as the synthesis of functionalized indeno[1,2-*b*]pyridin-2-ones is concerned, we successfully transformed the obtained bromo-derivatives **6** into unsaturated indenopyridin-2-ones **7** in moderate to good yields using *t*-BuOK in THF, albeit the reaction conditions were not fully optimized (Scheme 4). The obtained indenopyridin-2-ones **7** seemed to be valuable compounds, capable of further derivatization as the conjugated double bond to carbonyl group in lactams has long been recognized as a reactive functional group.^20^ Furthermore, since we previously investigated the Michael addition reactions to unsaturated δ-thiolactams,^21^ we ran one successful lactam **7b** to thiolactam **8b** transformation test, in order to check the prospect of the availability of α,β-unsaturated indenopyridine-2-thione derivatives, which could be further explored in addition reactions as part of other synthetic projects (Scheme 4).

**Scheme 4:**
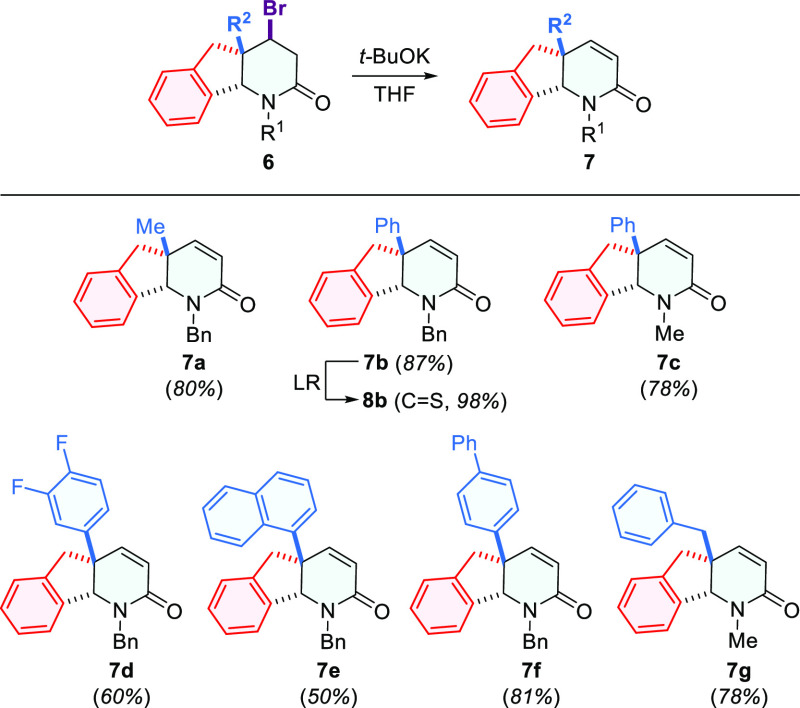
Synthesis of 1,4a,5,9b-Tetrahydro-2*H*-indeno[1,2-*b*]pyridine-2-ones **7** and Thione Analogue **8b**

As many reactions with the use of NBS and NIS leading to bromo- and iodo-substituted analogues, respectively, have been reported in the literature,^22^ we decided to expand the range of accessible indenopyridin-2-one products to obtain iodo-derivatives of **6**. Unfortunately, the treatment of 5-phenyl-substituted 3,6-dihydropyridone **2b** with NIS did not lead to any product, which was also the fact when 4,5-diphenyl-substituted substrate **2s** was used (Scheme 5, lower part). Only 4-substituted 6-benzyl 3,6-dihydropyridones turned out to be reactive upon treatment with NIS (Scheme 5, upper part). However, product analysis showed that, instead of iodo-substituted indenopyridin-2-ones, surprisingly, 5-benzyl-3-iodosubstituted 2-pyridones **9** were formed. To be more exact, in the case of 4-Ph substrates **2n**–**p**, 2-pyridones **9n**–**p** were the only products, while, for 4-Me substrates, apart from 2-pyridones **9q** and **9r**, benzomorphan **4q** and **4r** were also formed. It is important to emphasize that the structure of compound **9** proves that again, in the process of its formation, the benzyl group was transferred from the C6 to the C5 position.

**Scheme 5:**
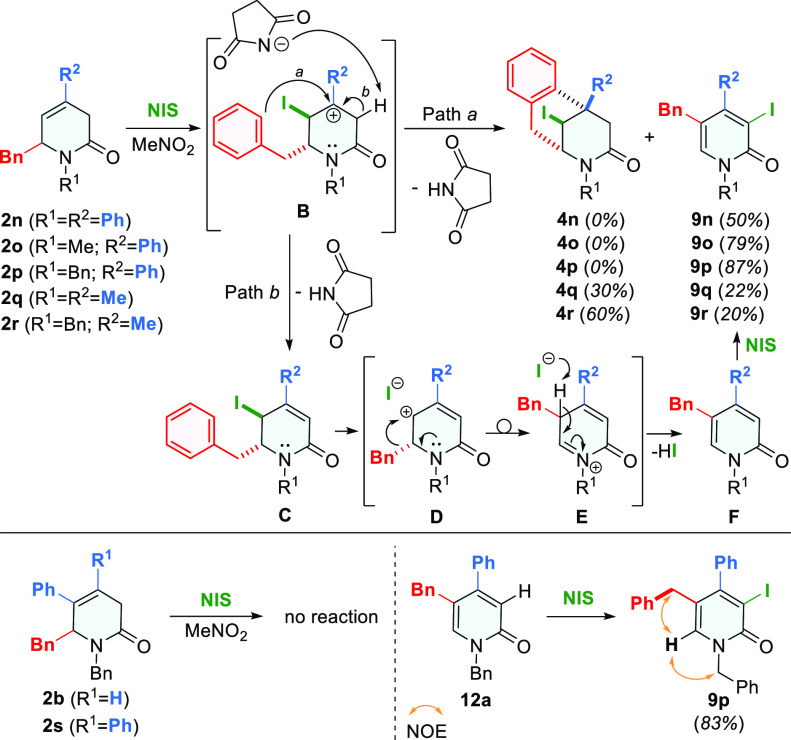
Reactions of **2** and **12a** with NIS

A reasonable mechanism for the formation of both iodo-products **4** and **9** is depicted in Scheme 5 (upper part). Since the presence of Me and Ph substituents at C4 is essential for obtaining **9** and these groups are capable of stabilizing carbenium ion (more efficient for Ph), it is reasonable to assume that at the first reaction stage carbocation **B** is formed. Its subsequent transformation can occur in two ways. Path **a** runs through intramolecular electrophilic substitution involving the benzyl ring, leading to iodobenzomorphanone **4**. Path **b** covers the intermediate product **C** formation via E1 elimination, followed by benzyl group transfer from C6 to C5 in carbocation **D**, which is created by dissociation of the iodide anion, which subsequently eliminates a proton from *N*-acyliminium cation **E**, yielding 2-pyridone **F**. Intermediate 2-pyridone **F** is immediately iodinated by NIS at the unoccupied C3 position. The following premises have contributed to the formulation of the above-proposed mechanism. The first is that the stable Br-analogue products, comparable to iodo-intermediate **C**, were previously isolated for C4-substituted substrates in the reaction with NBS (Scheme 1).^8^

The second premise is the easiness of iodination of 2-pyridone in the reaction with NIS, occurring at the last mechanism step, which was supported by a successful reaction test performed for compound **12a** upon treatment with NIS (Scheme 5, lower part). Finally, the formation of both postulated intermediate products **C** and **F** was observed during the reaction, which was followed by recording ^1^H NMR spectra in properly selected time intervals, in an NMR tube, in CD_3_NO_2_ solution by mixing of **2p** with NIS (see the Supporting Information, Scheme S17).

At the last stage of the study, the functionalization potential of iodo-pyridone **9** was successfully verified by a few cross-coupling reactions performed under standard conditions as well as by I–Mg exchange reaction followed by hydrolysis and deuterolysis (Scheme 6).

**Scheme 6:**
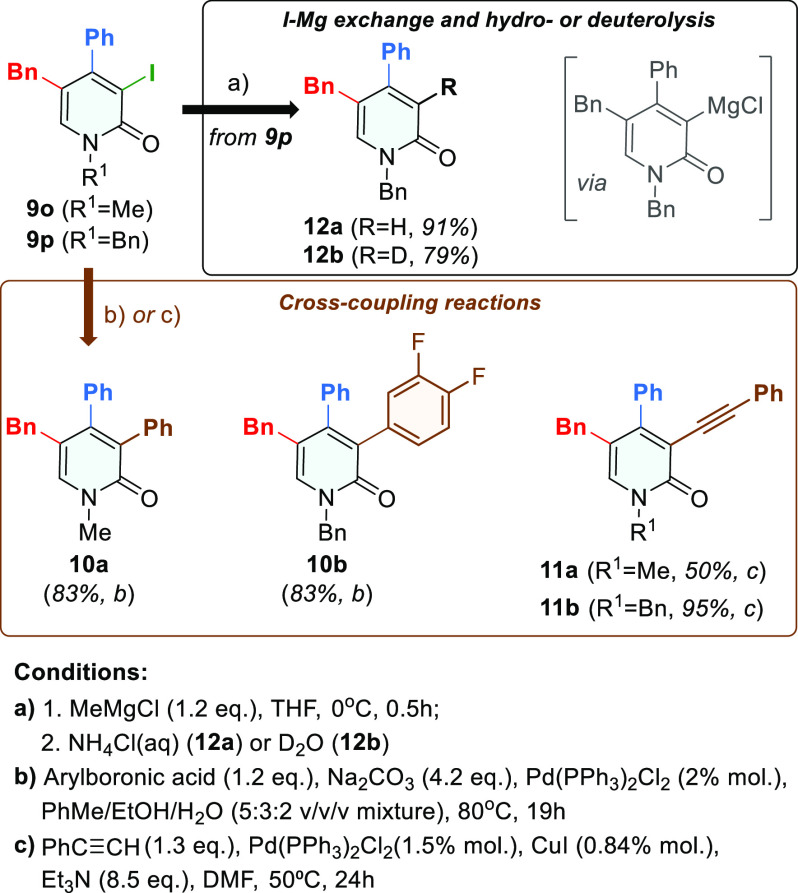
Synthesis of Functionalized 5-Bn-Substituted 2-Pyridones

In conclusion, we have demonstrated that the initially observed aza-semipinacol rearrangement relying on the transfer of a benzyl group permits obtaining functionalized indeno[1,2-*b*]pyridin-2-ones containing all-carbon quaternary stereogenic centers and 3-iodo-5-benzyl-substituted 2-pyridones. These two types of compounds, hardly accessible by other synthetic routes and capable of further functionalization, may gain interest in the drug development area due to the fact that compounds of this class show noteworthy pharmacological activity. It should be emphasized that, while the direction of the reactions via aza-pinacol rearrangements is determined by the presence of substituents at C5 or C4 in 6-benzyl-3,6-dihydropyridones and the application of NBS or NIS as a halogenating reagent, respectively, used under the same conditions, the success of these reactions depends on the possibility of creating a stable *N*-acyliminium cation, the formation of which is the driving force behind both rearrangements. Further studies focused on a novel aza-semipinacol-type rearrangement in δ-lactams that includes checking a more comprehensive range of substituents, and establishing the details of the mechanism are ongoing.

## Notes

The data underlying this study are available in the published article and its Supporting Information.

## Supporting Information Available

Experimental procedures, spectroscopic data, and ^1^H and ^13^C NMR spectra for all new compounds (PDF)
