# Supplementary material for: Divergent Synthesis of Functionalized Indenopyridin-2-ones and 2-Pyridones via Benzyl Group Transfer: Two Cases of Aza-semipinacol-Type Rearrangement
Source: Org Lett. 2022 Nov 11;24(46):8498–502. doi: 10.1021/acs.orglett.2c03361 (PMC9706813; doi:10.1021/acs.orglett.2c03361)
Supplement: Supplementary file 1 — ol2c03361_si_001.pdf [file ol2c03361_si_001.pdf]

Supporting Information

Divergent synthesis of functionalized indenopyridin-2-ones and 2-pyridones *via* benzyl group transfer: Two cases of aza-semipinacol-type rearrangement

Jacek G. Sośnicki,†* Aleksandra Borzyszkowska-Ledwig,† Tomasz J. Idzik,† Magdalena M. Lubowicz,† Gabriela Maciejewska,‡ Łukasz Struk†

†West Pomeranian University of Technology, Szczecin, Faculty of Chemical Technology and Engineering, Department of Organic and Physical Chemistry, Al. Piastów 42, Szczecin, 71-065, Poland.
‡Wrocław University of Technology, Faculty of Chemistry, Wybrzeże Wyspiańskiego 27, 50-370 Wrocław, Poland
sosnicki@zut.edu.pl

Table of Contents

1. General information…………………………………………...…………………… S2
2. Preparation of substrates …………………………………….….…….……..……S3
2a. Preparation of 2-pyridones **1d-1f** and **1j** ……………………………………………..S3
2b. Preparation of compound **1g**.. S6
2c. Preparation of compound **1h**.. S7
2d. Preparation of compound **1s**...S9
2e. Preparation of 6-benzyl-3,6-dihydropyridin-2-ones **2**..S10
2f. Synthesis of compounds **2i**, **2k**/**3k** and **2l**………………………………………………... S15
2g. Synthesis of 1,3,3,6-tetrabenzyl-5-phenyl-3,6-dihydropyridin-2(1*H*)-one (**2m**)………… S18
3. Procedure for the synthesis of **4a**, **5a**, **5b**, **6b** as a part of the preliminary studies............ S19
4. Procedure for the synthesis of **6a** and **6b** from 6-hydroksy lactams **5a** and **5b** as a part of the preliminary studies...S21
5. Synthesis of bromoindenopyridin-2-ones **6** from lactams **2**... S22
6. Synthesis of 1,4a,5,9b-tetrahydro-2*H*-indeno[1,2-*b*]pyridine-2-ones **7**………………… S28
7. Synthesis of indenopyridine-2-thione **8b**……………………………………………… S31
8. General procedure for the synthesis of 3-iodopyridin-2-ones **9** ……………………….. S32
9. Procedure for the synthesis of 2-pyridones **10a**, **10b** ………………………………….. S35
10. Preparation of 2-pyridones **11a**, **11b** ……………………………………….…... S36
11. Procedure for the synthesis of 2-pyridones **12a** and **12b** ……………………………… S38
12 Study of **9p** formation by following of the progress of the reaction between **2p** and NIS with the aid of ^{1}H NMR spectrocsopy ……………………………………….. S39
13. ^{1}H and ^{13}C NMR spectra ………………………………………………….……… S42
14. 14. Selected 2D NMR spectra of **5a**, **5b**, **6a**, **6c**, **6d**, **9o**, **9p**, **9q**, **9r** and **11b**…………… S102
15. References …………………………………………………………………............ S112

1. General information

Melting points were determined on a Boetius hot stage apparatus. ^{1}H, ^{13}C and ^{19}F NMR spectroscopic measurements were performed on a Bruker DPX 400 Avance III HD spectrometer, operating at 400.2, 100.6 and 376.6 MHz, respectively. TMS (internal standard, $\delta_{H,C}$ = 0 ppm) and $PhCF_3$ (external standard, 0.05% in $CDCl_3$, δ_F = –62.61 ppm)[1] was used as reference and spectra were acquired in 5 mm probes at 21 °C. For NMR analyses MestReNova (version 12.0.3) programs were used. For detailed peak assignments, 2D spectra were acquired using Bruker software (^{1}H,^{1}H DFQ-COSY, ^{13}C,^{1}H COSY, ^{1}H,^{1}H NOESY, ^{1}H,^{13}C HMBC). In the ^{1}H,^{1}H NOESY spectra the optimized mixing time, varied from 0.7 s to 0.8 s, was used. The ^{1}H,^{13}C HMBC long-range correlations were acquired for $J_{C,H}$=10 Hz. The standard abbreviation for multiplicities were used (s = singlet, d = doublet, t = triplet, q =quartet, quint = quintet, m = multiplet, sxt = sextet, spt = septet, etc. and dm = doublet of multiplets). Gas chromatography-mass spectrometry (GC-MS) measurements were carried out on a Hewlett-Packard instrument model HP 6890 equipped with a mass detector HP 5973 and on an Agilent 78206b GC system equipped with a mass (Agilent 5977E MSD) and FID detectors. HRMS analyses (ESI+) were performed on a Waters LCT premier XE (TOF) using acetonitrile as solvent. Crude post-reaction mixtures were analyzed by GC-MS and ^{1}H NMR spectroscopy.

n-BuLi (1.6 M in hexane), *sec*-BuLi (1.4 M in cyclohexane), MeLi (3.0 M in diethoxymethane), PhMgCl (1 M in 2-MeTHF), BnMgCl (2.0 M in THF and1.0 M in 2-MeTHF), MeMgCl (3.0 M in THF), metallic magnesium (Mg), 1-(chloromethyl)naphthalene were purchased from Aldrich. *n*-BuLi (2.5 M in hexane), BnMgCl (1.4 M in THF) *i*-PrMgCl (2.0M in THF) were purchased from Acros, 5-bromo-2-methoxypyridine, TMSOTf, NIS, Lawesson's reagent from Fluorchem, NBS form Fluka, *t*-BuOK from TCI and 4-(chloromethyl)-1,1'-biphenyl from Apollo Scientific.

Reactions in tetrahydrofuran (THF) and dimethoxyethane (DME) solutions were performed under argon in flame-dried flasks and liquid components were added from a syringe. Anhydrous toluene and THF were purified by distillation over sodium metal under argon prior to use. NBS was recrystallized from water. Products were purified by flash column chromatography on silica gel (63-200 μm, Merck) using appropriate solvents.

2. Preparation of substrates

Compounds (**2a**, **2b** and **2r**)[2], **2c**[3] and (**2o**, **2p**, **2q**)[4] were prepared earlier.

2a. Preparation of 2-pyridones **1d-1f** and **1j**

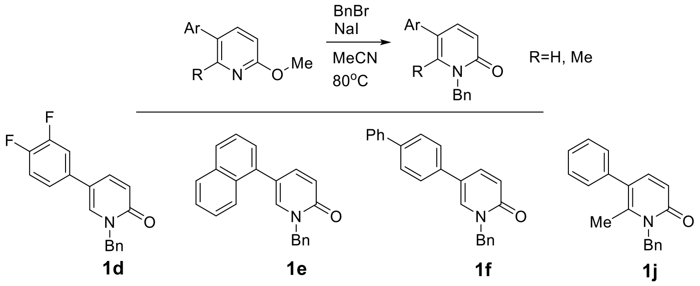

Scheme S1

Compounds **1d-1f, 1j** (Scheme S1) were prepared according to the procedure described earlier,[5] which with some applied modifications is as follows:

The mixture of 5-aryl-2-methoxypyridine, NaI and benzyl bromide was heated in CH_3CN at 80°C for appropriate time (GC-MS control). Subsequently, the mixture was quenched with saturated aqueous ammonium chloride (NH_4Cl), then it was allowed to cool down to rt. The aqueous layer was extracted with ethyl acetate and the combined organic layers were dried over $MgSO_4$. Filtration, concentration *in vacuo* and purification by column chromatography on SiO_2, using a mixture of appropriate solvents yielded **1d** or **1e** or **1f**.

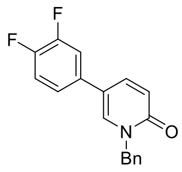

1d

1-Benzyl-5-(3,4-difluorofenylo)pirydyn-2(1*H*)-on (**1d**): Yield 80% (0.858 g). Reagents: 5-(3,4-difluorophenyl)-2-methoxypyridine[3] (0.798 g, 3.607 mmol), NaI (1.08 g, 7.215 mmol), BnBr (1.234 g, 7.215 mmol) in 20 mL of MeCN. Reaction time 10 h. Other: NH_4Cl (sat. 3 mL), ethyl acetate (3 x 30 mL). The crude product purified by column chromatography (SiO_2, *n*-hexane : ethyl acetate, 2:1) gave white solid. M.p. 132-134°C. Spectroscopic data: ^1H NMR ($CDCl_3$, 400 MHz): δ 7.53 (dd, 1H, *J* = 9.4, 2.7 Hz, CH-4), 7.43 (d, 1H, *J* = 2.7 Hz, CH-6), 7.40 – 7.28 (m, 5H, C_6H_5), 7.22 – 7.11 (m, 2H, CH-2', CH-5'), 7.05 (dddd, 1H, *J* = 8.5, 3.9, 2.3, 1.3 Hz, CH-6'), 6.71 (d, 1H, *J* = 9.4 Hz, CH-3), 5.21 (s, 2H, NCH_2). $^{13}C\{H\}$ NMR ($CDCl_3$, 101 MHz): δ 161.8 (C=O), 150.4 (*J* = 249.8, 12.8 Hz), 149.6 (*J* = 249.2, 12.0 Hz) (C-3', C-4'), 138.8 (CH-4), 136.1 (Ph), 134.7 (CH-6), 133.6 (*J* = 6.4, 4.4 Hz, C-1'), 129.0 (2C) (Ph), 128.3, 128.1 (2C), 121.8 (*J* = 6.4, 3.5 Hz, CH-6'), 121.5 (CH-3), 118.6 (C-5), 117.9 (*J* = 17.4 Hz, CH-2'), 114.9 (*J* = 18.2 Hz, CH-5'), 52.3 (NCH_2). GC-MS (EI, 70eV): m/z = 297 (51) [M^+], 296 (23), 191 (13), 151 (10), 91 (100), 65 (12). HRMS (ESI-TOF) m/z: $[M + Na]^+$ Calcd for $C_{18}H_{13}F_2NO$ 320.0863; Found 320.0865.

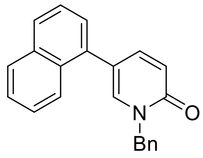

1e

1-Benzyl-5-(naftalen-1-ylo)pirydyn-2(1*H*)-on (**1e**): Yield 95% (1.25 g). Reagents: 2-methoxy-5-(naphthalen-1-yl)pyridine[3] (1 g, 4.25 mmol), NaI (0.967 g, 6.45 mmol), BnBr (0.94 g, 5.5 mmol) in 25 mL of MeCN. Reaction time 24 h. Other: NH_4Cl (sat. 10 mL), ethyl acetate (4 x 75 mL). The crude product purified by column chromatography (SiO_2, *n*-hexane : ethyl acetate, 1:1) gave brown oil. Spectroscopic data: ^{1}H NMR ($CDCl_3$, 400 MHz): δ 7.88 (dd, 1H, *J* = 7.8, 1.5 Hz, ArH), 7.83 (dd, 1H, *J* = 8.3, 1.2 Hz, ArH), 7.78 (dd, 1H, *J* = 8.5, 1.3 Hz, ArH), 7.53 – 7.42 (m, 4H, CH-4, ArH), 7.40 (d, 1H, *J* = 2.5 Hz, CH-6), 7.37 – 7.27 (m, 6H, ArH), 6.74 (d, 1H, *J* = 9.3 Hz, CH-3), 5.22 (s, 2H, NCH_2). ^{13}C{H} NMR ($CDCl_3$, 101 MHz): δ 162.0 (C=O), 142.1 (CH-4), 136.8 (CH-6), 136.3 (Ar), 134.7, 133.9, 131.5, 129.0 (2C) (ArH), 128.6, 128.4, 128.3 (2C), 128.1, 126.9, 126.5, 126.1, 125.4, 125.0, 120.4 (CH-3), 119.6 (C-5), 52.2 (NCH_2). GC-MS (EI, 70eV): m/z = 311 (99) [M^+], 310 (49), 234 (17), 205 (28), 165 (30), 164 (15), 91 (100), 65 (13). HRMS (ESI-TOF) m/z: $[M + H]^+$ Calcd for $C_{22}H_{18}NO$ 312.1388; Found 312.1393.

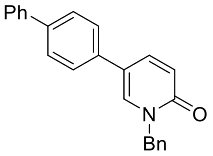

1f

5-([1,1'-Bifenyl]-4-yl)-1-benzylpirydyn-2(1*H*)-on (**1f**): Yield 87% (1.12g). Reagents: 5-([1,1'-Biphenyl]-4-yl)-2-methoxypyridine* (1 g, 3.8 mmol), NaI (0.854 g, 5.7 mmol), BnBr (0.851 g, 4.98 mmol) in 20 mL of MeCN. Reaction time 28 h. Other: NH_4Cl (sat. 10 mL), ethyl acetate (4 x 75 mL). The crude product purified by column chromatography (SiO_2, *n*-hexane : ethyl acetate, 1:1) gave brown solid. M.p. 187-190ºC. Spectroscopic data: ^{1}H NMR ($CDCl_3$, 400 MHz): δ 7.65 (dd, 1H, *J* = 9.4, 2.7 Hz, CH-4), 7.63 – 7.56 (m, 4H, ArH), 7.53 (d, 1H, *J* = 2.7 Hz, CH-6), 7.48 – 7.39 (m, 4H, ArH), 7.38 – 7.27 (m, 6H, ArH), 6.73 (d, 1H, *J* = 9.4 Hz, CH-3), 5.23 (s, 2H, NCH_2). ^{13}C{H} NMR ($CDCl_3$, 101 MHz): δ 162.0 (C=O), 140.3 (Ar), 140.2, 139.3 (CH-4), 136.3 (Ar), 135.3, 134.5 (CH-6), 129.0 (2C) (ArH), 128.9 (2C), 128.1 (3C), 127.7, 127.5 (2C), 127.0 (2C), 126.1 (2C), 121.3 (CH-3), 120.1 (C-5), 52.3 (NCH_2). GC-MS (EI, 70eV): m/z = 337 (48) [M^+], 336 (21), 231 (13), 191 (16), 91 (100), 65 (11). HRMS (ESI-TOF) m/z: $[M + H]^+$ Calcd for $C_{24}H_{20}NO$ 338.1545; Found 338.1547.

***** 5-([1,1'-Biphenyl]-4-yl)-2-methoxypyridine was prepared according to method described earlier[6] with some modifications:

To the mixture of DMF (60 mL) and water (60 mL) in a 250 mL flask 5-bromo-2-methoxypyridine (3 g, 16.0 mmol), [1,1'-biphenyl]-4-ylboronic acid (3.48 g, 17.5 mmol), $K_3PO_4 \cdot 5H_2O$ (3.28 g, 11.9 mmol) and $PdCl_2$ (0.056 g, 0.32 mmol) were added. The resulting solution was stirred for 22 h at room temperature in open flask. After this time brine (100 mL) was added and the aqueous layer was extracted with diethyl ether (4 x 100 mL) and the organic layer was washed with brine and dried with $MgSO_4$.

The mixture was filtered, and the solvents were evaporated under reduced pressure. The crude product purified by column chromatography (SiO_2, $CHCl_3$) gave 3.7 g of white solid (yield 89%). M.p. 159-161ºC. Spectroscopic data: ^{1}H NMR ($CDCl_3$, 400 MHz): δ 8.44 (dd, 1H, *J* = 2.5, 0.7 Hz, CH-6), 7.83 (dd, 1H, *J* = 8.6, 2.5 Hz, CH-4), 7.72 – 7.56 (m, 6H, ArH), 7.51 – 7.41 (m, 2H, ArH), 7.41 – 7.33 (m, 1H, ArH), 6.83 (dd, 1H, *J* = 8.7, 0.7 Hz, CH-3), 3.99 (s, 3H, OCH_3). ^{13}C{H} NMR ($CDCl_3$, 101 MHz): δ 163.7 (C-2), 144.9 (CH-6), 140.5 (Ar), 140.2, 137.3 (CH-4), 136.8 (Ar), 129.6, 128.9 (2C) (ArH), 127.7, 127.4 (2C), 127.03 (2C), 127.00 (2C), 110.9 (CH-3), 53.6 (OCH_3). GC-MS (EI, 70 eV); m/z = 261 (100) [M^+], 260 (66), 232 (38), 230 (17), 191 (15), 189 (15), 165 (6), 115 (7). HRMS (ESI-TOF) m/z: $[M + H]^+$ Calcd for $C_{18}H_{16}NO$ 262.1232; Found 262.1236.

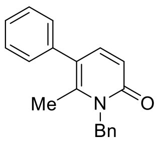

1j

1-Benzyl-6-methyl-5-phenylpyridin-2(1*H*)-one (**1j**): Yield 60% (0.25 g). Reagents: 6-methoxy-2-methyl-3-phenylpyridine* (0.3 g, 1.5 mmol), NaI (0.45 g, 3 mmol), BnBr (0.51 g, 3 mmol). Reaction was conducted 48 h at 110ºC time without solvent. Other: NH_4Cl (sat. 15 mL), ethyl acetate (4 x 60 mL). The crude product purified by column chromatography (SiO_2, *n*-hexane : ethyl acetate, 4:1) gave white solid. M.p. 105-107ºC. Spectroscopic data: ^{1}H NMR ($CDCl_3$, 400 MHz): δ 7.44 – 7.03 (m, 11H, $2C_6H_5$, CH-4), 6.63 (dd, 1H, *J* = 9.4, 0.8 Hz, CH-3), 5.46 (s, 2H, NCH_2), 2.23 (s, 3H, CH_3). ^{13}C{H} NMR ($CDCl_3$, 101 MHz): δ 163.3 (C=O), 143.6 (C-6), 141.8 (CH-4), 139.1 (C_6H_5), 136.5, 129.6 (2C) (C_6H_5), 128.9 (2C), 128.5, 127.3, 127.3 (2C), 126.5 (2C), 120.7 (C-5), 117.5 (CH-3), 47.8 (NCH_2), 18.0 (CH_3). GC-MS (EI, 70eV): m/z = 275 (99) [M^+], 274 (56), 260 (12), 198 (24), 169 (29), 156 (18), 115 (24), 91 (100), 65 (17). HRMS (ESI-TOF) m/z: $[M + H]^+$ Calcd for $C_{19}H_{18}NO$ 276.1388; Found 276.1392.

*Synthesis of 6-methoxy-2-methyl-3-phenylpyridine (according to the procedure descrbed earlier)[6]:

To the mixture of DMF (40 mL) and water (40 mL) in a 250 mL round bottom flask 5-bromo-6-methyl-2-methoxypyridine (2 g, 9.9 mmol), phenylboronic acid (2.41 g, 19 mmol), K_3PO_4 (1.28 g, 7.5 mmol) and $PdCl_2$ (0.086 g, 0.049 mmol) were added. The resulting solution was stirred for 22h at room temperature in an open flask. After this time brine (70 mL) was added, and the mixture was filtered through celite. The aqueous layer was extracted with ethyl acetate (3 x 60 mL) then organic layer was dried with $MgSO_4$. The mixture was filtered and the solvents were evaporated under reduced pressure. The crude product was purified by column chromatography on (SiO_2, *n*-hexane : ethyl acetate, 20:1) gave 1.7 g of 6-methoxy-2-methyl-3-phenylpyridine as colorless oil (76% yield). Spectroscopic data: ^{1}H NMR ($CDCl_3$, 400 MHz): δ 7.45 – 7.38 (m, 3H, CH-4, C_6H_5), 7. 36 – 7.24 (m, 3H, C_6H_5), 6.62 (d, 1H, *J* = 8.3 Hz, CH-3), 3.96 (s, 3H, OCH_3), 2.42 (s, 3H, 6-CH_3). ^{13}C NMR ($CDCl_3$, 101 MHz): δ 162.6 (C-2), 153.2 (C-6), 140.3 (CH-4), 140.2 (C_6H_5), 129.7 (C-5), 129.3 (2C), (C_6H_5), 126.9, 128.3 (2C), 107.3 (CH-3), 53.4 (OCH_3), 23.1 (6-CH_3). GC-MS (EI, 70eV): m/z = 199 (76) [M^+], 198 (100), 170 (37), 168 (31), 154 (12), 115 (25). HRMS (ESI-TOF) m/z: $[M + H]^+$ Calcd for $C_{13}H_{14}NO$ 200.1075; Found 200.1075.

2b. Preparation of compound **1g**

Compound **1g** was prepared according to the sequence:

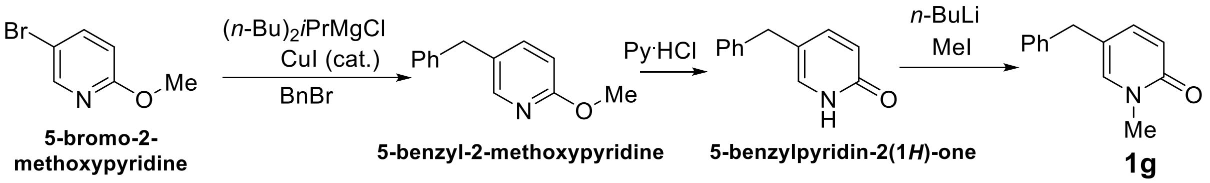

Scheme S2

Synthesis of **5-benzyl-2-methoxypyridine** (according to the procedure descrbed earlier[7] with some modifications):

To a cooled (0°C) and stirred solution of 3.9 mmol of *i*-PrMgCl (1.9 ml, 2.0 M in THF) in dry THF (20 ml) in a Schlenk flask, 7.7 mmol of *n*-BuLi (3.1 ml, 2.5 M in hexane) was added *via* syringe over 1 min under argon and the mixture was stirred for 5 min. To the yellow, cooled (0°C) solution 1.453 g (7.7 mmol) 5-bromo-2-methoxypyridine was added *via* syringe. The resulting solution was stirred for 45 min at 0°C and then 1.976 g (11.55 mmol) of benzyl bromide and 0.147 g (0.77 mmol) of copper(I) iodide was added. The mixture was continuously stirred for 30 minutes at 0°C and 180 minutes at room temperature. After addition of aqueous saturated NH_4Cl (10 mL), the aqueous layer was extracted with ethyl acetate (2 x 75 ml) and the combined organic layers were dried over $MgSO_4$. Filtration, concentration *in vacuo* and purification by flash column chromatography yielded 1.247 g (81%) of 5-benzyl-2-methoxypyridine[8] as yellow oil.

5-Benzylpyridin-2(1*H*)-one[9] was obtained according to the procedure described earlier[10]:

A mixture of 5-benzyl-2-methoxypyridine 0.916 g (4.59 mmol) and pyridine hydrochloride (5.27 g, 45.95 mmol) was heated in a 100-mL flask at 160ºC (oil bath) while continuous stirring for 20 minutes. After cooling to rt. brine (20 mL) was added followed with water (10 mL). White solid was filtered off, dissolved in ethyl acetate and passed through a pad of SiO_2 and washed with ethyl acetate till whole product (0.681 g, yield 80%) was obtained (TLC-control). The crude product purified by crystallization (*n*-hexane : ethyl acetate) gave white solid. M.p. 151-152°C.

Synthesis of **1g**:

To a cooled (0°C) and stirred solution of 5-benzylpyridin-2(1*H*)-one (0.5 g, 2.7 mmol) in anhydrous THF (40 mL) in a 100 mL Schlenk flask equipped with septum and argon balloon, 1.77 mL *n*-BuLi [2.8 mmol, (1.6 M in hexane)] was added from a syringe over a few minute. The mixture was stirred for 5 min. To a cooled (0°C) solution methyl iodide 1.15 g (8.1 mmol) was added from a syringe. The resulting solution was stirred for 10 min. Subsequently, the septum was removed and the flask was equipped with condenser crowned with argon balloon and placed in oil bath at 55°C. The mixture was continuously stirred for 18 hour. After cooling to rt and addition of aqueous saturated NH_4Cl (15 mL), the aqueous layer was extracted with ethyl acetate (3 x 50 mL) and combined organic layers were dried over $MgSO_4$.

Filtration, concentration *in vacuo* and purification by flash column chromatography using ethyl acetate as eluent yielded titled compound as yellow oil (0.504 g, 94%).

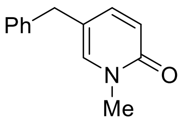

1g

5-Benzyl-1-methylpyridin-2(1*H*)-one[11] (**1g**): ^{1}H NMR ($CDCl_3$, 400 MHz): δ 7.35 – 7.29 (m, 2H, C_6H_5), 7.27 – 7.22 (m, 1H, C_6H_5), 7.19 (dd, 1H, *J* = 9.3, 2.5 Hz, CH-4), 7.18 – 7.14 (m, 2H, C_6H_5), 7.04 (dd, 1H, *J* = 2.5, 0.7 Hz, CH-6), 6.53 (dd, 1H, *J* = 9.3, 0.7 Hz, CH-3), 3.71 (s, 2H, 5-CH_2), 3.51 (s, 3H, NCH_3). ^{13}C{H} NMR ($CDCl_3$, 101 MHz): δ 162.5 (C=O), 141.4 (CH-4), 139.4 (C_6H_5), 136.3 (CH-6), 128.8 (2C), (C_6H_5), 128.7, 126.7 (2C), 120.7 (CH-3), 118.8 (C-5), 37.7 (NCH_3), 37.6 (5-CH_2). GC-MS (EI, 70 eV); m/z = 199 (100) [M^+], 198 (43), 170 (30), 128 (14), 122 (31), 94 (30).

2c. Preparation of compound **1h**

Compound **1h** was prepared according to the sequence:

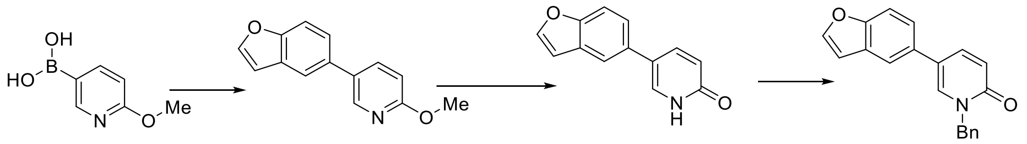

(6-methoxypyridin- -3-yl)boronic acid **5-(benzofuran-5-yl)-2-me- thoxypyridine** **5-(benzofuran-5-yl)py- ridin-2(1*H*)-one** **1h**

Scheme S3

Synthesis of **5-(benzofuran-5-yl)-2-methoxypyridine**:

In the round bottom flask containing 40 mL degassed DMF 5-bromobenzofuran (0.924 g, 4.69 mmol), **6-methoxypyridin-3-yl)boronic acid** (0.861 g, 5.63 mmol) and tetrakis(triphenylphosphino) palladium(0) (0.136 g, 0.117 mmol, 0.025 equiv.) were placed. The mixture was stirred at rt for 60 min. Next 21.5 mL of 1.0 M degassed aqueous Na_2CO_3 solution was added, and the reaction mixture was heated under argon at 80°C for 12 h. Solvent was removed *in vacuo*, ethyl acetate was added, and the organic layer was washed with brine, separated, and dried over $MgSO_4$. The crude product was purified by column chromatography on silica gel using a mixture of *n*-hexane and ethyl acetate (10:1 v/v) as the eluent, yielding 1.005 g of **5-(benzofuran-5-yl)-2-methoxypyridine** as white solid (96% yield). M.p. 55-57°C. Spectroscopic data: ^{1}H NMR ($CDCl_3$, 400 MHz): δ 8.40 (dd, 1H, *J* = 2.6, 0.8 Hz, CH-6), 7.80 (dd, 1H, *J* = 8.6, 2.6 Hz, CH-4), 7.71 (d, 1H, *J* = 1.9 Hz, CH-4'), 7.66 (d, 1H, *J* = 2.2 Hz, CH-2'), 7.56 (dt, 1H, *J* = 8.6, 0.8 Hz, CH-7'), 7.43 (dd, 1H, *J* = 8.6, 1.9 Hz, CH-6'), 6.82 (dd, 1H, *J* = 8.6, 0.8 Hz, CH-3), 6.80 – 6.81 (m, 1H, CH-3'), 3.99 (s, 3H, OCH_3). ^{13}C{H} NMR ($CDCl_3$, 101 MHz): δ 163.4 (C-2), 154.5 (C-7a'), 145.8 (CH-2'), 145.1 (CH-6), 137.8 (CH-4), 133.1, 130.6, 128.1, (C-3a', C-5, C-5'), 123.5 (CH-6'), 119.3 (CH-4'), 111.8 (CH-7'), 110.8 (CH-3), 106.7 (CH-3'), 53.5 (OCH_3). GC-MS (EI, 70 eV); m/z = 225 (100) [M+], 224 (85), 196 (43), 195 (23), 194 (23), 155 (18). HRMS (ESI-TOF) m/z: $[M + H]^+$ Calcd for $C_{14}H_{12}NO_2$ 226.0868; Found 226.0864.

Synthesis of **5-(benzofuran-5-yl)pyridin-2(1*H*)-one** (according to the procedure described earlier)[10]:

A mixture of 5-(benzofuran-5-yl)-2-methoxypyridine (0.692 g, 3.0 mmol) and pyridine hydrochloride (3.44 g, 30.0 mmol) was heated in a 100-mL flask at 160ºC (oil bath) while continuous stirring for 20 minutes. After cooling to rt. brine (15 mL) was added followed with 7 mL of water. White solid was filtered off, dissolved in ethyl acetate and passed through a pad of SiO_2 and washed with ethyl acetate till whole product (0.461 g, yield 71%) was obtained (TLC-control). The crude product purified by crystallization (*i*-PrOH) gave white solid. M.p. 203-205°C. Spectroscopic data: ^{1}H NMR (DMSO-d_6, 400 MHz): δ 11.88 (br s, 1H, NH), 8.03 (d, 1H, *J* = 2.2 Hz, CH-2'), 7.87 (dd, 1H, *J* = 9.5, 2.8 Hz, CH-4), 7.82 (d, 1H, *J* = 2.0 Hz, CH-4'), 7.71 (d, 1H, *J* = 2.8 Hz, CH-6), 7.63 (d, 1H, *J* = 8.6 Hz, CH-7'), 7.49 (dd, 1H, *J* = 8.6, 2.0 Hz, CH-6'), 6.98 (d, 1H, *J* = 2.2 Hz, CH-3'), 6.47 (d, 1H, *J* = 9.5 Hz, CH-3). ^{13}C{H} NMR (101 MHz, DMSO) δ 161.6 (C=O), 153.5 (C-7a'), 146.6 (C-2'), 140.5 (CH-4), 132.5 (CH-6), 131.4 (C-3a'), 127.8 (C-5'), 122.2 (CH-6'), 119.9 (CH-3), 118.4 (C-5), 117.9 (CH-4'), 111.5 (CH-7'), 106.8 (CH-3'). HRMS (ESI-TOF) m/z: $[M + H]^+$ Calcd for $C_{13}H_{10}NO_2$ 212.0712; Found 212.0722.

Synthesis of **1h**:

To a cooled (0°C) and stirred solution of **5-(benzofuran-5-yl)pyridin-2(1*H*)-one** (0.256 g, 1.2 mmol) in anhydrous THF (20 mL) in a 100-mL Schlenk flask equipped with septum and argon balloon, 0.51 mL of *n*-BuLi [1.2 mmol, (2.5 M in hexane)] was added from a syringe over a few minute. The mixture was stirred for 5 min. To a cooled (0°C) solution benzyl bromide 0.267 g (1.56 mmol) was added from a syringe. The resulting solution was stirred for10 min. Subsequently the septum was removed and the flask was equipped with condenser crowned with argon balloon and placed in oil bath at 70°C. The mixture was continuously stirred for 18 hour. After cooling to rt and addition of aqueous saturated NH_4Cl (10 mL), the aqueous layer was extracted with ethyl acetate (2 x 50 mL) and combined organic layers were dried over $MgSO_4$. Filtration, concentration *in vacuo* and purification by flash column chromatography (SiO_2, *n*-hexane : ethyl acetate, 5:1) gave 0.303 g (83%) of **1h** as pale yellow oil.

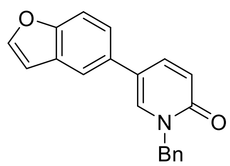

1h

5-(Benzofuran-5-yl)-1-benzylpirydyn-2(1*H*)-on (**1h**): ^{1}H NMR ($CDCl_3$, 400 MHz): δ 7.65 – 7.61 (m, 2H, CH-4, CH-2'), 7.55 (d, 1H, *J* = 1.9 Hz, CH-4'), 7.50 (d, 1H, *J* = 8.6 Hz, CH-7'), 7.47 (d, 1H, *J* = 2.6 Hz, CH-6), 7.36 – 7.28 (m, 5H, C_6H_5), 7.25 (dd, 1H, *J* = 8.6, 1.9 Hz, CH-6'), 6.77 (dd, 1H, *J* = 2.2, 0.9 Hz, CH-3'), 6.72 (d, 1H, *J* = 9.3 Hz, CH-3), 5.23 (s, 2H, NCH_2). ^{13}C{H} NMR ($CDCl_3$, 101 MHz): δ 161.9 (C=O), 154.4 (C-7a'), 145.9 (C-2'), 139.9 (CH-4), 136.4 (C_6H_5), 134.5 (CH-6), 131.6 (C-3a'), 129.0 (2C) (C_6H_5), 128.2 (C-5'), 128.12, 128.08, (2C) (C_6H_5), 122.7 (CH-6'), 121.2 (CH-3), 121.0 (C-5), 118.5 (CH-4'), 111.8 (CH-7'), 106.6 (CH-3'), 52.2 (NCH_2). GC-MS (EI, 70 eV); m/z =

301 (42) [M+], 300 (22), 224 (9), 195 (16), 155 (16), 91 (100), 65 (15). HRMS (ESI-TOF) m/z: [M + H]$^+$ Calcd for $C_{20}H_{16}NO_2$ 302.1181; Found 302.1186.

2d. Preparation of compound **1s**

Compound **1s** was prepared according to the sequence:

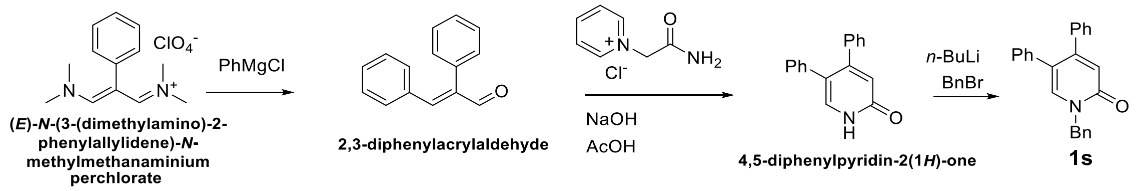

Scheme S4

Synthesis of **2,3-diphenylacrylaldehyde** (according to the procedure descrbed earlier[12]):

To a cooled (0 °C) and stirred solution of (*E*)-*N*-(3-(dimethylamino)-2-phenylallylidene)-*N*-methylmethanaminium perchlorate[13] (4 g, 13.2 mmol) in anhydrous THF (70 mL) in a 250 mL Schlenk flask equipped with septum and argon balloon, PhMgCl [26.4 mL, 26.4 mmol, (1 M in 2-MeTHF)] was added *via* syringe over a few minute. Subsequently the septum was removed and the flask was equipped with condenser crowned with argon balloon and placed in oil bath at 70°C. The mixture was continuously stirred for 3 hours, and then at rt overnight (16 h). After this time the solution was cooled to 0°C and 10% aqueous solution of HCl was added. Reaction mixture was stirred for 30 min. The excess of acid was neutralized with 10% aqueous solution of sodium hydroxide. The aqueous layer was extracted with chloroform (2 x 100 mL) and combined organic layers were dried over $MgSO_4$. Filtration, concentration *in vacuo* and purification by crystallization (MeOH : H_20) yielded 1.859 g of 2,3-diphenylacrylaldehyde as a white solid (68%,). M.p. 90-94°C. (Lit. 93-94°C.[14])

Synthesis of **4,5-diphenylpyridin-2(1*H*)-one** (according to the procedure descrbed earlier[15]):

In a 100 mL single-necked round-bottomed flask equipped with a magnetic stirrer 1-(2-amino-2-oxoethyl)-1-pyridinium chloride (1.243 g, 7.2 mmol) was dissolved in 15 mL of methanol, then 7.2 mL of 1 M NaOH solution was added and stirred for 5 minutes. Subsequently, 2,3-diphenylacrylaldehyde (1 g, 4.8 mmol) was dissolved in 5 mL of methanol and added in one portion maintaining stirring for 20 minutes. After this time 30 mL of acetic acid was added and the solution was stirred for another hour. Subsequently, all of the solvents were distilled off under normal pressure (bath temperature 150°C), following by the addition of water (60 mL) to the cooled flask, placed in an ice-bath. After one hour the resulting oil was extracted with ethyl acetate (2 x 100 mL) and combined organic layers were dried over $MgSO_4$. Filtration, concentration *in vacuo* and purification by crystallized from methanol yielded 4,5-diphenylpyridin-2(1*H*)-one[16] as a white solid (32%, 0.381 g).

Synthesis of 1-benzyl-4,5-diphenylpyridin-2(1*H*)-one (**1s**) (according to the procedure descrbed earlier)[2,10]:

To a cooled (0°C) and stirred solution of 4,5-diphenylpyridin-2(1*H*)-one (0.283 g, 1.16 mmol) in anhydrous THF (20 mL) placed in a 100 mL Schlenk flask equipped with septum and argon balloon, *n*-BuLi (0.46 mL, 1.16 mmol, 2.5 M in hexane)] was added from a syringe over a few minutes. The mixture was stirred for 5 min. To a cooled (0°C) solution benzyl bromide (0.258 g, 1.51 mmol) was added from a syringe. The resulting solution was stirred for10 min. Subsequently, the septum was removed and the flask was equipped with a condenser crowned with argon balloon and placed in oil bath at 70°C. The mixture was continuously stirred for 18 hour. After cooling to rt aqueous saturated NH_4Cl solution (10 mL) was added and the aqueous layer was extracted with ethyl acetate (2 x 50 mL) and combined organic layers were dried over $MgSO_4$. Filtration, concentration *in vacuo* and purification by flash column chromatography (SiO_2, *n*-hexane : ethyl acetate, 3:7) gave 0.336 g (87%) of **1s** as pale yellow oil.

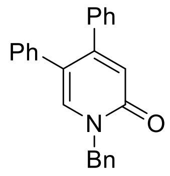

1s

1-Benzyl-4,5-diphenylpyridin-2(1*H*)-one (**1s**): ^{1}H NMR ($CDCl_3$, 400 MHz): δ 7.42 – 7.32 (m, 5H, C_6H_5), 7.31 (s, 1H, CH-6), 7.27 – 7.13 (m, 6H, 2C_6H_5), 7.12 – 7.05 (m, 2H, C_6H_5), 6.96 – 6.92 (m, 2H, C_6H_5), 6.70 (s, 1H, CH-3), 5.24 (s, 2H, NCH_2). ^{13}C{H} NMR (101 MHz, $CDCl_3$) δ 162.0 (C=O), 152.7 (C-4), 137.9 (Ar), 136.9 (CH-6), 136.6 (Ar), 136.4, 129.4 (2C) (ArH), 129.0 (2C), 128.8 (2C), 128.3 (2C), 128.22, 128.17 (2C), 128.14, 128.10 (2C), 127.0, 121.1 (C-5), 120.7 (CH-3), 51.9 (NCH_2). GC-MS (EI, 70 eV); m/z = 337 (55) [M^+], 336 (40), 260 (17), 231 (25), 189 (17), 91 (100), 65 (14). HRMS (ESI-TOF) m/z: [M + H]$^+$ Calcd for $C_{24}H_{20}NO$ 338.1545; Found 338.1542.

2e. Preparation of 6-benzyl-3,6-dihydropyridin-2-ones **2**

6-Benzyl-3,6-dihydropyridin-2-ones **2** were obtained according to the method described earlier.[2] In some reactions regioisomeric derivatives **3** were formed and these compounds were isolated in pure state when yield exceeded 10% (Scheme S5).

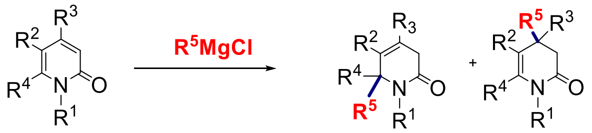

1 **2** **3**

Entry	1/2/3	R¹	R²	R³	R⁴	R⁵	Isolated yield [%] **2, 3**
1	**d**	Bn	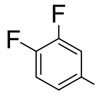	H	H	Bn	87, 9
2	**e**	Bn	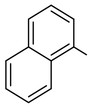	H	H	Bn	75, 0
3	**f**	Bn	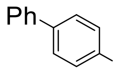	H	H	Bn	75, 0

4	**g**	Me	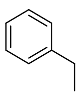	H	H	Bn	77, 19
5	**h**	Bn	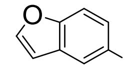	H	H	Bn	87, 0
6	**i**	Bn	Ph	H	H	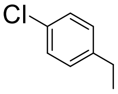	73, 0
7	**j**	Bn	Ph	H	Me	Bn	24, 31
8	**k**	Bn	Ph	H	H	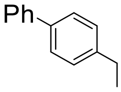	64, 12
9	**l**	Bn	Ph	H	H	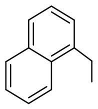	75, 0
10	**n**	Ph	H	Ph	H	Bn	88, 0
11	**s**	Bn	Ph	Ph	H	Bn	93, 0

Scheme S5

General procedure[2]:

A stirred solution of BnMgCl (1.4 mL, 2.8 mmol, 1.35 equiv, 2.0 M in THF or 1.0 M in 2-MeTHF) in dry THF (10 mL) placed in a Schlenk flask (50 mL) was cooled to 0ºC, and *s*-BuLi (4.05 mL, 5.6 mmol, 2.7 equiv, 1.4 M in cyclohexane) was added from a syringe over 3 minutes under argon. The resulting solution was stirred for 5 minutes, and was cooled to -80ºC. The cold solution containing lithium benzyldi(*sec*-butyl) magnesiate and LiCl was transferred by a syringe to a precooled (-80 ºC) solution of *N*-substituted 2-pyridone **1** (2.07 mmol, 1 equiv), in THF (25 mL), prepared in another Schlenk flask (100 mL). The resulting solution was stirred for 120 min at -80 ºC (TLC or GC/MS control). After this time, the mixture was carefully quenched with saturated aqueous NH_4Cl solution (5 mL), then it was allowed to warm up to room temp. and diluted with water (ca. 10 mL). The aqueous layer was extracted with ethyl acetate (3 x 30 mL), and the combined organic layers were dried with $MgSO_4$. The mixture was filtered, and the solvents were evaporated under reduced pressure. The crude product was purified by column chromatography on silica gel using a mixture of appropriate solvents to give the desired product.

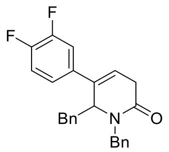

2d

(6*RS*)-1,6-Dibenzyl-5-(3,4-difluorophenyl)-3,6-dihydropyridin-2(1*H*)-one (**2d**): Yield 87%. The crude product purified by column chromatography (SiO_2, *n*-hexane : ethyl acetate, 6:1) gave colorless oil. ^1H NMR ($CDCl_3$, 400 MHz): δ 7.44 – 7.22 (m, 8H, ArH), 7.10 (dt, 1H, *J* = 10.0, 8.4 Hz, ArH), 6.92 – 7.02 (m, 3H, ArH), 6.87 (ddd, 1H, *J* = 8.9, 4.2, 1.9 Hz, ArH), 5.88 (dd, 1H, *J* = 5.8, 2.2 Hz, =CH-4), 5.75 (d, 1H, *J* = 15.2 Hz, NCHH), 4.46 – 4.56 (m, 1H, CH-6), 3.92 (d, 1H, *J* = 15.2 Hz, NCHH), 3.09 (dd, 1H, *J* = 13.8, 4.8 Hz, CHH-6), 2.82

(ddd, 1H, *J* = 21.3, 5.8, 1.1 Hz, CHH-3), 2.62 (dd, 1H, *J* = 13.8, 3.9 Hz, 6-CHH), 1.86 (dt, 1H, *J* = 21.3, 2.6 Hz, CHH-3). ^{13}C{H} NMR (101 MHz, $CDCl_3$) δ 169.0 (C=O), 150.2 (J_{CF}=249.0, 12.7 Hz), (C-3', C-4'), 150.0 (J_{CF}=249.3, 13.3 Hz), 136.7 (=C-5, Ar), 135.26, 135.19, 135.0 (J_{CF}=5.3, 3.7 Hz, C-1'), 130.2 (2C), 128.9 (2C), 128.3 (2C), 127.7 (3C), 127.2 (ArH), 123.2 (=CH-4), 122.1 (J_{CF}=6.2, 3.4 Hz, CH-6'), 117.7 (J_{CF}=17.2 Hz, CH-2'), 115.2 (J_{CF}=18.0 Hz, CH-5'), 59.2 (CH-6), 47.2 (NCH_2), 37.0 (6-CH_2), 32.7 (CH_2-3). ^{19}F{H} NMR (377 MHz, $CDCl_3$) δ -136.45 (d, *J* = 21.2 Hz), -138.07 (d, *J*=21.6 Hz). GC-MS (EI, 70eV): m/z = 389 (1), [M+], 298 (37), 91 (100), 65 (10). HRMS (ESI-TOF) m/z: [M + H]$^+$ Calcd for $C_{25}H_{22}F_2NO$ 390.1669; Found 390.1672.

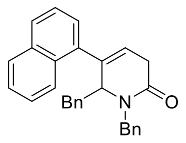

2e

(6*RS*)-1,6-Dibenzyl-5-(naphthalen-1-yl)-3,6-dihydropyridin-2(1*H*)-one (**2e**): Yield 75%. The crude product purified by column chromatography (SiO_2, *n*-hexane : ethyl acetate, 4:1) gave white solid. M.p. 158-160ºC. ^{1}H NMR ($CDCl_3$, 400 MHz): δ 7.78 (ddt, 2H, *J* = 8.1, 4.0, 0.8 Hz, ArH), 7.48 – 7.27 (m, 11H, ArH), 7.17 (dd, 2H, *J* = 7.3, 2.1 Hz, ArH), 7.10 (d, 1H, *J* = 8.5 Hz, ArH), 6.98 (ddd, 1H, *J* = 8.2, 6.8, 1.3 Hz, ArH), 5.83 (d, 1H, *J* = 14.6 Hz, NCHH), 5.78 (dd, 1H, *J* = 5.8, 2.0 Hz, =CH-4), 4.50 (ddd, 1H, *J* = 4.9, 4.4, 2.4 Hz, CH-6), 3.56 (d, 1H, *J* = 14.6 Hz, NCHH), 3.07 (dd, 1H, *J* = 13.9, 4.9 Hz, 6-CHH), 2.88 (ddd, 1H, *J* = 21.0, 5.8, 0.8 Hz, CHH-3), 2.66 (dd, 1H, *J* = 13.9, 4.4 Hz, 6-CHH), 2.06 (dt, 1H, *J* = 21.0, 2.4, 2.0 Hz, CHH-3). ^{13}C{H} NMR ($CDCl_3$, 101 MHz): δ 169.5 (C=O), 137.7, 137.3, 136.9, 136.1, 133.6, 131.1 (=C-5, Ar), 130.3 (2C), 128.9 (2C), 128.8 (2C), 128.40 (2C), 128.37, 128.3, 127.8, 127.1, 126.9, 126.1, 125.8, 125.3 (ArH), 125.1 (=CH-4), 124.9 (ArH), 61.0 (CH-6), 47.1 (NCH_2), 37.1 (6-CH_2), 33.2 (CH_2-3). GC-MS (EI, 70eV): m/z = 403 (1) [M$^+$], 313 (12), 312 (49), 165 (9), 91 (100), 65 (8). HRMS (ESI-TOF) m/z: [M + H]$^+$ Calcd for $C_{29}H_{26}NO$ 404.2014; Found 404.2023.

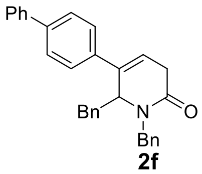

2f

(6*RS*)-5-([1,1'-Biphenyl]-4-yl)-1,6-dibenzyl-3,6-dihydropyridin-2(1*H*)-one (**2f**): The crude product purified by column chromatography (SiO_2, *n*-hexane : ethyl acetate, 4:1) gave yellow solid. M.p. 160-162ºC. ^{1}H NMR ($CDCl_3$, 400 MHz): δ 7.64 – 7.51 (m, 4H, ArH), 7.47 – 7.22 (m, 13H, ArH), 7.08 – 6.95 (m, 2H, ArH), 5.97 (dd, 1H, *J* = 5.8, 2.6 Hz, =CH-4), 5.77 (d, 1H, *J* = 15.3 Hz, NCHH), 4.70 (dd, 1H, *J* = 4.6, 3.9 Hz, CH-6), 3.95 (d, 1H, *J* = 15.3 Hz, NCHH), 3.13 (dd, 1H, *J* = 13.7, 4.6 Hz, 6-CHH), 2.82 (ddd, 1H, *J* = 21.0, 5.8, 1.2 Hz, CHH-3), 2.69 (dd, 1H, *J* = 13.7, 3.9 Hz, 6-CHH), 1.84 (dt, 1H, *J* = 21.2, 2.6 Hz, CHH-3). ^{13}C{H} NMR (101 MHz, $CDCl_3$) δ 169.40 (C=O), 140.8 140.3, 136.9, 136.6, 136.2, 135.6, 130.3 (2C), 128.9 (5C), 128.2 (2C), 127.7 (2C), 127.5, (3C), 127.1, 127.0 (2C), 126.5 (2C), (Ar, ArH, =C-5), 122.1 (=CH-4), 59.2 (CH-6), 47.2 (NCH_2), 37.0 (6-CH_2), 32.8 (CH_2-3). GC-MS (EI, 70eV): m/z = 429 (2) [M$^+$], 338 (40), 91 (100), 65 (7). HRMS (ESI-TOF) m/z: [M + H]$^+$Calcd for $C_{31}H_{28}NO$ 430.2171; Found 430.2171.

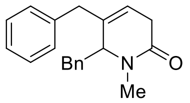

2g

(6*RS*)-5,6-Dibenzyl-1-methyl-3,6-dihydropyridin-2(1*H*)-one (**2g**): Yield 77%. The crude product purified by column chromatography (SiO_2, *n*-hexane : ethyl acetate, 1:2) gave colorless oil. ^{1}H NMR ($CDCl_3$, 400 MHz): δ 7.33 (tt, *J* = 6.9, 2H, 1.0 Hz, ArH), 7.22 – 7.28 (m, 4H, ArH), 7.18 (dd, 2H, *J* = 7.0, 1.8 Hz, ArH), 7.07 – 7.01 (m, 2H, ArH), 5.35 (ddt, 1H, *J* = 5.2, 2.2, 1.5 Hz, =CH-4), 3.86 (dddd, 1H, *J* = 4.9, 3.4, 3.2, 1.5 Hz, CH-6), 3.52 (d, 1H, *J* = 15.5 Hz, 5-CHH), 3.33 (ddt, 1H, *J* = 15.5, 3.2, 1.5 Hz, 5-CHH), 3.04 (dd, 1H, *J* = 13.8, 4.9 Hz, 6-CHH), 2.98 (s, 3H, NCH_3), 2.89 (dd, 1H, *J* = 13.8, 3.4 Hz, 6-CHH), 2.52 (ddt, 1H, *J* = 21.1, 5.2, 1.5 Hz, CHH-3), 1.71 (dt, 1H, *J* = 21.1, 2.3 Hz, CHH-3). $^{13}C\{H\}$ NMR (101 MHz, $CDCl_3$) δ 169.3 (C=O), 138.2 135.5, 134.9, (Ar, =C-5), 130.0 (2C), 128.9 (2C), 128.7 (2C), 128.2 (2C), 127.0, 126.7 (ArH), 122.0 (=CH-4), 63.2 (CH-6), 40.3 (5-CH_2), 36.8 (6-CH_2), 33.3 (NCH_3), 32.1 (CH_2-3). GC-MS (EI, 70eV): m/z = 291 (<1) [M^+], 200 (100), 169 (7), 128 (7), 91 (70), 65 (10). HRMS (ESI-TOF) m/z: $[M + Na]^+$ Calcd for $C_{20}H_{21}NNaO$ 314.1521; Found 314.1525.

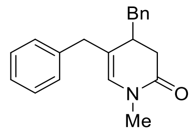

3g

(4*RS*)-4,5-Dibenzyl-1-methyl-3,4-dihydropyridin-2(1*H*)-one (**3g**): The crude product purified by column chromatography (SiO_2, *n*-hexane : ethyl acetate, 1:1) gave pale brown oil. ^{1}H NMR ($CDCl_3$, 400 MHz): δ 7.88 – 6.71 (m, 10H, $2C_6H_5$), 5.76 (t, 1H, *J* =1.4 Hz, =CH-6), 3.28 (d, 1H, *J* = 15.4 Hz, 5-CHH), 3.12 (dd, 1H, *J* = 15.4, 1.7 Hz, 5-CHH), 2.99 (s, 3H, NCH_3), 2.69 (dd, 1H, *J* = 13.2, 5.6 Hz, 4-CHH), 2.48 (dd, 1H, *J* = 13.2, 8.6 Hz, 4-CHH), 2.28 – 2.44 (m, 3H, CH_2-3, CH-4). $^{13}C\{H\}$ NMR ($CDCl_3$, 101 MHz): δ 168.3 (C=O), 139.1, 139.0 (Ar), 129.3 (2C), 128.9 (2C), 128.5 (2C), 128.3 (2C), 126.8, 126.5, 126.4 (ArH), 122.7 (=C-5), 38.9 (5-CH_2), 38.1 (4-CH_2), 36.7 (CH-4), 35.8 (CH_2-3), 33.4 (NCH_3). GC-MS (EI, 70eV): m/z = 291 (4) [M^+], 200 (100), 169 (8), 91 (54). HRMS (ESI-TOF) m/z: $[M + H]^+$ Calcd for $C_{20}H_{22}NO$ 292.1708; Found 292.1708.

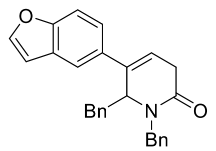

2h

(6*RS*)-5-(Benzofuran-5-yl)-1,6-dibenzyl-3,6-dihydropyridin-2(1*H*)-one (**2h**): Yield 87%. The crude product purified by column chromatography (SiO_2, *n*-hexane : ethyl acetate, 3:1) gave pale yellow oil. ^{1}H NMR ($CDCl_3$, 400 MHz): δ 7.64 (d, 1H, *J* = 2.2 Hz, CH-2'), 7.46 (dt, 1H, *J* = 8.7, 0.8 Hz, CH-7'), 7.42 – 7.23 (m, 9H, CH-4', 2 x C_6H_5), 7.13 (dd, 1H, *J* = 8.6, 1.9 Hz, CH-6'), 7.06 – 7.00 (m, 2H, C_6H_5), 6.75 (dd, 1H, *J* = 2.2, 1.0 Hz, CH-3'), 5.87 (dd, 1H, *J* = 5.7, 2.3 Hz, =CH-4), 5.78 (d, 1H, *J* = 15.2 Hz, NCHH), 4.77 – 4.61 (m, 1H, CH-6), 3.94 (d, 1H, *J* = 15.2 Hz, NCHH), 3.10 (dd, 1H, *J* = 13.7, 4.7 Hz, 6-CHH), 2.81 (ddd, 1H, *J* = 21.1, 5.7, 1.2 Hz, CHH-3), 2.64 (dd, 1H, *J* = 13.7, 3.9 Hz, 6-CHH), 1.84 (dt, 1H, *J* = 21.1, 2.3 Hz, CHH-3). $^{13}C\{H\}$ NMR (101 MHz, $CDCl_3$) δ 169.6 (C=O), 154.7 (C7a'), 145.8 (CH-2'), 137.0 (2C), 135.7, 133.1, (C_6H_5, C-5, C-5'), 130.3 (2C), 128.9 (2C), 128.2 (2C), (C_6H_5), 127.9 (C-3a),

127.7 (2C), 127.5, 127.1 (C_6H_5), 122.9 (CH-6’), 121.8 (=CH-4), 118.9 (CH-4’), 111.7 (CH-7’), 106.7 (CH-3’), 60.0 (CH-6), 47.2 (NCH_2), 36.8 (6-CH_2), 32.8 (CH_2-3). GC-MS (EI, 70 eV); m/z = 393 (1) [M^+], 302 (41), 91 (100), 65 (7). HRMS (ESI-TOF) m/z: $[M + H]^+$ Calcd for $C_{27}H_{24}NO_2$ 394.1807; Found 394.1818.

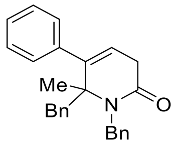
2j

(6*RS*)-1,6-dibenzyl-6-methyl-5-phenyl-3,6-dihydropyridin-2(1*H*)-one (**2j**): Yield 24%. The crude product purified by column chromatography (SiO_2, *n*-hexane : ethyl acetate, 8:1, then 4:1) gave white solid. M.p. 54-55°C. ^{1}H NMR (400 MHz, $CDCl_3$) δ 7.50 – 7.07 (m, 15H, $3C_6H_5$), 5.65 (dd, 1H, *J* = 5.5, 2.3 Hz, =CH-4), 5.56 (d, 1H, *J* = 16.0 Hz, NCHH), 4.18 (d, 1H, *J* = 16.0 Hz, NCHH), 3.16 (d, 1H, *J* = 14.1 Hz, 6-CHH), 3.09 (d, 1H, *J* = 14.1 Hz, 6-CHH), 2.73 (dd, 1H, *J* = 21.8, 5.5 Hz, CHH-3), 1.64 (dd, 1H, *J* = 21.9, 2.3 Hz, CHH-3), 1.20 (s, 3H, 6-CH_3). ^{13}C{H} NMR ($CDCl_3$, 101 MHz): δ 170.4 (C=O), 141.1, 140.5, 139.3, 135.9, (Ar, =C-5), 130.7 (2C), 129.1 (2C), 128.4 (2C), 128.3 (2C), 128.2 (2C), 127.4, 127.3, 126.6 (2C), 126.5, (ArH), 124.9 (=CH-4), 65.9 (C-6), 45.9 (NCH_2), 44.3 (6-CH_2), 32.4 (CH_2-3), 27.3 (6-CH_3). GC-MS (EI, 70eV): m/z = 367 (<1) [M^+], 276 (94), 91 (100), 65 (8). HRMS (ESI-TOF) m/z: $[M + H]^+$ Calcd for $C_{26}H_{26}NO$ 368.2014; Found 368.2010.

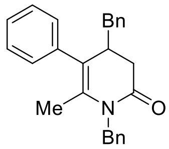
3j

(4*RS*)-1,4-Dibenzyl-6-methyl-5-phenyl-3,4-dihydropyridin-2(1*H*)-one (**3j**): Yield 31%. The crude product purified by column chromatography (SiO_2, *n*-hexane : ethyl acetate, 8:1, then 4:1) gave white solid. M.p. 118-119°C. ^{1}H NMR ($CDCl_3$, 400 MHz): δ 7.39 – 7.08 (m, 13H, ArH), 7.03 – 6.96 (m, 2H, ArH), 5.27 (d, 1H, *J* = 16.0 Hz, NCHH), 4.68 (d, 1H, *J* = 16.0 Hz, NCHH), 2.81 – 2.67 (m, 3H, CHH-3, 4-CHH, CH-4), 2.60 (d, 1H, *J* = 13.7 Hz, 4-CHH), 2.47 (dd, 1H, *J* = 13.3, 10.2 Hz, CHH-3), 1.83 (s, 3H, 6-CH_3). ^{13}C{H} NMR ($CDCl_3$, 101 MHz): δ 169.7 (C=O), 140.3 (=C-6), 139.0, 138.6, 131.8, (Ar), 129.6 (2C), 129.3 (2C), 128.7 (2C), 128.3 (2C), 128.3 (2C), 127.1, 127.0 (2C), 126.8, 126.2, (ArH), 123.4 (=C-5), 45.2 (NCH_2), 39.9 (CH-4), 37.2 (CH_2-3), 35.6 (4-CH_2), 16.9 (CH_3). GC-MS (EI, 70eV): m/z = 367 (<1) [M^+], 276 (94), 91 (100), 65 (8). HRMS (ESI-TOF) m/z: $[M + H]^+$ Calcd for $C_{26}H_{26}NO$ 368.2014; Found 368.2017.

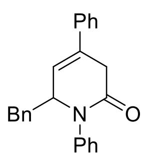
2n

(6*RS*)-6-Benzyl-1,4-diphenyl-3,6-dihydropyridin-2(1*H*)-one (**2n**): Yield 89%. The crude product purified by column chromatography (SiO_2, *n*-hexane : ethyl acetate, 2:1) gave white solid 147-149 ^{o}C. ^{1}H NMR (400 MHz, $CDCl_3$) δ 7.57 – 7.47 (m, 2H, C_6H_5), 7.22 – 7.45 (m, 11H, $2C_6H_5$), 7.15 – 7.06 (m, 2H, C_6H_5), 6.13 (dd, 1H, *J* = 4.7, 2.7 Hz, =CH-5), 4.89 – 4.75 (m, 1H, CH-6), 3.28 (dd, 1H, *J* = 20.7, 2.3 Hz, CHH-3), 2.99 (dd, 1H, *J* = 13.2, 3.6 Hz, 6-CHH), 2.91 (ddd, 1H, *J* = 20.7, 3.6, 2.8 Hz, CHH-3), 2.82 (dd, 1H, *J* = 13.2, 7.8 Hz, 6-CHH). ^{13}C{H} NMR ($CDCl_3$, 101 MHz): δ 168.1 (C=O), 140.7, 138.2, 136.1, 134.1, ($3C_6H_5$, =C-4), 129.9

(2C), 129.4 (2C), 128.7 (2C), 128.3 (2C), 128.1, 127.9 (2C), 127.4, 126.8, 125.1 (2C), (3C_6H_5), 120.8 (=CH-5), 63.1 (CH-6), 40.7 (6-CH_2), 35.0 (CH_2-3). GC-MS (EI, 70eV): m/z = 339 (<1) [M^+], 248 (100), 207 (16), 115 (12), 91 (11), 77 (13). HRMS (ESI-TOF) m/z: $[M + H]^+$ Calcd for $C_{24}H_{22}NO$ 340.1701; Found 340.1703.

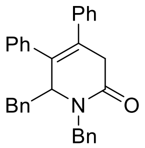

2s

(6*RS*)-1,6-Dibenzyl-4,5-diphenyl-3,6-dihydropyridin-2(1*H*)-one (**2s**): Yield 93% The crude product purified by column chromatography (SiO_2, *n*-hexane : ethyl acetate, 5:1) gave white solid 159-161 ºC. ^1H NMR ($CDCl_3$, 400 MHz): δ 7.47 – 7.39 (m, 2H, C_6H_5), 7.39 – 7.29 (m, 6H, 2 C_6H_5), 7.18 – 7.12 (m, 5H, C_6H_5), 7.11 – 7.05 (m, 3H, C_6H_5), 6.91 – 6.85 (m, 2H, C_6H_5), 6.76 – 6.69 (m, 2H, C_6H_5), 5.78 (d, 1H, *J* = 15.1 Hz, NCH<u>H</u>), 4.64 – 4.44 (m, 1H, CH-6), 3.92 (d, 1H, *J* = 15.1 Hz, NC<u>H</u>H), 3.04 (dd, 1H, *J* = 13.7, 4.7 Hz, 6-CH<u>H</u>), 2.77 (dd, 1H, *J* = 20.0, 0.7 Hz, CH<u>H</u>-3), 2.63 (dd, 1H, *J* = 13.7, 3.8 Hz, 6-C<u>H</u>H), 2.16 (dd, 1H, *J* = 20.0, 2.7 Hz, C<u>H</u>H-3). $^{13}C\{H\}$ NMR ($CDCl_3$, 101 MHz): δ 169.8 (C=O), 139.6, 138.3, 136.9, 135.9, 132.9, 132.8, (=C-4, =C-5, 4C_6H_5), 130.5 (2C), 129.8 (2C), 128.9 (2C), 128.4 (2C), 128.3 (2C), 128.0 (2C), 127.9 (2C), 127.8 (2C), 127.6, 127.3, 127.2, 126.9, (4C_6H_5), 62.6 (CH-6), 47.0 (NCH_2), 37.8 (CH_2-3), 36.2 (6-CH_2). GC-MS (EI, 70eV): m/z = 429 (<1) [M^+], 338 (45), 91 (100). HRMS (ESI-TOF) m/z: $[M + H]^+$ Calcd for $C_{31}H_{28}NO$ 430.2171; Found 430.2169.

2f. Synthesis of compounds **2i**, **2k**/**3k** and **2l**

Part I. Synthesis of Grignard reagents A, B and C:

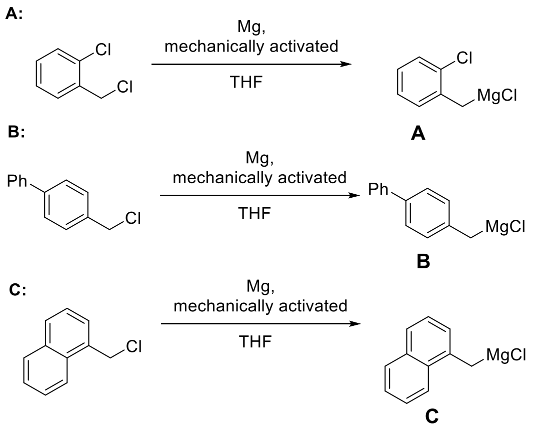

Scheme S6

The Schlenk flask (**A**: 250 mL, **B**: 100 mL, C: 250 mL) equipped with a 3 cm (**A**, **C**), 2 cm (**B**) long stir bar and with an argon balloon was heated twice at 500ºC with heating gun, then metallic magnesium (**A**: 4.53 g, 0.186 mol, **B**: 2.101 g, 0.0104 mol, **C**: 7 g, 0.29 mmol), finely broken porcelain (of the same weight as magnesium) was added and heated again 3 times in short time intervals and then vigorously constantly stirred for 15 h (**A**, **B**) 0.5 h (**C**) to grind the magnesium. (In the case of reaction C 0.12 g of iodine was added and stirred 1h with heating until the violet color is missing.) Subsequently, dry THF (**A**: 156 mL **B**: 52 mL, **C**: 150 mL) was added under argon. In a second Schlenk flask a solution of [**A**:

1-(chloromethyl)-1-chlorobenzene (5 g, 0.0311 mol) in 10 mL of dry THF; **B**: 4-(chloromethyl)-1,1'-biphenyl (2.1 g, 0.01 mol) in 7 mL of dry THF; **C**: 1-(chloromethyl)naphthalene (8.45 g, 0.048 mol) in 10 mL of dry THF) was prepared and added dropwise from a syringe to a suspension of Mg in THF (prepared in the first flask) over 120 minutes constantly stirring at a speed of 500 rpm and after this time was allowed to stand for 30 minutes. A clear Grignard solution was collected by a syringe from above the unreacted Mg and transfer into another Schlenk flask under argon. The clear solution titrated using a method described in literature,[17] indicated the following molarity: 0.067 M (**A**, **B**), 0.05 M (**C**).

Part II. Synthesis of **2i**, **2k/3k** and **2l** by addition of Grignard reagents **A**, **B** and **C** to appropriate 2-pirydone:

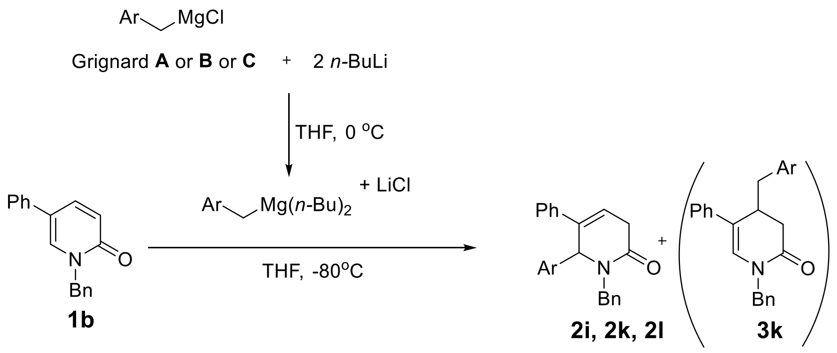

Scheme S7

In a 100 mL Schlenk flask equipped with an argon filled balloon, a solution of the previously prepared Grignard reagent [**A** (43 mL, 2.9 mmol, 0.067 M, 1.2 equiv), **B** (35 mL, 2.1 mmol, 0.067 M, 1.5 equiv), **C** (46 mL, 2.3 mmol, 0.05M, 1.2 equiv)], and *n*-BuLi [**A** (3.6 mL, 5.8 mmol, 1.6 M in hexane 2.4 equiv), **B** (1.7 mL, 4.2 mmol, 2.5 M in hexane, 3.0 equiv), **C** (1.85 mL, 4.6 mol, 2.5 M in hexane, 2.4 equiv)] was stirred at 0°C for 10 min, and then it was cooled to -80ºC. A solution of the obtained benzylmagnesiate reagent was then transferred to a precooled (-80ºC) solution of 1-benzyl-5-phenylpyridin-2(1*H*)-one [**A**: (0.64 g, 2.45 mmol in 12 mL of dry THF), **B**: (0.363 g, 1.4 mmol in 8 mL of dry THF, **C**: (0.5 g, 1.9 mmol in 20 mL of dry THF)]. The reaction was carried out for 120 min (**A**), 60 min (**B**) or 45 min (**C**) at -80ºC. After this time, the mixture was quenched with saturated aqueous ammonium chloride (NH_4Cl, 12 mL), then it was allowed to warm up to rt. The aqueous layer was extracted with ethyl acetate (3 x 75 mL), and the combined organic layers were dried with $MgSO_4$. The mixture was filtered, and the solvents were evaporated under reduced pressure. The crude products were purified by column chromatography on silica gel using a mixture of appropriate solvents to give the desired product.

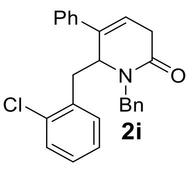

2i

(6*RS*)-1-Benzyl-6-(2-chlorobenzyl)-5-phenyl-3,6-dihydropyridin-2(1*H*)-one (**2i**): Yield 76% (0.691 g). The crude product purified by column chromatography (SiO_2, *n*-hexane : ethyl acetate, 1:1) gave pale yellow semisolid. ^{1}H NMR ($CDCl_3$, 400 MHz): δ 7.35 – 7.04 (m, 14H, ArH), 6.07 (dd, 1H, *J* = 6.0, 2.3 Hz, =CH-4), 5.59 (d, 1H, *J* = 15.3 Hz, NCHH), 4.73 (ddd, 1H, *J* = 6.6, 5.3, 2.3 Hz, CH-6), 3.53 (d, 1H, *J* = 15.3 Hz, NCHH), 3.20 (dd, 1H, *J* = 13.6, 5.3 Hz, 6-CHH), 3.12 (dd, 1H, *J* = 20.7, 6.0 Hz, CHH-3), 2.96 (dd, 1H, *J* = 13.6, 6.6 Hz, 6-CHH), 2.67 (dt, 1H, *J* = 20.7, 2.3 Hz, CHH-3). ^{13}C{H} NMR ($CDCl_3$, 101 MHz): δ 169.29 (C=O), 139.1 137.4, 137.0, 134.6, 134.4, (=C-5, Ar), 132.4, 129.5, 128.7 (2C), 128.6 (2C), 128.6, 127.9 (2C), 127.6, 127.5, 126.7, 125.8 (2C), (ArH), 121.4 (=CH-4), 58.3 (CH-6), 48.3 (NCH_2), 36.7 (6-CH_2), 33.3 (CH_2-3). GC-MS (EI, 70eV): m/z = 387 (1) [M^+], 262 (47), 125 (7), 91 (100), 65 (7). HRMS (ESI-TOF) m/z: $[M + H]^+$ Calcd for $C_{25}H_{23}ClNO$ 388.1468; Found 388.1461.

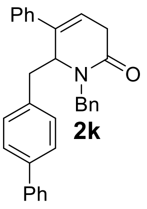

2k

(6*RS*)-6-([1,1'-Biphenyl]-4-ylmethyl)-1-benzyl-5-phenyl-3,6-dihydropyridin-2(1*H*)-one (**2k**): Yield 64% (0.384 g). The crude product purified by column chromatography (SiO_2, *n*-hexane : ethyl acetate, 12:1) gave pale brown semisolid. ^{1}H NMR ($CDCl_3$, 400 MHz): δ 7.70 (d, 2H, *J* = 7.7 Hz, ArH), 7.62 (d, 2H, *J* = 7.8 Hz, ArH), 7.52 (t, 2H, *J* = 7.6 Hz, ArH), 7.48 – 7.35 (m, 9H, ArH), 7.31 (d, 2H, *J* = 7.4 Hz, ArH), 7.18 (d, 2H, *J* = 7.8 Hz, ArH), 5.99 (dd, 1H, *J* = 5.7, 2.4 Hz, =CH-4), 5.90 (d, 1H, *J* = 15.3 Hz, NCHH), 4.80 (ddd, 1H, *J* = 4.6, 3.7, 2.4 Hz, CH-6), 4.10 (d, 1H, *J* = 15.3 Hz, NCHH), 3.21 (dd, 1H, *J* = 13.8, 4.6 Hz, 6-CHH), 2.92 (dd, 1H, *J* = 21.2, 5.7 Hz, CHH-3), 2.76 (dd, 1H, *J* = 13.8, 3.7 Hz, 6-CHH), 2.03 (dt, 1H, *J* = 21.2, 2.4 Hz, CHH-3). ^{13}C{H} NMR ($CDCl_3$, 101 MHz): δ 169.5 (C=O), 140.7, 140.0, 137.9, 137.1, 136.8, 134.8, (=C-5, Ar), 130.9 (2C), 129.0 (2C), 128.98 (2C), 128.95 (2C), 128.1, 127.8 (2C), 127.7, 127.5, 127.1 (2C), 126.8 (2C), 126.3 (2C), (ArH), 122.2 (=CH-4), 59.5 (CH-6), 47.3 (NCH_2), 36.7 (6-CH_2), 33.0 (CH_2-3). GC-MS (EI, 70 eV); m/z = 429 (2) [M^+], 262 (84), 167 (10), 91 (100). HRMS (ESI-TOF) m/z: $[M + H]^+$ Calcd for $C_{31}H_{28}NO$ 430.2171; Found 430.2170.

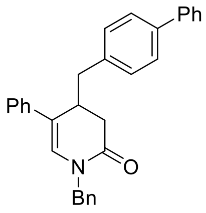

3k

(4*RS*)-4-([1,1'-Biphenyl]-4-ylmethyl)-1-benzyl-5-phenyl-3,4-dihydropyridin-2(1*H*)-one (**3k**): Yield 12% (0.068 g).The crude product purified by column chromatography (SiO_2, *n*-hexane : ethyl acetate, 12:1) gave pale yellow oil. ^{1}H NMR ($CDCl_3$, 400 MHz): δ 7.52 – 7.46 (m, 2H, ArH), 7.44 – 7.40 (m, 2H, ArH), 7.35 (dd, 2H, *J* = 8.2, 7.0 Hz, ArH), 7.30 – 7.18 (m, 11H, ArH), 7.14 (d, 2H, *J* = 8.3 Hz, ArH), 6.39 (s, 1H, =CH-6), 4.71 (d, 1H, *J* = 14.9 Hz, NCHH), 4.64 (d, 1H, *J* = 14.9 Hz, NCHH), 3.09 – 2.99 (m, 1H, CH-4), 2.80 (dd, 1H, *J* = 13.6, 4.2 Hz, 4-CHH), 2.67 – 2.55 (m, 2H, CH_2-3), 2.49 (dd, 1H, *J* = 13.6, 10.3 Hz, 4-CHH). ^{13}C{H} NMR ($CDCl_3$, 101 MHz): δ 167.3 (C=O), 139.9, 138.4, 136.7, 136.10, 136.06, (Ar), 128.8 (2C), 127.8 (2C), 127.76 (2C), 127.73 (3C),

126.8 (2C), 126.7, 126.1 (2C), 126.0 (2C), 125.9, (ArH), 124.5 (=CH-6), 123.9 (2C) (ArH), 121.7 (=C-5), 48.1 (NCH_2), 36.6 (4-CH_2), 35.6 (CH-4), 34.1 (CH_2-3). GC-MS (EI, 70 eV); m/z = 429 (3) [M^+], 262 (71), 167 (10), 91 (100). HRMS (ESI-TOF) m/z: $[M + H]^+$ Calcd for $C_{31}H_{28}NO$ 430.2171; Found 430.2172.

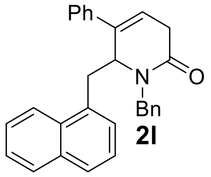
2l

(6*RS*)-1-Benzyl-6-(naphthalen-1-ylmethyl)-5-phenyl-3,6-dihydropyridin-2(1*H*)-one (**2l**): Yield 75% (0.58 g). The crude product purified by column chromatography (SiO_2, *n*-hexane : ethyl acetate, 3:2) gave yellow oil. ^{1}H NMR ($CDCl_3$, 400 MHz): δ 7.85 (dd, 1H, *J* = 8.1, 1.3 Hz, ArH), 7.77 (d, 1H, *J* = 8.2 Hz, ArH), 7.61 (d, 1H, *J* = 8.4 Hz, ArH), 7.48 (ddd, 1H, *J* = 8.1, 6.8, 1.2 Hz, ArH), 7.38 (ddd, 2H, *J* = 8.6, 6.8, 1.8 Hz, ArH), 7.30 – 7.16 (m, 7H, ArH), 7.06 (dd, 2H, *J* = 6.9, 2.8 Hz, ArH), 6.93 – 6.86 (m, 2H, ArH), 5.92 (dd, 1H, *J* = 6.1, 2.2 Hz, =CH-4), 5.49 (d, 1H, *J* = 15.1 Hz, NCH<u>H</u>), 4.76 (dt, 1H, *J* = 7.3, 4.4, 2.2 Hz, CH-6), 3.46 (dd, 1H, *J* = 14.1, 4.4 Hz, 6-CH<u>H</u>), 3.25 (dd, 1H, *J* = 14.1, 7.3 Hz, 6-C<u>H</u>H), 3.20 (d, 1H, *J* = 15.1 Hz, NC<u>H</u>H), 3.04 (dd, 1H, *J* = 20.8, 6.1 Hz, CH<u>H</u>-3), 2.52 (dt, 1H, *J* = 20.8, 2.2 Hz, C<u>H</u>H-3). ^{13}C{H} NMR ($CDCl_3$, 101 MHz): δ 169.4 (C=O), 139.4, 137.6, 136.8, 133.7, 132.9, 132.2, (=C-5, Ar), 128.8, 128.7 (2C), 128.6 (3C), 128.0, 127.9, 127.7 (2C), 127.4, 126.0 (2C), 125.9, 125.7, 125.5, 123.6, (ArH), 121.2 (=CH-4), 59.1 (CH-6), 48.2 (NCH_2), 36.0 (6-CH_2), 33.4 (CH_2-3). GC-MS (EI, 70eV): m/z = 403 (1) [M^+], 312 (49), 165 (9), 91 (100), 65 (8). HRMS (ESI-TOF) m/z: $[M + H]^+$ Calcd for $C_{29}H_{26}NO$ 404.2010; Found 404.2014.

2g. Synthesis of 1,3,3,6-tetrabenzyl-5-phenyl-3,6-dihydropyridin-2(1*H*)-one (**2m**)

Compound **2m** was prepared according to the procedure described earlier[2]:

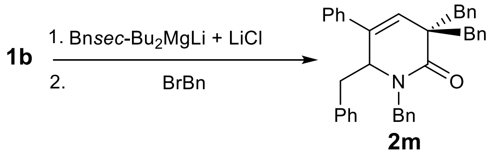

Scheme S8

A stirred solution of BnMgCl (2.8 mL, 1.8 mmol, 1.4 M in THF, 1.35 equiv) in dry THF (10 mL) in a Schlenk flask was cooled to 0ºC under argon, and *s*-BuLi (2.8 mL, 3.7 mmol, 1.3 M in cyclohexane, 2.7 equiv) was added from a syringe over 5 minutes. The resulting solution was stirred for 5 minutes, and then it was cooled to -80ºC. The solution containing lithium benzyldi(*sec*-butyl)magnesate and LiCl was then transferred by syringe to a precooled (-80ºC) solution of 1-benzyl-5-phenylpyridin-2(1*H*)-one (0.355 g, 1.38 mmol) in dry THF (25 ml) . The reaction was carried out for 45 min at -80 ºC. After this time BnBr (0.58 g, 0.0339 mmol, 0.4 ml, 2.5 equiv) was added and the mixture was allowed to warm up to rt with stirring. The reaction was continued for another 2 hours at rt. Subsequently, the mixture was quenched with saturated aqueous ammonium chloride (NH_4Cl, 10 mL). The aqueous layer was

extracted with ethyl acetate (3 x 30 mL), and the combined organic layers were dried with $MgSO_4$. The mixture was filtered, and the solvents were evaporated under reduced pressure. The crude products purified by column chromatography on silica gel using *n*-hexane : ethyl acetate, 10:1 as eluent gave 0.582 g of white solid product (80% yield).

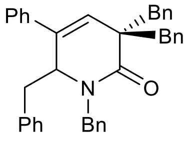

2m

1,3,3,6-Tetrabenzyl-5-phenyl-3,6-dihydropyridin-2(1*H*)-one (**2m**). M.p. 178-180ºC. ^{1}H NMR ($CDCl_3$, 400 MHz): δ 7.36 (t, 2H, *J* = 7.5 Hz, C_6H_5), 7.31 – 6.96 (m, 19H, $5C_6H_5$), 6.82 – 6.63 (m, 2H, C_6H_5), 6.37 – 6.18 (m, 2H, C_6H_5), 5.71 (d, 1H, *J* = 15.4 Hz, NCH<u>H</u>), 5.67 (s, 1H, =CH-4), 4.17 (dd, 1H, *J* = 5.0, 4.4 Hz, CH-6), 3.65 (d, 1H, *J* = 15,4 Hz, NC<u>H</u>H), 3.40 (d, 1H, *J* = 12.8 Hz, 3-CH<u>H</u>), 2.91 (d, 1H, *J* = 12.7 Hz, 3-CH<u>H</u>), 2.63 (d, 1H, *J* = 12.8 Hz, 3-C<u>H</u>H), 2.44 (dd, 1H, *J* = 14.4, 4.4 Hz, 6-CH<u>H</u>), 2.33 (d, 1H, *J* = 12.7 Hz, 3-C<u>H</u>H), 1.82 (dd, 1H, *J* = 14.4, 5.0 Hz, 6-C<u>H</u>H). ^{13}C{H} NMR ($CDCl_3$, 101 MHz): δ 171.6 (C=O), 139.0 (=C-5), 137.6, 137.4, 137.3, 136.5, 136.0, (Ar), 131.2 (2C), 130.9 (2C), 129.6 (2C), (ArH), 129.0 (=CH-4), 128.7 (2C), 128.4 (2C), 128.3 (2C), 128.1 (2C), 127.9 (2C), 127.7, 127.4 (2C), 126.9, 126.7, 126.6, 126.4 (2C), 126.2, (ArH), 58.3 (CH-6), 50.2 (C-3), 46.7 (3-CH_2), 46.4 (NCH_2), 46.0 (3-CH_2), 38.5 (6-CH_2). GC-MS (EI, 70eV): m/z = 533 (<1) [M^+], 442 (51), 260 (29), 207 (29), 91 (100). HRMS (ESI-TOF) m/z: $[M + H]^+$ Calcd for $C_{39}H_{36}NO$ 534.2797; Found 534.2802.

3. Procedure for the synthesis of **4a**, **5a**, **5b**, **6b** as a part of the preliminary studies

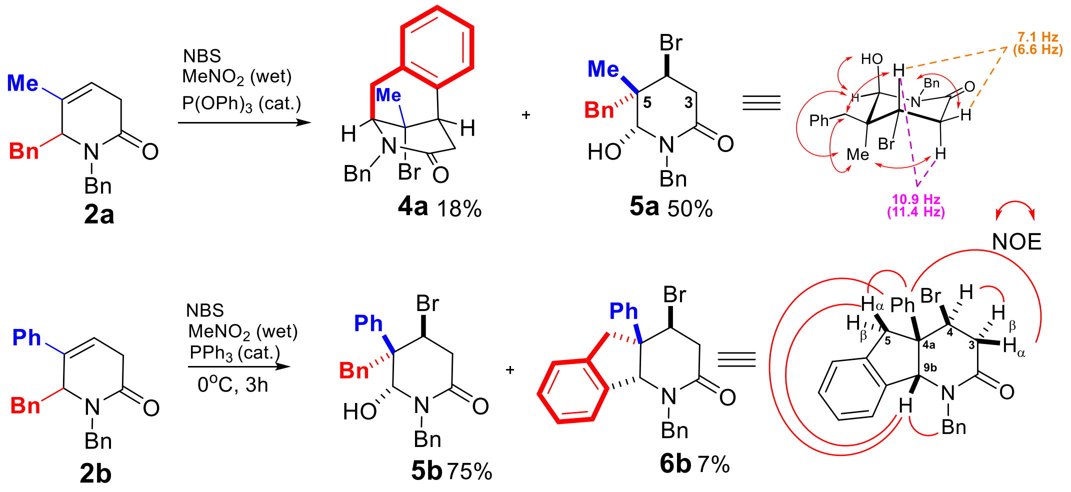

Scheme S9

To a stirred solution of C5-substituted *N*,6-dibenzyl-3,6-dihydropyridin-2(1*H*)-ones (1.0 mmol) [**2a** (C5-Me) or **2b** (C5-Ph)] in wet $MeNO_2$ (25 mL), placed in 50 mL flask, triphenyl phosphite for **2a** (0.175 mmol, 0.0543 g) or triphenylphosphine for **2b** (0.3 mmol, 0.0787 g) and *N*-bromosuccinimide (1.5 mmol, 0.2670 g) were added. The resulting orange-yellow solution was stirred in the dark for 10 h at room temperature for **2a** or 3h at 0ºC for **2b**. After this time 2% solution of Na_2SO_3 (10 mL) and then

saturated aqueous solution of $NaHCO_3$ (10 mL) were added and the mixture was stirred for additional 10 min. The aqueous layer was extracted with ethyl acetate (3 × 50 mL) and the combined organic layers were dried over Na_2SO_4. The mixture was filtered and the solvents were evaporated under reduced pressure. The crude product was purified by column chromatography on silica gel using a mixture of *n*-hexane, ethyl acetate and chloroform in volume ratio v/v=7:1:2 at the first stage, allowing isolation of 0.0664 g of product **4a** (18% yield) and 0.031 g of **6b** (7% yield). In the second purification step solvent mixture was replaced with *n*-hexane and ethyl acetate (in volume ratio 1:2), allowing to isolate compounds: **5a** (0.1935 g, 50% yield) and **5b** (0.3378 g, 75% yield).

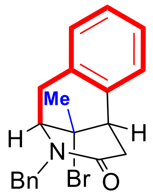

4a

(2*SR*,6*RS*,11*RS*)-3-Benzyl-11-bromo-11-methyl-2,3,5,6-tetrahydro-2,6-methano-benzo[*d*]azocin-4(1*H*)-one (**4a**): Semi-solid. ^{1}H NMR ($CDCl_3$, 400 MHz): δ 7.37-7.15 (m, 7 H, ArH-8, ArH-9, C_6H_5), 7.11-7.07 (m, 1 H, ArH-7), 7.04-7.00 (m, 1 H, ArH-10), 5.37 (d, 1 H, *J* = 14.9 Hz, NCHH), 4.01 (d, 1 H, *J* = 14.9 Hz, NCHH), 3.80 (dt, 1 H, *J* = 4.0, 2.0 Hz, H-2), 3.41 (dd, 1 H, *J* = 17.7, 6.2 Hz, CHH_β-5), 3.33 (d, 1 H, *J* = 6.2 Hz, CH-6), 3.08 (dd, 1 H, *J* = 17.7, 4.0 Hz, CHH_β-1), 3.02 (dd, 1 H, *J* = 17.7, 2.0 Hz, CHH_α-1), 2.60 (d, 1H, *J* = 17.7 Hz, CHH_α-5), 1.87 (s, 3 H, 11-CH_3). ^{13}C{H} NMR ($CDCl_3$, 101 MHz): δ 167.8 (C=O), 138.2, 136.2, 129.4, 129.2, 129.1, 128.8 (2C), 128.4 (2C), 127.61, 127.56, 127.5, (Ar), 64.8 (C-11), 62.2 (CH-2), 48.6 (NCH_2), 46.3 (CH-6), 41.6 (CH_2-5), 32.9 (CH_2-1), 29.9 (11-CH_3). GC-MS (EI, 70eV): m/z = 369 (27), [M^+], 290 (11), 155 (16), 148 (25), 143 (55), 128 (28), 115 (20), 106 (16), 91 (100), 65 (10); IR (KBr pellet): ν = 3032 (w), 2980 (w), 1636 (s), 1494 (m), 1456 (m), 1414 (w), 1304 (m), 1148 (w), 1108 (w), 1072 (m), 954 (w), 840 (w), 804 (w), 742 (w) 704 (m) cm^{-1}. HRMS (ESI-TOF) m/z: $[M + H]^+$ Calcd for $C_{20}H_{21}BrNO$ 370.0807; Found 370.0798.

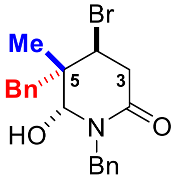

5a

(4*SR*,5*SR*,6*RS*)-1,5-Dibenzyl-4-bromo-6-hydroxy-5-methylpiperidin-2-one (**5a**): White solid. M.p. 173-175°C (*n*-hexane : ethyl acetate). ^{1}H NMR ($CDCl_3$, 400 MHz): δ = 7.30 – 7.12 (m, 10 H, 2 x C_6H_5), 4.77 (dd, 1 H, *J* = 10.9, 7.1 Hz, CH_{ax}-4), 4.58 (d, *J* = 14.6 Hz, 1 H, NCHH), 4.49 (d, *J* = 14.6 Hz, 1 H, NCHH), 4.37 (d, 1 H, *J* = 4.8 Hz, CH_{eq}-6), 3.55 – 3.50 (br s, 1 H, 6-OH_{ax}), 3.21 (dd, 1 H, *J* = 18.5, 7.1 Hz, CHH_{eq}-3), 3.03 (dd, 1 H, *J* = 18.5, 10.9 Hz, CHH_{ax}-3), 2.95 (d, 1 H, *J* = 13.3 Hz, 5-CHH), 2.88 (d, 1 H, *J* = 13.3 Hz, 5-CHH), 0.84 (s, 3 H, 5-CH_3). ^{13}C{H} NMR ($CDCl_3$, 101 MHz): δ 168.4 (C=O), 136.6, 136.5, 130.7 (2C), 128.9 (3C), 128.3 (2C), 128.2, 128.0, 126.7, (2 x C_6H_5), 83.4 (CH-6), 51.9 (CH-4), 49.0 (NCH_2), 43.4 (C-5), 42.5 (5-CH_2), 41.0 (CH_2-3), 15.4 (5-CH_3). GC-MS (EI, 70eV): m/z = 389 (7) [M+2], 387 (7), [M^+], 308 (18), 174 (20), 145 (20), 136 (70), 132 (13), 129 (18), 106 (22), 91 (100), 65 (12). IR (KBr pellet): ν = 3350 br (m), 3032 (w), 2980 (w), 1636 (s), 1494 (m), 1456 (m), 1414 (w), 1304 (m),

1148 (w), 1108 (w), 1072 (m), 954 (w), 840 (w), 804 (w), 742 (w), 704 (m) cm^{-1}. HRMS (ESI-TOF) m/z: $[M + H]^+$ Calcd for $C_{20}H_{23}BrNO_2$ 388.0912; Found 388.0910.

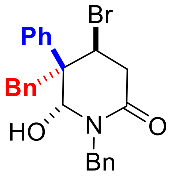

5b

(4*SR*,5*RS*,6*RS*)-1,5-Dibenzyl-4-bromo-6-hydroxy-5-phenylpiperidin-2-one (**5b**): White solid. M.p. 175-177ºC (petroleum ether : *n*-hexane). ^{1}H NMR ($CDCl_3$, 400 MHz): δ 7.37 – 7.20 (m, 8H, ArH), 7.20 – 7.07 (m, 3H, ArH), 7.03 (t, 2H, *J* = 7.5 Hz, ArH), 6.65 – 6.57 (m, 2H, ArH), 5.19 (dd, 1H, *J* = 12.7, 5.1 Hz, CH-4), 4.93 (d, 1H, *J* = 4.4 Hz, CH-6), 4.72 (d, 1H, *J* = 14.5 Hz, NCHH), 4.55 (d, 1H, *J* = 14.5 Hz, NCHH), 3.50 (d, 1H, *J* = 13.8 Hz, 5-CHH), 3.16 (d, 1H, *J* = 13.8 Hz, 5-CHH), 3.04 (d, 1H, *J* = 4.4 Hz, 6-OH), 3.04 (dd, 1H, *J* = 17.6, 5.1 Hz, $CHH_β$-3), 2.59 (dd, 1H, *J* = 17.6, 12.7 Hz, $CHH_α$-3). ^{13}C{H} NMR ($CDCl_3$, 101 MHz): δ 168.1 (C=O), 137.0, 136.4, 136.3, (Ar), 130.0 (2C), 129.1 (2C), 129.0 (2C), 128.2, 128.1 (4C), 128.0 (2C), 127.6, 126.7, (ArH), 82.2 (CH-6), 50.9 (C-5), 50.0 (CH-4), 49.0 (NCH_2), 44.4 (5-CH_2), 41.2 (CH_2-3). GC-MS (EI, 70eV): m/z = 451 (1) [M+2], 449 (1), [M^+], 370 (14), 261 (9), 234 (17), 156 (24), 136 (21), 129 (19), 115 (13), 106 (10), 91 (100), 65 (9). IR (KBr pellet): ν = 3400 br (m), 3060 (w), 3028 (w), 2944 (w), 1630 (s), 1496 (w), 1478 (w), 1452 (w), 1412 (w), 1306 (w), 1256 (w), 1142 (w), 1064 (m), 932 (w), 834 (w), 748 (w), 700 (m) cm^{-1}. HRMS (ESI-TOF) m/z: $[M + H]^+$ Calcd for $C_{25}H_{25}BrNO_2$ 450.1069; Found 450.1054.

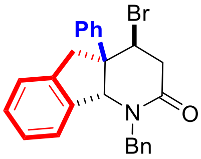

6b

(4*SR*,4a*RS*,9b*RS*)-1-Benzyl-4-bromo-4a-phenyl-1,3,4,4a,5,9b-hexahydro-2*H*-indeno[1,2-*b*]pyridine-2-one (**6b**): Colorless solid. M.p. 176-178ºC (petroleum ether : *n*-hexane). ^{1}H NMR ($CDCl_3$, 400 MHz): δ 7.59 – 7.54 (m, 2 H, ArH), 7.48 (d, 1H, *J* = 7.1 Hz, ArH), 7.43 – 7.27 (m, 6H, ArH), 7.17 (t, 1H, *J* = 7.1 Hz, ArH), 7.02 (t, 2H, *J* = 8.1 Hz, ArH), 6.98 – 6.93 (m, 2H, ArH), 6.00 (d, 1H, *J* = 13.8 Hz, NCHH), 5.30 (s, 1H, CH-9b), 4.45 (dd, 1H, *J* = 12.7, 5.1 Hz, CH-4), 4.29 (d, 1H, *J* = 13.8 Hz, NCHH), 3.74 (d, 1H, *J* = 15.6 Hz, $CHH_β$-5), 3.22 (d, 1H, *J* = 15.6 Hz, $CHH_α$-5), 2.92 (dd, 1H, *J* = 17.6, 5.1 Hz, $CHH_β$-3), 2.62 (dd, 1H, *J* = 17.6, 12.7 Hz, $CHH_α$-3). ^{13}C{H} NMR ($CDCl_3$, 101 MHz): δ 167.4 (C=O), 142.0, 139.5, 138.6, 135.8, 130.3 (2C), 128.9 (2C), 128.6 (2C), 128.5, 128.4 (2C), 127.6, 127.42, 127.37, 126.0, 123.5, (Ar), 67.0 (CH-9b), 55.8 (C-4a), 50.8 (NCH_2), 50.0 (CH-4), 42.9 (CH_2-3), 39.7 (CH_2-5). GC-MS (EI, 70eV): m/z = 433 (16) [M+2], 431 (16), [M^+], 352 (23), 351 (30), 260 (13), 246 (21), 217 (25), 204 (20), 192 (15), 160 (16), 115 (16), 106 (42), 91 (100), 65 (10). HRMS (ESI-TOF) m/z: $[M + H]^+$ Calcd for $C_{25}H_{23}BrNO$ 432.0963; Found 432.0966.

4. Procedure for the synthesis of **6a** and **6b** from 6-hydroksylactams **5a** and **5b** as a part of the preliminary studies

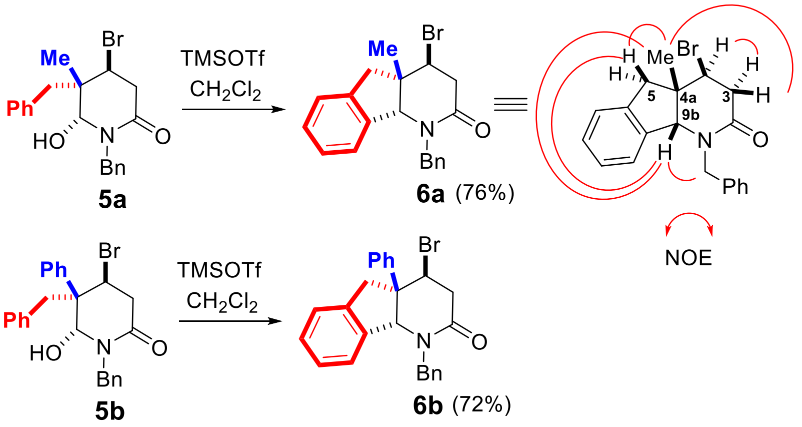

Scheme S10

To a stirred solution of lactam **5a** (0.54 mmol) or **5b** (0.33 mmol) in CH_2Cl_2 freshly distilled over CaH_2 (**5a**: 15 mL, **5b**: 9 mL), placed in a Schlenk flask equipped with a condenser (under argon), TMSOTf (**5a**: 1.08 mmol, **5b**: 0.668 mmol) was added. The resulting solution was stirred (**5a**: at 40ºC for 18 h, **5b**: at room temperature for 18 h). After this time aqueous, saturated solution of $NaHCO_3$ was added (7-10 mL) and the mixture was extracted with ethyl acetate (3 × 30-45 mL), and the combined organic layers were dried with $MgSO_4$. The mixture was filtered and the solvents were evaporated under reduced pressure. The crude product purified by column chromatography on silica gel using appropriates solvents.

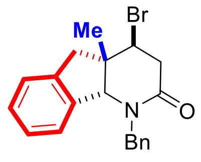

6a

(4*SR*,4a*SR*,9b*RS*)-1-Benzyl-4-bromo-4a-methyl-1,3,4,4a,5,9b-hexahydro-2*H*-indeno[1,2-*b*]pyridine-2-one (**6a**): Yield 76% (0.146 g). The crude product purified by column chromatography on silica gel using the mixture of *n*-hexane : AcOEt (6:1) gave white solid. M.p. 101–102ºC. ^{1}H NMR ($CDCl_3$, 400 MHz): δ 7.50 – 7.03 (m, 9H, C_6H_5, C_6H_4), 5.88 (d, 1H, *J* = 14.7 Hz, NCH<u>H</u>), 4.56 (d, 1H, *J* = 1.2 Hz, CH-9b), 4.24 (dd, 1H, *J* = 9.9, 7.5 Hz, CH-4), 4.09 (d, 1H, *J* = 14.7 Hz, NC<u>H</u>H), 3.21 (d, 1H, *J* = 15.8 Hz, CH$\underline{H_\beta}$-5), 3.09 – 3.00 (m, 2H, CH_2-3), 2.70 (dd, 1H, *J* = 15.8, 1.2 Hz, CH$\underline{H_\alpha}$ -5), 1.20 (s, 3H, 4a-CH_3). ^{13}C{H} NMR ($CDCl_3$, 101 MHz): δ 166.8 (C=O), 141.9, 139.5, 136.4, (Ar), 128.7 (2C), 128.34 (2C), 128.3, 127.9, 127.1, 126.1, 123.5, (ArH), 67.5 (CH-9b), 51.3 (CH-4), 49.5 (NCH_2), 49.1 (C-4a), 42.5 (CH_2-5), 39.7 (CH_2-3), 18.9 (4a-CH_3). GC-MS (EI, 70eV): m/z = 369 (19) [M^+], 290 (16), 289 (16), 185 (14), 169 (13), 157 (36), 143 (28), 141 (28), 129 (30), 128 (30), 115 (25), 106 (68), 91 (100), 65 (19). HRMS (ESI-TOF) m/z: $[M + H]^+$ Calcd for $C_{20}H_{21}BrNO$ 370.0807; Found 370.0815.

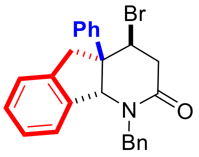

6b

(4*SR*,4a*RS*,9b*RS*)-1-Benzyl-4-bromo-4a-phenyl-1,3,4,4a,5,9b-hexahydro-2*H*-indeno[1,2-*b*]pyridine-2-one (**6b**): Yield 72% (0.144). The crude product was purified by column chromatography on silica gel using *n*-hexane : ethyl acetate, 8:1 as eluent.

5. Synthesis of bromoindenopyridin-2-ones **6** from lactams **2**

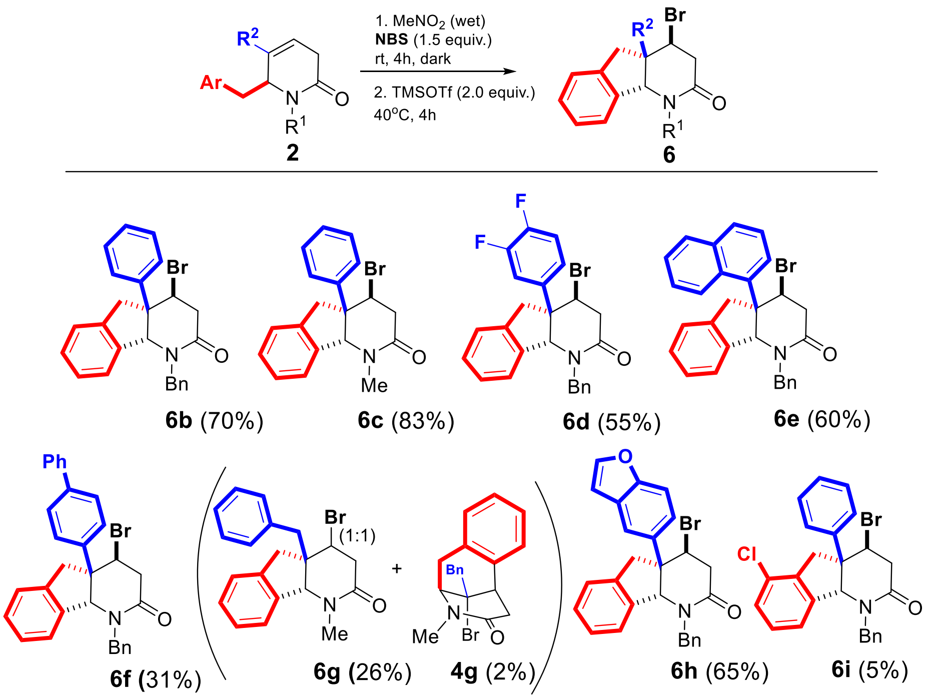

Scheme S11

Procedure for the synthesis of **6c** as representative example:

To a stirred solution of 3,6-dihydropyridin-2(1*H*)-one **2c** (0.276 g, 1.0 mmol) in wet $MeNO_2$ (20 mL) placed in 50 mL flask 1.5 equiv of *N*-bromosuccinimide (1.49 mmol, 0.265 g) was added. The flask contents were stirred for 4 h at room temperature in the dark. After this time 2% solution of Na_2SO_3 (4 mL) and then saturated aqueous solution of $NaHCO_3$ (4 mL) were added and the mixture was stirred for additional 10 min. The aqueous layer was extracted with ethyl acetate (3 × 30 mL) and the combined organic layers were dried over Na_2SO_4. The mixture was filtered and the solvents were evaporated under reduced pressure. The crude reaction mixture was dissolved in a minimum amount of $CHCl_3$ and the solution was transferred into 100 mL Schlenk flask, then the solvent was evaporated and the residue was dried under vacuum (ca. 0.1 mmHg) with heating (ca 120°C) three times for 4 minutes using argon for pressure equalization. Subsequently, 40 mL of CH_2Cl_2 (freshly distilled over CaH_2) was added to the flask under argon followed by TMSOTf (0.442 g, 2.0 mmol, 2 eq.). The flask was equipped with

condenser crowned with balloon with argon. The resulting solution was stirred at 40°C until the substrate was consumed completely (TLC or ^{1}H NMR control) After this time, the mixture was quenched with saturated aqueous $NaHCO_3$ (15 mL). The mixture was extracted with ethyl acetate (3 × 40 mL), and the combined organic layers were dried with $MgSO_4$. The mixture was filtered and the solvents were evaporated under reduced pressure. The crude product purified by column chromatography (SiO_2, *n*-hexane : ethyl acetate, 3:1) gave 0.294 g of white solid (83% yield).

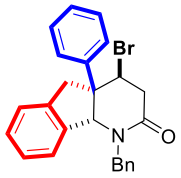

6b

(4*SR*,4a*RS*,9b*RS*)-1-Benzyl-4-bromo-4a-phenyl-1,3,4,4a,5,9b-hexahydro-2*H*-indeno[1,2-*b*]pyridine-2-one (**6b**): [Prepared from 1,6-dibenzyl-5-phenyl-3,6-dihydropyridin-2(1*H*)-one (**2b)** (0.171 g). The crude product purified by column chromatography on silica gel using appropriate solvents (*n*-hexane : ethyl acetate, 1:3), gave 0.146 g (70% yield).

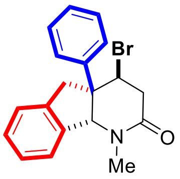

6c

(4*SR*,4a*RS*,9b*RS*)-4-Bromo-1-methyl-4a-phenyl-1,3,4,4a,5,9b-hexahydro-2*H*-indeno[1,2-*b*]pyridine-2-one (**6c**): Yield 83% (0.294 g). [Prepared from 6-benzyl-1-methyl-5-phenyl-3,6-dihydropyridin-2(1*H*)-one (**2c)** (0.276 g). Reaction time 4 h.] The crude product purified by column chromatography (SiO_2, *n*-hexane : ethyl acetate, 3:1) gave white solid. M.p. 171-174°C (petroleum ether : *n*-hexane). ^{1}H NMR ($CDCl_3$, 400 MHz): δ 7.43 – 7.24 (m, 9 H, ArH), 5.17 (s, 1 H, CH-9b), 4.45 (dd, 1H, *J* = 12.8, 5.3 Hz, CH-4), 3.80 (d, 1H, *J* = 15.6 Hz, CH<u>H</u>$_β$-5), 3.49 (s, 3H, NCH_3), 3.27 (d, 1H, *J* = 15.7 Hz, CH<u>H</u>$_α$-5), 2.89 (dd, 1H, *J* = 17.6, 5.3 Hz, CH<u>H</u>$_β$-3), 2.62 (dd, 1H, *J* = 17.6, 12.8 Hz, CH<u>H</u>$_α$-3). $^{13}C\{H\}$ NMR ($CDCl_3$, 101 MHz): δ 167.7 (C=O), 142.0, 139.9, 138.3, 128.6, 128.5 (2C), 128.2 (2C), 127.9, 127.4, 125.9, 123.5, (Ar), 71.2 (CH-9b), 56.0 (C-4a), 50.1 (CH-4), 42.9 (CH_2-5), 39.7 (CH_2-3), 36.5 (NCH_3). GC-MS (EI, 70eV): m/z = 355 (12), [M^+], 276 (100), 258 (27), 248 (24), 220 (14), 218 (16), 217 (56), 215 (17), 204 (19), 202 (22), 198 (16), 191 (12), 131 (17), 115 (20), 91 (40). HRMS (ESI-TOF) m/z: $[M + H]^+$Calcd for $C_{19}H_{19}BrNO$ 356.0650; Found 356.0656.

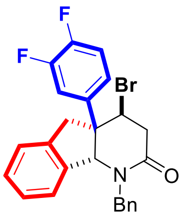

6d

(4*SR*,4a*RS*,9b*RS*)-1-Benzyl-4-bromo-4a-(3,4-difluorophenyl)-1,3,4,4a,5,9b-hexahydro-2*H*-indeno[1,2-*b*]pyridine-2-one (**6d**): Yield 55% (0.229 g). [Prepared from **2d** (0.347 g). Reaction time 72h. After 24 and 48 hours additional portions of TMSOTf and 10 ml of CH_2Cl_2 were added]. The crude product purified by column chromatography (SiO_2, *n*-hexane : ethyl acetate, 8:1) gave white solid. M.p. 189-190°C (petroleum ether : ethyl acetate). ^{1}H NMR ($CDCl_3$, 400 MHz): δ 7.58 – 7.51 (m, 2H, ArH), 7.51 – 7.47 (m, 1H, ArH), 7.43 – 7.38 (m, 3H, ArH), 7.36 – 7.28 (m, 3H, ArH), 6.87 (ddd, 1H, *J* = 12.0, 7.6, 2.4 Hz, ArH), 6.73 (dt, 1H, *J* = 10.0, 8.4 Hz, ArH), 6.57 (dddd, 1H, *J* = 8.4, 4.0,

2.4, 1.4 Hz, ArH), 6.04 (d, 1H, J = 13.7 Hz, NCH<u>H</u>), 5.24 (s, 1H, CH-9b), 4.41 (dd, 1H, J = 12.9, 5.4 Hz, CH-4), 4.23 (d, 1H, J = 13.7 Hz, NC<u>H</u>H), 3.68 (d, 1H, J = 15.5 Hz, CH$\underline{H_\beta}$-5), 3.16 (d, 1H, J = 15.5 Hz, CH$\underline{H_\alpha}$-5), 2.94 (dd, 1H, J = 17.7, 5.4 Hz, CH$\underline{H_\beta}$-3), 2.56 (dd, 1H, J = 17.7, 12.9 Hz, CH$\underline{H_\alpha}$-3). ^{13}C{H} NMR ($CDCl_3$, 101 MHz): δ 167.0 (C=O), 149.6 (J_{CF} = 249.9, 15.3 Hz, C-4'), 149.4 (J_{CF} = 248.0, 15.5 Hz, C-3'), 141.6, 138.0, (Ar), 136.4 (J_{CF} = 4.7 Hz, C-1'), 135.5 (Ar), 130.2 (2C), 129.1 (2C), 128.83, 128.77, 127.6, 126.1, ArH, 124.4 (J_{CF} = 6.0, 3.7 Hz, CH-6'), 123.5 (ArH), 118.1 (J_{CF} = 18.4 Hz, CH-2'), 116.2 (J_{CF} = 16.9 Hz, C-5'), 66.7 (CH-9b), 55.2 (C-4a), 50.7 (NCH_2), 49.4 (CH-4), 42.9 (CH_2-5), 39.7 (CH_2-3). ^{19}F NMR ($CDCl_3$, 377 MHz): δ -137.09 (d, J = 21.6 Hz), -138.77 (d, J = 21.6 Hz). GC-MS (EI, 70eV): m/z = 467 (9), [M^+], 388 (10), 253 (10), 160 (22), 127 (23), 106 (49), 91 (100). HRMS (ESI-TOF) m/z: [M + Na]$^+$ Calcd for $C_{25}H_{20}BrF_2NNaO$ 490.0594; Found 490.0592.

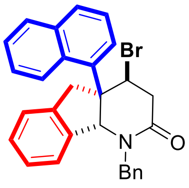

6e

(4*RS*,4a*SR*,9b*SR*)-1-Benzyl-4-bromo-4a-(naphthalen-1-yl)-1,3,4,4a,5,9b-hexahydro-2*H*-indeno[1,2-*b*]pyridin-2-one (**6e**): Yield 60% (0.345 g). [Prepared from **2e** (0.479 g). Reaction time 3 h.] The crude product purified by column chromatography (SiO_2, *n*-hexane : ethyl acetate, 10:1) gave white semisolid. ^{1}H NMR ($CDCl_3$, 400 MHz): δ 7.99 (dd, 1H, J = 7.1, 0.8 Hz, ArH), 7.75 (dd, 1H, J = 8.2, 0.7 Hz, ArH), 7.70 (d, 1H, J = 8.2 Hz, ArH), 7.62 (dd, 1H, J = 8.2, 7.1 Hz, ArH), 7.43 – 7.31 (m, 4H, ArH), 7.24 – 7.16 (m, 2H, ArH), 7.12 – 7.03 (m, 2H, ArH), 7.02 – 6.94 (m, 2H, ArH), 6.70 – 6.61 (m, 2H, ArH), 5.26 (d, 1H, J = 14.7 Hz, NC<u>H</u>H), 5.22 (t, 1H, J = 0.9 Hz, CH-9b), 4.59 (dd, J = 1H, 13.5, 2.9 Hz, CH-4), 4.30 (d, 1H, J = 14.7 Hz, NC<u>H</u>H), 3.23 (d, 1H, J = 13.8 Hz, CH<u>H</u>-5), 3.16 (d, 1H, J = 13.8 Hz, C<u>H</u>H-5), 2.82 (dd, 1H, J = 15.8, 2.9 Hz, CH$\underline{H_\beta}$-3), 2.37 (dd, 1H, J = 15.8, 13.5 Hz, CH$\underline{H_\alpha}$-3). ^{13}C{H} NMR ($CDCl_3$, 101 MHz): δ 170.3 (C=O), 142.5, 141.2, 138.2, 136.9, 135.6, 130.9, (Ar), 130.2 (2C), 129.1 (2C), 128.6 (2C), 128.3 (2C), 127.92, 127.85, 127.7, 126.9, 125.1, 124.7, 123.8, 119.5, ArH, 65.5 (CH-9b), 57.0 (C-4a), 52.2 (CH-4), 48.9 (NCH_2), 44.6 (CH_2-5), 41.1 (CH_2-3). GC-MS (EI, 70eV): *m/z* = 481 (2) [M^+], 311 (18), 310 (52), 220 (8), 179 (12), 178 (24), 165 (14), 132 (25), 106 (9), 91 (100), 65 (9). HRMS (ESI-TOF) m/z: [M + Na]$^+$Calcd for $C_{29}H_{24}BrNNaO$ 504.0939; Found 504.0939.

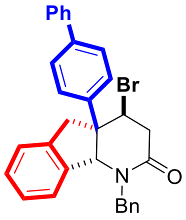

6f

(4*SR*,4a*RS*,9b*RS*)-4a-([1,1'-Biphenyl]-4-yl)-1-benzyl-4-bromo-1,3,4,4a,5,9b-hexahydro-2*H*-inde-no[1,2-*b*]pyridin-2-one (**6f**): Yield 31% (0.124 g). [Prepared from **2f** (0.338 g). Reaction time 2 h.] The crude product purified by column chromatography (SiO_2, *n*-hexane : ethyl acetate, 10:1) gave white solid. M.p. 176-177ºC. ^{1}H NMR ($CDCl_3$, 400 MHz): δ 7.63 – 7.17 (m, 16H, ArH), 7.08 – 6.96 (m, 2H, ArH), 6.03 (d, 1H, J = 13.7 Hz, NCH<u>H</u>), 5.33 (s, 1H, CH-9b), 4.48 (dd, 1H, J = 12.8, 5.3 Hz, CH-4), 4.30 (d, 1H, J = 13.7 Hz, NC<u>H</u>H), 3.77 (d, 1H, J = 15.7 Hz, CH$\underline{H_\beta}$ -5), 3.25 (d, 1H, J = 15.7 Hz, CH$\underline{H_\alpha}$ -5), 2.95 (dd, 1H, J = 17.6, 5.3 Hz, CH$\underline{H_\beta}$-3), 2.68 (dd, 1H, J = 17.6, 12.8 Hz, CH$\underline{H_\alpha}$ -3). ^{13}C{H}

NMR ($CDCl_3$, 101 MHz): δ 167.4 (C=O), 142.0, 140.4, 140.2, 138.6, 138.5, 135.8, (Ar), 130.3 (2C), 129.0 (2C), 128.9 (2C), 128.8 (2C), 128.7, 128.6, 127.4 (2C), 127.1 (2C), 126.3 (2C), 126.1, 123.5, (ArH), 67.1 (CH-9b), 55.7 (C-4a), 50.8 (NCH_2), 50.0 (CH-4), 42.9 (CH_2-5), 39.9 (CH_2-3). GC-MS (EI, 70eV): m/z = 507 (0) [M^+], 428 (32), 427 (100), 410 (19), 336 (34), 322 (48), 320 (20), 293 (27), 215 (12), 207 (29), 191 (13), 165 (15), 115 (13), 91 (84), 65 (11). HRMS (ESI-TOF) m/z: $[M + Na]^+$ Calcd for $C_{31}H_{26}BrNNaO$ 530.1095; Found 530.1091.

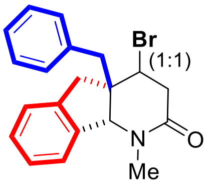

6g

(4*SR*,4a*SR*,9b*RS*)-4a-Benzyl-4-bromo-1-methyl-1,3,4,4a,5,9b-hexahydro-2*H*-indeno[1,2-b]pyridine-2-one and (4*RS*,4a*SR*,9b*RS*)-4a-benzyl-4-bromo-1-methyl-1,3,4,4a,5,9b-hexahydro-2*H*-indeno[1,2-*b*]pyridine-2-one (~1.0 : 0.9 mixture of diastereomers) (**6g**). Yield 26% (0.117 g). [Prepared from **2g** (0.350 g). Reaction time 24h.] The crude product purified by column chromatography (SiO_2, *n*-hexane : ethyl acetate, 10:1) gave white semisolid. ^{1}H NMR ($CDCl_3$, 400 MHz) δ 8.58 – 6.14 (m, 18H, ArH), 4.60 (s, 1H, CH-9b), 4.51 (s, 1H, CH-9b), 4.43 (dd, 1H, *J* = 6.5, 1.8 Hz, CH-4), 4.40 (dd, 1H, *J* = 6.4, 2.6 Hz, CH-4), 3.60 (d, 1H, *J* = 16.7 Hz, 5-CHH), 3.32 (d, 1H, *J* = 13.9 Hz, 5-CHH), 3.28 (s, 3H, NCH_3), 3.23 (d, 1H, *J* = 13.9 Hz, 5-CHH), 3.20 – 2.93 (m, 6H, 5-CH_2, CH_2-3, CH_2-3), 2.89 (d, 1H, *J* = 13.9 Hz, 5-CHH), 2.85 (s, 3H, NCH_3), 2.77 (d, 1H, *J* = 16.7 Hz, 5-CHH), 2.56 (d, 1H, *J* = 13.9 Hz, 5-CHH). ^{13}C{H} NMR ($CDCl_3$, 101 MHz): δ 166.8, 166.0, (2 x C=O), 141.41, 141.37, 139.9, 138.9, 136.6, 135.9, (2 x C_6H_5, 2 x C_6H_4), 131.0 (2C), 130.4 (2C), 129.3, 128.7 (2C), 128.6 (2C), 128.4, 127.3, 127.02, 127.00, 126.8, 126.3, 126.2, 126.0, 123.7, (2 x C_6H_5, 2 x C_6H_4), 67.41, 66.96, (2 x CH-9), 53.3 (C-4a), 51.9 (CH-4), 51.3 (C-4a), 49.3 (CH-4), 42.8, 40.7, (2 x 5-CH_2), 39.5, 39.3, (2 x 5-CH_2), 37.3, 35.8, (2 x CH_2-3), 35.7, 32.6, (2 x NCH_3). GC-MS (EI, 70eV): m/z = 369 (0) [M^+], 198 (85), 197 (100), 169 (13), 128 (12), 115 (13), 91 (19). HRMS (ESI-TOF) m/z: $[M + H]^+$ Calcd for $C_{20}H_{21}BrNO$ 370.0807; Found 370.0798.

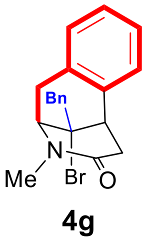

4g

(2*SR*,6*RS*,11*RS*)-11-Benzyl-11-bromo-3-methyl-2,3,5,6-tetrahydro-2,6-methanobenzo[*d*]azocin-4(1*H*)-one (**4g**): Yield ~2% (0.008g). [Prepared from **2g** (0.350 g)]. Reaction time 24h. The crude product purified by column chromatography (SiO_2, *n*-hexane : ethyl acetate, 10:1) gave white semisolid. ^{1}H NMR ($CDCl_3$, 400 MHz): δ 7.34 – 7.23 (m, 5H, ArH), 7.22 – 7.13 (m, 3H, ArH), 7.10 (dd, 1H, *J*=7.2, 1.8 Hz, ArH), 3.91 (dt, 1H, *J* = 4.4, 1.8 Hz, CH-2), 3.38 (dd, 1H, *J* = 17.8, 4.7 Hz, $CHH_β$-1), 3.31 (ddd, 1H, *J* = 6.5, 1.8, 1.2 Hz, CH-6), 3.24 – 3.21 (m, 2H, 11-CH_2), 3.19 (dd, 1H, *J* = 17.8, 1.8 Hz, $CHH_α$-1), 3.12 (dd, 1H, *J* = 18.0, 6.5 Hz, $CHH_β$-5), 3.05 (s, 3H, NCH_3), 2.45 (dd, 1H, *J* = 18.0, 1.2 Hz, $CHH_α$-5). ^{13}C{H} NMR ($CDCl_3$, 101 MHz): δ 168.1 (C=O), 138.4 (C-6a), 135.2, 131.2 (2C), (C_6H_5), 129.7, 129.2 (CH-7, CH-10), 129.2 (C-10a), 127.9 (3C, C_6H_5), 127.8, 127.4 (CH-8, CH-9), 70.0 (C-11), 65.4 (CH-

2), 44.4 (11-CH_2), 43.0 (CH-6), 41.0 (CH_2-5), 34.6 (NCH_3), 33.3 (CH_2-1). GC-MS (EI, 70eV): m/z = decomposition. HRMS (ESI-TOF) m/z: $[M + H]^+$Calcd for $C_{20}H_{21}BrNO$ 370.0807; Found 370.0816.

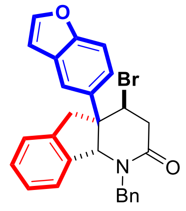

6h

(4*SR*,4a*RS*,9b*RS*)-4a-(Benzofuran-5-yl)-1-benzyl-4-bromo-1,3,4,4a,5,9b-hexahydro-2H-indeno[1,2-*b*]pyridine-2-one (**6h**): Yield 65% (0.207 g). [Prepared from **2h** (0.266 g). Reaction time 3 h.] The crude product purified by column chromatography (SiO_2, *n*-hexane : ethyl acetate, 6:1) gave brown semisolid. ^{1}H NMR ($CDCl_3$, 400 MHz): δ 7.72 – 7.57 (m, 2H, ArH), 7.56 – 7.41 (m, 5H, ArH), 7.38 – 7.29 (m, 3H, ArH), 7.22 (dd, 1H, *J* = 8.6, 1.0 Hz, ArH), 7.11 – 6.98 (m, 2H, ArH), 6.55 – 6.32 (m, 1H, ArH), 6.05 (d, 1H, *J* = 13.7 Hz, NCH<u>H</u>), 5.40 (s, 1H, CH-9b), 4.48 (dd, 1H, *J* = 12.8, 5.2 Hz, CH-4), 4.29 (d, 1H, *J* = 13.7 Hz, NC<u>H</u>H), 3.80 (d, 1H, *J* = 15.6 Hz, $CH\underline{H_\beta}$ -5), 3.27 (d, 1H, *J* = 15.6 Hz, $CH\underline{H_\alpha}$ -5), 2.91 (dd, 1H, *J* = 17.5, 5. Hz, $CH\underline{H_\beta}$ -3), 2.62 (dd, 1H, *J* = 17.5, 12.8 Hz, $CH\underline{H_\alpha}$ -3). ^{13}C{H} NMR ($CDCl_3$, 101 MHz): δ 167.5 (C=O), 153.9 (Ar), 145.2 (ArH), 142.2, 138.7, 136.0, 134.0, (Ar), 130.5 (2C), 129.0 (2C), 128.6, 128.5, 127.4, (ArH), 126.8 (Ar), 126.1, 125.4, 123.4, 120.8, (ArH), 110.2, 106.8 (ArH), 67.6 (CH-9b), 55.9 (C-4a), 50.9 (NCH_2), 50.4 (CH-4), 43.5 (CH_2-5), 39.9 (CH_2-3). GC-MS (EI, 70eV): m/z = 471 (14) [M^+], 392 (11), 281 (18), 244 (24), 207 (54), 202 (14), 160 (20), 131 (19), 106 (77), 91 (100), 65 (12). HRMS (ESI-TOF) m/z: $[M + H]^+$ Calcd for $C_{27}H_{23}BrNO_2$, 472.0912; Found 472.0907.

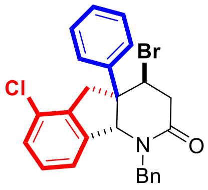

6i

(4*SR*,4a*RS*,9b*RS*)-1-Benzyl-4-bromo-6-chloro-4a-phenyl-1,3,4,4a,5,9b-hexahydro-2*H*-indeno[1,2-*b*]pyridine-2-one (**6i**): Yield ~6% (0.019 g). [Prepared from **2i** (0.279 g). Reaction time 96h. After every 24 hours the additional portion of TMSOTf and 10 ml of CH_2Cl_2 were added.] The crude product purified by column chromatography (SiO_2, *n*-hexane : ethyl acetate, 10:1 than 3 : 1) gave grey solid. M.p. 190-192ºC. ^{1}H NMR ($CDCl_3$, 400 MHz): δ 7.58 – 7.52 (m, 2H, ArH), 7.43 – 7.30 (m, 5H, ArH), 7.29 – 7.22 (m, 1H, ArH), 7.21 – 7.15 (m, 1H, ArH), 7.03 (dd, 2H, *J* = 8.6, 7.1 Hz, ArH), 6.99 – 6.92 (m, 2H, ArH), 5.97 (d, 1H, *J* = 13.8 Hz, NCH<u>H</u>), 5.32 (s, 1H, CH-9b), 4.42 (dd, 1H, *J* = 12.7, 5.3 Hz, CH-4), 4.25 (d, 1H, *J* = 13.8 Hz, NC<u>H</u>H), 3.88 (d, 1H, *J* = 16.3 Hz, $CH\underline{H_\beta}$ -5), 3.16 (d, 1H, *J* = 16.3 Hz, $CH\underline{H_\alpha}$ -5), 2.94 (dd, 1H, *J* = 17.6, 5.3 Hz, $CH\underline{H_\beta}$ -3), 2.62 (dd, 1H, *J* = 17.6, 12.7 Hz, $CH\underline{H_\alpha}$ -3). ^{13}C{H} NMR ($CDCl_3$, 101 MHz): δ 167.4 (C=O), 144.0, 139.0, 136.9, 135.6, 132.2, (Ar), 130.2 (2C), 129.0, 129.0 (2C), 128.8, 128.6, 128.4 (2C), 127.7 (2C), 127.6, 121.8, (ArH), 67.4 (CH-9b), 55.2 (C-4a), 50.7 (NCH_2), 49.7 (CH-4), 41.8 (CH_2-5), 39.8 (CH_2-3). GC-MS (EI, 70eV): m/z = 465 (<1) [M^+], 342 (21), 340 (21), 281 (13), 207 (32), 91 (100). HRMS (ESI-TOF) m/z: $[M + H]^+$ Calcd for $C_{25}H_{22}BrClNO$ 466.0573; Found 466.0580.

6. Synthesis of 1,4a,5,9b-tetrahydro-2*H*-indeno[1,2-*b*]pyridine-2-ones **7**

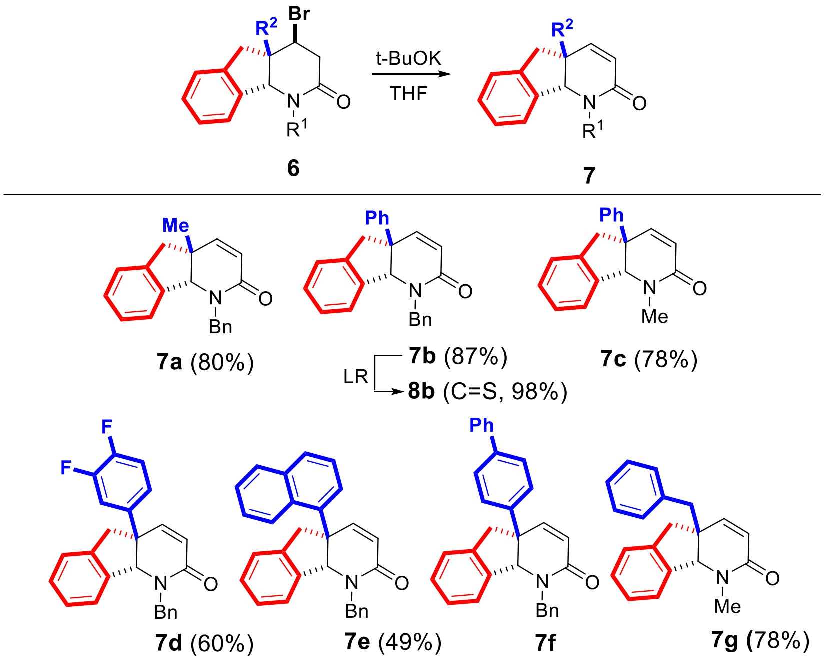

Scheme S12.

Procedure for the synthesis of **7c** as representative example:

A solution of bromoindenopyridin-2-one **6c** (0.304 g, 0.852 mmol) in dry THF (40 mL) in a Schlenk flask (100 mL) was stirred at room temperature under argon and *t*-BuOK (0.382 g, 3.41 mmol) was added. The resulting solution was stirred for 3 h (TLC control) and after this time the mixture was quenched with a saturated aqueous solution of NH_4Cl (20 mL). The aqueous layer was extracted with ethyl acetate (3 × 40 mL), and the combined organic layers were dried over $MgSO_4$. The mixture was filtered, and the solvents were evaporated under reduced pressure. The crude product purified by column chromatography (SiO_2, *n*-hexane : ethyl acetate, 3:1) gave 0.183 g of white solid (78% yield).

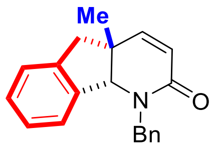

7a

(4a*RS*,9b*SR*)-1-Benzyl-4a-methyl-1,4a,5,9b-tetrahydro-2*H*-indeno[1,2-*b*]pyridine-2-one (**7a**): Yield 80% (0.0624 g). [Prepared from **6a** (0.100 g)]. The crude product purified by column chromatography (SiO_2, *n*-hexane : ethyl acetate, 3:1) gave white solid. M.p. 142-144ºC. ^{1}H NMR ($CDCl_3$, 400 MHz): δ 7.57 – 6.90 (m, 9H, C_6H_5, C_6H_4), 6.12 (dd, 1H, *J* = 9.9, 1.6 Hz, =CH-4), 5.83 (d, 1H, *J* = 9.9 Hz, =CH-3), 5.80 (d, 1H, *J* = 14.5 Hz, NCH<u>H</u>), 4.47 (d, 1H, *J* = 1.6 Hz, CH-9b), 4.17 (d, 1H, *J* = 14.5 Hz, NC<u>H</u>H), 2.90 (m, 2H, CH_2-5), 1.10 (s, 3H, CH_3). ^{13}C{H} NMR ($CDCl_3$, 101 MHz): δ 162.6 (C=O), 147.8 (=CH-4), 142.4, 139.4, 137.3, (Ar), 128.7 (2C), 128.6 (2C), 128.0, 127.7, 127.1, (ArH), 124.4 (=CH-3), 124.3, 123.2, (ArH), 67.7 (CH-9b), 50.2 (NCH_2), 46.5 (CH_2-5), 44.5 (C-4a), 23.1 (CH_3). GC-MS (EI, 70eV): m/z = 289 (100) [M+], 198 (22), 185 (28), 184 (40), 169 (64), 155 (24), 141 (34), 128 (30), 115 (26), 106 (16), 91 (72), 65 (15). HRMS (ESI-TOF) m/z: $[M + H]^+$ Calcd for $C_{20}H_{20}NO$ 290.1545; Found 290.1552.

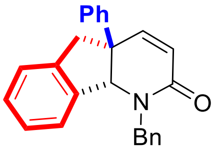

7b

(4a*RS*,9b*RS*)-1-Benzyl-4a-phenyl-1,4a,5,9b-tetrahydro-2*H*-indeno[1,2-*b*]pyridine-2-one (**7b**): Yield 87% (0.078 g). [Prepared from **6b** (0.111 g)]. The crude product purified by column chromatography (SiO_2, *n*-hexane : ethyl acetate, 8:1) gave white solid. M.p. 153-154ºC. ^{1}H NMR ($CDCl_3$, 400 MHz): δ 7.30 – 7.05 (m, 14 H, ArH), 6.37 (dd, 1H, *J* = 9.9, 1.7 Hz, =CH-4), 6.07 (d, 1H, *J* = 9.9 Hz, =CH-3), 5.68 (d, 1H, *J* = 14.5 Hz, NCHH), 5.06 (s, 1H, CH-9b), 4.24 (d, 1H, *J* = 14.5 Hz, NCHH), 3.56 (d, 1H, *J* = 15.8 Hz, CHH-5), 3.23 (d, 1H, *J* = 15.8 Hz, CHH-5). ^{13}C{H} NMR ($CDCl_3$, 100 MHz): δ 162.7 (C=O), 145.7 (=CH-4), 141.6, 141.4, 138.7, 136.4, (Ar), 128.7 (2C), 128.6 (2C), 128.5 (2C), 128.3, 127.5, 127.5, 127.2, 126.4 (2C), (ArH), 125.8 (=CH-3), 124.2, 123.2, (ArH), 69.0 (CH-9b), 53.5 (C-4a), 50.5 (NCH_2), 44.8 (CH_2-5). GC-MS (EI, 70eV): m/z = 351 (83) [M^+], 334 (20), 281 (15), 260 (36), 246 (67), 232 (11), 218 (27), 217 (49), 215 (41), 202 (32), 191 (16), 178 (9), 165 (9), 128 (25),106 (11), 91 (100). HRMS (ESI-TOF) m/z: $[M + H]^+$ Calcd for $C_{25}H_{22}NO$, 352.1701; Found 352.1705.

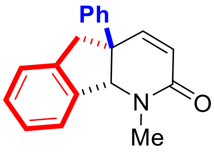

7c

(4a*RS*,9b*RS*)-1-Methyl-4a-phenyl-1,4a,5,9b-tetrahydro-2*H*-indeno[1,2-*b*]pyridine-2-one (**7c**): Yield 78% (0.183 g). [Prepared from **6c** (0.304 g)]. The crude product purified by column chromatography (SiO_2, *n*-hexane : ethyl acetate, 3:1) gave white solid. M.p. 188-190ºC. ^{1}H NMR ($CDCl_3$, 400 MHz): δ 7.45 – 7.21 (m, 9H, ArH), 6.31 (dd, 1H, *J* = 9.8, 1.8 Hz, =CH-4), 5.87 (d, 1H, *J* = 9.8 Hz, =CH-3), 5.07 (d, 1H, *J* = 1.8 Hz, CH-9b), 3.60 (d, 1H, *J* = 15.5 Hz, CHH-5), 3.42 (s, 3H, NCH_3), 3.34 (d, 1H *J* = 15.5 Hz, CHH-5). ^{13}C{H} NMR ($CDCl_3$, 101 MHz): δ 162.9 (C=O), 146.1 (=CH-4), 142.6, 142.0, 138.5, (Ar), 129.1 (2C), 128.4, 127.54, 127.50, 126.1 (2C), (ArH), 124.6 (=CH-3), 124.3, 123.3, (ArH), 71.7 (CH-9b), 53.3 (C -4a), 44.2 (CH_2-5), 36.2 (NCH_3). GC-MS (EI, 70eV): m/z = 275 (100) [M+], 258 (95), 244 (21), 220 (19), 217 (35), 215 (45), 202 (26), 144 (17), 131 (68), 115 (25), 89 (13), 77 (12). HRMS (ESI-TOF) m/z: $[M + H]^+$ Calcd for $C_{19}H_{18}NO$, 276.1388; Found: 276.1393.

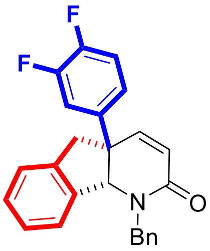

7d

(4a*RS*,9b*RS*)-1-Benzyl-4a-(3,4-difluorophenyl)-1,4a,5,9b-tetrahydro-2*H*-indeno[1,2-*b*]pyridine-2-one (**7d**): Yield 60% (0.025 g). [Prepared from **6d** (0.051 g)]. The crude product purified by column chromatography (SiO_2, *n*-hexane : ethyl acetate, 5:1) gave pale yellow solid. M.p. 137-139ºC (petroleum ether : ethyl acetate). ^{1}H NMR ($CDCl_3$, 400 MHz,) δ 7.31 – 7.17 (m, 5H, ArH), 7.14 (t, 2H, *J*=7.4 Hz, ArH), 7.07 (d, 2H, *J*=7.3 Hz, ArH), 6.95 – 6.84 (m, 2H, ArH), 6.84 – 6.75 (m, 1H, ArH), 6.28 (dd, 1H, *J* = 9.9, 1.8 Hz, =CH-4), 6.08 (d, 1H, *J* = 9.9 Hz, =CH-3), 5.78 (d, 1H, *J* = 14.3 Hz, NCHH), 4.94 (d, 1H, *J* = 1.8 Hz, CH-9b), 4.08 (d, 1H, *J* = 14.3 Hz, NCHH), 3.47 (d, 1H, *J* = 15.7 Hz, CHH-5), 3.18 (d, 1H, *J* = 15.7 Hz, CHH-5). ^{13}C{H} NMR ($CDCl_3$, 101 MHz): δ 162.2

(C=O), 149.9 (J_{CF} = 249.4, 12.8 Hz, C-4'), 149.4 (J_{CF} = 247.9, 12.7 Hz, C-3'), 144.4 (=CH-4), 141.2 (Ar), 138.3 (J_{CF} = 4.4 Hz, C-1'), 138.2, 136.3, (Ar), 128.8 (2C), 128.5 br (3C), 127.80, 127.77, ArH, 126.7 (=CH-3), 124.3, 123.1, (ArH), 122.6 (J_{CF} = 6.1, 3.5 Hz, CH-6'), 117.1 (J_{CF} = 17.0 Hz, CH-2'), 115.7 (J_{CF} = 18.2 Hz, CH-5'), 68.8 (CH-9b), 52.8 (C-4a), 50.2 (NCH_2), 44.7 (CH_2-5). ^{19}F NMR (377 MHz, $CDCl_3$) δ -136.20 (d, J = 21.4 Hz), -139.54 (d, J = 21.4 Hz). GC-MS (EI, 70eV): m/z = 387 (44) [M$^+$], 296 (21), 282 (47), 281 (12), 253 (23), 251 (20), 233 (14), 207 (19), 151 (11), 127 (15), 115 (10), 106 (12), 91 (100), 65 (21). HRMS (ESI-TOF) m/z: [M + H]$^+$ Calcd for $C_{25}H_{20}F_2NO$ 388.1513; Found 388.1520.

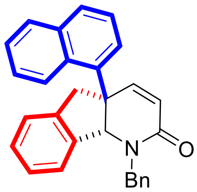

7e

(4a*RS*,9b*RS*)-1-Benzyl-4a-(naphthalen-1-yl)-1,4a,5,9b-tetrahydro-2*H*-indeno[1,2-*b*]pyridine-2-one (**7e**): Yield 50% (0.058 g). [Prepared from **6e** (0.140 g)]. The crude product purified by column chromatography (SiO_2, *n*-hexane : ethyl acetate, 5:1) gave pale brown solid. M.p. 162-163ºC. ^{1}H NMR ($CDCl_3$, 400 MHz): δ 7.70 – 7.58 (m, 2H, ArH), 7.53 (dd, 1H, J = 8.3, 6.9 Hz, ArH), 7.45 – 7.37 (m, 7H, ArH), 7.24 (dd, 1H, J = 7.2, 1.2 Hz, ArH), 7.10 – 6.92 (m, 3H, ArH), 6.63 – 6.45 (m, 2H, ArH), 6.29 (dd, 1H, J = 9.8, 1.2 Hz, =CH-4), 5.83 (d, 1H, J = 9.8 Hz, =CH-3), 5.69 (d, 1H, J = 14.6 Hz, NCH<u>H</u>), 5.18 (d, 1H, J = 1.2 Hz, CH-9b), 4.40 (d, 1H, J = 14.5 Hz, NC<u>H</u>H), 3.37 (d, 1H, J = 13.9 Hz, CH<u>H</u>-5), 2.95 (d, 1H, J = 13.9 Hz, C<u>H</u>H-5). ^{13}C{H} NMR ($CDCl_3$, 101 MHz): δ 162.8 (C=O), 145.1 (=CH-4), 144.1, 142.6, 137.8, 135.6, 135.5, 131.3, (Ar), 130.7 (2C), 129.1 (2C), 129.0 (2C), 128.2 (4C), 128.0, 126.6, 124.6, 123.9, (ArH), 121.0 (=CH-3), 119.8, 119.7 (ArH), 64.5 (CH-9b), 53.6 (C-4), 50.0 (NCH_2), 41.4 (CH_2-5). GC-MS (EI, 70eV): m/z = 401 (5) [M$^+$], 310 (20), 207 (18), 205 (38), 176 (10), 91 (100), 73 (11), 65 (10). HRMS (ESI-TOF) m/z: [M + Na]$^+$ Calcd for $C_{29}H_{23}NNaO$ 424.1677; Found 424.1684.

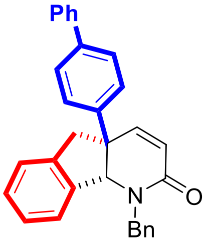

7f

(4a*RS*,9b*RS*)-4a-([1,1'-Biphenyl]-4-yl)-1-benzyl-1,4a,5,9b-tetrahydro-2*H*-indeno[1,2-*b*]pyridine-2-one (**7f**): Yield 81% (0.053 g). [Prepared from **6f** (0.078 g)]. The crude product purified by column chromatography (SiO_2, *n*-hexane : ethyl acetate, 6:1) gave pale yellow solid. M.p. 187-189ºC. ^{1}H NMR ($CDCl_3$, 400 MHz): δ 7.51 – 7.46 (m, 2H, ArH), 7.42 – 7.36 (m, 2H, ArH), 7.34 – 7.26 (m, 3H, ArH), 7.25 – 6.97 (m, 11H, ArH), 6.32 (dd, 1H, J = 9.9, 1.8 Hz, =CH-4), 6.02 (d, 1H, J = 9.9 Hz, =CH-3), 5.67 (d, 1H, J = 14.5 Hz, NCH<u>H</u>), 4.99 (d, 1H, J = 1.7 Hz, CH-9b), 4.12 (d, 1H, J = 14.5 Hz, NC<u>H</u>H), 3.54 (d, 1H, J = 15.7 Hz, CH<u>H</u>-5), 3.17 (d, 1H, J = 15.7 Hz, C<u>H</u>H-5). ^{13}C{H} NMR ($CDCl_3$, 101 MHz): δ 162.7 (C=O), 145.4 (=CH-4), 141.7 140.6, 140.4, 140.2, 138.8, 136.5, (Ar), 128.9 (2C), 128.7 (2C), 128.5 (2C), 128.4, 127.6, 127.5 (2C), 127.3 (2C), 127.1 (2C), 126.9 (2C), (ArH), 126.2 (=CH-3), 124.3 123.2, (ArH), 69.2 (CH-9b), 53.4 (C-4a), 50.4 (NCH_2), 44.8 (CH_2-5). GC-MS (EI, 70eV): m/z = 427 (80) [M$^+$], 410 (18), 336 (32), 322 (51), 320 (24), 294 (32), 293 (30), 291 (26), 265

(14), 215 (14), 207 (45), 191 (18), 165 (17), 115 (14), 91 (100), 65 (13). HRMS (ESI-TOF) m/z: [M + Na]$^+$ Calcd for $C_{31}H_{25}NNaO$ 450.1834; Found 450.1843.

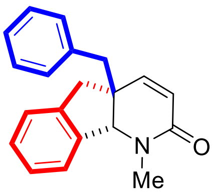

7g

(4a*RS*,9b*SR*)-4a-Benzyl-1-methyl-1,4a,5,9b-tetrahydro-2*H*-indeno[1,2-*b*]pyridine-2-one (**7g**): Yield 78% (0.046 g). [Prepared from **6g** (0.075 g)]. The crude product purified by column chromatography (SiO_2, *n*-hexane : ethyl acetate, 3:1) gave white semisolid. ^1H NMR ($CDCl_3$, 400 MHz): δ 7.37 – 7.07 (m, 9H, ArH), 6.06 (dd, 1H, *J* = 9.9, 1.2 Hz, =CH-4), 5.84 (d, 1H, *J* = 9.9 Hz, =CH-3), 4.63 (d, 1H, *J* = 1.2 Hz CH-9b), 3.29 (s, 3H, NCH_3), 3.18 (d, 1H, *J* = 15.4 Hz, CHH-5), 3.06 (d, 1H, *J* = 13.2 Hz, 4a-CHH), 2.97 (d, 1H, *J* = 13.2 Hz, 4a-CHH), 2.84 (d, 1H, *J* = 15.4 Hz, CHH-5). $^{13}C\{H\}$ NMR ($CDCl_3$, 101 MHz): δ 162.8 (C=O), 146.4 (ArH), 141.9, 138.8, 136.5 (Ar), 130.1 (2C), 128.4 (2C), 128.1, 127.2, 126.9, (ArH), 125.3 (=CH-3), 124.3, 123.3, (ArH), 69.5 (CH-9b), 50.9 (C-4a), 43.5, 42.2 (CH_2-5, 4a-CH_2), 36.1 (NCH_3). GC-MS (EI, 70eV): m/z = 289 (1) [M+], 199 (12), 198 (89), 197 (100), 169 (13), 128 (15), 115 (12), 91 (20). HRMS (ESI-TOF) m/z: [M + H]$^+$ Calcd for $C_{20}H_{20}NO$ 290.1545; Found 290.1557.

7. Synthesis of indenopyridine-2-thione **8b**

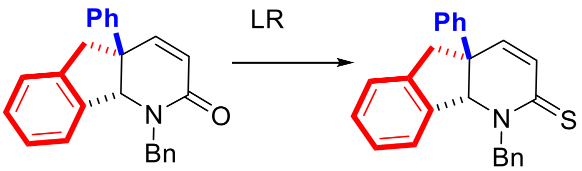

7b **8b**

Scheme S13

Lawesson's reagent (LR) (0.06 g, 0.14 mmol) was added to the solution of compound **7b** (0.089 g, 0.253 mmol) in dry toluene (5 mL). The resulting mixture was stirred for 1 h at 80°C and then concentrated to 1/3 volume under reduced pressure and in this form was applied to a column chromatography on silica gel using a mixture of *n*-hexane and ethyl acetate in a ratio of 10:1. Subsequently, it was additionally purified by column chromatography on silica gel using chloroform to afford compound **8b** in 98% yield (0.092 g), as yellow solid.

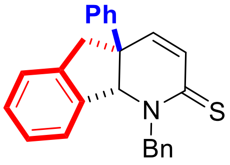

8b

(4a*RS*,9b*RS*)-1-Benzyl-4a-phenyl-1,4a,5,9b-tetrahydro-2*H*-indeno[1,2-*b*]pyridine-2-thione (**8b**): M.p. 170-171ºC (petroleum ether : ethyl acetate). ^1H NMR ($CDCl_3$, 400 MHz): δ 7.32 – 7.30 (m, 14H, ArH), 6.64 (d, 1H, *J* = 9.7 Hz, =CH-3), 6.61 (d, 1H, *J* = 14.4 Hz, NCHH), 6.04 (dd, 1H, *J* = 9.7, 1.6 Hz, =CH-4), 5.24 (d, 1H, *J* = 1.5 Hz, CH-9b), 4.55 (d, 1H, *J* = 14.4 Hz, NCHH), 3.60 (d, 1H, *J* = 15.7 Hz, CHH-5), 3.22

(d, 1H, J = 15.7 Hz, CHH-5).^{13}C{H} NMR ($CDCl_3$, 101 MHz): δ 188.7 (C=S), 140.5, 140.0, 139.1 (Ar), 136.0 (=CH-4), 134.9 (Ar), 132.8 (=CH-3), 128.7 (2C), 128.7, 128.5 (4C), 127.8, 127.6, 127.3, 126.4 (2C), 124.4, 122.9 (ArH), 70.8 (CH-9b), 57.5 (NCH_2), 53.9 (C-4a), 45.1 (CH_2-5). GC-MS (EI, 70eV): m/z = 367 (99) [M+], 335 (20), 334 (70), 276 (17), 243 (34), 229 (63), 215 (36), 185 (17), 152 (14), 115 (30), 91 (100), 89 (21). HRMS (ESI-TOF) m/z: $[M + H]^+$Calcd for $C_{25}H_{22}NS$, 368.1473; Found 368.1487.

8. General procedure for the synthesis of 3-iodopyridin-2-ones **9**

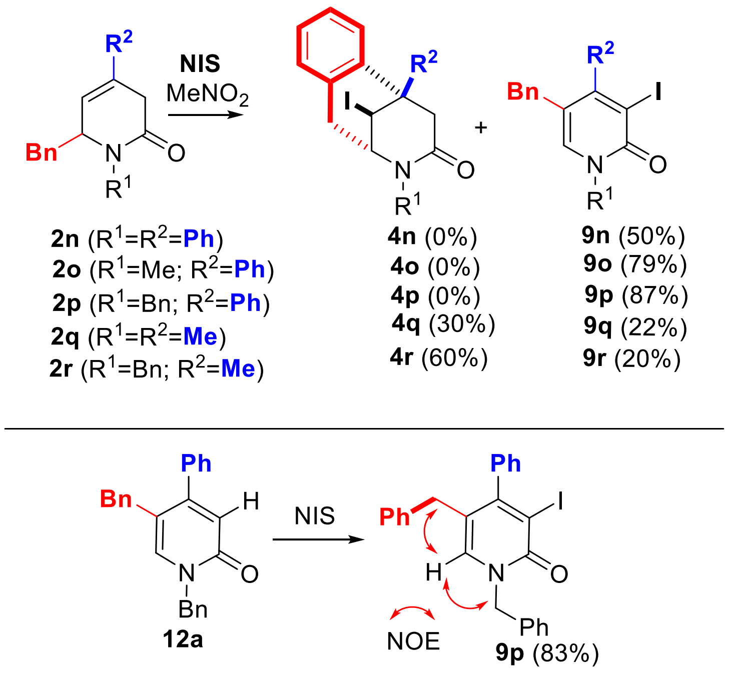

Scheme S14

To a stirred solution of 4-substituted-3,6-dihydropyridin-2(1*H*)-one **2** (1.86 mmol) in "wet" $MeNO_2$ (30 mL, commercial grade reagent)* placed in a 100 mL flask *N*-iodosuccinimide (2.09 g, 9.3 mmol, 5 equiv.)* was added and the resulting solution was stirred for 24 h at room temperature, in the dark. (In the case of a larger scale the reaction time may be prolongated even for few days). After the reaction is completed (TLC control) the mixture was quenched with a saturated aqueous solutions of $NaHCO_3$ (10 mL) and Na_2SO_3 (10 mL) and solution was stirred additional 10 minutes. The aqueous layer was extracted with ethyl acetate (3 × 60 mL), and the combined organic layers were dried over Na_2SO_4. The mixture was filtered, and the solvents were evaporated under reduced pressure. The crude product was purified by column chromatography on silica gel, using appropriate mixture of *n*-hexane and ethyl acetate. (* - unless specified otherwise)

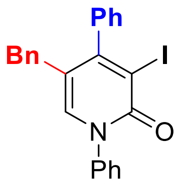

9n

5-Benzyl-3-iodo-1,4-diphenylpyridin-2(1*H*)-one (**9n**): Yield 50% (0.147 g). [Prepared from **2n** (0.215 g). $MeNO_2$ (10 mL, commercial grade reagent). Reaction time 21 h.] The crude product purified by column chromatography (SiO_2, *n*-hexane : ethyl acetate, 4:1) gave yellow brown semisolid ^{1}H NMR ($CDCl_3$, 400 MHz): δ 7.53 – 7.30 (m, 8H, 2x C_6H_5), 7.21 – 7.13 (m, 3H, C_6H_5), 7.12 (s, 1H, CH-6), 7.03 – 6.92 (m, 2H, C_6H_5), 6.83 (dd, 2H, *J* = 7.6, 1.9 Hz, C_6H_5), 3.52 (s, 2H, CH_2). ^{13}C{H} NMR ($CDCl_3$, 101 MHz): δ 160.1, 159.2 (C-2, C-4), 141.7, 141.1, 138.8 (C_6H_5), 136.2 (CH-6), 129.3 (2C), 128.62, 128.60 (2C), 128.5 (2C), 128.4 (2C), 128.3, 127.5 (2C), 126.5 (2C), 126.4, (C_6H_5), 118.7 (C-5), 100.7 (C-3), 37.3 (CH_2). GC-MS (EI, 70eV): m/z = 463 (100) [M^+], 462 (13), 308 (10), 258 (15), 230 (14), 215 (10), 203 (16), 202 (23), 104 (12), 91 (28), 77 (36). HRMS (ESI-TOF) m/z: $[M + H]^+$ Calcd for $C_{24}H_{19}INO$ 464.0511; Found 464.0515.

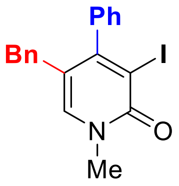

9o

5-Benzyl-3-iodo-1-methyl-4-phenylpyridin-2(1*H*)-one (**9o**): Yield 79% (0.230 g). [Prepared from **2o** (0.203 g). $MeNO_2$ (7 mL, commercial grade reagent).] The crude product purified by column chromatography (SiO_2, *n*-hexane : ethyl acetate, 1:1) gave white solid. M.p. 176-179ºC. ^{1}H NMR ($CDCl_{3,}$ 400 MHz): δ 7.38 – 7.34 (m, 3 H, ArH), 7.22 – 7.14 (m, 3 H, ArH), 6.97 (s, 1 H, =CH-6), 6.95 – 6.91 (m, 2 H, 2 x H'-2), 6.83 (dd, 2 H, *J* = 7.5, 1.8 Hz, 2 x H"-2), 3.63 (s, 3 H, NCH_3), 3.48 (s, 2,H, CH_2). ^{13}C{H} NMR ($CDCl_3$, 101 MHz): δ 159.9 (C-2), 159.6 (C-4), 141.8 (C-1'), 139.0 (C-1"), 136.8 (CH-6), 128.7 (C-2"), 128.5, 128.4 (C-3', C-3"), 128.2 (C-4'), 127.5 (C-2'), 126.4 (C-4"), 118.6 (C-5), 99.4 (C-3), 39.2 (CH_3), 37.2 (CH_2). GC-MS (EI, 70eV): m/z = 401 (100) [M^+], 244 (13), 202 (17), 196 (13), 168 (10), 115 (10), 91 (18). HRMS (ESI-TOF) m/z: $[M + H]^+$ Calcd for $C_{19}H_{17}INO$ 402.0355; Found 402.0362.

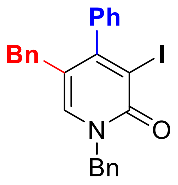

9p

1,5-Dibenzyl-3-iodo-4-phenylpyridin-2(1*H*)-one (**9p**): Yield 87% (1.503 g). [Prepared from **2p** (1.28 g). $MeNO_2$ (58 mL, commercial grade reagent). NIS (2.45 g, 10.9 mmol, 3 equiv. Reaction time 5 days.] The crude product purified by column chromatography (SiO_2, *n*-hexane : ethyl acetate, 3:1) gave white solid. M.p. 155-157ºC. ^{1}H NMR ($CDCl_3$, 400 MHz): δ 7.38 – 7.30 (m, 8 H, ArH), 7.17 – 7.11 (m, 3 H, ArH), 7.00 (s, 1 H, H-6), 6.94 – 6.89 (m, 2 H, H'-2), 6.75 (dd, 2 H, *J* = 6.5, 2.8 Hz, 2 x H"-2), 5.19 (s, 2 H, NCH_2), 3.44 (s, 2 H, CH_2). ^{13}C{H} NMR ($CDCl_3$, 101 MHz): δ 159.5 (C-2, C-4), 141.7 (C-1'), 138.9 (C-1"), 136.0 (NBn), 135.7 (CH-6), 128.9 (C-2"), 128.59, 128.57, 128.42, 128.38, 128.34, 128.25 (NBn, C-3', C-3"), 128.2 (C-4'), 127.5 (C-2'), 126.4 (C-4"), 118.8 (C-5), 100.0 (C-3), 54.0 (NCH_2), 37.3 (CH_2). GC-MS (EI, 70eV): m/z = 477 (63) [M^+], 476 (21), 370 (10), 202 (12), 91 (100), 65 (12). HRMS (ESI-TOF) m/z: $[M + H]^+$ Calcd for $C_{25}H_{21}INO$ 478.0668; Found 478.0675.

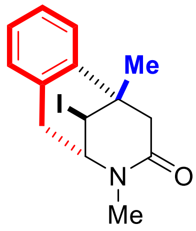

4q

(2*RS*,6*SR*,11*SR*)-11-Iodo-3,6-dimethyl-2,3,5,6-tetrahydro-2,6-methanobenzo[d]azocin-4(1*H*)-on (**4q**): Yield 30% (0.231 g). [Prepared from **2q** (0.486 g). $MeNO_2$ (36 mL, commercial grade reagent). Reaction time 24 h.] The crude product purified by column chromatography (SiO_2, *n*-hexane : ethyl acetate, 4:1 then 1:1) gave yellow solid. M.p. 106-107ºC. ^1H NMR ($CDCl_3$, 400 MHz): δ 7.45 – 7.37 (m, 1H, CH-7), 7.24 – 7.14 (m, 2H, CH-8, CH-9), 7.07 – 6.99 (m, 1H, CH-10), 4.79 (t, 1H, *J* = 2.0 Hz, CH-11), 4.17 (dt, 1H, *J* = 4.0, 2.5, 2.0 Hz, CH-2), 3.25 (dd, 1H, *J* = 17.2, 4.0 Hz, $CH\underline{H}_{\alpha}$-1), 3.03 (dd, 1H, *J* = 17.3, 2.5 Hz, $CH\underline{H}_{\beta}$-1), 3.00 (s, 3H, NCH_3), 2.83 (d, 1H, *J* = 17.5 Hz, $CH\underline{H}_{\beta}$-5), 2.50 (dd, 1H, *J* = 17.5, 2.0 Hz, $CH\underline{H}_{\alpha}$-5), 1.56 (s, 3H 6-CH_3). $^{13}C\{H\}$ NMR ($CDCl_3$, 101 MHz): δ 168.0 (C=O), 138.5 (C-6a), 130.3 (C-10a), 129.5 (CH-10), 127.6, 127.4 (CH-8, CH-9), 127.1 (CH-7), 64.5 (CH-2), 46.7 (CH_2-5), 40.7 (CH-11), 38.9 (C-6), 36.3 (CH_2-1), 33.9 (NCH_3), 29.1 (6-CH_3). GC-MS (EI, 70 eV); m/z = 341 (6) [M^+], 214 (100), 143 (15), 141 (15), 131 (18), 129 (22), 128 (22), 115 (19), 84 (10), 69 (8). HRMS (ESI-TOF) m/z: $[M + H]^+$ Calcd for $C_{14}H_{17}INO$ 342.0355; Found 342.0360.

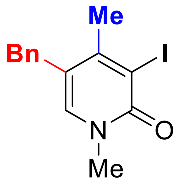

9q

5-Benzyl-3-iodo-1,4-dimethylpyridin-2(1*H*)-one (**9q**): Yield 22% (0.168 g). [Prepared from **2q** (0.486 g). $MeNO_2$ (36 mL, commercial grade reagent).] The crude product purified by column chromatography (SiO_2, *n*-hexane : ethyl acetate, 4:1 than 1:1) gave white solid. M.p. 120-121ºC. ^1H NMR ($CDCl_3$, 400 MHz): δ 7.35 – 7.20 (m, 3H, C_6H_5), 7.11 (d, 2H, *J* = 7.2 Hz, C_6H_5), 6.93 (s, 1H, CH-6), 3.8 (s, 2H, 5-CH_2), 3.57 (s, 3H, N-CH_3), 2.30 (s, 3H, 4-CH_3). $^{13}C\{H\}$ NMR ($CDCl_3$, 101 MHz): δ 159.8, 155.1 (C-2, C-4), 138.6 (C_6H_5), 136.2 (CH-6), 128.8 (2C), 128.4 (2C), 126.7, (C_6H_5), 117.9 (C-5), 100.2 (C-3), 38.9 (N-CH_3), 37.1 (5-CH_2), 26.6 (4-CH_3). GC-MS (EI, 70 eV); m/z = 339 (100) [M^+], 262 (9), 212 (8), 184 (11), 141 (9), 128 (13), 115 (10), 91 (9). HRMS (ESI-TOF) m/z: $[M + H]^+$ Calcd for $C_{14}H_{15}INO$ 340.0198; Found 340.0210.

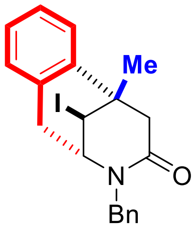

4r

(2*RS*,6*SR*,11*SR*)-3-Benzyl-11-iodo-6-methyl-2,3,5,6-tetrahydro-2,6-methanobenzo[d]azocin-4(1*H*)-one(**4r**): Yield 60% (0.134 g). [Prepared from **2r** (0.156 g). $MeNO_2$ (10 mL, commercial grade reagent). Reaction time 48 h.] The crude product purified by column chromatography (SiO_2, *n*-hexane : ethyl acetate, 6:1) gave white solid. M.p. 141-143ºC. ^1H NMR ($CDCl_3$, 400 MHz): δ 7.41 (dd, 1H, *J* = 7.6, 1.6 Hz, ArH), 7.38 – 7.27 (m, 5H, ArH), 7.25 – 7.14 (m, 2H, ArH), 7.00 – 6.95 (m, 1H, ArH), 5.32 (d, 1H, *J* = 14.9 Hz, $NCH\underline{H}$), 4.75 (t, 1H, *J* = 2.1 Hz, CH-11), 4.09 (dt, 1H, *J* = 4.0, 2.1 Hz, CH-2), 4.02 (d, 1H, *J* = 14.9 Hz, $NC\underline{H}H$), 3.16 (dd, 1H, *J* = 17.2, 4.0 Hz, $CH\underline{H}$-5), 2.96 (dd, 1H, *J* = 17.2, 2.1 Hz, $C\underline{H}H$-5), 2.93 (d, *J* = 17.7 Hz, 1H, CHH_{β}-5), 2.61 (dd, *J* = 17.7, 2.0 Hz, 1H, $CH\underline{H}_{\alpha}$-5), 1.55 (s, 3H,

6-CH_3). $^{13}C\{H\}$ NMR ($CDCl_3$, 101 MHz): δ 168.2 (C=O), 138.6, 136.3, 130.5 (Ar), 129.5, 128.8 (2C), 128.4 (2C), 127.7 (2C), 127.4, 127.2, (ArH), 60.6 (CH-2), 48.1 (NCH_2), 46.9 (CH_2-5), 40.8 (CHI-11), 38.7 (C-6), 36.5 (CH_2-1), 29.0 (6-CH_3). GC-MS (EI, 70 eV); m/z = 417 (41) [M^+], 290 (19), 143 (35), 141 (13), 128 (24), 115 (14), 91 (100), 65 (10). HRMS (ESI-TOF) m/z: $[M + Na]^+$ Calcd for $C_{20}H_{20}INNaO$ 440.0487; Found 440.0498.

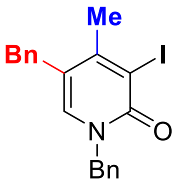

9r

1,5-Dibenzyl-3-iodo-4-methylpyridin-2(1*H*)-one (**9r**): Yield 20% (0.045 g). [Prepared from **2r** (0.156 g). $MeNO_2$ (10 mL, commercial grade reagent). Reaction time 48h.] The crude product purified by column chromatography (SiO_2, *n*-hexane : ethyl acetate, 6:1) gave white solid. M.p. 139-140°C. ^1H NMR ($CDCl_3$, 400 MHz): δ 7.37 – 7.19 (m, 8H, ArH), 7.09 – 7.01 (m, 2H, ArH), 6.93 (s, 1H, CH-6), 5.16 (s, 2H, NCH_2), 3.78 (s, 2H, 5-CH_2), 2.29 (s, 3H, 4-CH_3). $^{13}C\{H\}$ NMR ($CDCl_3$, 101 MHz): δ 159.4 (C-2), 155.1 (C-4), 138.6, 136.1 (Ar), 135.0 (CH-6), 128.9 (2C), 128.8 (2C), 128.4 (2C), 128.3 (2C), 128.2, 126.7 (ArH), 118.2 (C-5), 100.8 (C-3), 53.7 (NCH_2), 37.2 (5-CH_2), 26.7 (4-CH_3). GC-MS (EI, 70 eV); m/z = 415 (46) [M^+], 324 (97), 197 (9), 182 (19), 167 (56), 154 (10), 91 (100), 65 (22). HRMS (ESI-TOF) m/z: $[M + H]^+$ Calcd for $C_{20}H_{19}INO$ 416.0511; Found 416.0504.

9. Procedure for the synthesis of 2-pyridones **10a**, **10b**

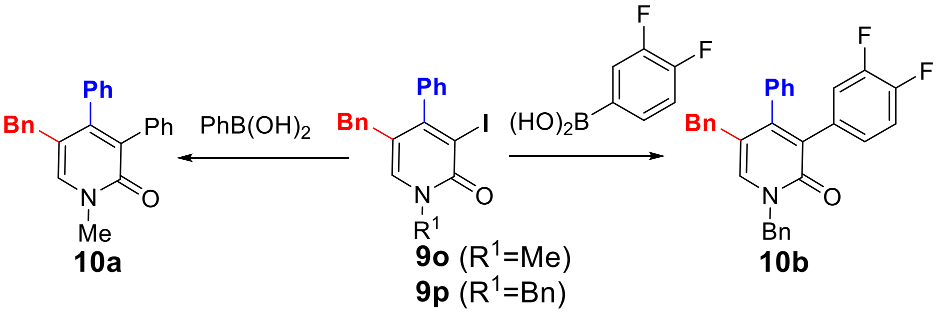

Scheme S15

To a 25-mL Schlenk flask, equipped with a magnetic stir bar and a condenser crowned with argon balloon, charged with 10 mL of 5 : 3 : 2 mixture of toluene, ethanol and water (degassed for 2 h using a stream of argon slowly bubbled through the solution at rt. upon vigorous stirring), 5-benzyl-3-iodo-4-phenylpyridin-2 (1*H*)-ones (**9o** or **9p**) (0.251 mmol), arylboronic acid (0.30145 mmol), Na_2CO_3 (0.1065 g, 1.05 mmol) and $PdCl_2P(Ph_3)_2$ (0.0049 mmol) as catalyst was added. The mixture was stirred at bath temp. 80°C for 19h. After this time the reaction mixture was cooled to rt. And aqueous saturated NaCl (5 mL) was added, the mixture was extracted with ethyl acetate (3 x 15 mL) and the combined organic layers were dried over $MgSO_4$. Filtration, concentration *in vacuo* and purification by flash column chromatography (silica gel, *n*-hexane : ethyl acetate, 1 : 1) gave **10a** or **10b** as white solid.

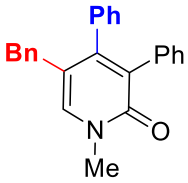

10a

6-Benzyl-1-methyl-4,5-diphenylpyridin-2(1*H*)-one (**10a**): Yield 83%. M.p. 174-176ºC. ^{1}H NMR ($CDCl_3$, 400 MHz): δ 7.23 – 6.99 (m, 12 H, ArH, =CH-6), 6.91 – 6.88 (m, 2 H, ArH), 6.88 – 6.84 (m, 2 H, ArH), 3.58 (s, 3 H, CH_3), 3.52 (s, 2 H, CH_2). ^{13}C{H} NMR ($CDCl_3$, 101 MHz): δ 161.8 (C-2), 151.7 (C-4), 139.5, 137.3 (Ar), 135.9 (CH-6), 135.8 (Ar), 131.3 (C-3), 130.7 (2C), 129.0 (2C), 128.8 (2C), 128.4 (2C), 127.7 (2C), 127.3 (2C), 127.1, 126.6, 126.2, (ArH), 118.2 (C-5), 38.1 (NCH_3), 36.7 (5-CH_2). GC-MS (EI, 70eV): m/z = 351 (67) [M^+], 350 (100). HRMS (ESI-TOF) m/z: $[M + H]^+$ Calcd for $C_{25}H_{22}NO$, 352.1701; Found 352.1705.

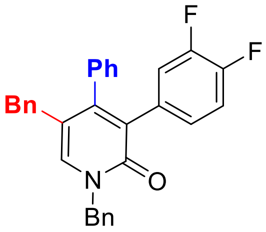

10b

1,5-Dibenzyl-3-(3,4-difluorophenyl)-4-phenylpyridin-2(1*H*)-one (**10b**): Yield 83%. M.p. 96-98ºC. ^{1}H NMR ($CDCl_3$, 400 MHz): δ 7.39 – 7.31 (m, 5 H, ArH), 7.19 – 7.10 (m, 6 H, ArH), 7.08 (s, 1 H, =CH-6), 6.93 – 6.84 (m, 2 H, ArH), 6.84 – 6.78 (m, 4 H, ArH), 6.76 – 6.71 (m, 1 H, ArH), 5.17 (s, 2 H, NCH_2), 3.48 (s, 2 H, 5-CH_2). ^{13}C{H} NMR ($CDCl_3$, 101 MHz): δ 161.0 (C-2), 152.2 (C-4), 149.3 (J_{CF} = 246.5, 12.5 Hz), 149.1, 139.2, 136.7, (J_{CF} = 248, 12.5 Hz), 136.3 (Ar), 135.4 (CH-6), 132.7 (J_{CF} = 6.6, 4.4 Hz), 129.5 (C-5), 128.9, 128.8, 128.6, 128.6, 128.3, 128.2, 128.1, 128.0, 127.5, 127.1 (J_{CF} = 6.2, 3.3 Hz), 126.3, (ArH, Ar), 119.9 (J_{CF} = 17.6 Hz), 118.5 (C-3), 116.2 (J_{CF} = 16.9 Hz), 52.9 (NCH_2), 36.7 (5-CH_2). ^{19}F NMR (377 MHz, $CDCl_3$): δ -140.13 (d, *J* = 21.5 Hz), -139.20 (d, *J* = 21.7 Hz). GC-MS (EI, 70 eV); m/z =463 (51) [M^+], 462 (36), 356 (10), 294 (8), 92 (8), 91 (100). HRMS (ESI-TOF) m/z: $[M + H]^+$ Calcd for $C_{31}H_{24}F_2NO$ 464.1826; Found 464.1826.

10. Preparation of 2-pyridones **11a**, **11b**

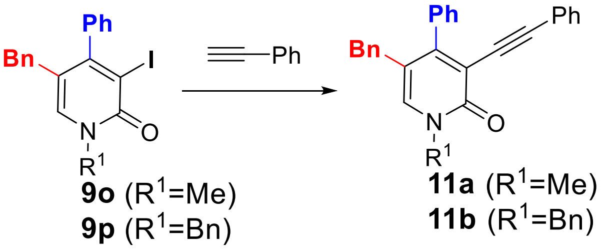

Scheme S16

To a 25-mL flask equipped with a magnetic stir bar and a condenser crowned with argon pyridin, charged with a mixture of anhydrous DMF (2 mL) 3-iodo-4-phenylpyridin-2(1*H*)-one 8 (0.314 mmol), degassed for 2 h using a stream of argon slowly bubbled through the solution at rt. During vigorous stirring, phenylacetylene (0.41 mmol), $Pd(PPh_3)_2Cl_2$ (3.3 mg), CuI (0.5 mg) were added. Subsequently

anhydrous Et_3N (0.375 mL) was added over 10 minutes and the mixture was heated at 50ºC for 24 hours. After this time the reaction mixture was cooled to rt. And aqueous saturated NaCl (5 mL) was added, the mixture was extracted with ethyl acetate (3 x 15 mL) and the combined organic layers were washed with brine (5 mL), dried over $MgSO_4$ and filtered through the pad of Celite. Concentration in vacuo and purification by flash column chromatography on silica gel using a mixture of appropriate solvents to give the desired product.

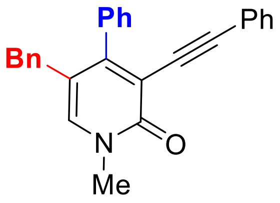

11a

5-Benzyl-1-methyl-4-phenyl-3-(phenylethynyl)pyridine-2(1*H*)-on (**11a**): Yield 50% (0.070 g). Prepared from **9o** (0.15 g). The crude product purified by column chromatography (SiO_2, *n*-hexane : ethyl acetate, 1:1) gave pale yellow solid. M.p. 174-175ºC. ^{1}H NMR ($CDCl_3$, 400 MHz): δ 7.41 – 7.36 (m, 3H, ArH), 7.24 – 7.14 (m, 8H, ArH), 7.12 – 7.07 (m, 2H, ArH), 7.00 (s, 1H, CH-6), 6.93 – 6.87 (m, 2H, ArH), 3.58 (s, 3H, NCH_3), 3.55 (s, 2H, 5-CH_2). ^{13}C{H} NMR ($CDCl_3$, 101 MHz): δ 161.4 (C=O), 156.7 (C-6), 139.2, 137.3 (Ar), 136.6 (CH-6), 131.5 (2C), 128.7 (2C), 128.5 (4C), 128.1 (2C), 128.0, 128.0 (3C), 126.4 (ArH), 123.4 (Ar), 118.2 (C-5), 115.2 (C-3), 98.4, 85.5 (C≡C), 38.1 (NCH_3), 36.2 (5-CH_2). GC-MS (EI, 70 eV); m/z = 375 (63) [M^+], 374 (100), 298 (7), 226 (7), 148 (11), 91 (6). HRMS (ESI-TOF) m/z: $[M + H]^+$ Calcd for $C_{27}H_{22}NO$ 376.1701; Found 376.1703.

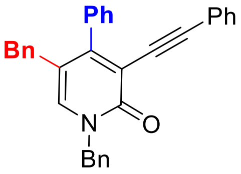

11b

1,5-Dibenzyl-4-phenyl-3-(phenylethynyl)pyridine-2(1*H*)-one (**11b**): Yield 95% (0.135 g). Prepared from **9p** (0.111 g). The crude product purified by column chromatography (SiO_2, *n*-hexane : ethyl acetate, 3:1) gave yellow solid. M.p. 185-187ºC. ^{1}H NMR ($CDCl_3$, 400 MHz): δ 7.41 – 7.28 (m, 8H, ArH), 7.22 – 7.11 (m, 8H, ArH), 7.11 – 7.06 (m, 2H, ArH), 7.04 (s, 1H, CH-6), 6.84 – 6.78 (m, 2H, ArH), 5.17 (s, 2H, NCH_2), 3.51 (s, 2H, 5-CH_2). ^{13}C{H} NMR ($CDCl_3$, 101 MHz): δ 161.0 (C=O), 156.6 (C-6), 139.1, 137.2, 136.1 (Ar), 135.5 (CH-6), 131.5 (2C), 128.9 (2C), 128.6 (2C), 128.4 (2C), 128.4 (2C), 128.4 (2C), 128.1, 128.04 (2C), 128.0 (2C), 128.01, 127.98, 126.3 (ArH), 123.4 (Ar), 118.5 (C-5), 115.6 (C-3), 98.5, 85.6, (C≡C), 52.7 (NCH_2), 36.3 (5-CH_2). GC-MS (EI, 70 eV); m/z = 451 (57) [M^+], 450 (32), 360 (16), 282 (44), 254 (23), 226 (10), 91 (100), 65 (12). HRMS (ESI-TOF) m/z: $[M + Na]^+$ Calcd for $C_{33}H_{25}NNaO$ 474.1834; Found 474.1835.

11. Procedure for the synthesis of 2-pyridones **12a** and **12b**

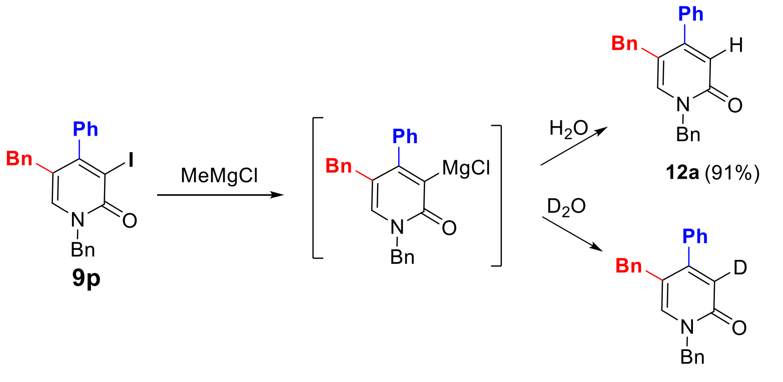

Scheme S17

To a cooled (0°C) and stirred solution of **9p** (0.31 mmol, 0.150 g) in 8 mL anhydrous THF, in 50-mL Schlenk flask, a solution of methylmagnesium chloride (3.0 M w THF; 0.372 mmol, 0,12 mL, 1.2 equiv) was added. The mixture was stirred at 0°C for 30 min. After this time aqueous saturated NH_4Cl solution (5 mL) (product **12a**) or D_2O (10 equiv, product **12b**). Then the mixture was extracted with ethyl acetate (3 x 10 mL) and the combined organic layers were dried over $MgSO_4$. Filtration, concentration in vacuo and purification by flash column chromatography on silica gel, using a mixture of appropriate solvents yielded desired product.

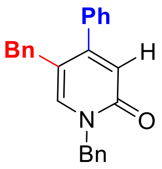

1,5-Dibenzyl-4-phenylpyridin-2(1*H*)-on (**12a**): Yield 91%. The crude product purified by column chromatography (SiO_2, *n*-hexane : ethyl acetate, 3:1) gave yellow oil. ^{1}H NMR ($CDCl_3$, 400 MHz): δ 7.44 – 7.27 (m, 8H, C_6H_5), 7.15 – 7.11 (m, 5H, C_6H_5), 7.02 (s, 1H, CH-6), 6.83 (dd, 1H, *J* = 7.3, 2.0 Hz, C_6H_5), 6.61 (s, 1H, CH-3), 5.14 (s, 2H, N-CH_2), 3.61 (s, 2H, 5-CH_2). ^{13}C{H} NMR ($CDCl_3$, 101 MHz): δ 161.7 (C-2), 155.2 (C-4), 139.3, 137.8 (C_6H_5), 136.6 (CH-6), 136.2, 128.9 (2C), 128.6 (2C), 128.5 (2C), 128.27 (2C), 128.25, 128.25 (2C), 128.1, 128.0 (2C), 126.3 (C_6H_5), 120.4 (CH-3), 52.1 (N-CH_2), 35.9 (5-CH_2). GC-MS (EI, 70 eV); m/z = 351 (100) [M^+], 350 (69), 274 (22), 245 (35), 202 (11), 91 (82). HRMS (ESI-TOF) m/z: $[M + H]^+$ Calcd for $C_{25}H_{22}NO$ 352.1701; Found 352.1700.

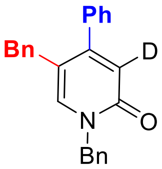

1,5-Dibenzyl-4-phenylpyridin-2(1*H*)-one-*3-d* (**12b**): Yield 79%. The crude product purified by column chromatography (SiO_2, *n*-hexane : ethyl acetate, 2:1) gave yellow oil. ^{1}H NMR ($CDCl_3$, 400 MHz): δ 7.38 – 7.26 (m, 8H, C_6H_5), 7.18 – 7.09 (m, 5H, C_6H_5), 7.03 (s, 1H, CH-6), 6.83 (dd, 2H, *J* = 7.5, 2.0 Hz, C_6H_5), 5.14 (s, 2H, NCH_2), 3.61 (s, 2H, 5-CH_2). ^{13}C{H} NMR ($CDCl_3$, 101 MHz): δ 161.7, 154.9 (C-2, C-4), 139.4, 137.9 (C_6H_5), 136.5 (CH-6), 136.3, 128.9 (2C), 128.5 (2C), 128.4 (2C), 128.3 (2C), 128.3, 128.2

(2C), 128.04, 127.98 (2C), 126.3 (3 x C_6H_5), 120.1 (J_{CD} = 25.3 Hz, CD-3), 118.6 (C-5), 51.9 (NCH_2), 35.8 (5-CH_2). GC-MS (EI, 70 eV); m/z = 352 (100) [M^+], 351 (73), 275 (22), 246 (35), 203 (10), 91 (81). HRMS (ESI-TOF) m/z: $[M + H]^+$ Calcd for $C_{25}H_{21}DNO$ 353.1764; Found 353.1780.

12. Study of **9p** formation by following of the progress of the reaction between **2p** and NIS with the aid of ^1H NMR spectrocsopy

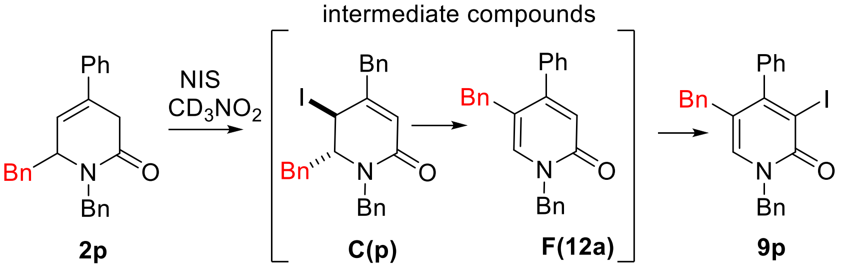

Scheme S17. Reaction of **2p** with NIS in CD_3NO_2 investigated by ^1H NMR measurements (see Fig.S1)

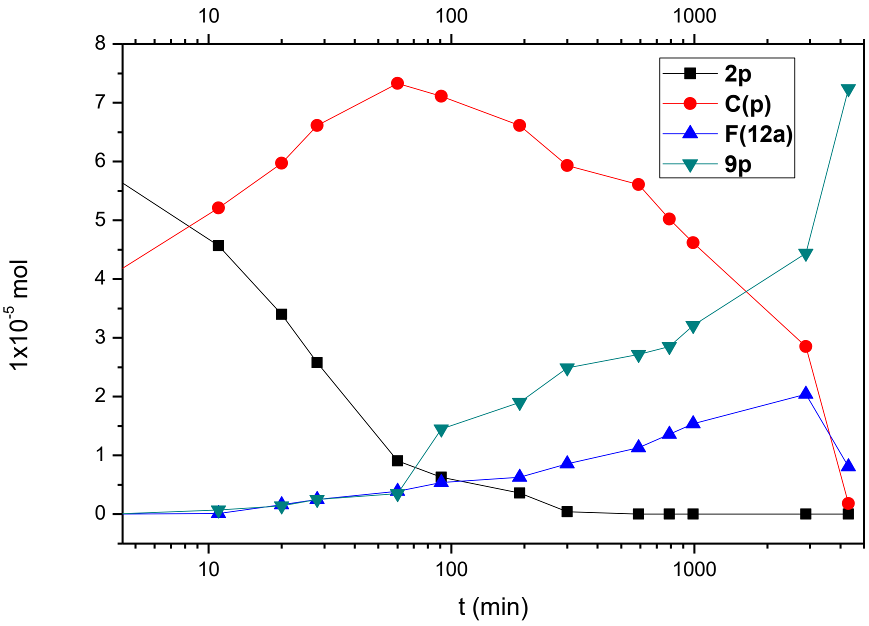

Figure S1. The composition of the reaction mixture (scheme S17) conducted in the NMR tube, starting from **2p**, quantified by ^1H NMR spectra at different time intervals. (The time axis is shown on a logarithmic scale for better readability across the entire range of reaction time.)

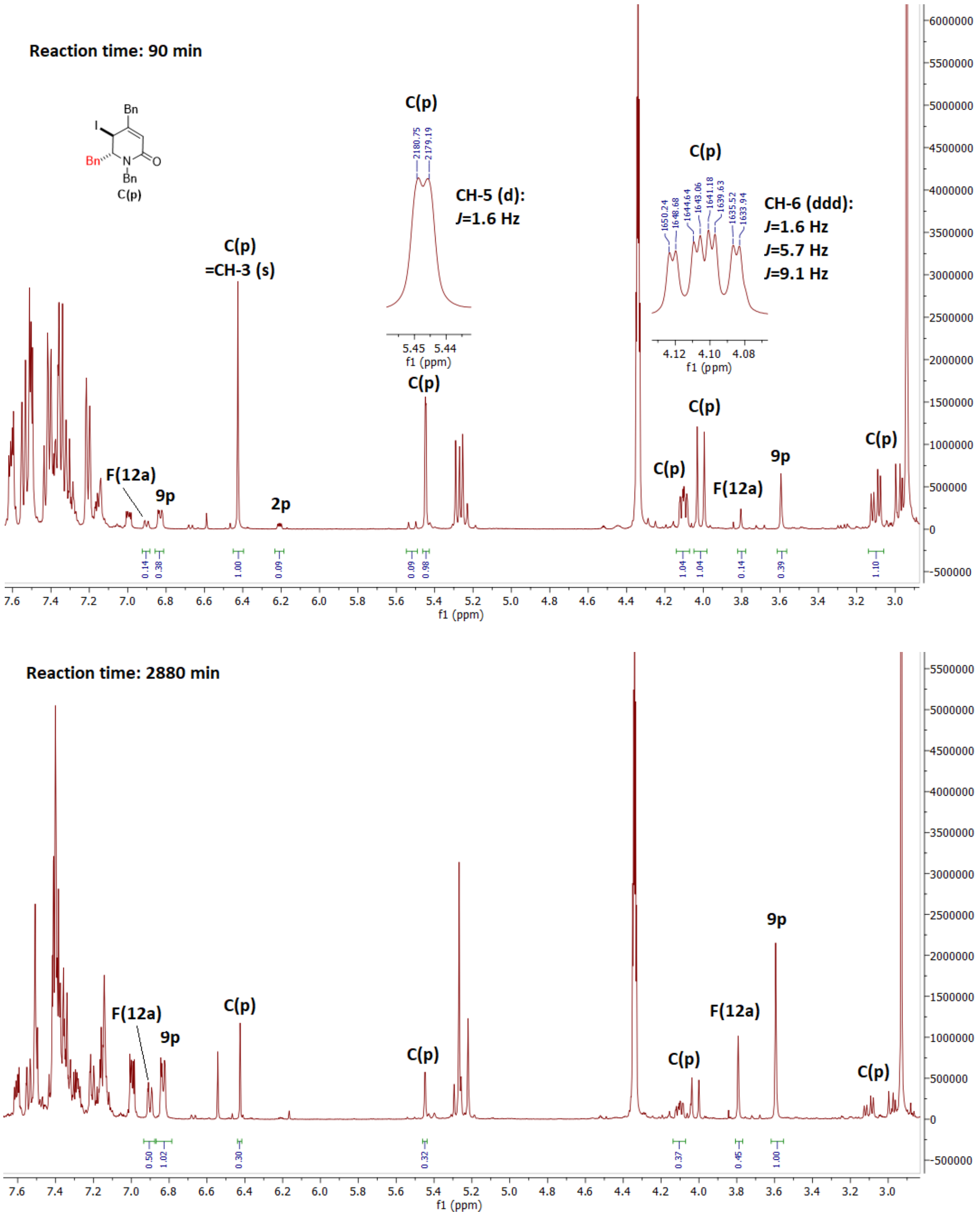

Figure S2. Representative ^{1}H NMR spectra recorded after 90 min (upper part) and 2880 min (lower part) of the reaction of **2p** with NIS (2.2 equiv.) conducted in NMR tube in CD_3NO_2 at rt.

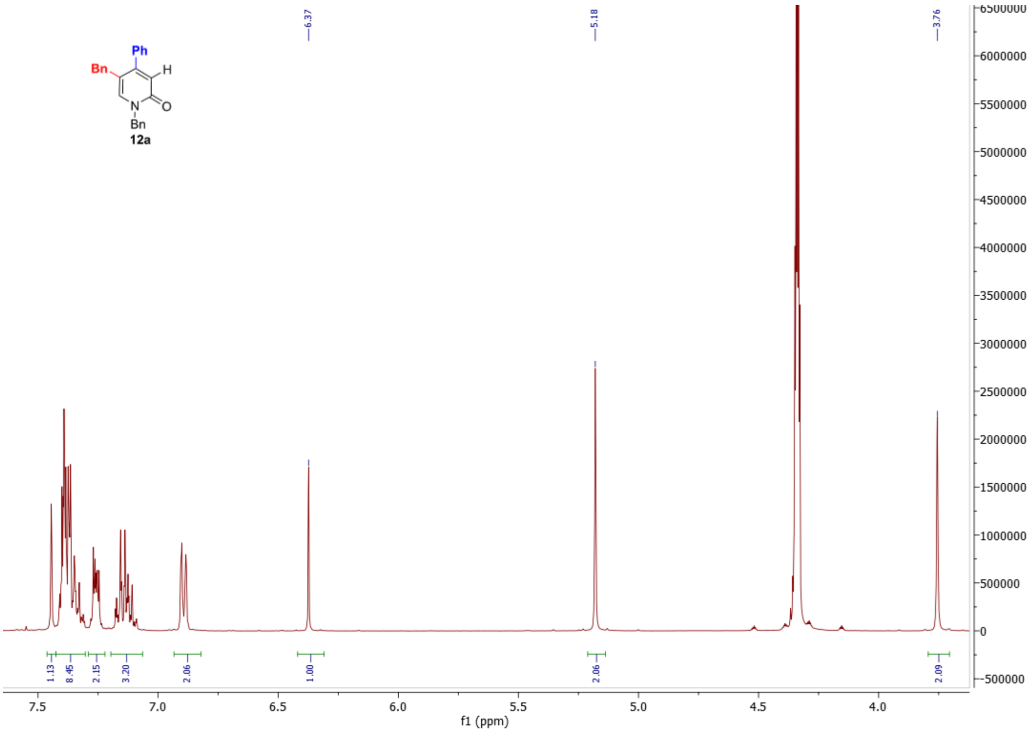

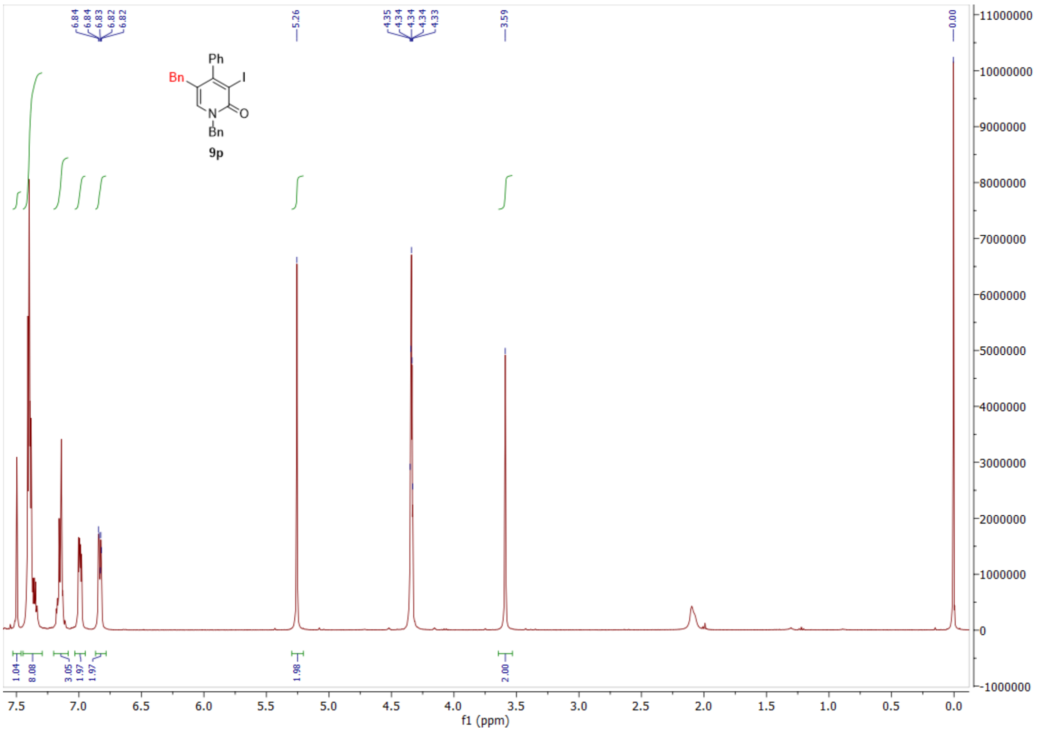

Figure S3. ^{1}H NMR spectra of **12a** and **9p** taken in CD_3NO_2 as references (see fig. S2)

13. ^{1}H and ^{13}C NMR spectra

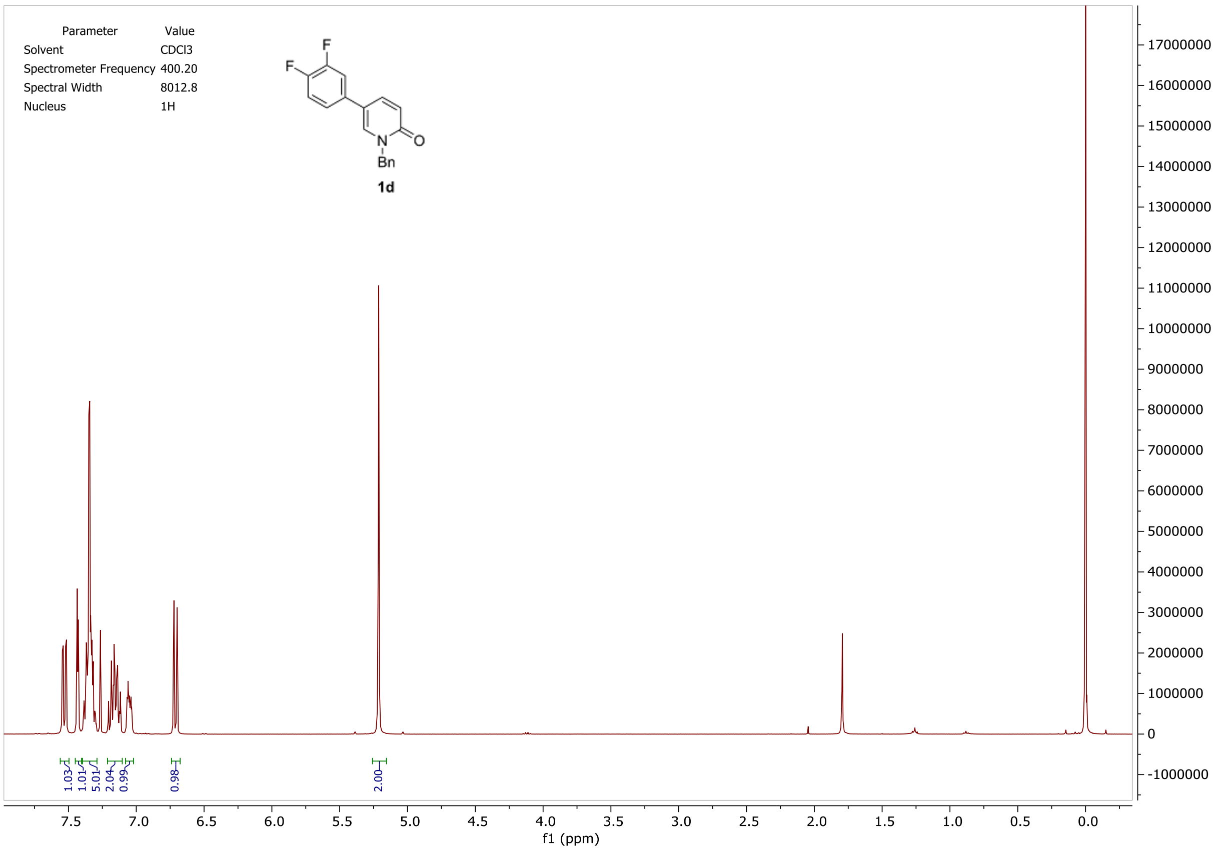
Parameter Value
Solvent CDCl3
Spectrometer Frequency 400.20
Spectral Width 8012.8
Nucleus 1H
1d
1.03 1.01 5.01 2.04 0.99 0.98 2.00
f1 (ppm)

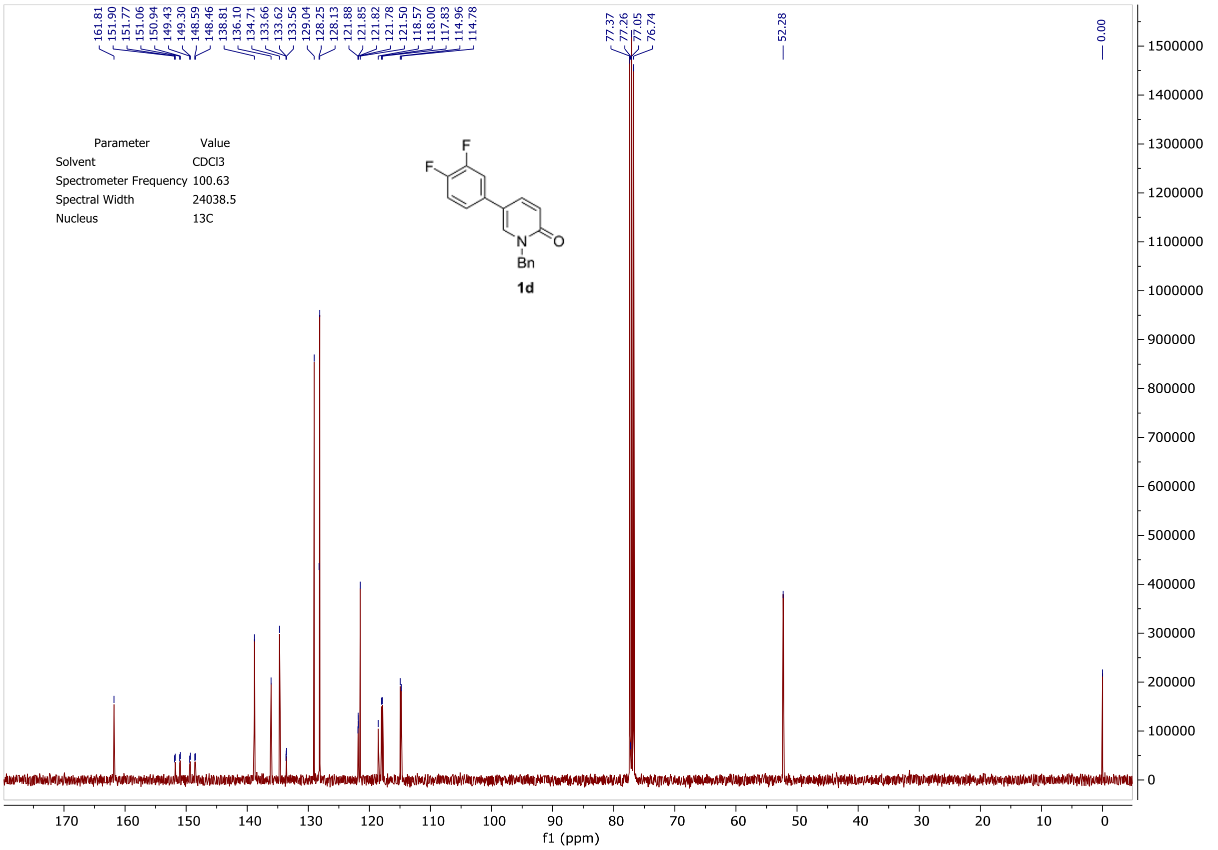
161.81 151.90 151.77 151.06 150.94 149.43 149.30 148.59 148.46 138.81 136.10 134.71 133.66 133.62 133.56 129.04 128.25 128.13 121.88 121.85 121.82 121.78 121.50 118.57 118.00 117.83 114.96 114.78
77.37 77.26 77.05 76.74
52.28
0.00
Parameter Value
Solvent CDCl3
Spectrometer Frequency 100.63
Spectral Width 24038.5
Nucleus 13C
1d
f1 (ppm)

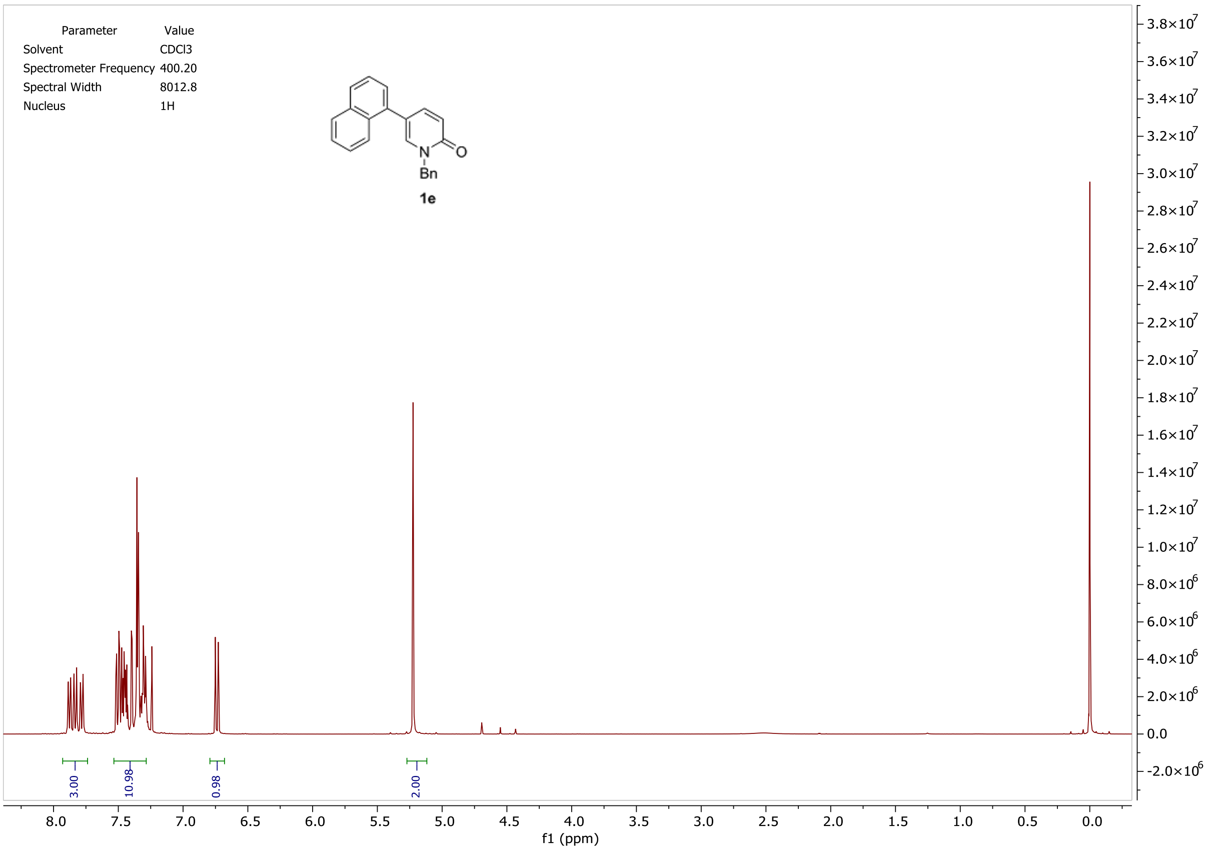
Parameter Value
Solvent CDCl3
Spectrometer Frequency 400.20
Spectral Width 8012.8
Nucleus 1H
N
O
Bn
1e
3.00
10.98
0.98
2.00
f1 (ppm)

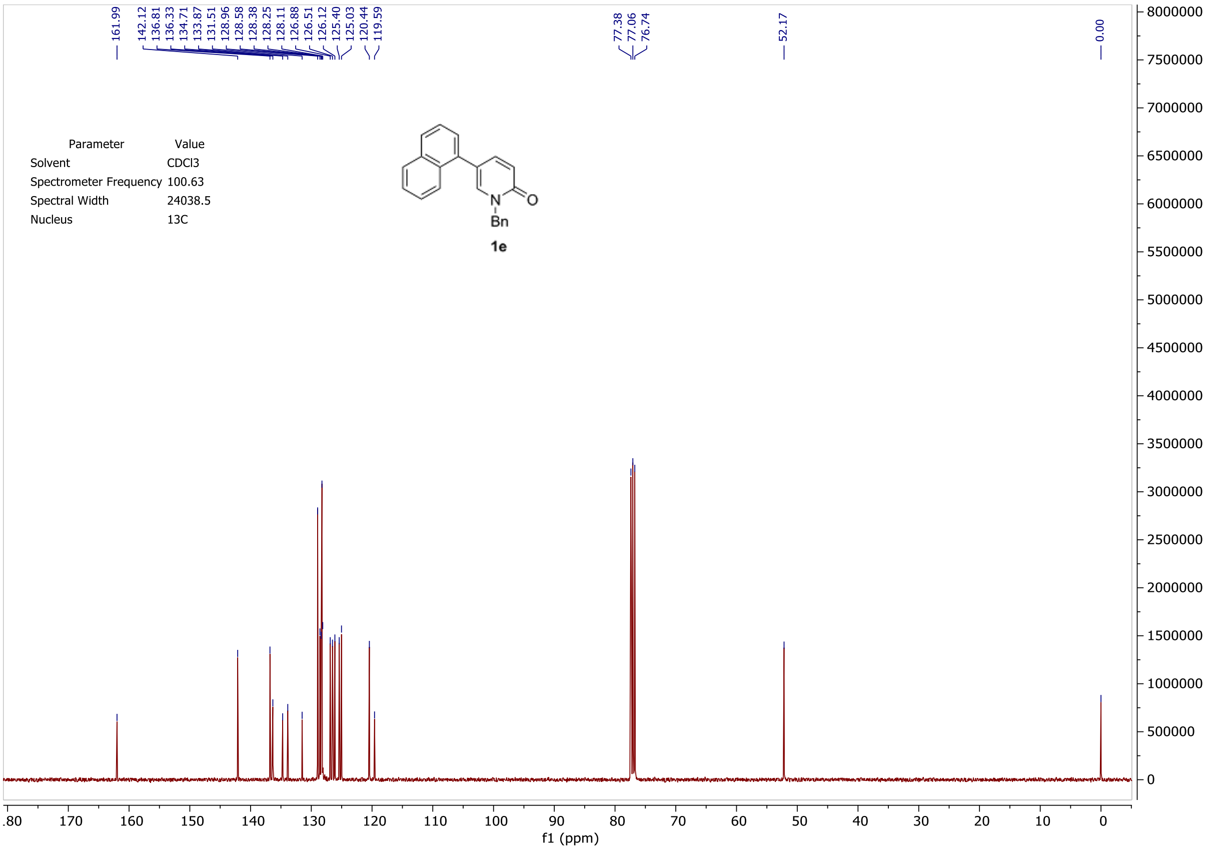
161.99
142.12
136.81
136.33
134.71
133.87
131.51
128.96
128.58
128.38
128.25
128.11
126.88
126.51
126.12
125.40
125.03
120.44
119.59
77.38
77.06
76.74
52.17
0.00
Parameter Value
Solvent CDCl3
Spectrometer Frequency 100.63
Spectral Width 24038.5
Nucleus 13C
N
O
Bn
1e
f1 (ppm)

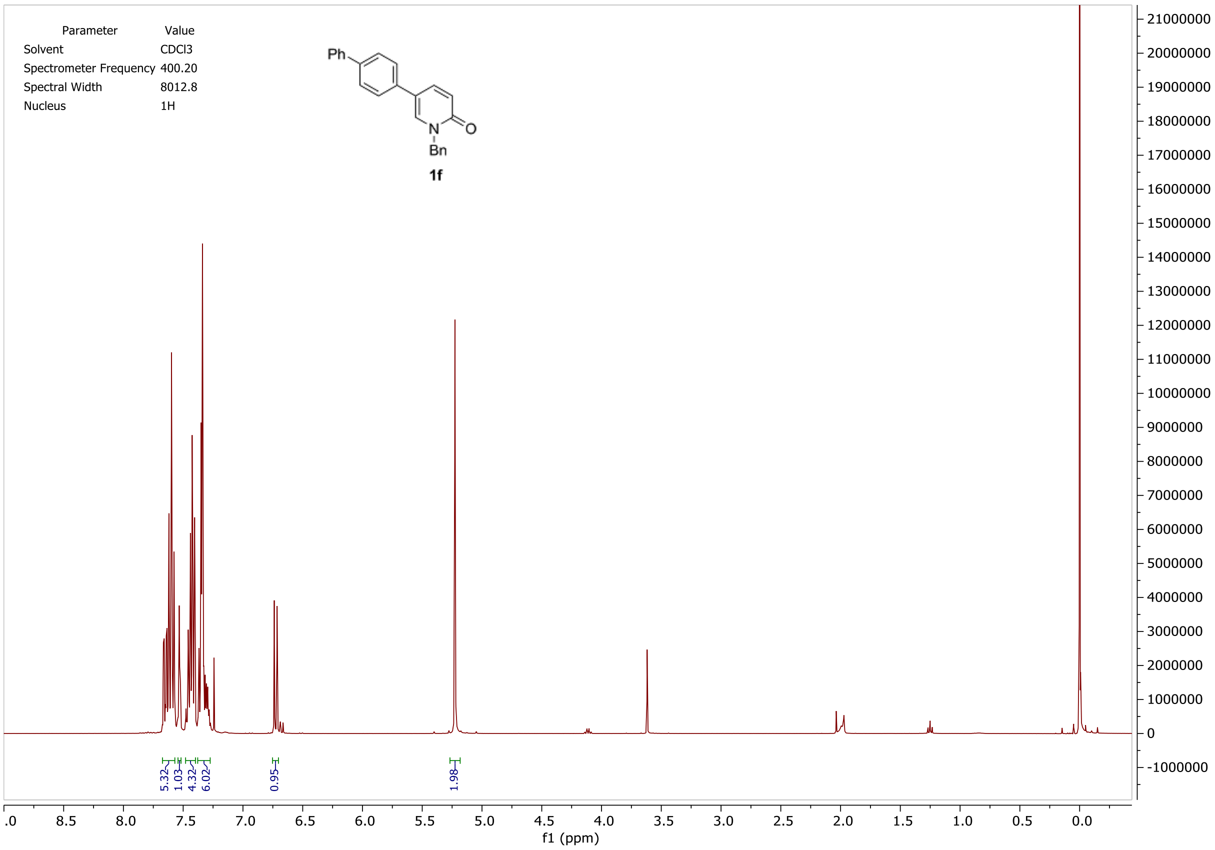
Parameter Value
Solvent CDCl3
Spectrometer Frequency 400.20
Spectral Width 8012.8
Nucleus 1H
Ph
N
O
Bn
1f
5.32
1.03
4.32
6.02
0.95
1.98
f1 (ppm)

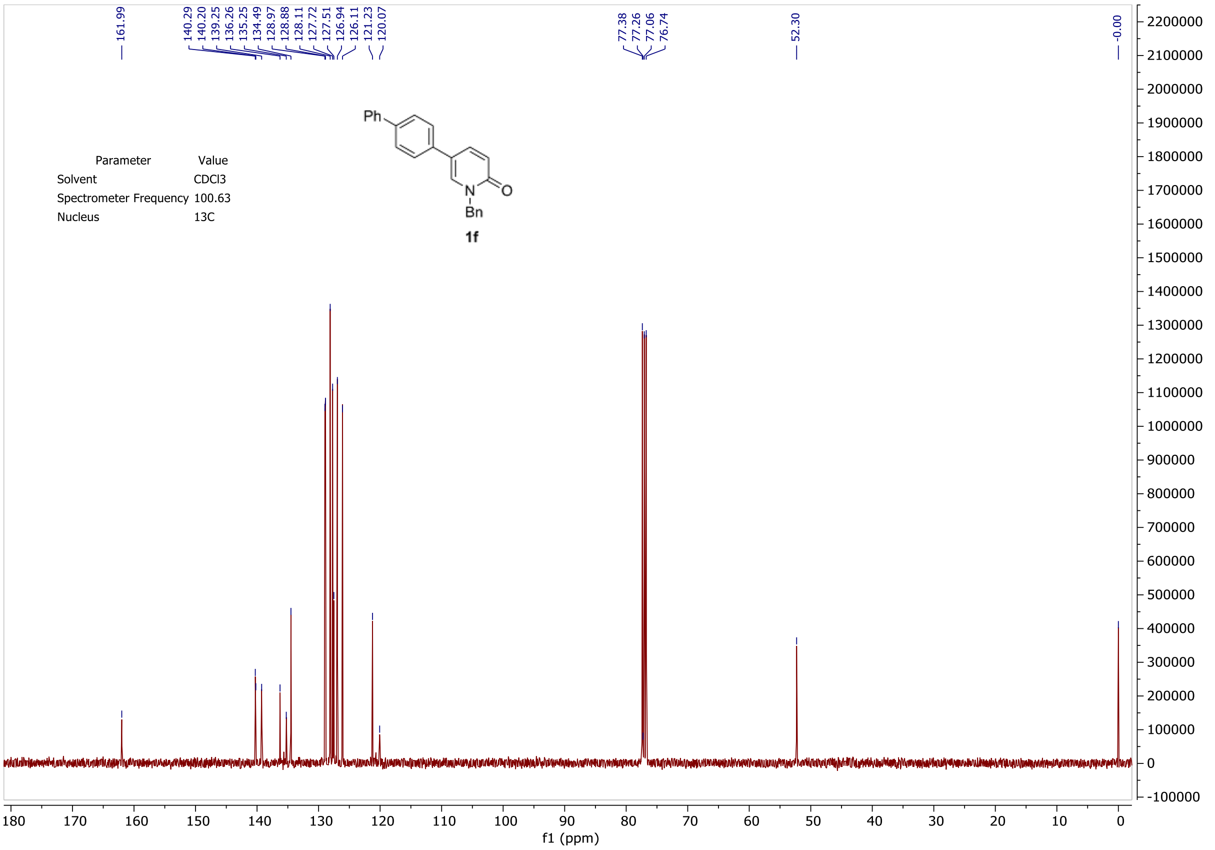
161.99
140.29
140.20
139.25
136.26
135.25
134.49
128.97
128.88
128.11
127.72
127.51
126.94
126.11
121.23
120.07
77.38
77.26
77.06
76.74
52.30
-0.00
Parameter Value
Solvent CDCl3
Spectrometer Frequency 100.63
Nucleus 13C
Ph
N
O
Bn
1f
f1 (ppm)

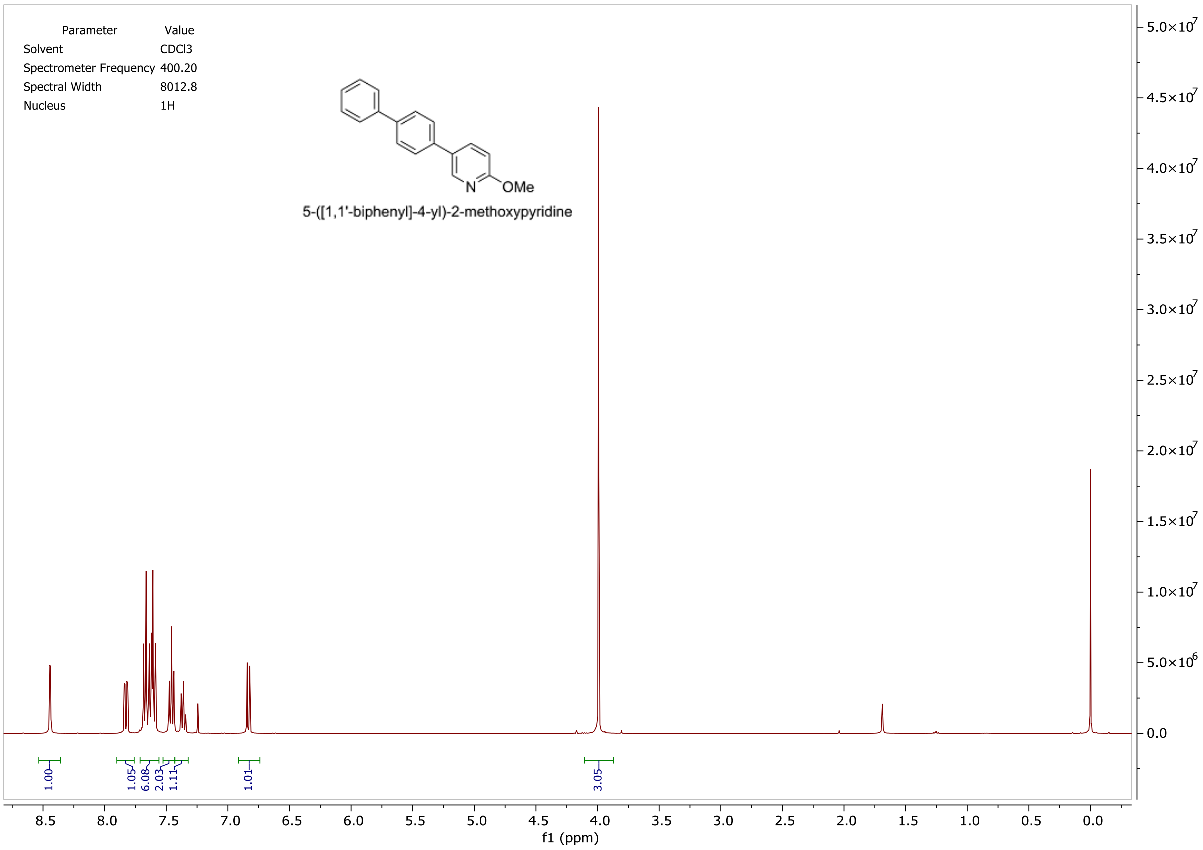
Parameter Value
Solvent CDCl3
Spectrometer Frequency 400.20
Spectral Width 8012.8
Nucleus 1H
N OMe
5-([1,1'-biphenyl]-4-yl)-2-methoxypyridine
1.00
1.05
6.08
2.03
1.11
1.01
3.05
f1 (ppm)

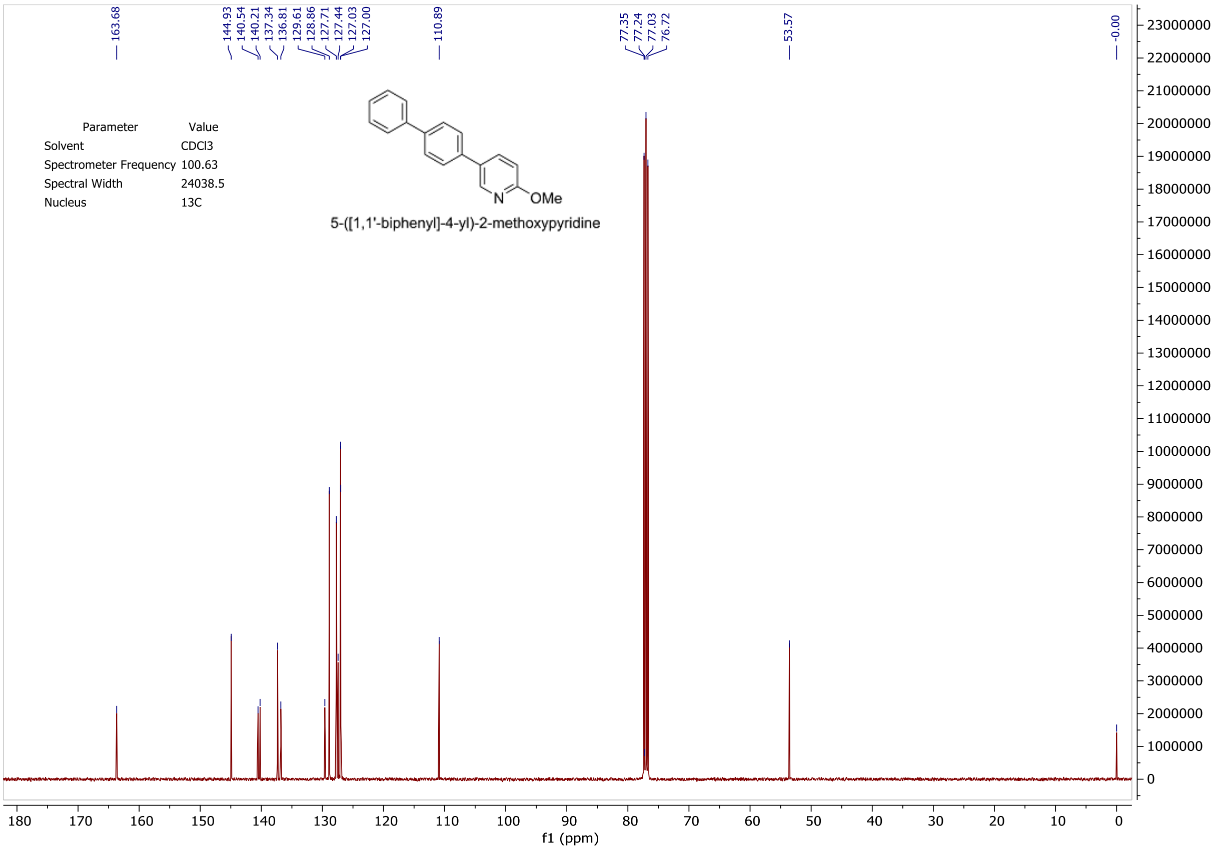
163.68
144.93
140.54
140.21
137.34
136.81
129.61
128.86
127.71
127.44
127.03
127.00
110.89
77.35
77.24
77.03
76.72
53.57
-0.00
Parameter Value
Solvent CDCl3
Spectrometer Frequency 100.63
Spectral Width 24038.5
Nucleus 13C
N OMe
5-([1,1'-biphenyl]-4-yl)-2-methoxypyridine
f1 (ppm)

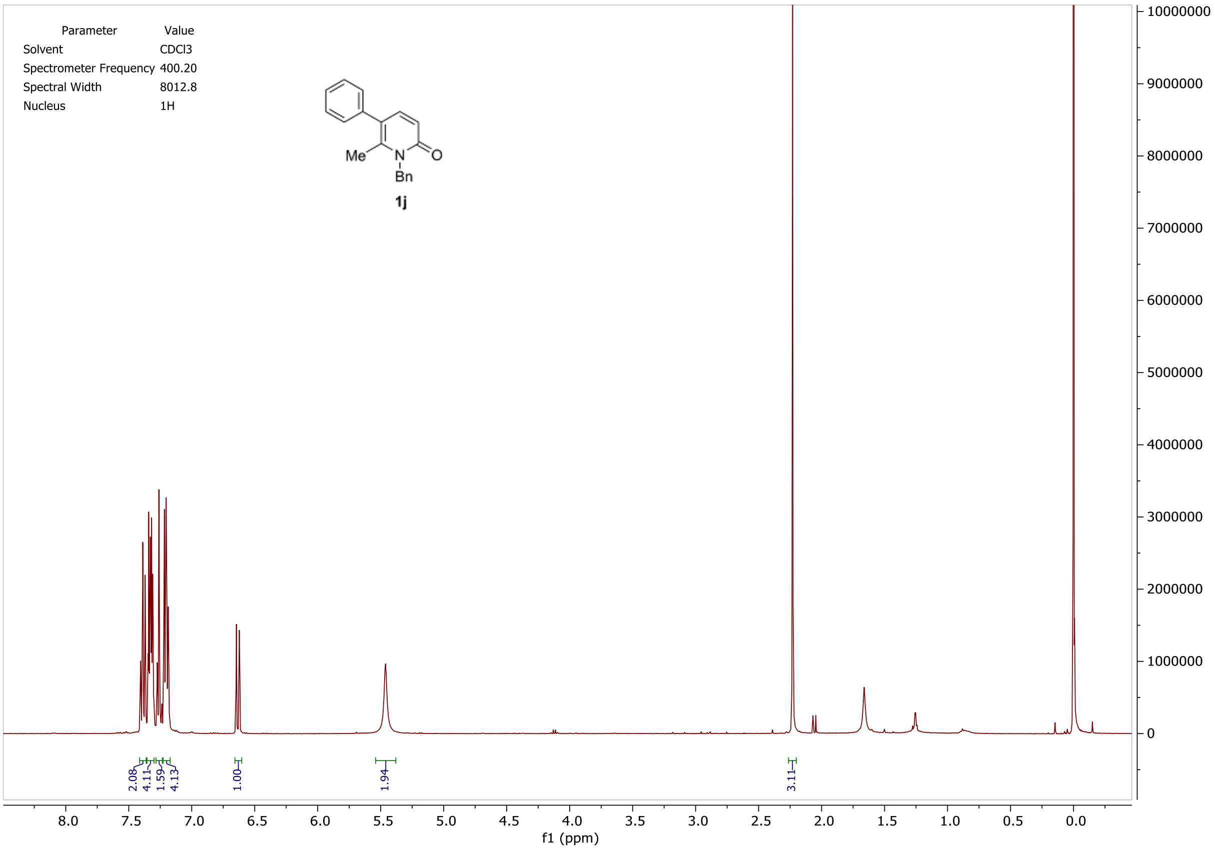

Parameter Value
Solvent CDCl3
Spectrometer Frequency 400.20
Spectral Width 8012.8
Nucleus 1H
Me
N
O
Bn
1j
2.08
4.11
1.59
4.13
1.00
1.94
3.11
f1 (ppm)

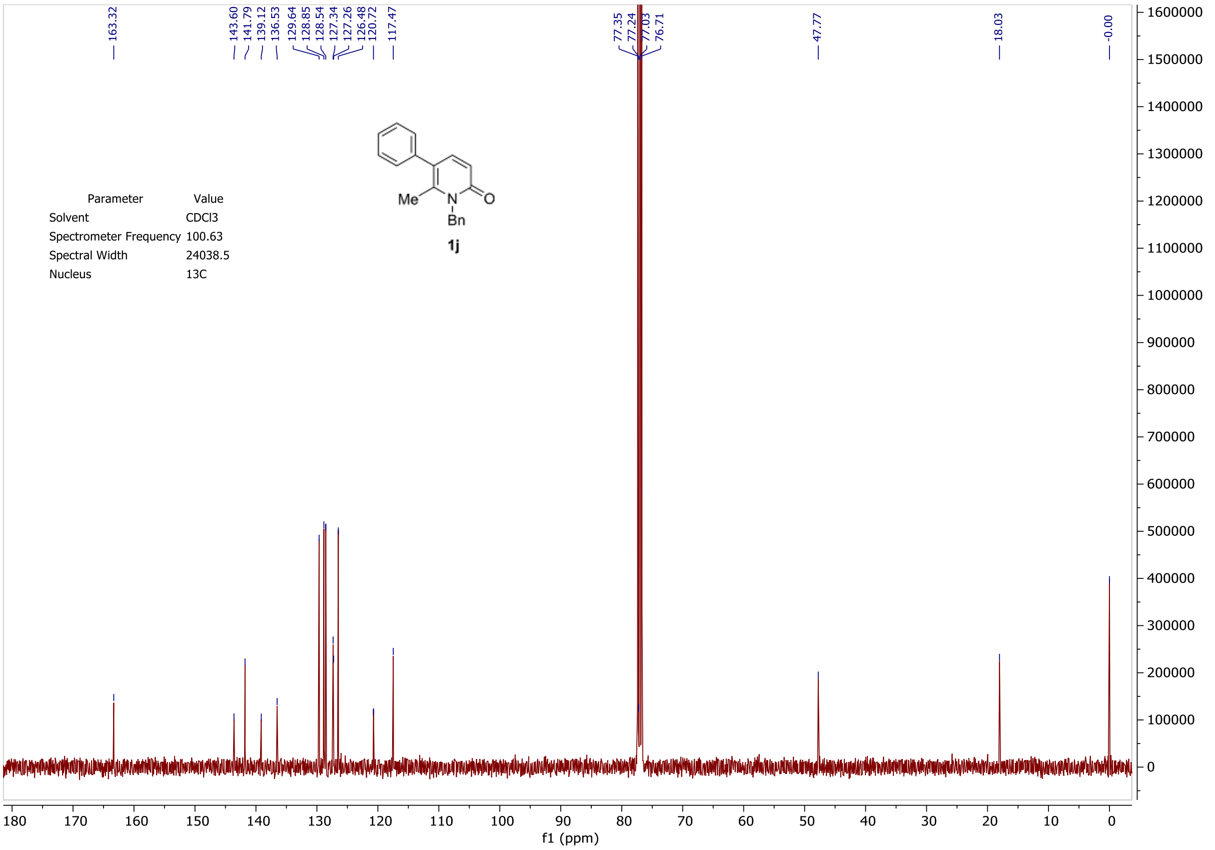

163.32
143.60
141.79
139.12
136.53
129.64
128.85
128.54
127.34
127.26
126.48
120.72
117.47
77.35
77.24
77.03
76.71
47.77
18.03
-0.00
Parameter Value
Solvent CDCl3
Spectrometer Frequency 100.63
Spectral Width 24038.5
Nucleus 13C
Me
N
O
Bn
1j
f1 (ppm)

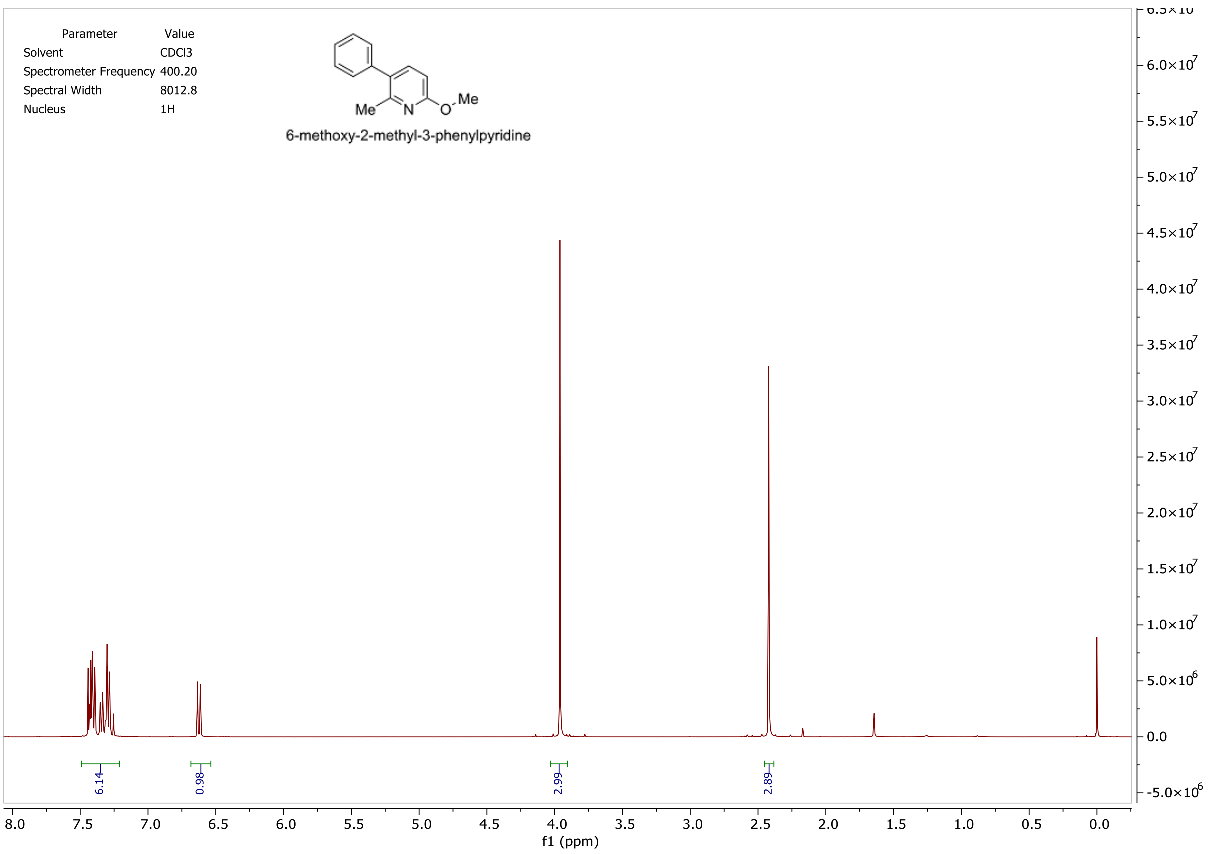
Parameter Value
Solvent CDCl3
Spectrometer Frequency 400.20
Spectral Width 8012.8
Nucleus 1H
6-methoxy-2-methyl-3-phenylpyridine
Me N O Me
6.14
0.98
2.99
2.89
f1 (ppm)

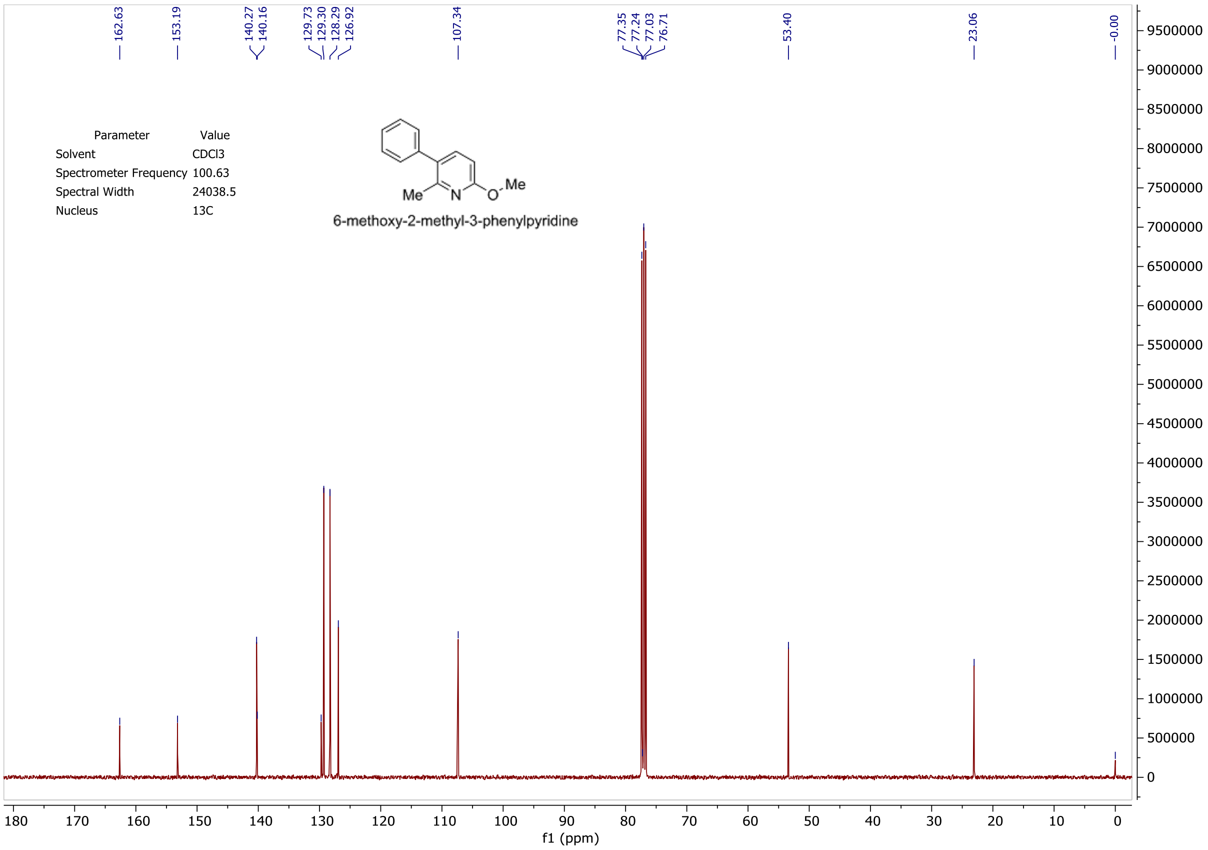
162.63
153.19
140.27
140.16
129.73
129.30
128.29
126.92
107.34
77.35
77.24
77.03
76.71
53.40
23.06
-0.00
Parameter Value
Solvent CDCl3
Spectrometer Frequency 100.63
Spectral Width 24038.5
Nucleus 13C
6-methoxy-2-methyl-3-phenylpyridine
Me N O Me
f1 (ppm)

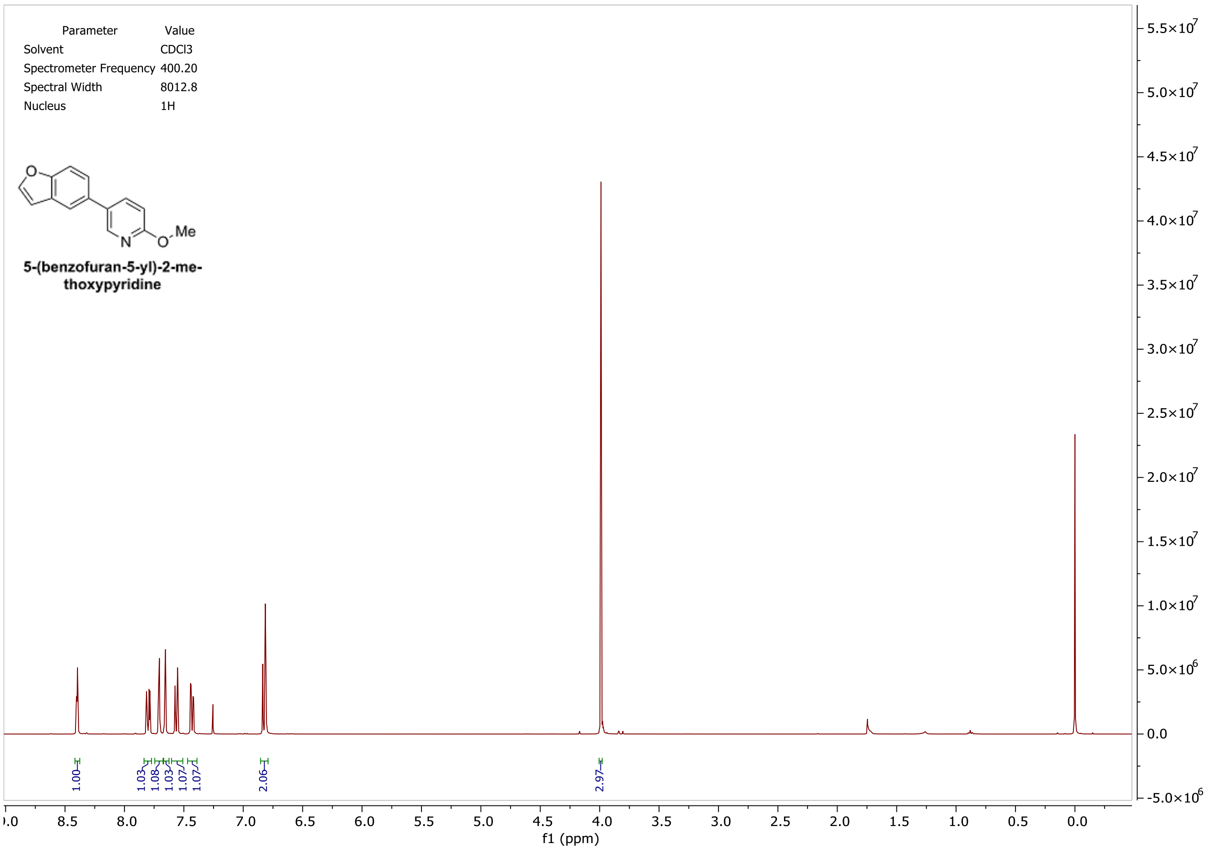

Parameter Value
Solvent CDCl3
Spectrometer Frequency 400.20
Spectral Width 8012.8
Nucleus 1H
5-(benzofuran-5-yl)-2-methoxypyridine
1.00
1.03
1.08
1.03
1.07
1.07
2.06
2.97
f1 (ppm)

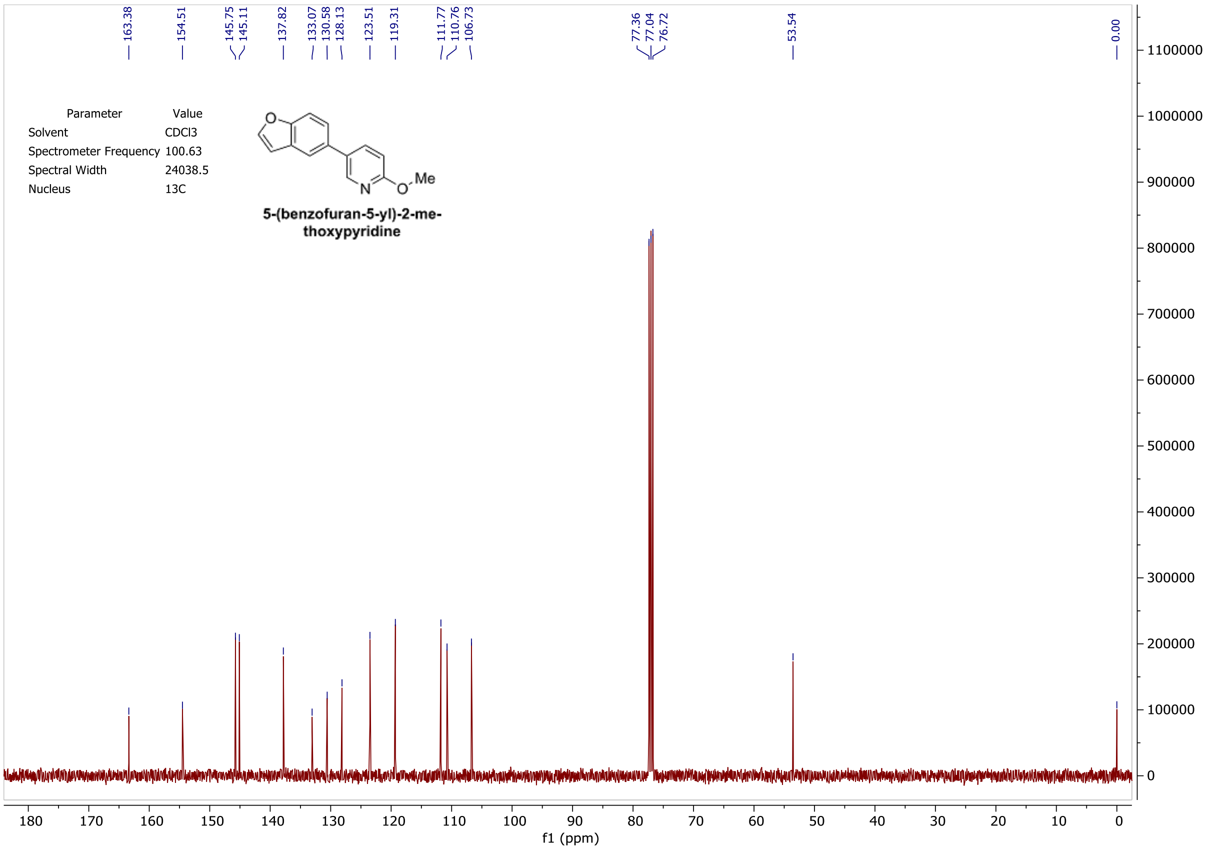

163.38
154.51
145.75
145.11
137.82
133.07
130.58
128.13
123.51
119.31
111.77
110.76
106.73
77.36
77.04
76.72
53.54
0.00
Parameter Value
Solvent CDCl3
Spectrometer Frequency 100.63
Spectral Width 24038.5
Nucleus 13C
5-(benzofuran-5-yl)-2-methoxypyridine
f1 (ppm)

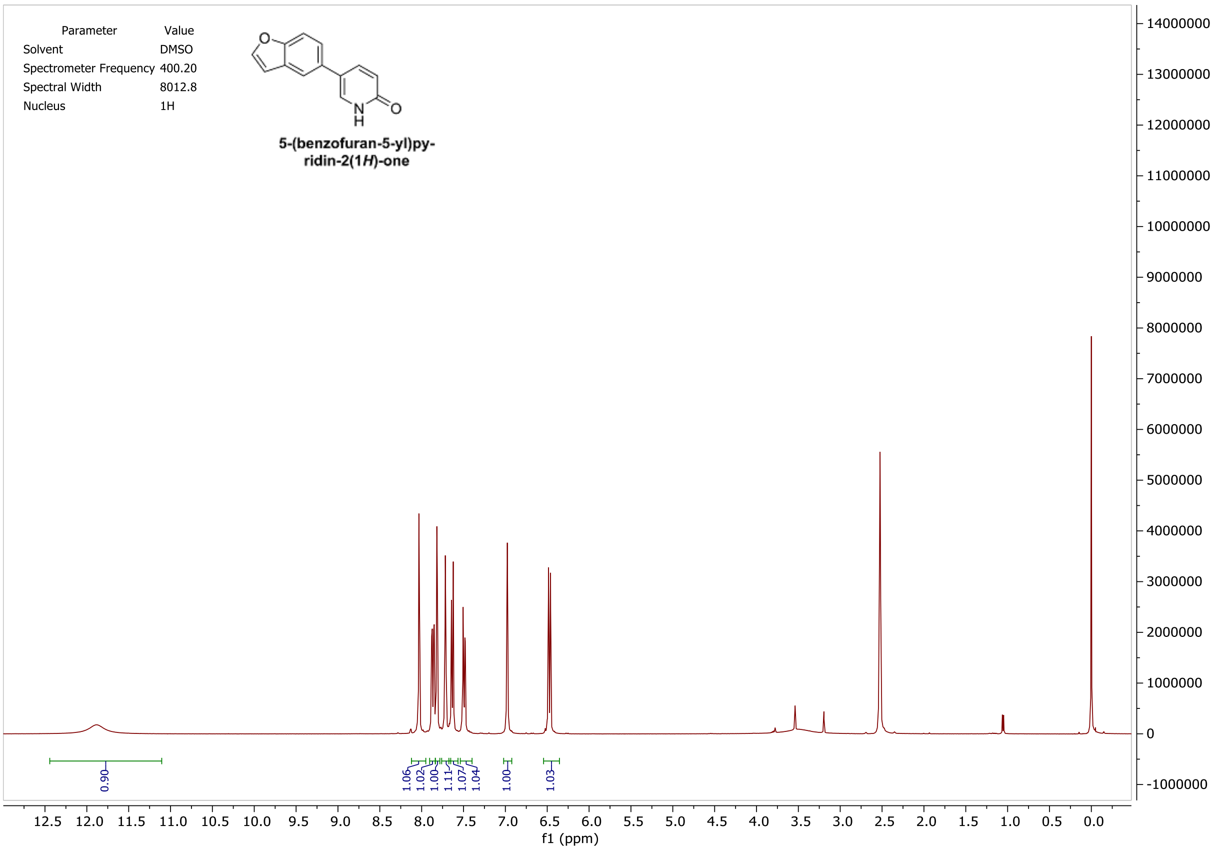

Parameter Value
Solvent DMSO
Spectrometer Frequency 400.20
Spectral Width 8012.8
Nucleus 1H
5-(benzofuran-5-yl)pyridin-2(1*H*)-one
0.90
1.06
1.02
1.00
1.11
1.07
1.04
1.00
1.03
f1 (ppm)

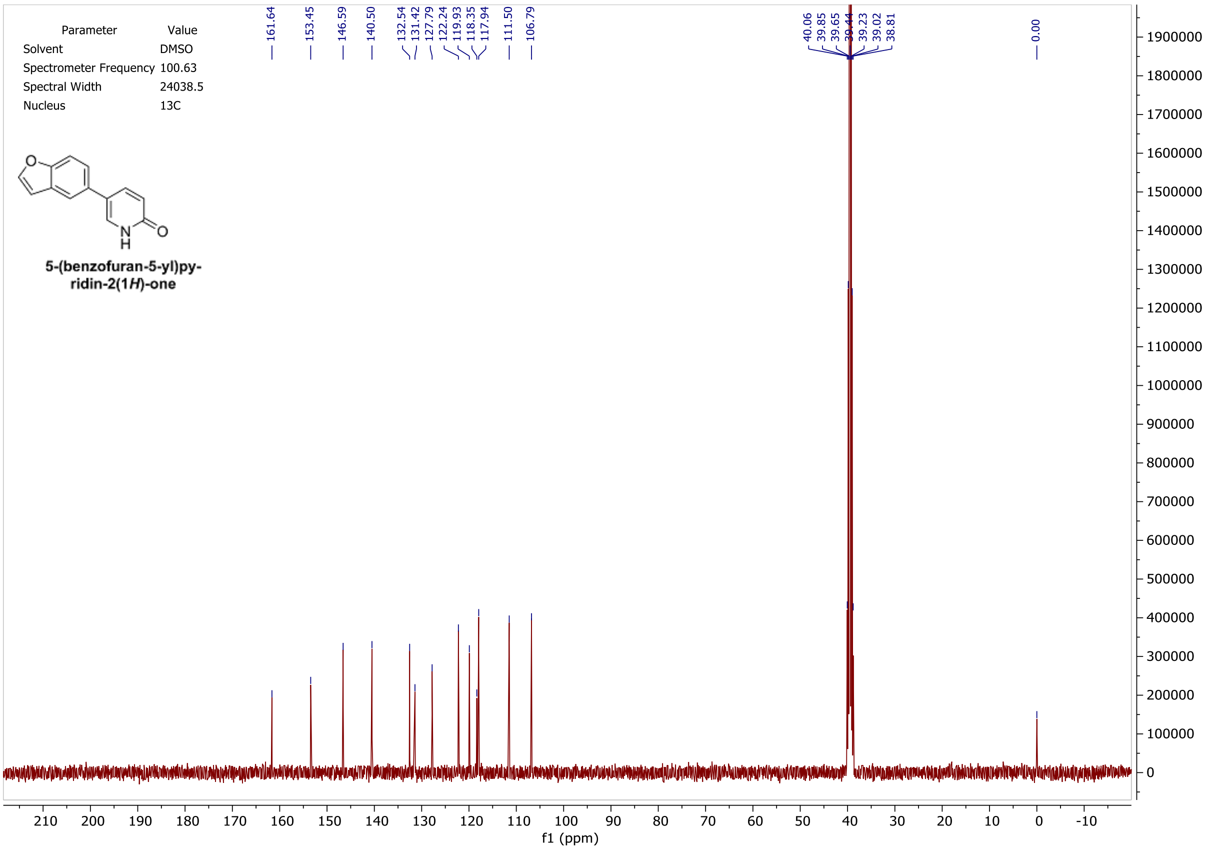

Parameter Value
Solvent DMSO
Spectrometer Frequency 100.63
Spectral Width 24038.5
Nucleus 13C
5-(benzofuran-5-yl)pyridin-2(1*H*)-one
161.64
153.45
146.59
140.50
132.54
131.42
127.79
122.24
119.93
118.35
117.94
111.50
106.79
40.06
39.85
39.65
39.44
39.23
39.02
38.81
0.00
f1 (ppm)

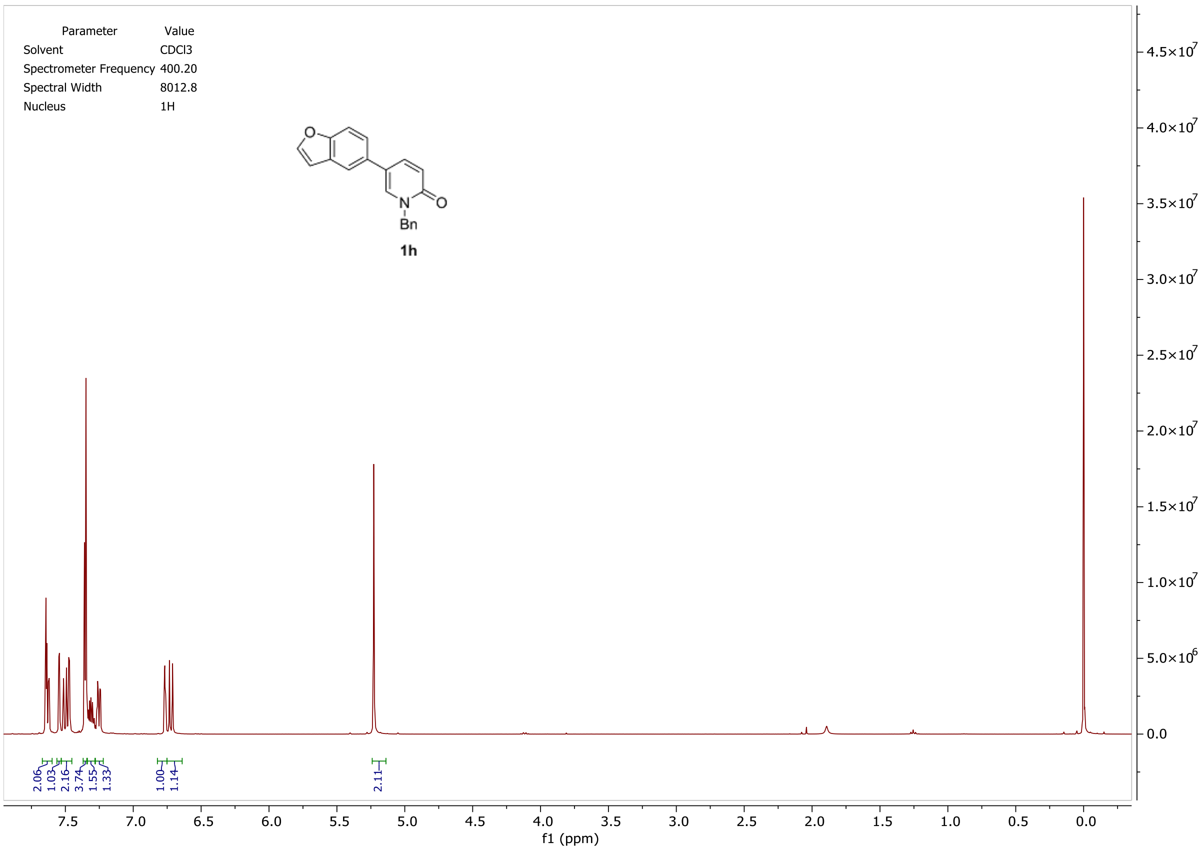
Parameter Value
Solvent CDCl3
Spectrometer Frequency 400.20
Spectral Width 8012.8
Nucleus 1H
N
O
Bn
1h
2.06
1.03
2.16
3.74
1.55
1.33
1.00
1.14
2.11
f1 (ppm)

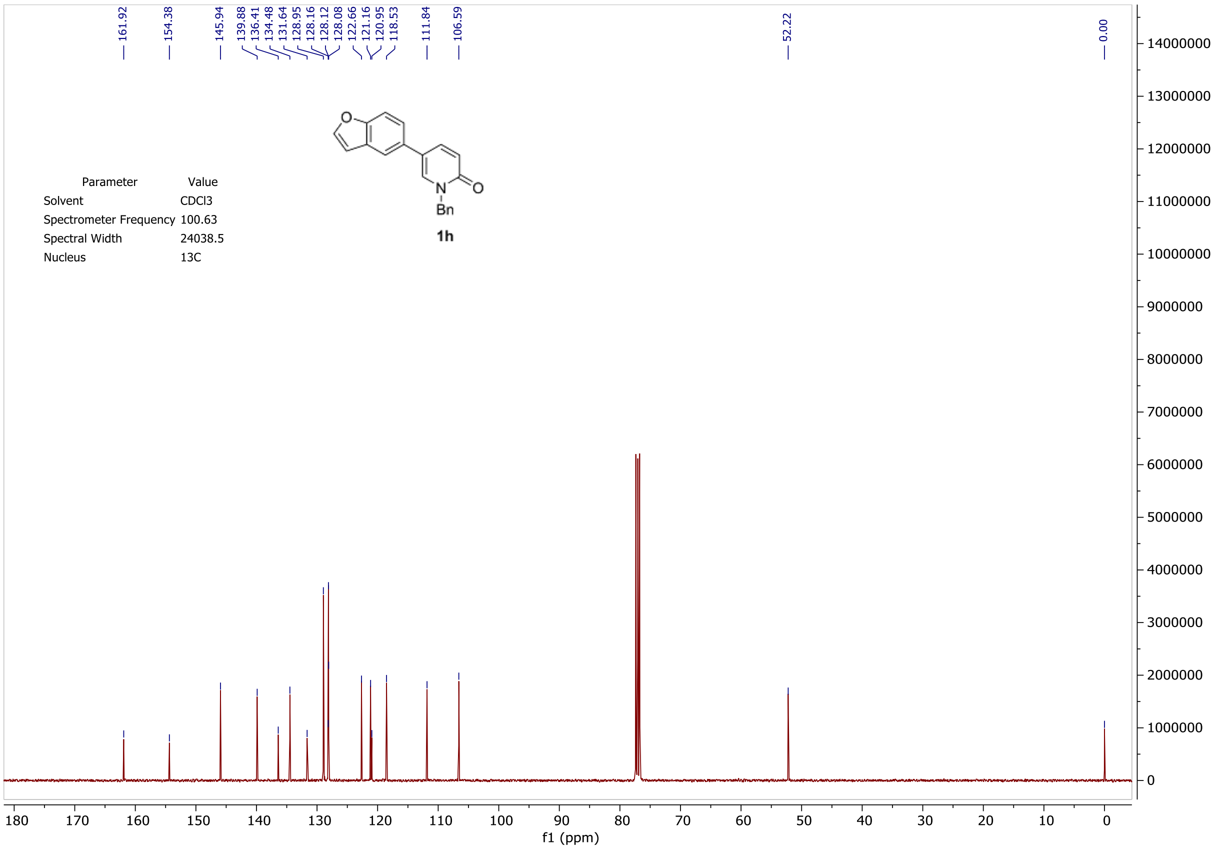
161.92
154.38
145.94
139.88
136.41
134.48
131.64
128.95
128.16
128.12
128.08
122.66
121.16
120.95
118.53
111.84
106.59
52.22
0.00
Parameter Value
Solvent CDCl3
Spectrometer Frequency 100.63
Spectral Width 24038.5
Nucleus 13C
N
O
Bn
1h
f1 (ppm)

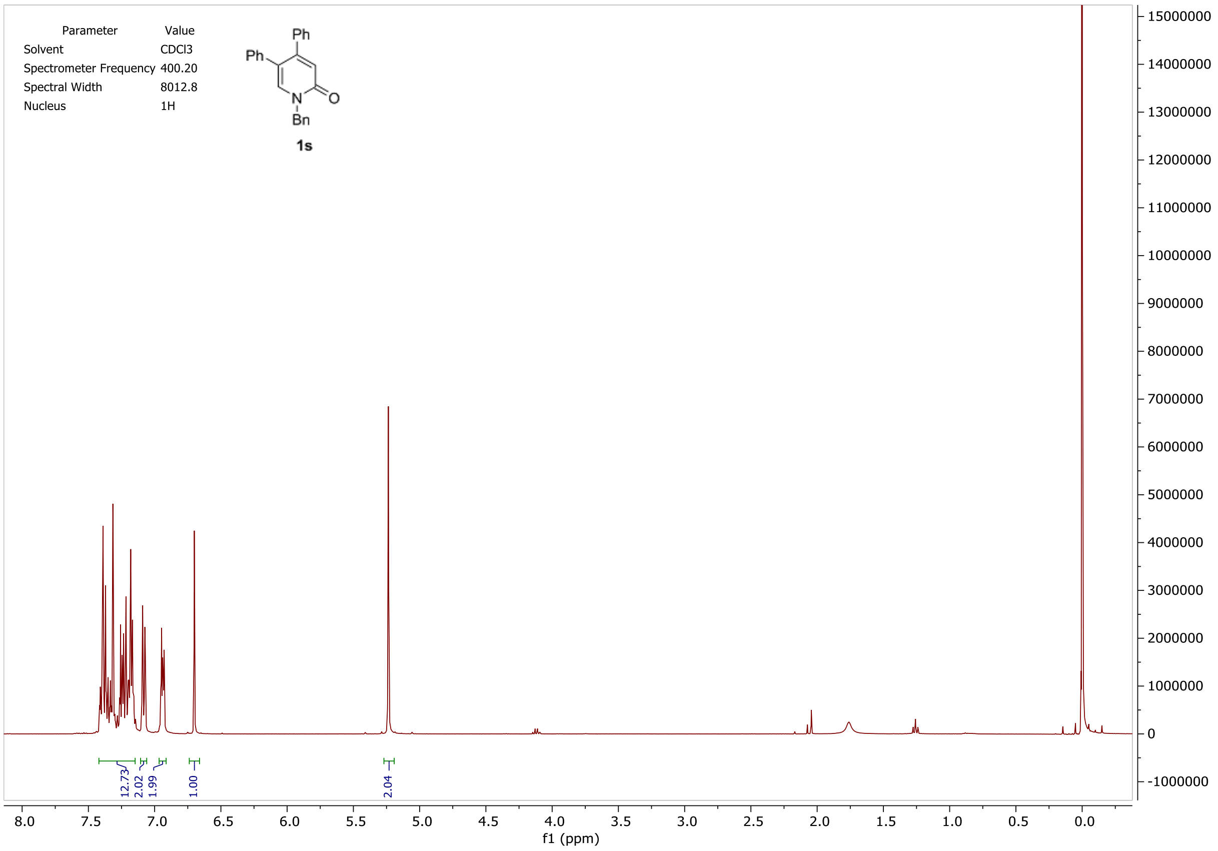

Parameter Value
Solvent CDCl3
Spectrometer Frequency 400.20
Spectral Width 8012.8
Nucleus 1H
Ph
Ph
N
O
Bn
1s
12.73
2.02
1.99
1.00
2.04
f1 (ppm)

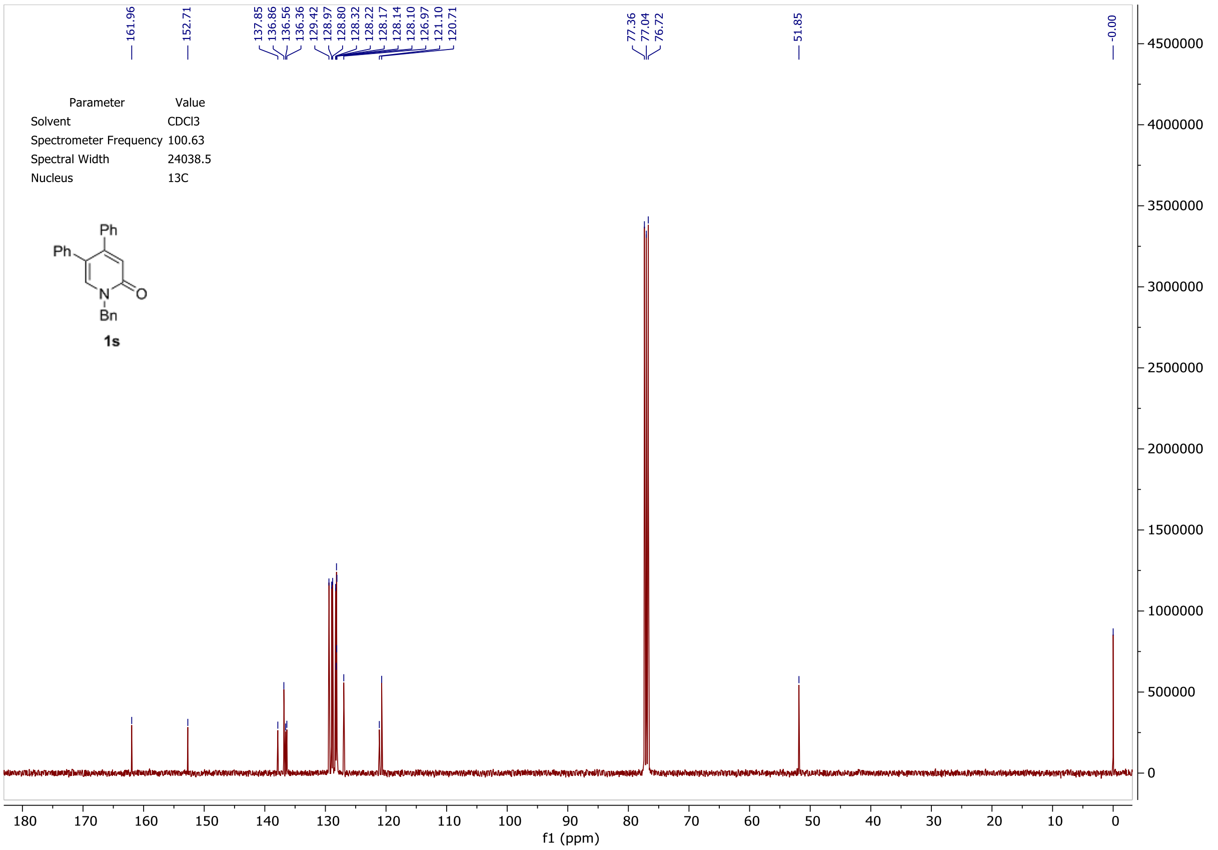

161.96
152.71
137.85
136.86
136.56
136.36
129.42
128.97
128.80
128.32
128.22
128.17
128.14
128.10
126.97
121.10
120.71
77.36
77.04
76.72
51.85
-0.00
Parameter Value
Solvent CDCl3
Spectrometer Frequency 100.63
Spectral Width 24038.5
Nucleus 13C
Ph
Ph
N
O
Bn
1s
f1 (ppm)

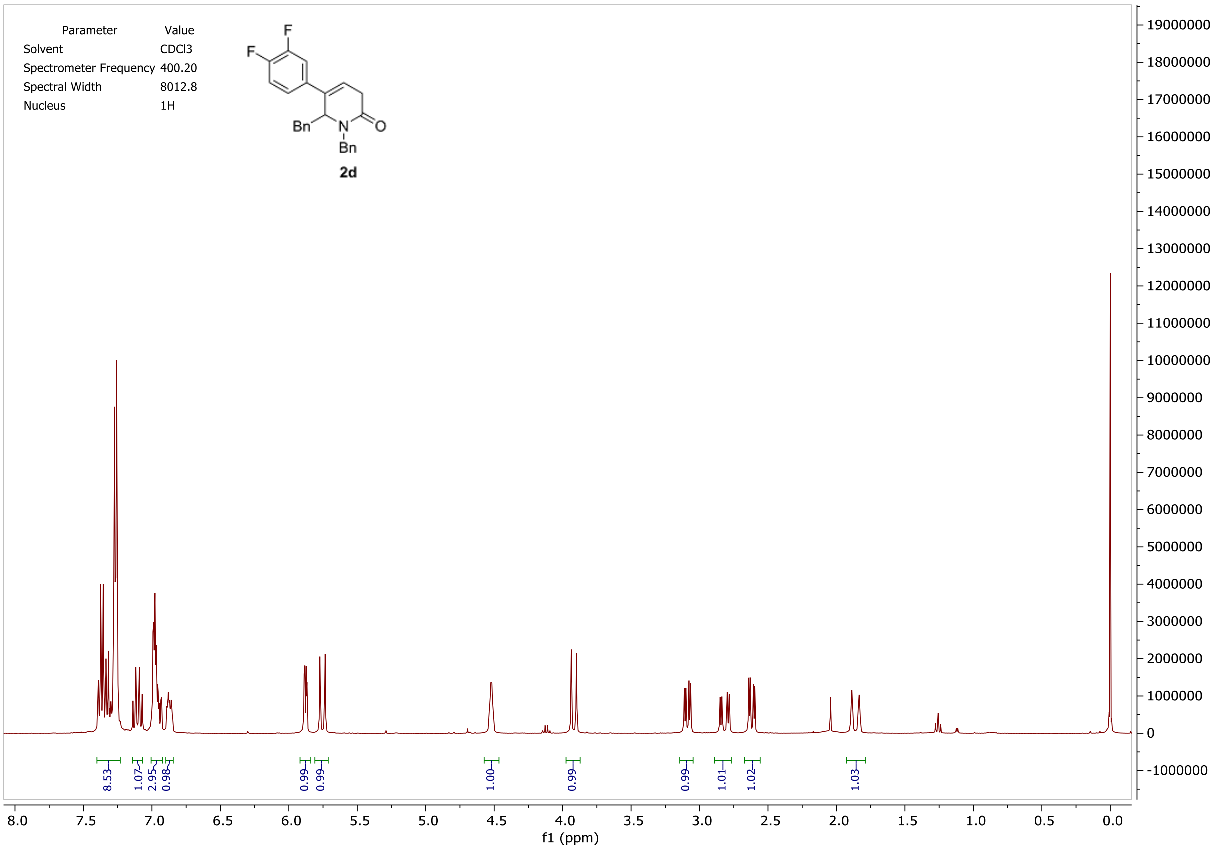
Parameter Value
Solvent CDCl3
Spectrometer Frequency 400.20
Spectral Width 8012.8
Nucleus 1H
F
F
Bn
N
O
Bn
2d
8.53
1.07
2.95
0.98
0.99
0.99
1.00
0.99
0.99
1.01
1.02
1.03
f1 (ppm)

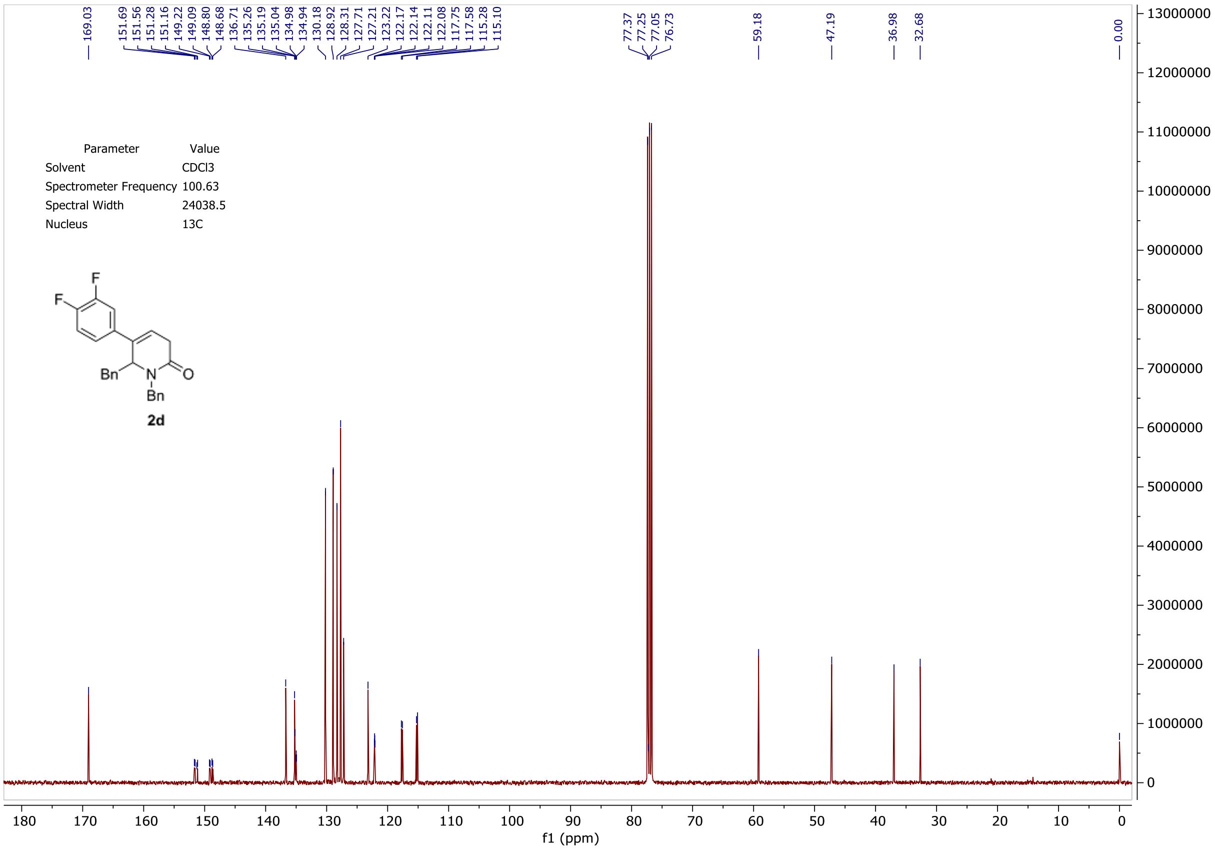
169.03
151.69
151.56
151.28
151.16
149.22
149.09
148.80
148.68
136.71
135.26
135.19
135.04
134.98
134.94
130.18
128.92
128.31
127.71
127.21
123.22
122.17
122.14
122.11
122.08
117.75
117.58
115.28
115.10
77.37
77.25
77.05
76.73
59.18
47.19
36.98
32.68
0.00
Parameter Value
Solvent CDCl3
Spectrometer Frequency 100.63
Spectral Width 24038.5
Nucleus 13C
F
F
Bn
N
O
Bn
2d
f1 (ppm)

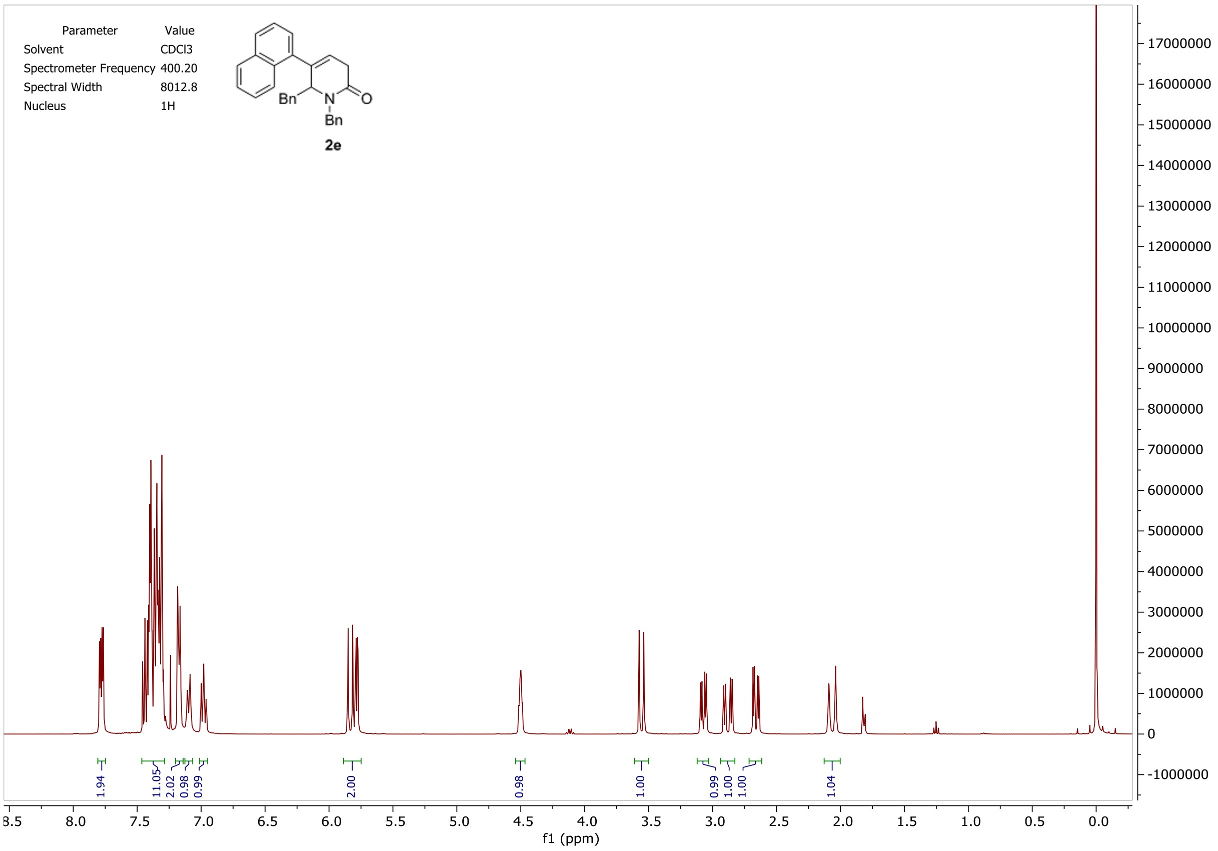
Parameter Value
Solvent CDCl3
Spectrometer Frequency 400.20
Spectral Width 8012.8
Nucleus 1H
Bn
N
Bn
O
2e
1.94
11.05
2.02
0.98
0.99
2.00
0.98
1.00
0.99
1.00
1.00
1.04
f1 (ppm)

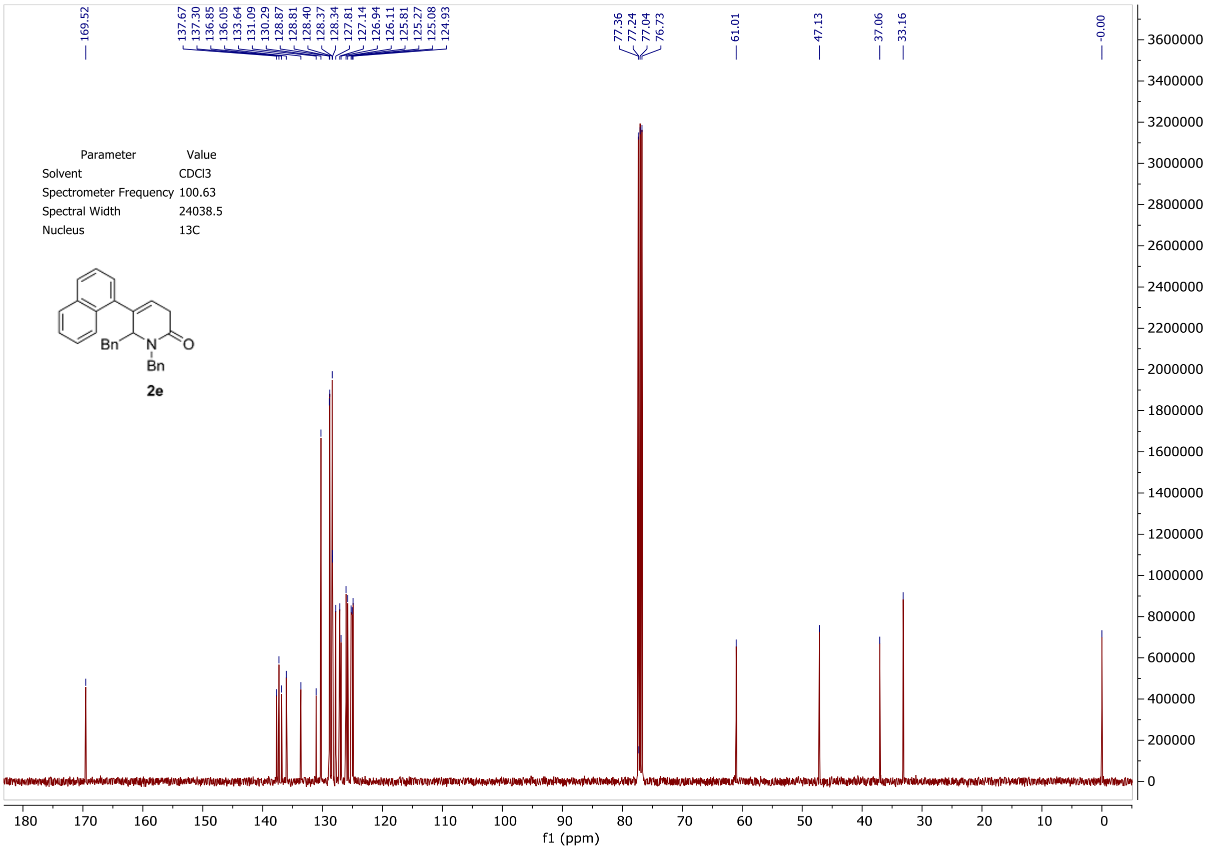
169.52
137.67
137.30
136.85
136.05
133.64
131.09
130.29
128.87
128.81
128.40
128.37
128.34
127.81
127.14
126.94
126.11
125.81
125.27
125.08
124.93
77.36
77.24
77.04
76.73
61.01
47.13
37.06
33.16
-0.00
Parameter Value
Solvent CDCl3
Spectrometer Frequency 100.63
Spectral Width 24038.5
Nucleus 13C
Bn
N
Bn
O
2e
f1 (ppm)

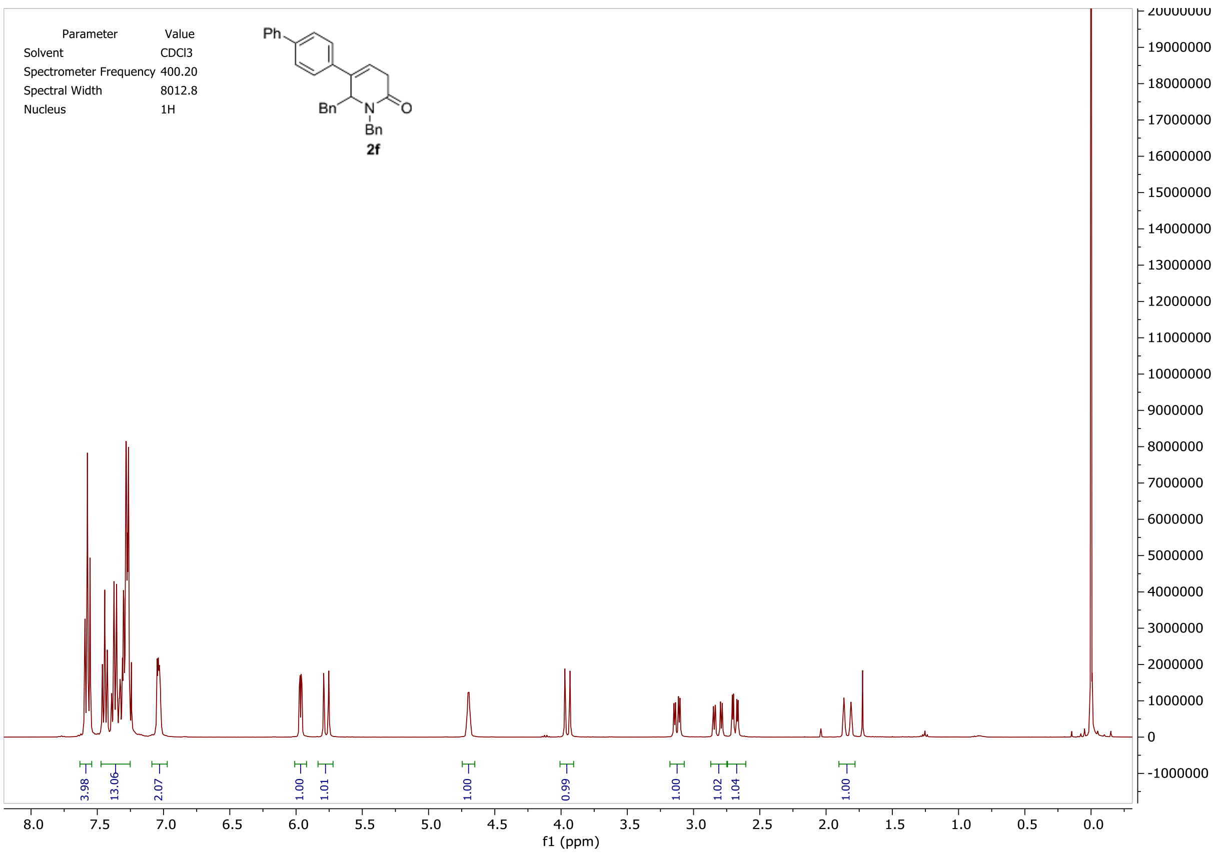
Parameter Value
Solvent CDCl3
Spectrometer Frequency 400.20
Spectral Width 8012.8
Nucleus 1H
Ph
Bn
N
O
Bn
2f
3.98
13.06
2.07
1.00
1.01
1.00
0.99
1.00
1.02
1.04
1.00
f1 (ppm)

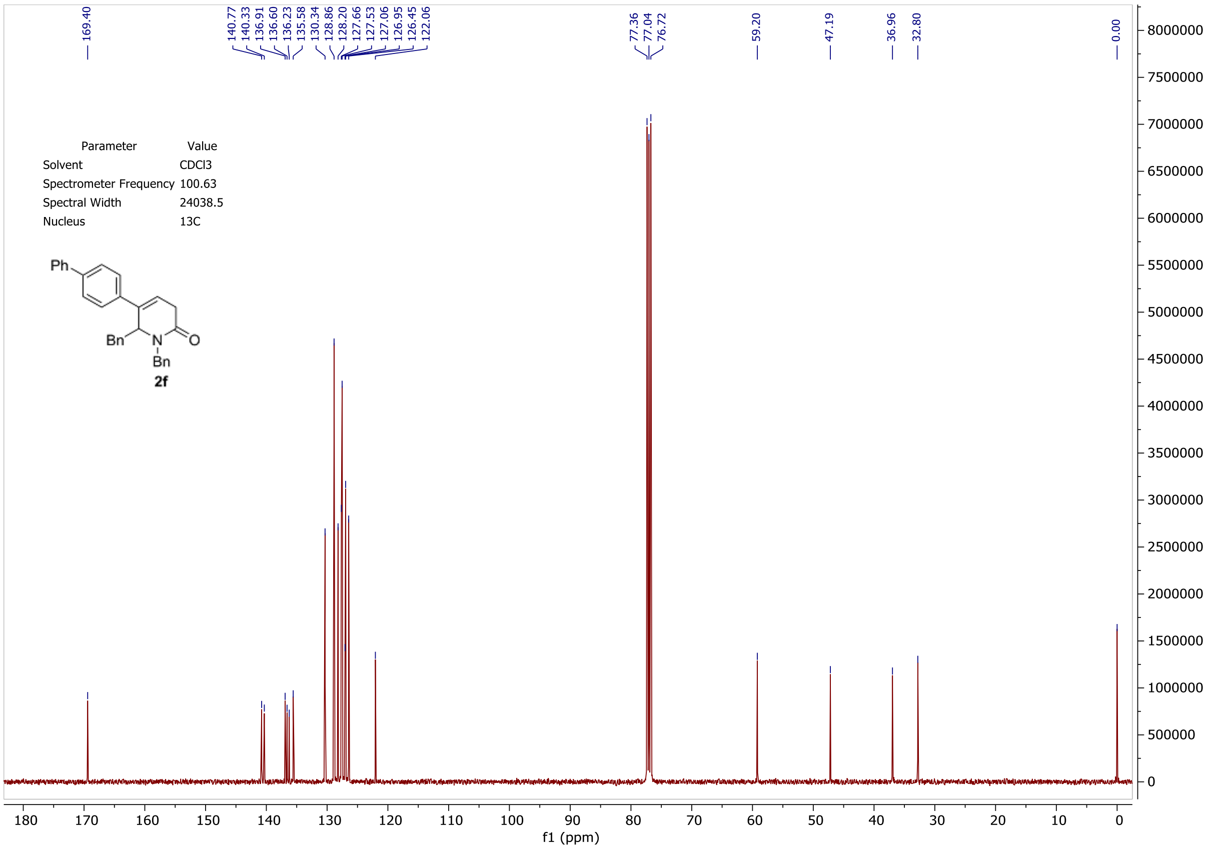
169.40
140.77
140.33
136.91
136.60
136.23
135.58
130.34
128.86
128.20
127.66
127.53
127.06
126.95
126.45
122.06
77.36
77.04
76.72
59.20
47.19
36.96
32.80
0.00
Parameter Value
Solvent CDCl3
Spectrometer Frequency 100.63
Spectral Width 24038.5
Nucleus 13C
Ph
Bn
N
O
Bn
2f
f1 (ppm)

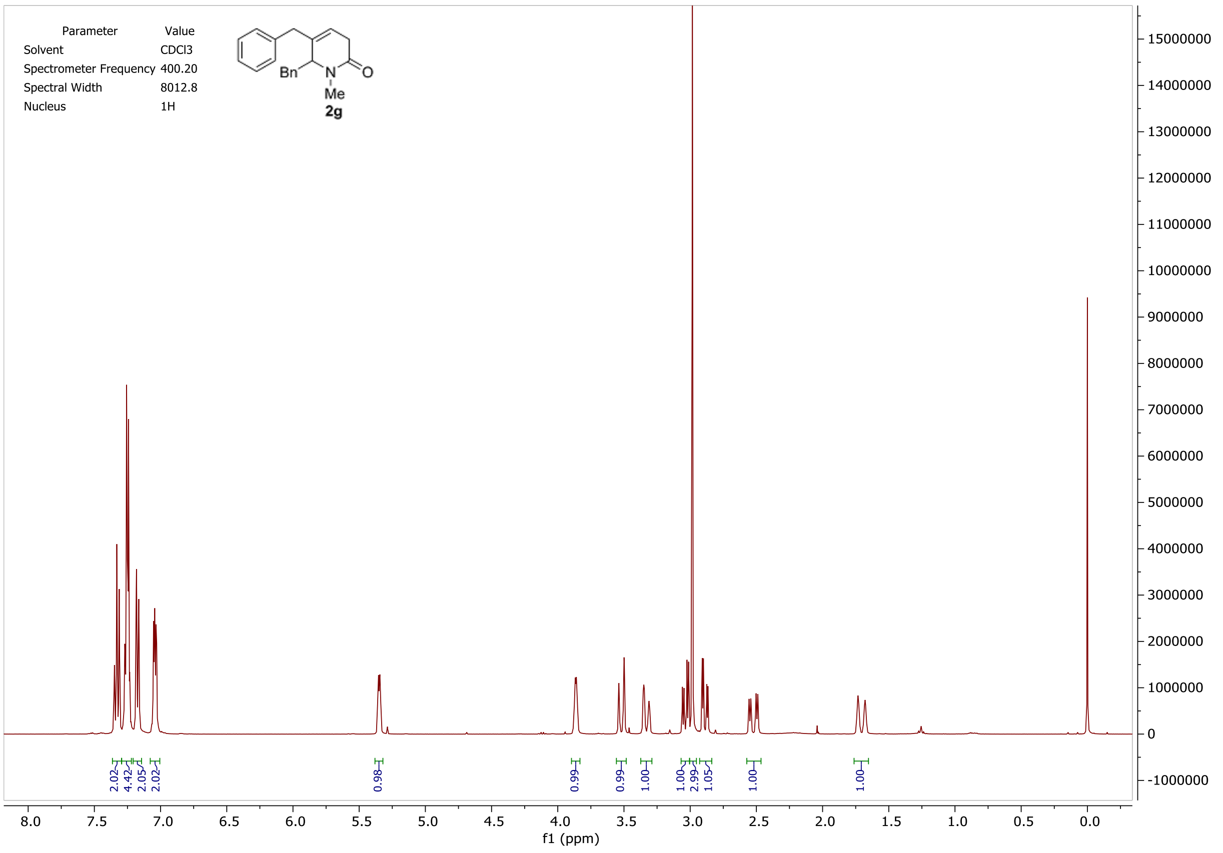

Parameter Value
Solvent CDCl3
Spectrometer Frequency 400.20
Spectral Width 8012.8
Nucleus 1H
Bn
N
O
Me
2g
2.02
4.42
2.05
2.02
0.98
0.99
0.99
1.00
1.00
2.99
1.05
1.00
1.00
f1 (ppm)

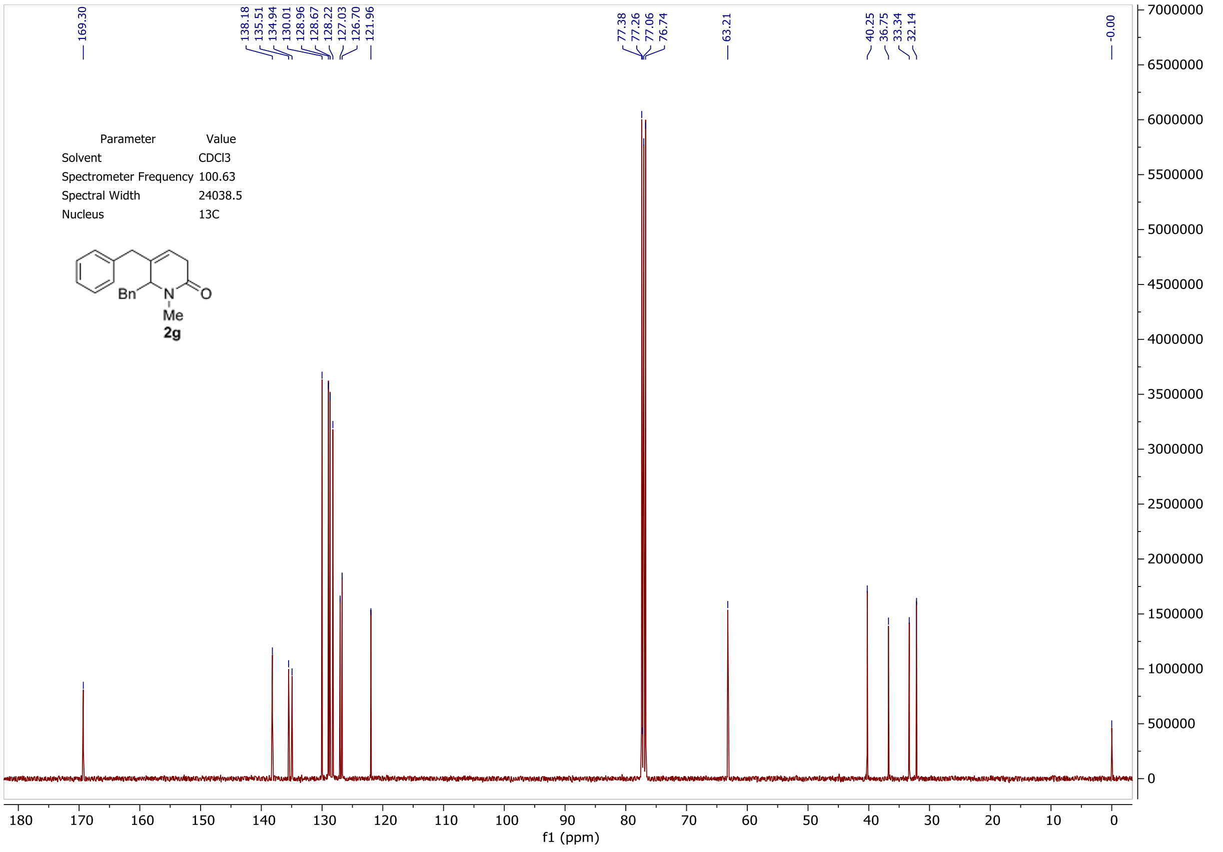

169.30
138.18
135.51
134.94
130.01
128.96
128.67
128.22
127.03
126.70
121.96
77.38
77.26
77.06
76.74
63.21
40.25
36.75
33.34
32.14
-0.00
Parameter Value
Solvent CDCl3
Spectrometer Frequency 100.63
Spectral Width 24038.5
Nucleus 13C
Bn
N
O
Me
2g
f1 (ppm)

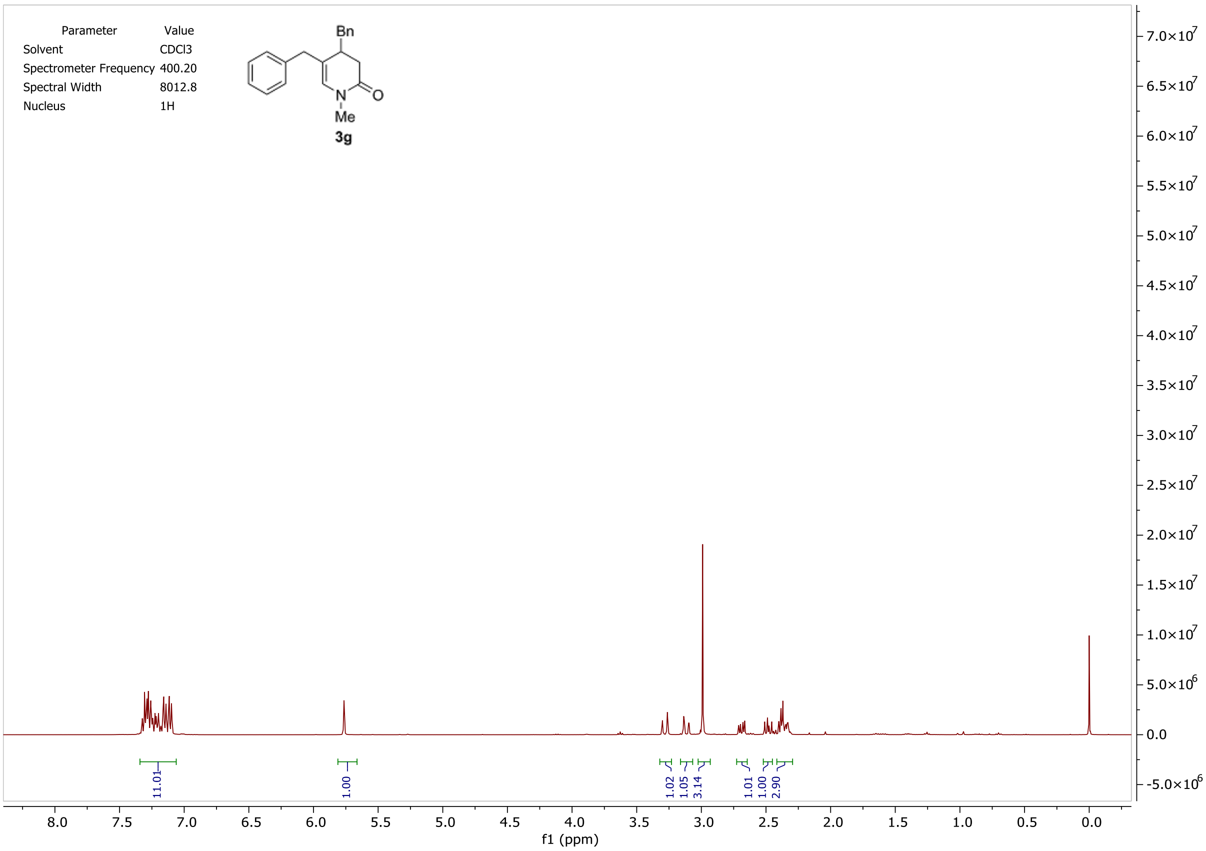
Parameter Value
Solvent CDCl3
Spectrometer Frequency 400.20
Spectral Width 8012.8
Nucleus 1H
Bn
N
O
Me
3g
11.01
1.00
1.02
1.05
3.14
1.01
1.00
2.90
f1 (ppm)

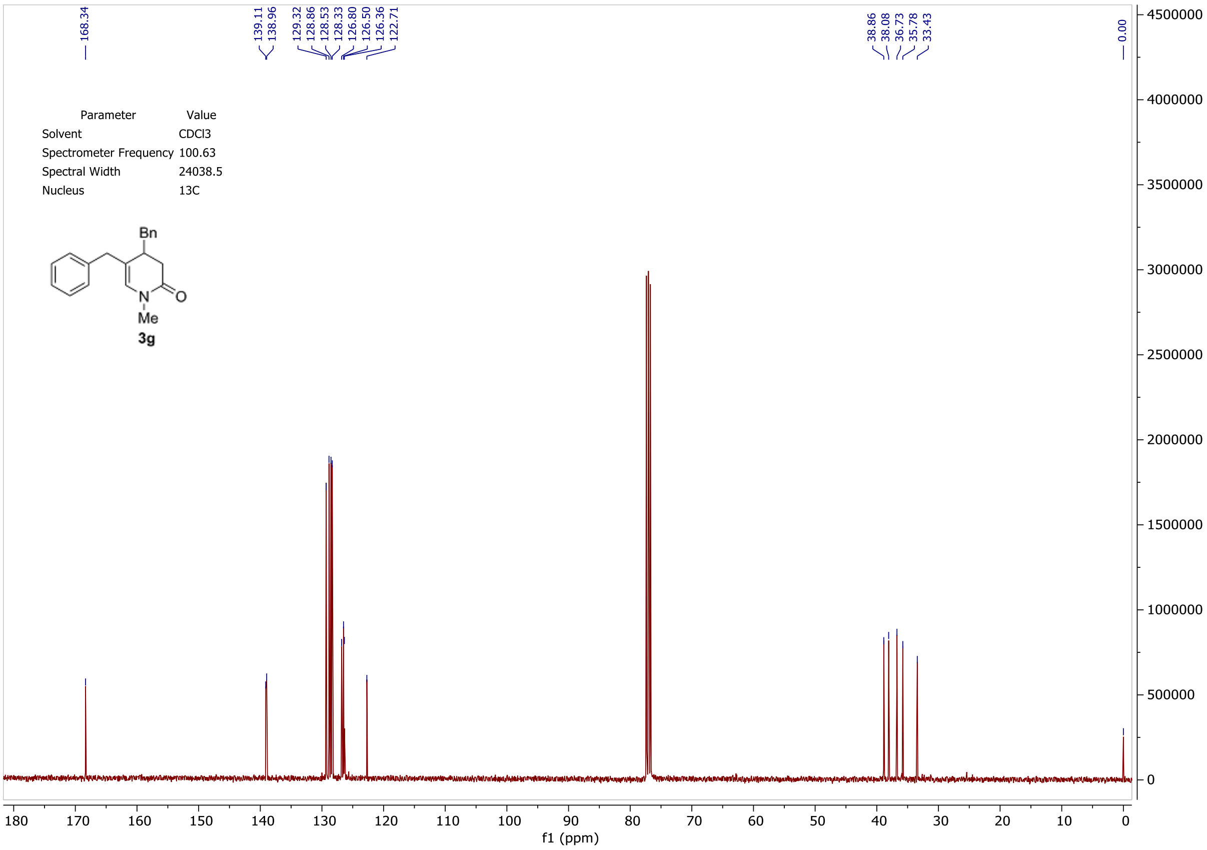
168.34
139.11
138.96
129.32
128.86
128.53
128.33
126.80
126.50
126.36
122.71
38.86
38.08
36.73
35.78
33.43
0.00
Parameter Value
Solvent CDCl3
Spectrometer Frequency 100.63
Spectral Width 24038.5
Nucleus 13C
Bn
N
O
Me
3g
f1 (ppm)

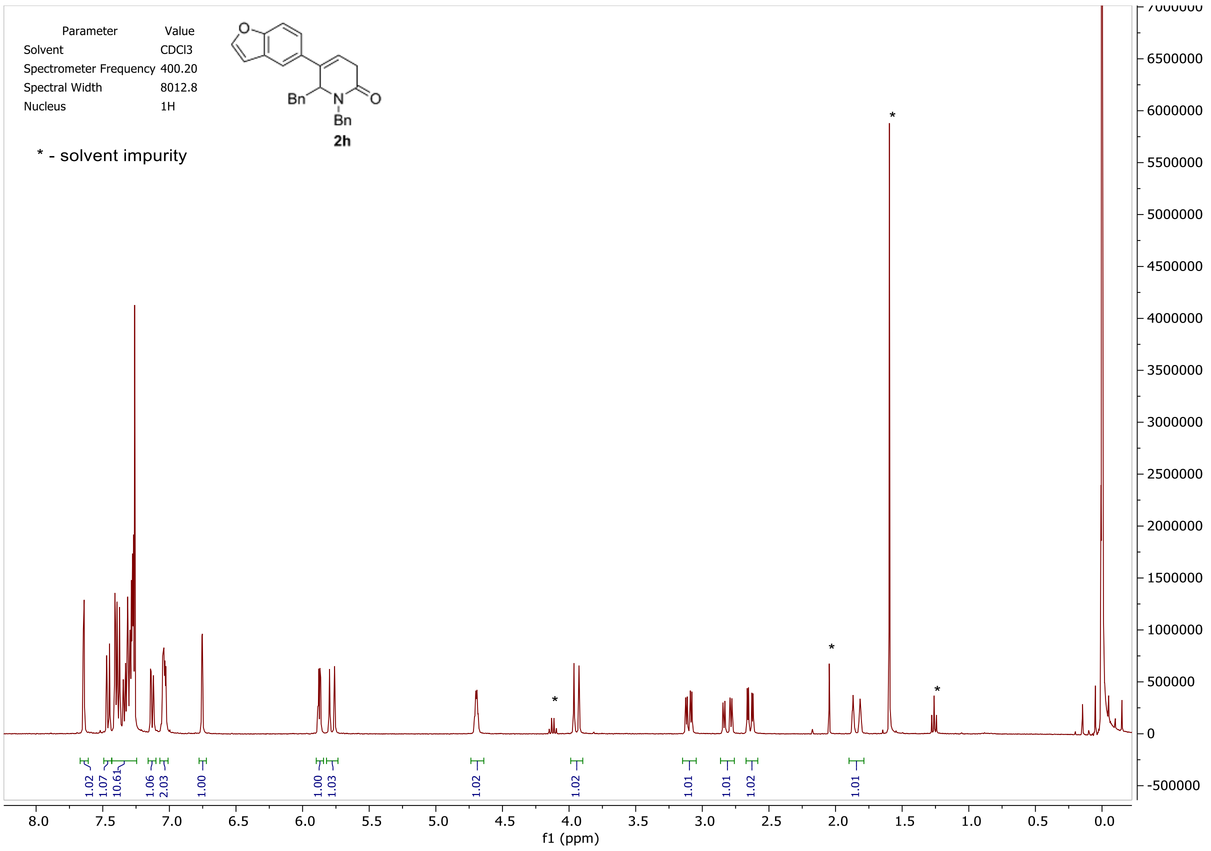
Parameter Value
Solvent CDCl3
Spectrometer Frequency 400.20
Spectral Width 8012.8
Nucleus 1H
* - solvent impurity
Bn
N
Bn
O
2h
1.02
1.07
10.61
1.06
2.03
1.00
1.00
1.03
1.02
1.02
1.01
1.01
1.02
1.01
f1 (ppm)

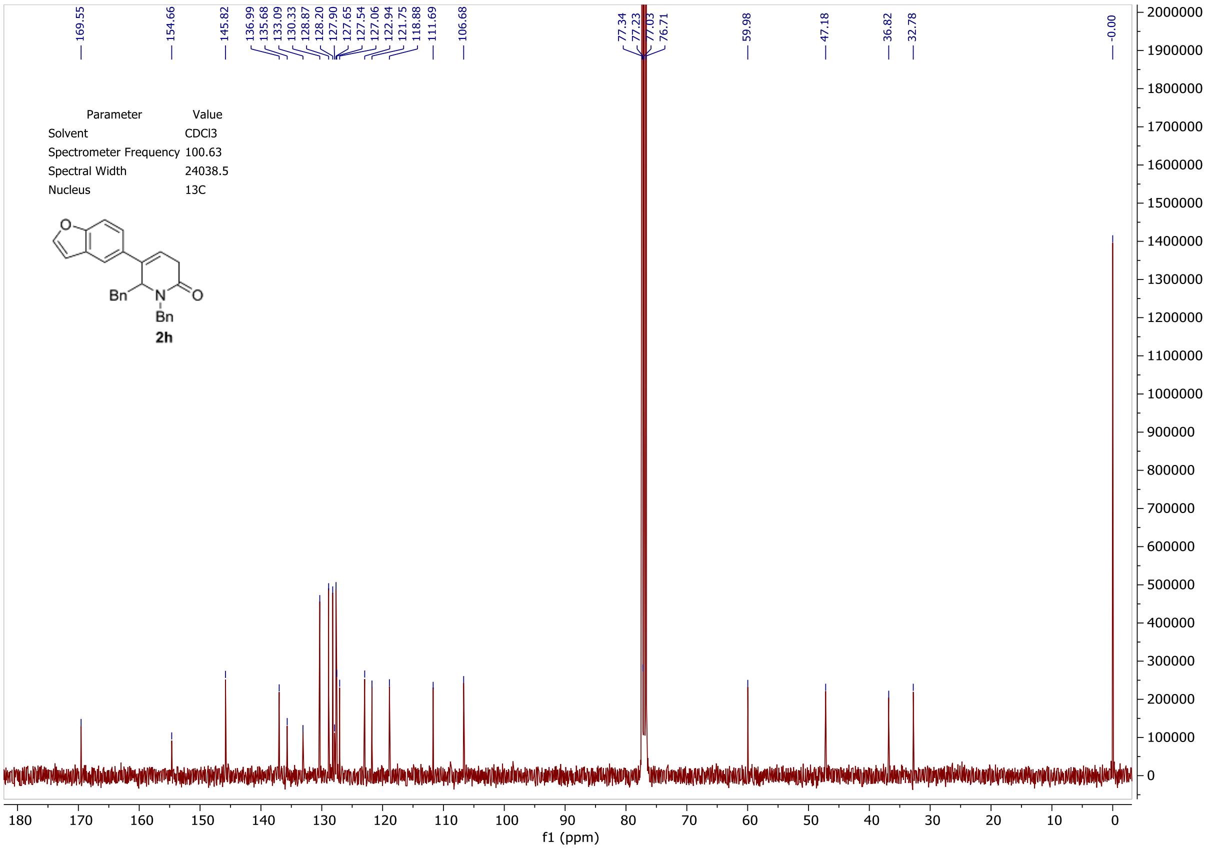
169.55
154.66
145.82
136.99
135.68
133.09
130.33
128.87
128.20
127.90
127.65
127.54
127.06
122.94
121.75
118.88
111.69
106.68
77.34
77.23
77.03
76.71
59.98
47.18
36.82
32.78
-0.00
Parameter Value
Solvent CDCl3
Spectrometer Frequency 100.63
Spectral Width 24038.5
Nucleus 13C
Bn
N
Bn
O
2h
f1 (ppm)

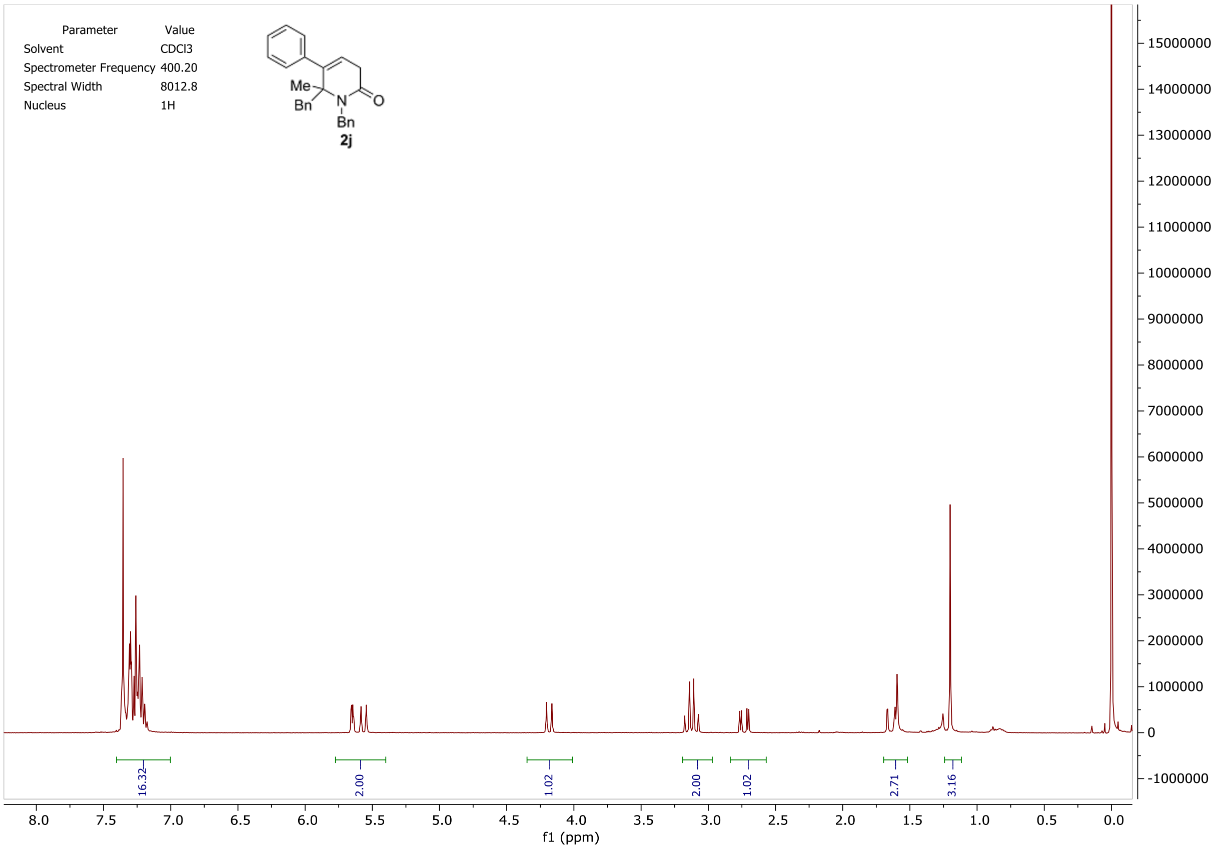
Parameter Value
Solvent CDCl3
Spectrometer Frequency 400.20
Spectral Width 8012.8
Nucleus 1H
Me
Bn
N
Bn
O
2j
16.32
2.00
1.02
2.00
1.02
2.71
3.16
f1 (ppm)

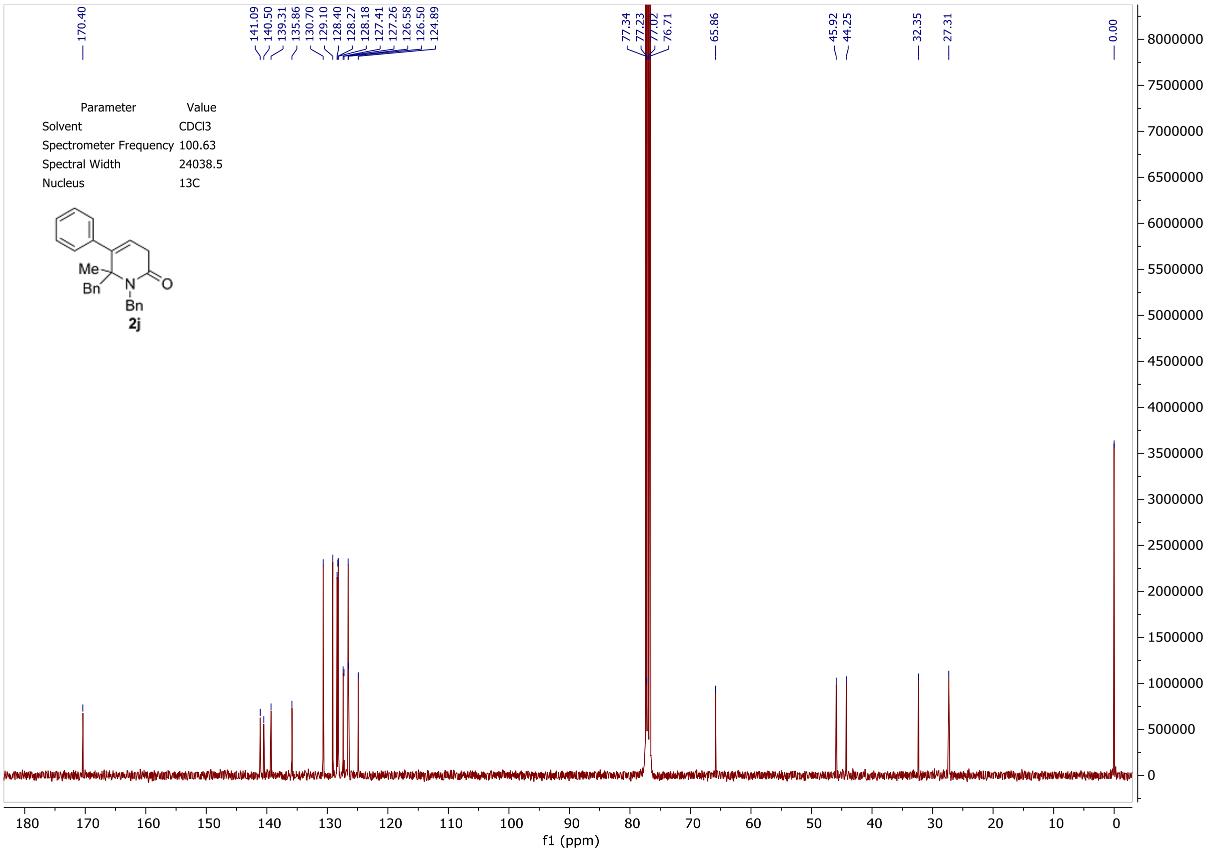
170.40
141.09
140.50
139.31
135.86
130.70
129.10
128.40
128.27
128.18
127.41
127.26
126.58
126.50
124.89
77.34
77.23
77.02
76.71
65.86
45.92
44.25
32.35
27.31
0.00
Parameter Value
Solvent CDCl3
Spectrometer Frequency 100.63
Spectral Width 24038.5
Nucleus 13C
Me
Bn
N
Bn
O
2j
f1 (ppm)

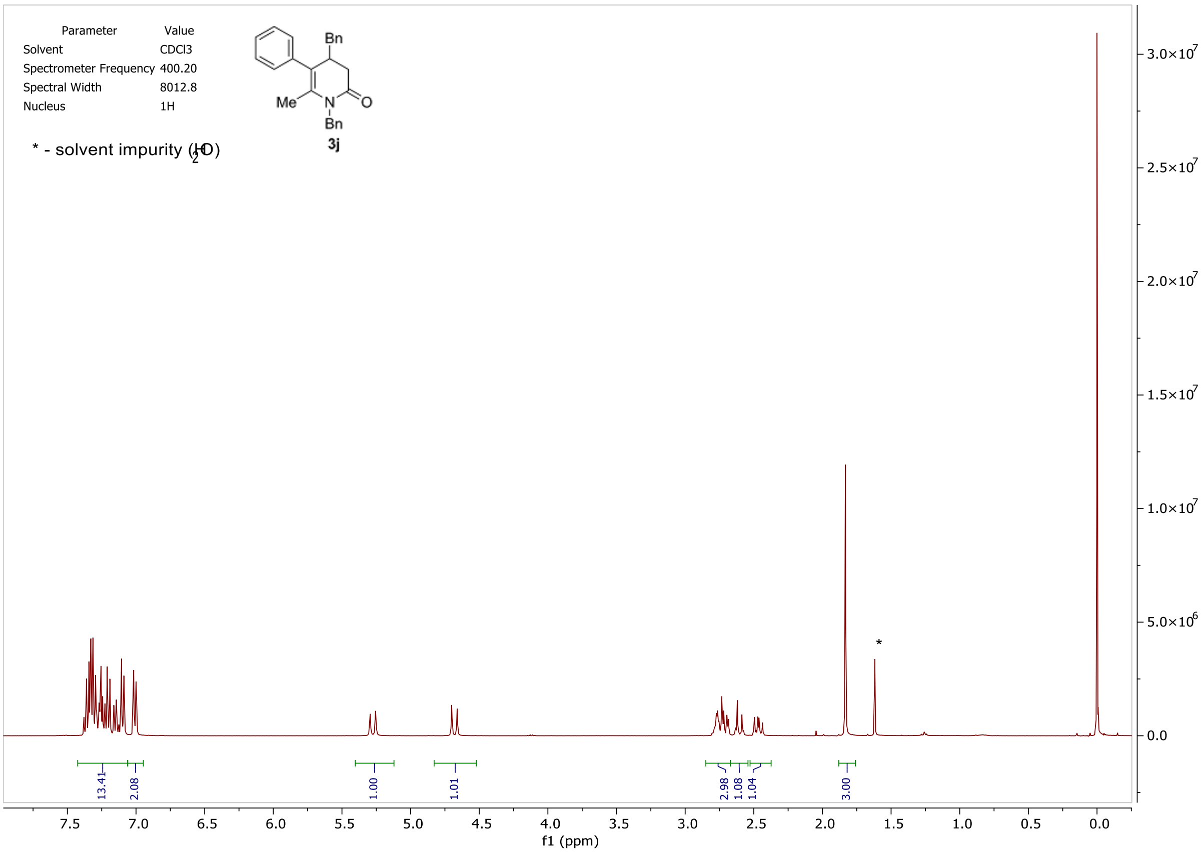
Parameter Value
Solvent CDCl3
Spectrometer Frequency 400.20
Spectral Width 8012.8
Nucleus 1H
* - solvent impurity (H2O)
Bn
Me
N
O
Bn
3j
13.41
2.08
1.00
1.01
2.98
1.08
1.04
3.00
f1 (ppm)

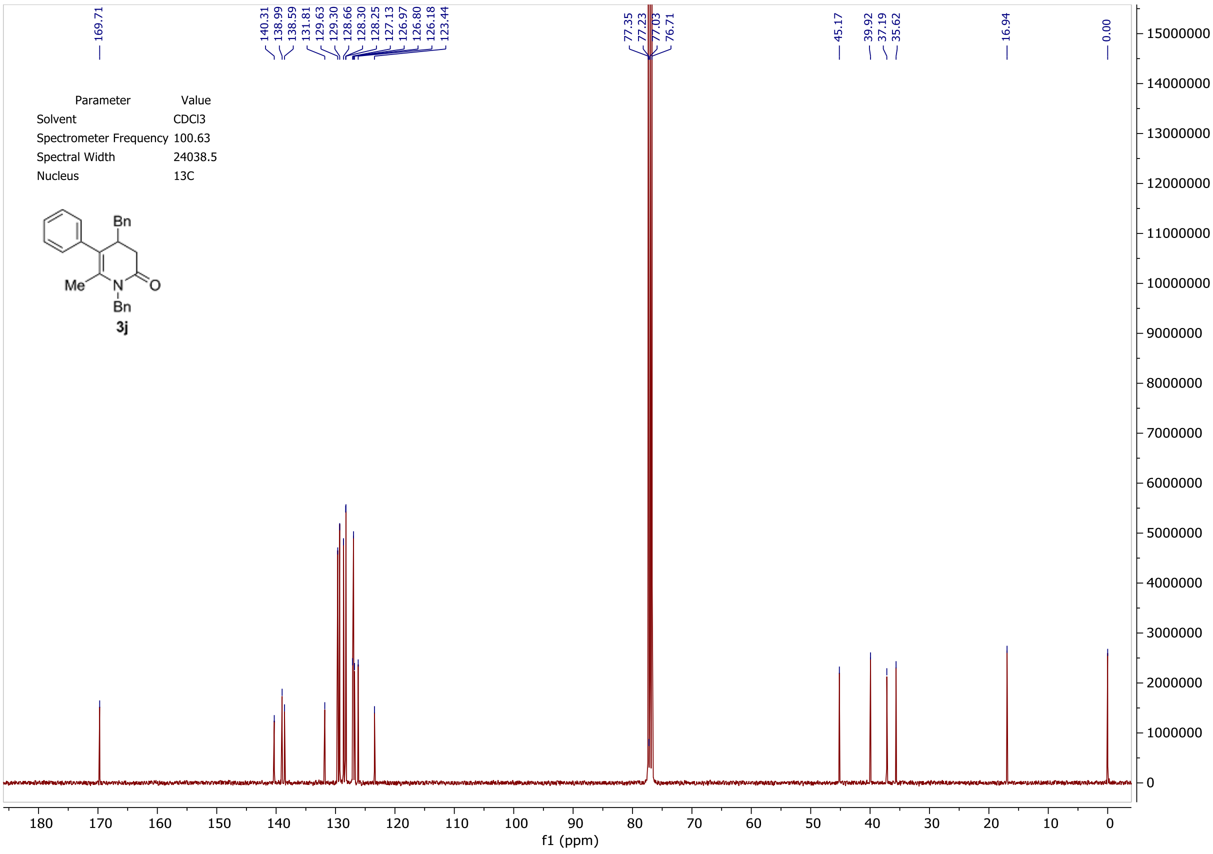
169.71
140.31
138.99
138.59
131.81
129.63
129.30
128.66
128.30
128.25
127.13
126.97
126.80
126.18
123.44
77.35
77.23
77.03
76.71
45.17
39.92
37.19
35.62
16.94
0.00
Parameter Value
Solvent CDCl3
Spectrometer Frequency 100.63
Spectral Width 24038.5
Nucleus 13C
Bn
Me
N
O
Bn
3j
f1 (ppm)

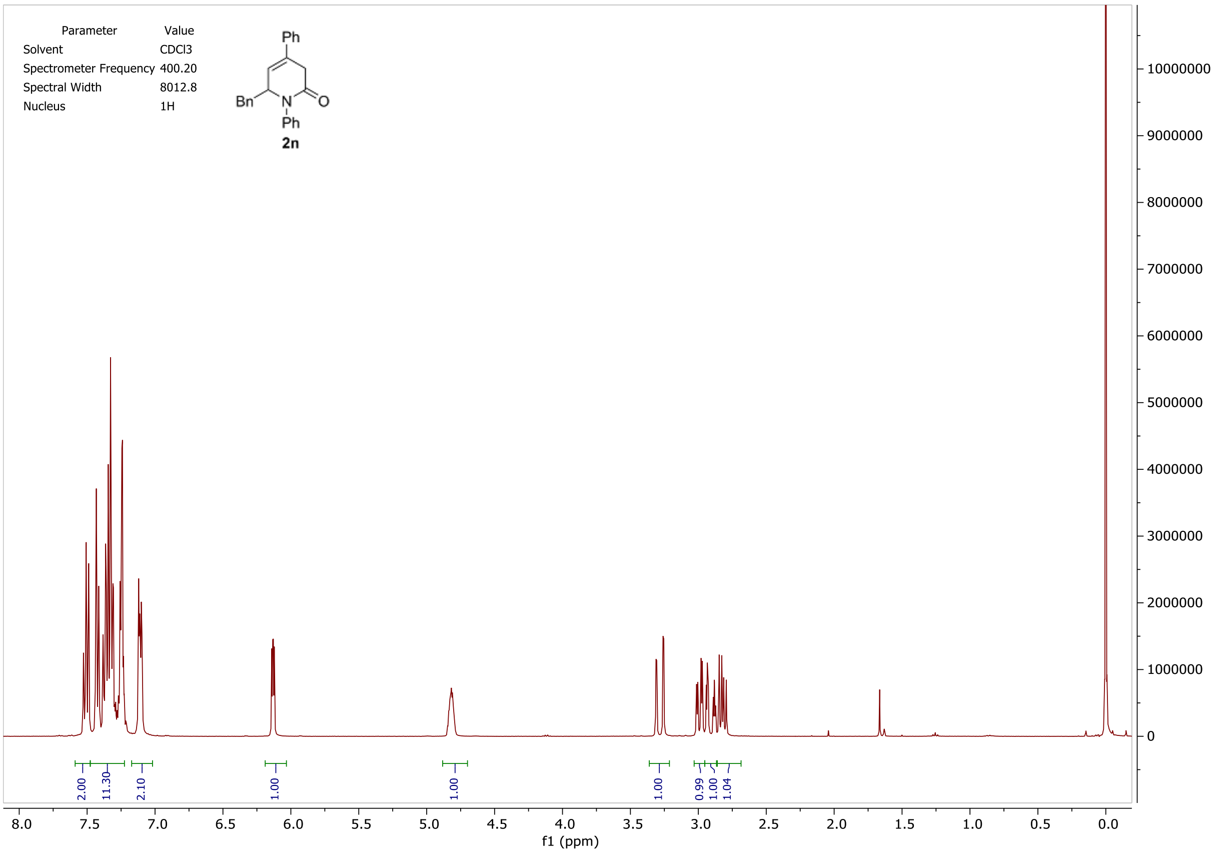
Parameter Value
Solvent CDCl3
Spectrometer Frequency 400.20
Spectral Width 8012.8
Nucleus 1H
Ph
Bn
N
O
Ph
2n
2.00
11.30
2.10
1.00
1.00
1.00
0.99
1.00
1.04
f1 (ppm)

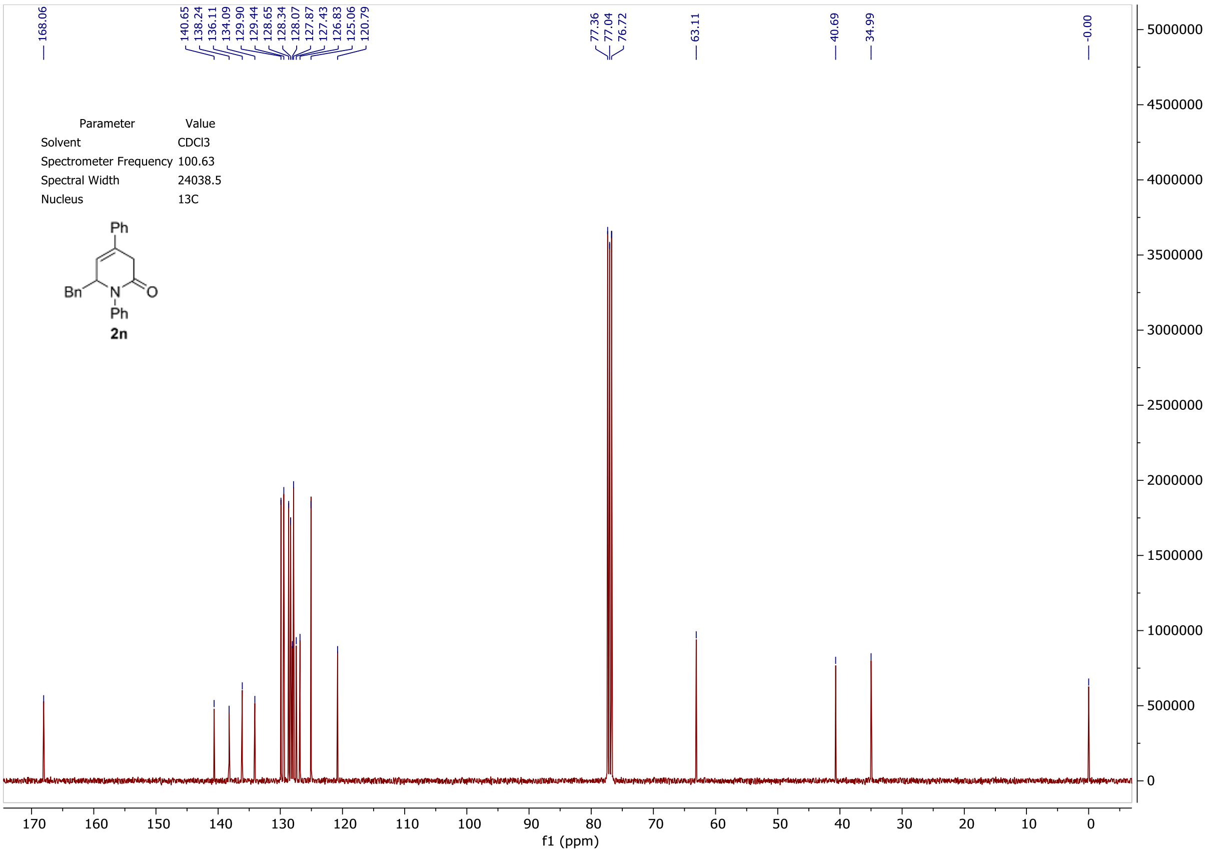
168.06
140.65
138.24
136.11
134.09
129.90
129.44
128.65
128.34
128.07
127.87
127.43
126.83
125.06
120.79
77.36
77.04
76.72
63.11
40.69
34.99
-0.00
Parameter Value
Solvent CDCl3
Spectrometer Frequency 100.63
Spectral Width 24038.5
Nucleus 13C
Ph
Bn
N
O
Ph
2n
f1 (ppm)

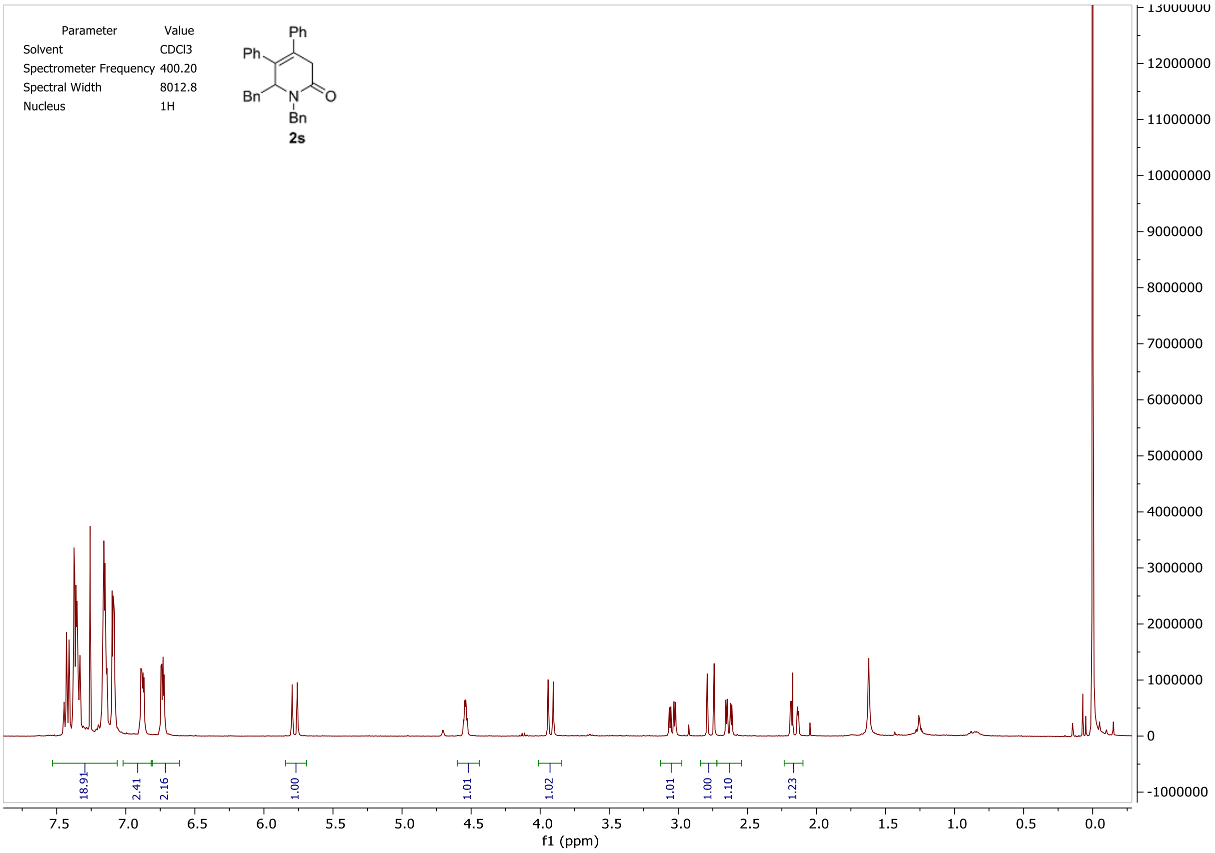
Parameter Value
Solvent CDCl3
Spectrometer Frequency 400.20
Spectral Width 8012.8
Nucleus 1H
Ph
Ph
Bn
N
O
Bn
2s
18.91
2.41
2.16
1.00
1.01
1.02
1.01
1.00
1.10
1.23
f1 (ppm)

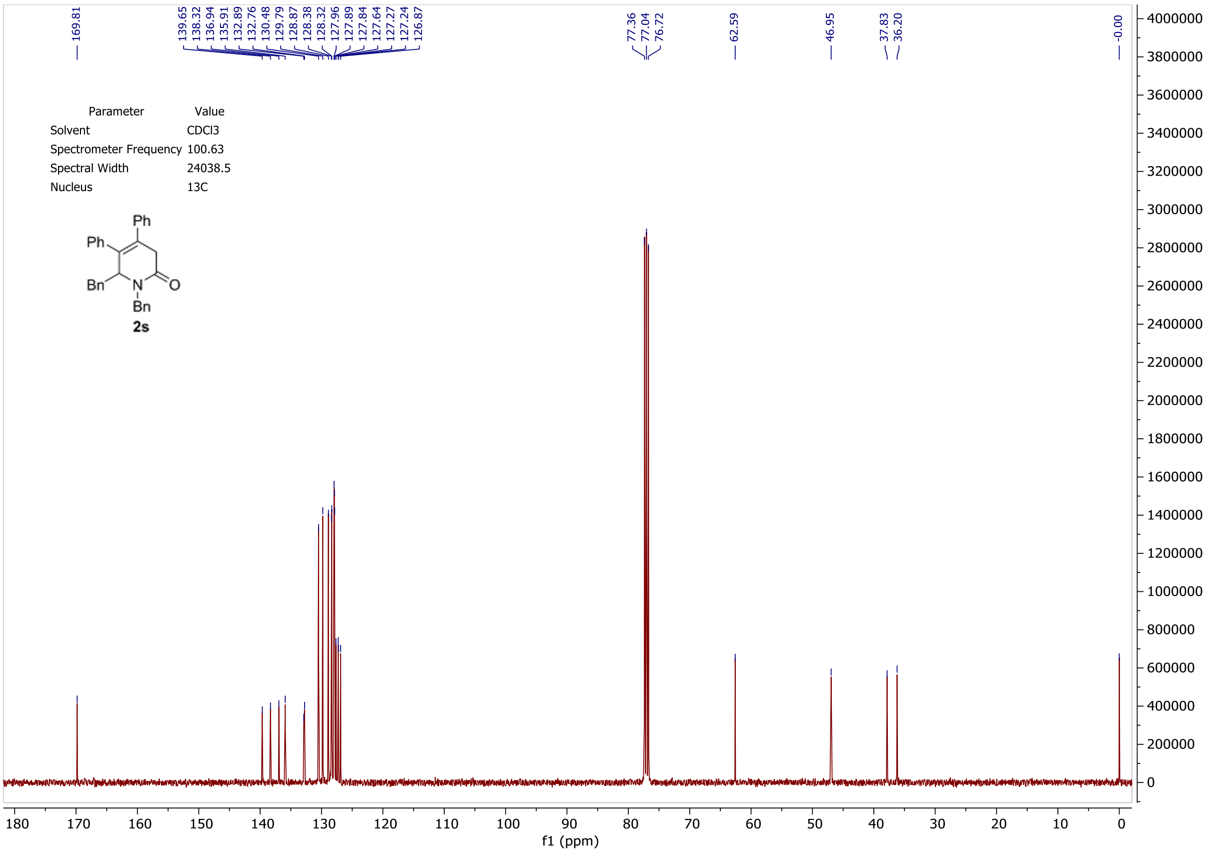
169.81
139.65
138.32
136.94
135.91
132.89
132.76
130.48
129.79
128.87
128.38
128.32
127.96
127.89
127.84
127.64
127.27
127.24
126.87
77.36
77.04
76.72
62.59
46.95
37.83
36.20
-0.00
Parameter Value
Solvent CDCl3
Spectrometer Frequency 100.63
Spectral Width 24038.5
Nucleus 13C
Ph
Ph
Bn
N
O
Bn
2s
f1 (ppm)

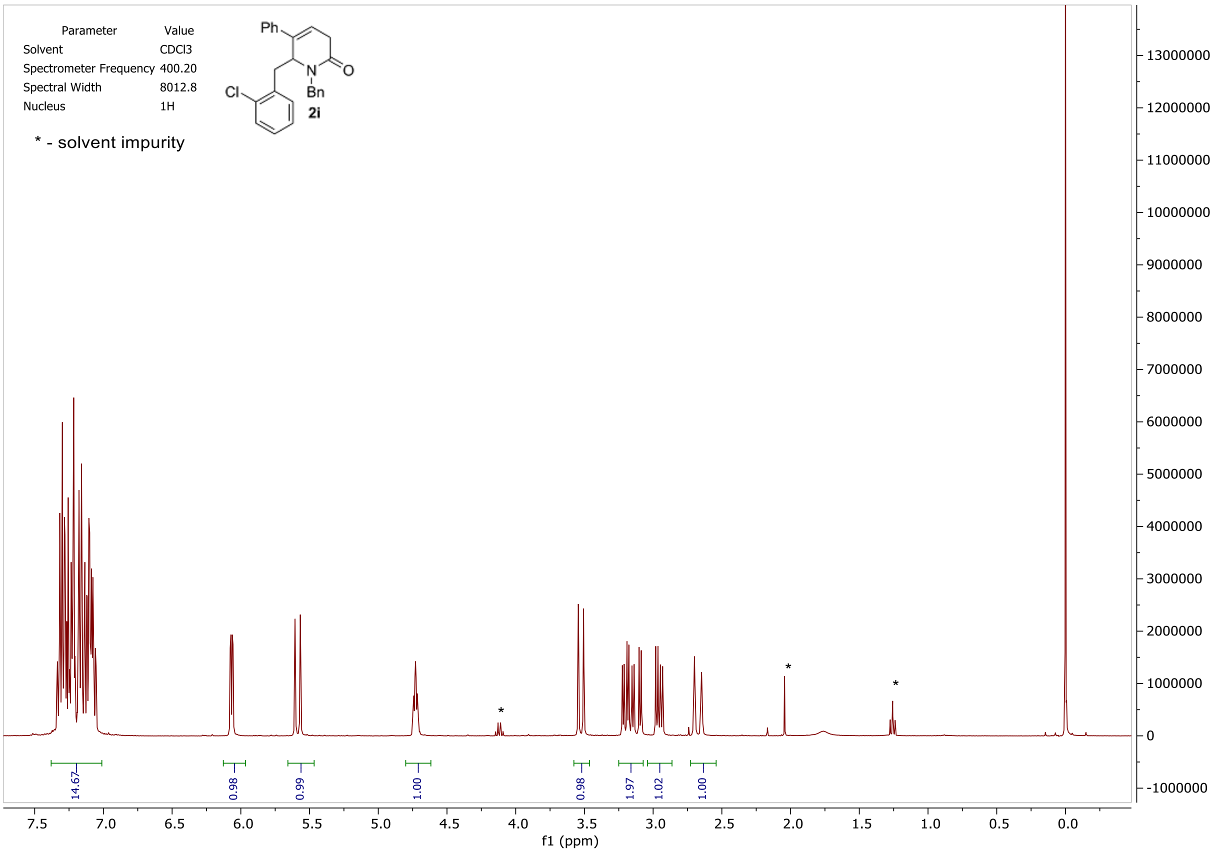

Parameter Value
Solvent CDCl3
Spectrometer Frequency 400.20
Spectral Width 8012.8
Nucleus 1H
* - solvent impurity
Ph
Cl
N
O
Bn
2i
14.67
0.98
0.99
1.00
0.98
1.97
1.02
1.00
f1 (ppm)

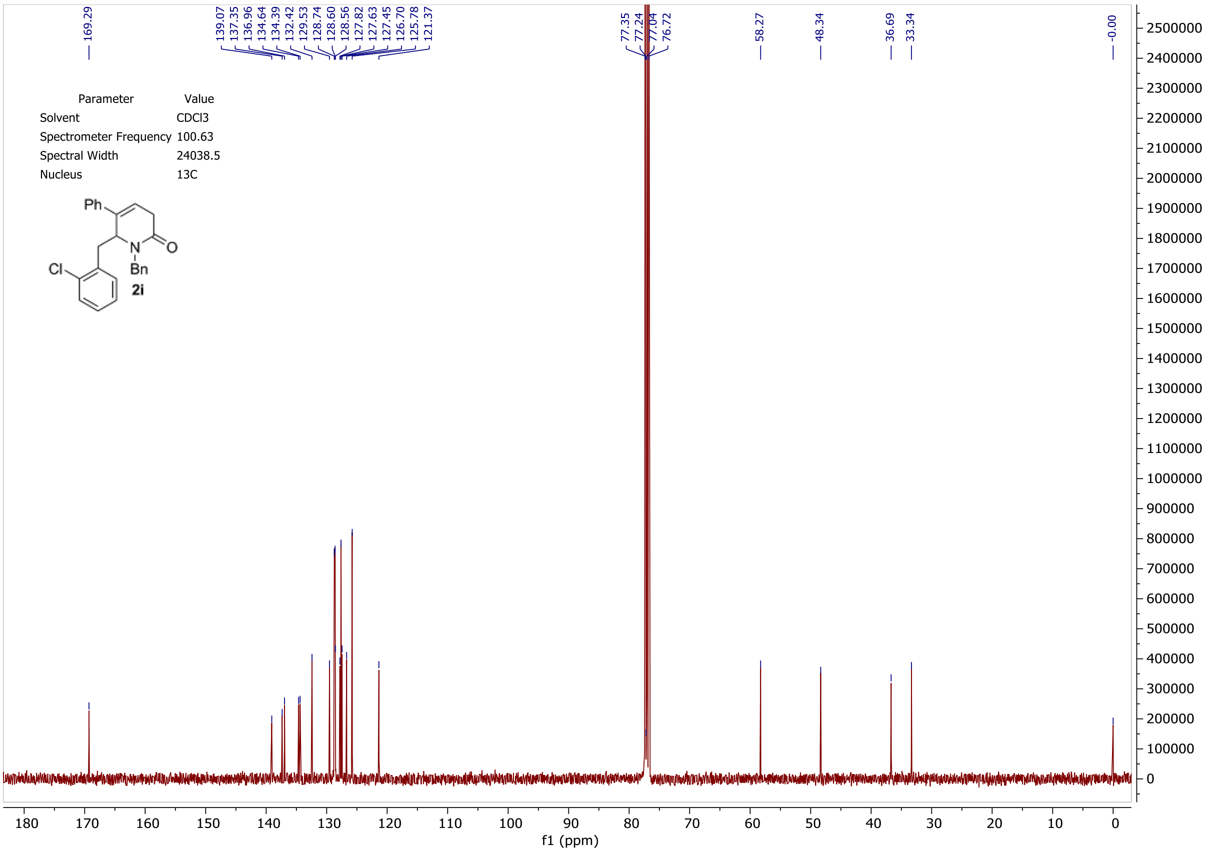

169.29
139.07
137.35
136.96
134.64
134.39
132.42
129.53
128.74
128.60
128.56
127.82
127.63
127.45
126.70
125.78
121.37
77.35
77.24
77.04
76.72
58.27
48.34
36.69
33.34
-0.00
Parameter Value
Solvent CDCl3
Spectrometer Frequency 100.63
Spectral Width 24038.5
Nucleus 13C
Ph
Cl
N
O
Bn
2i
f1 (ppm)

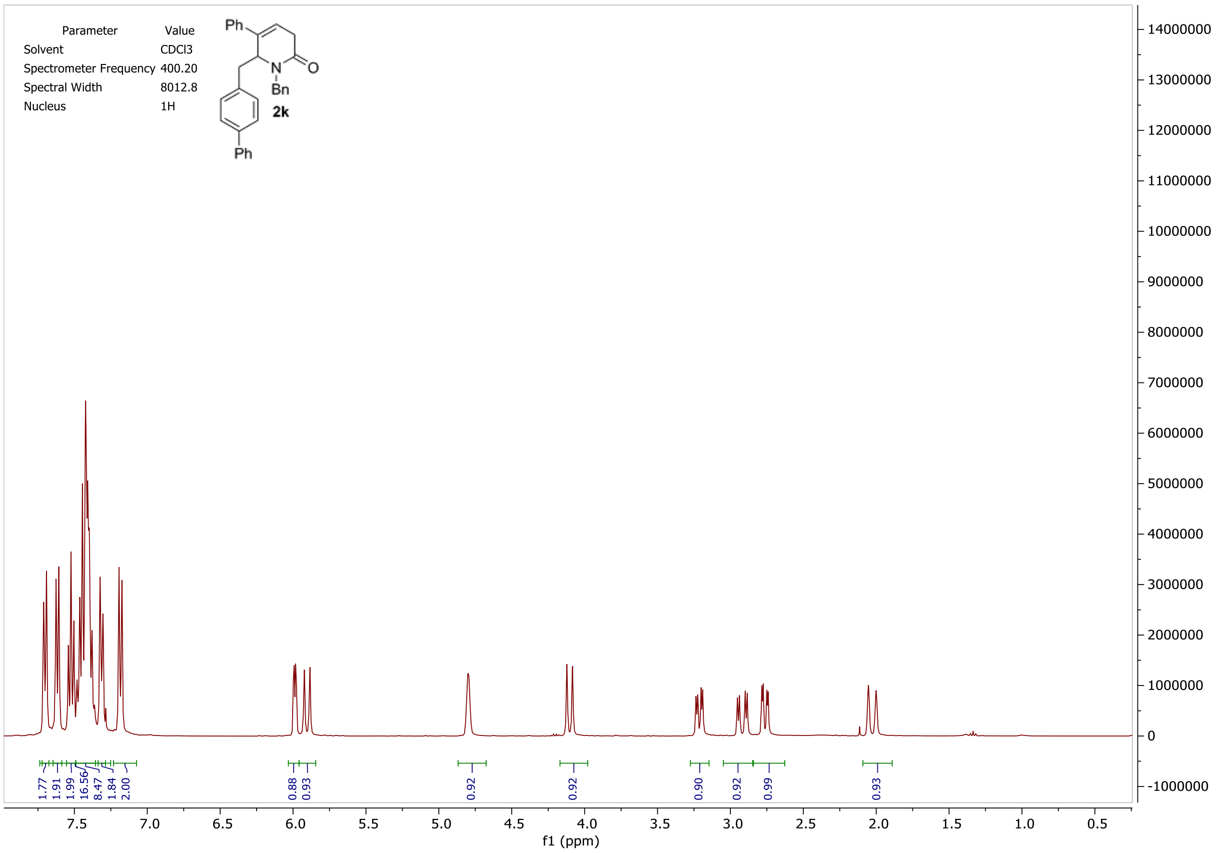
Parameter Value
Solvent CDCl3
Spectrometer Frequency 400.20
Spectral Width 8012.8
Nucleus 1H
Ph
O
N
Bn
2k
Ph
1.77 1.91 1.99 16.56 8.47 1.84 2.00
0.88 0.93
0.92
0.92
0.90 0.92 0.99
0.93
f1 (ppm)

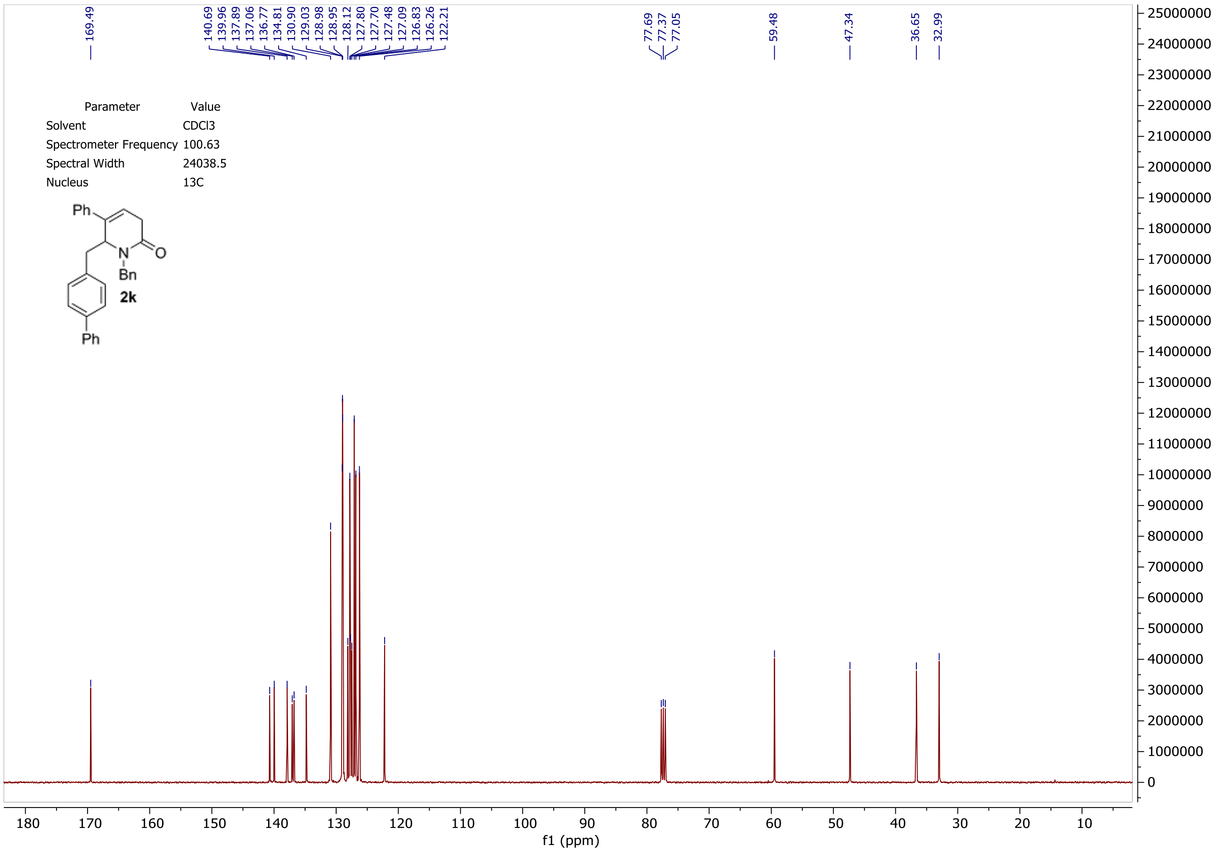
169.49
140.69 139.96 137.89 137.06 136.77 134.81 130.90 129.03 128.98 128.95 128.12 127.80 127.70 127.48 127.09 126.83 126.26 122.21
77.69 77.37 77.05
59.48
47.34
36.65
32.99
Parameter Value
Solvent CDCl3
Spectrometer Frequency 100.63
Spectral Width 24038.5
Nucleus 13C
Ph
O
N
Bn
2k
Ph
f1 (ppm)

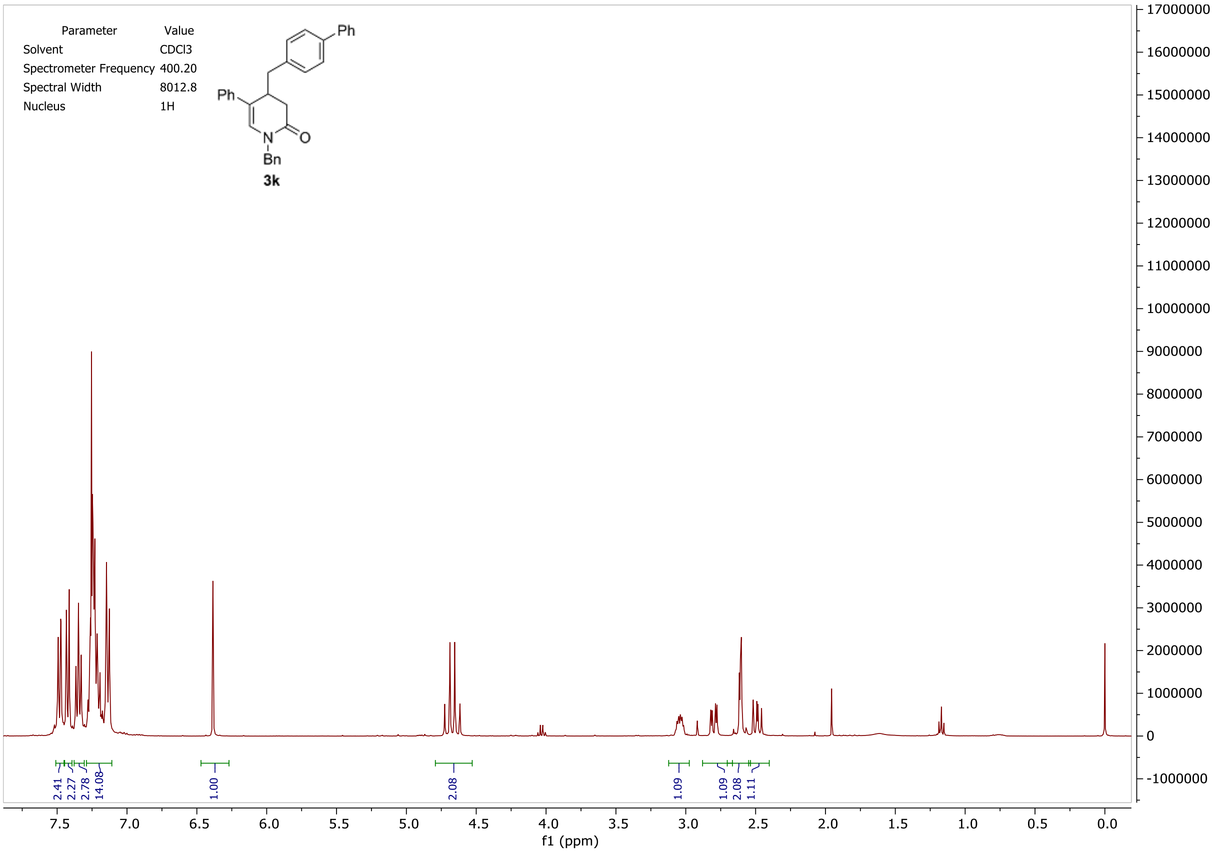

Parameter Value
Solvent CDCl3
Spectrometer Frequency 400.20
Spectral Width 8012.8
Nucleus 1H
Ph
Ph
N
O
Bn
3k
2.41
2.27
2.78
14.08
1.00
2.08
1.09
1.09
2.08
1.11
f1 (ppm)

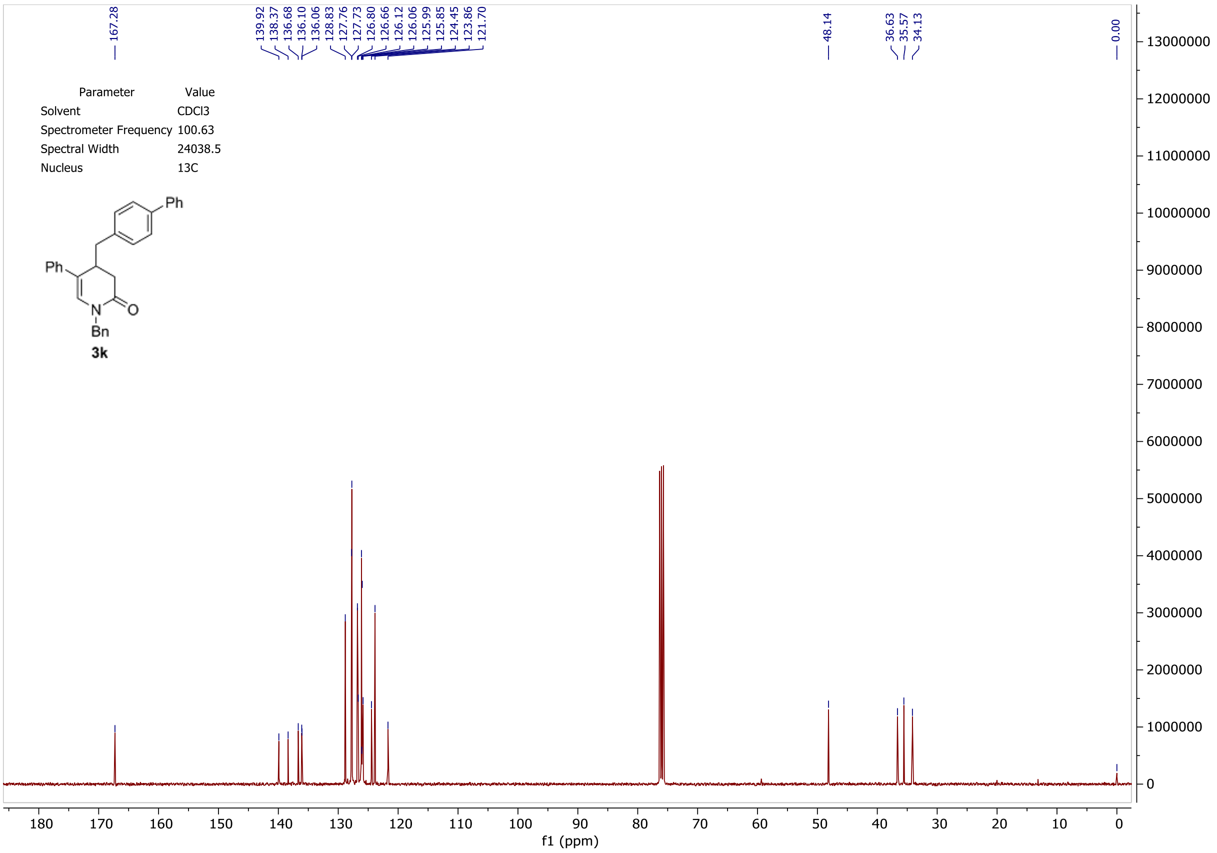

167.28
139.92
138.37
136.68
136.10
136.06
128.83
127.76
127.73
126.80
126.66
126.12
126.06
125.99
125.85
124.45
123.86
121.70
48.14
36.63
35.57
34.13
0.00
Parameter Value
Solvent CDCl3
Spectrometer Frequency 100.63
Spectral Width 24038.5
Nucleus 13C
Ph
Ph
N
O
Bn
3k
f1 (ppm)

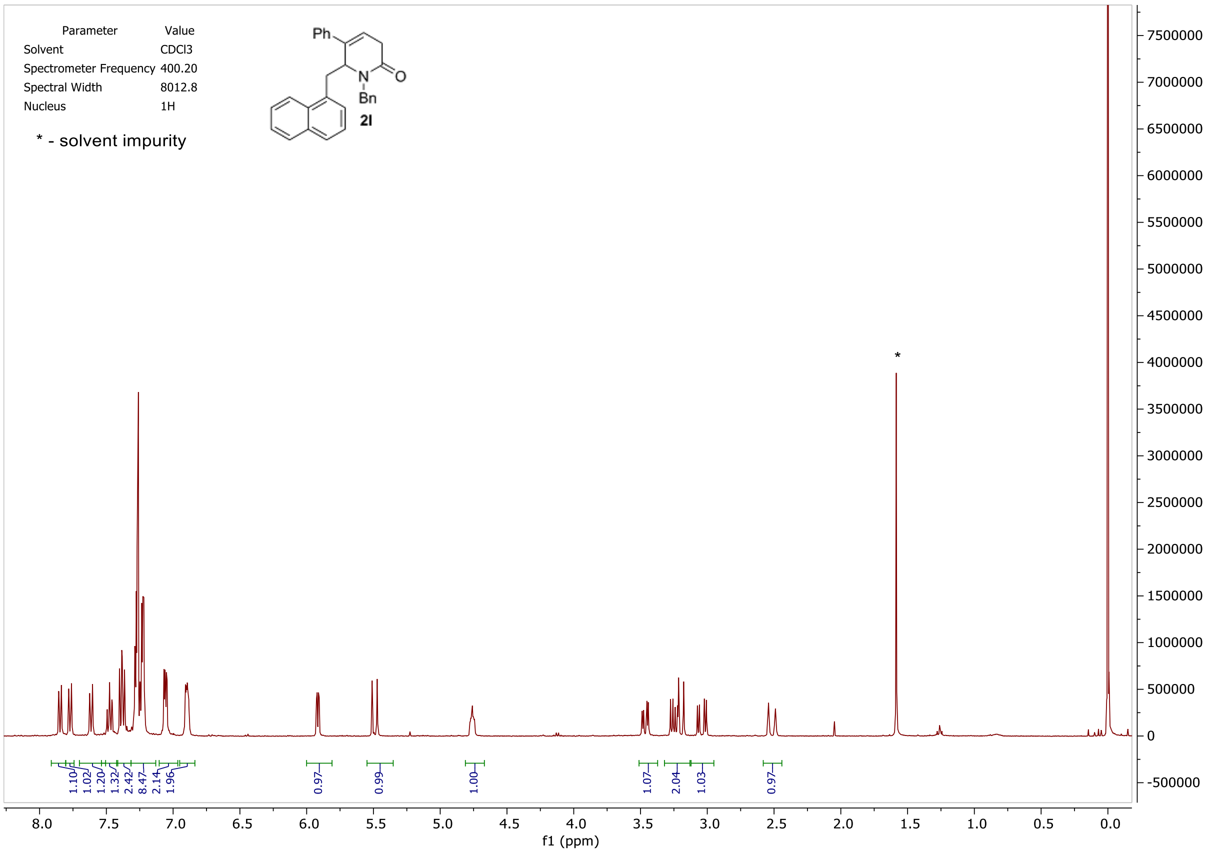
Parameter Value
Solvent CDCl3
Spectrometer Frequency 400.20
Spectral Width 8012.8
Nucleus 1H
* - solvent impurity
Ph
N
O
Bn
2l
1.10 1.02 1.20 1.32 2.42 8.47 2.14 1.96
0.97
0.99
1.00
1.07 2.04 1.03
0.97
f1 (ppm)

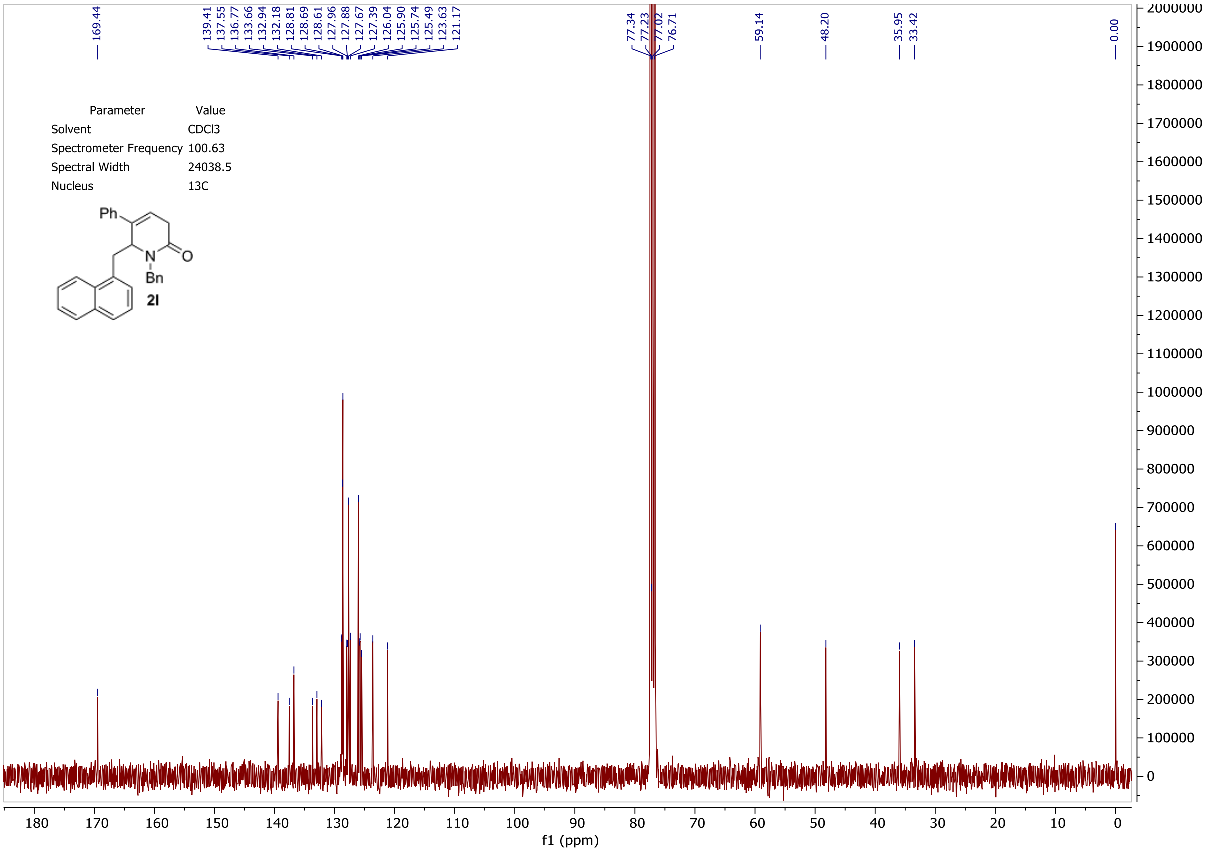
169.44
139.41 137.55 136.77 133.66 132.94 132.18 128.81 128.69 128.61 127.96 127.88 127.67 127.39 126.04 125.90 125.74 125.49 123.63 121.17
77.34 77.23 77.02 76.71
59.14
48.20
35.95 33.42
0.00
Parameter Value
Solvent CDCl3
Spectrometer Frequency 100.63
Spectral Width 24038.5
Nucleus 13C
Ph
N
O
Bn
2l
f1 (ppm)

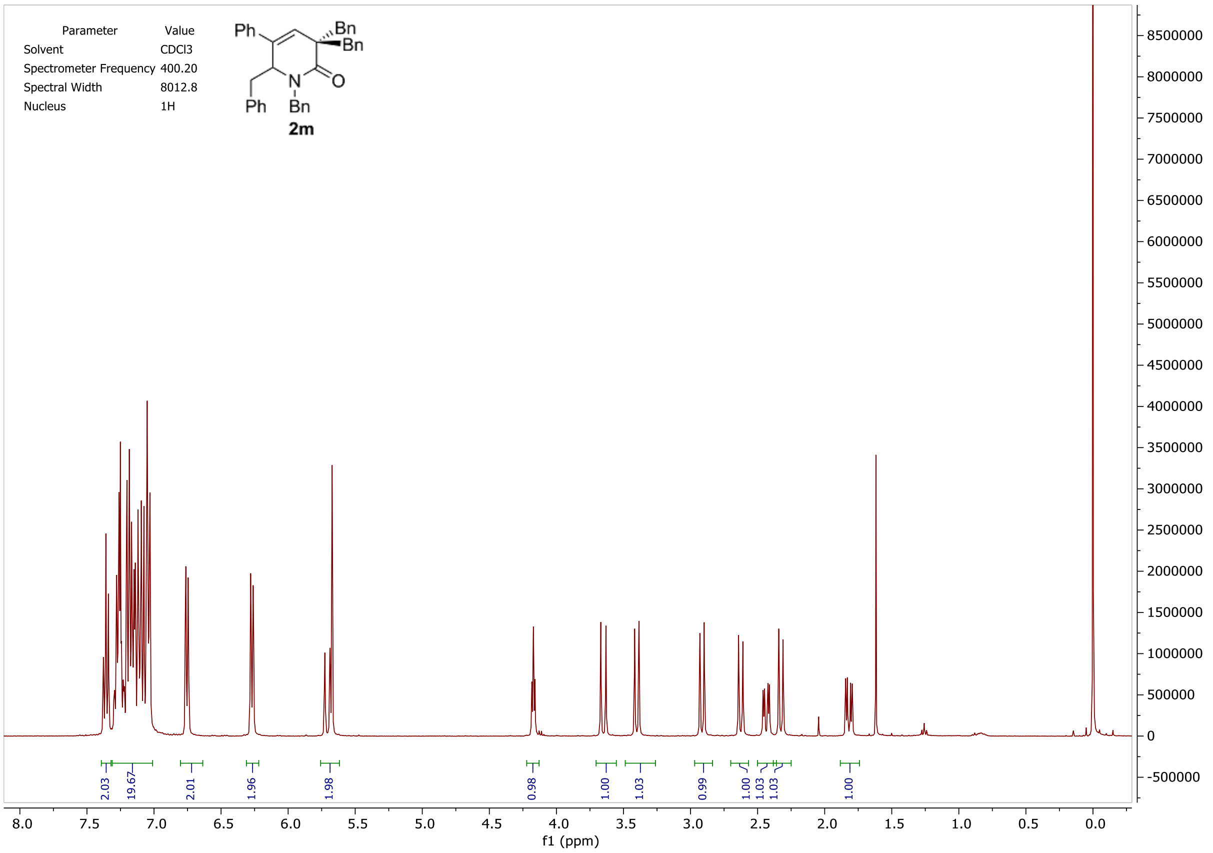

Parameter Value
Solvent CDCl3
Spectrometer Frequency 400.20
Spectral Width 8012.8
Nucleus 1H
Ph
Bn
Bn
O
N
Ph
Bn
2m
f1 (ppm)

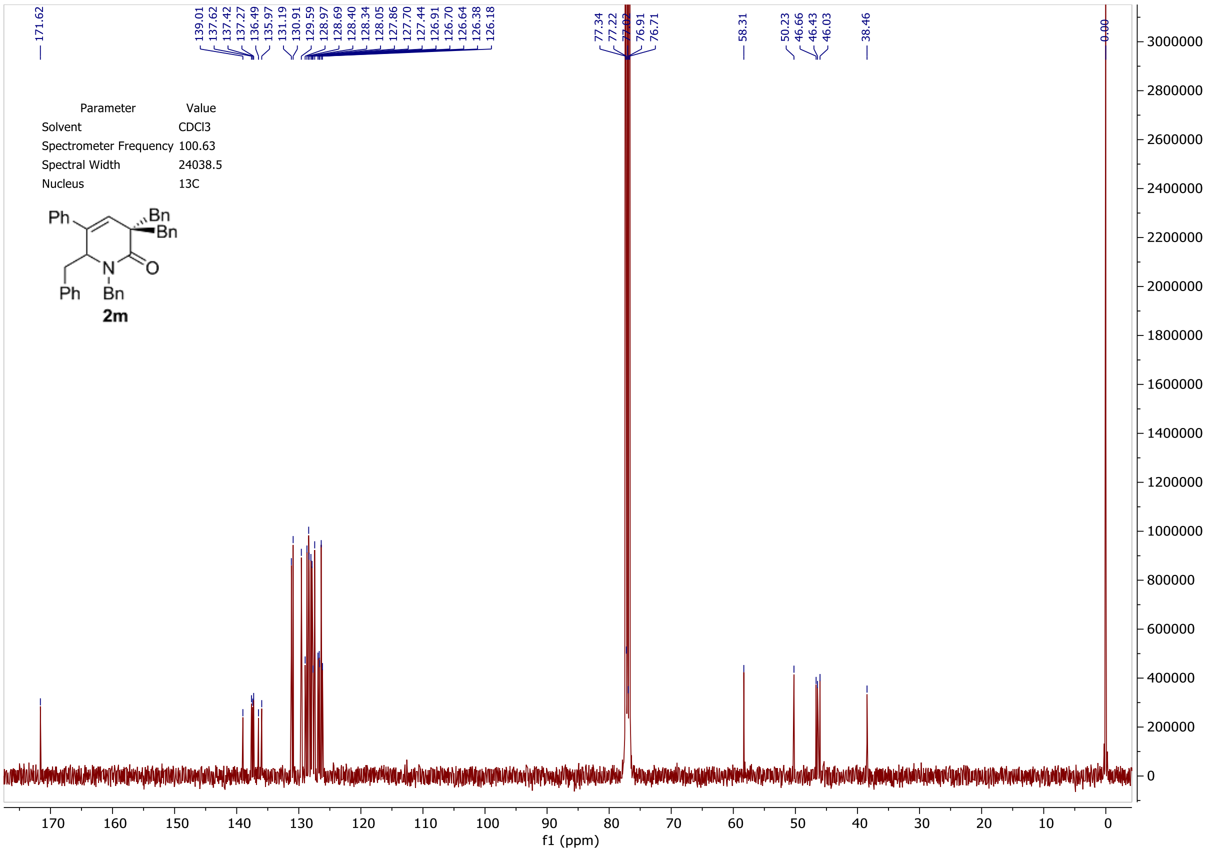

171.62
139.01 137.62 137.42 137.27 136.49 135.97 131.19 130.91 129.59 128.97 128.69 128.40 128.34 128.05 127.86 127.70 127.44 126.91 126.70 126.64 126.38 126.18
77.34 77.22 77.02 76.91 76.71
58.31
50.23 46.66 46.43 46.03
38.46
0.00
Parameter Value
Solvent CDCl3
Spectrometer Frequency 100.63
Spectral Width 24038.5
Nucleus 13C
Ph
Bn
Bn
O
N
Ph
Bn
2m
f1 (ppm)

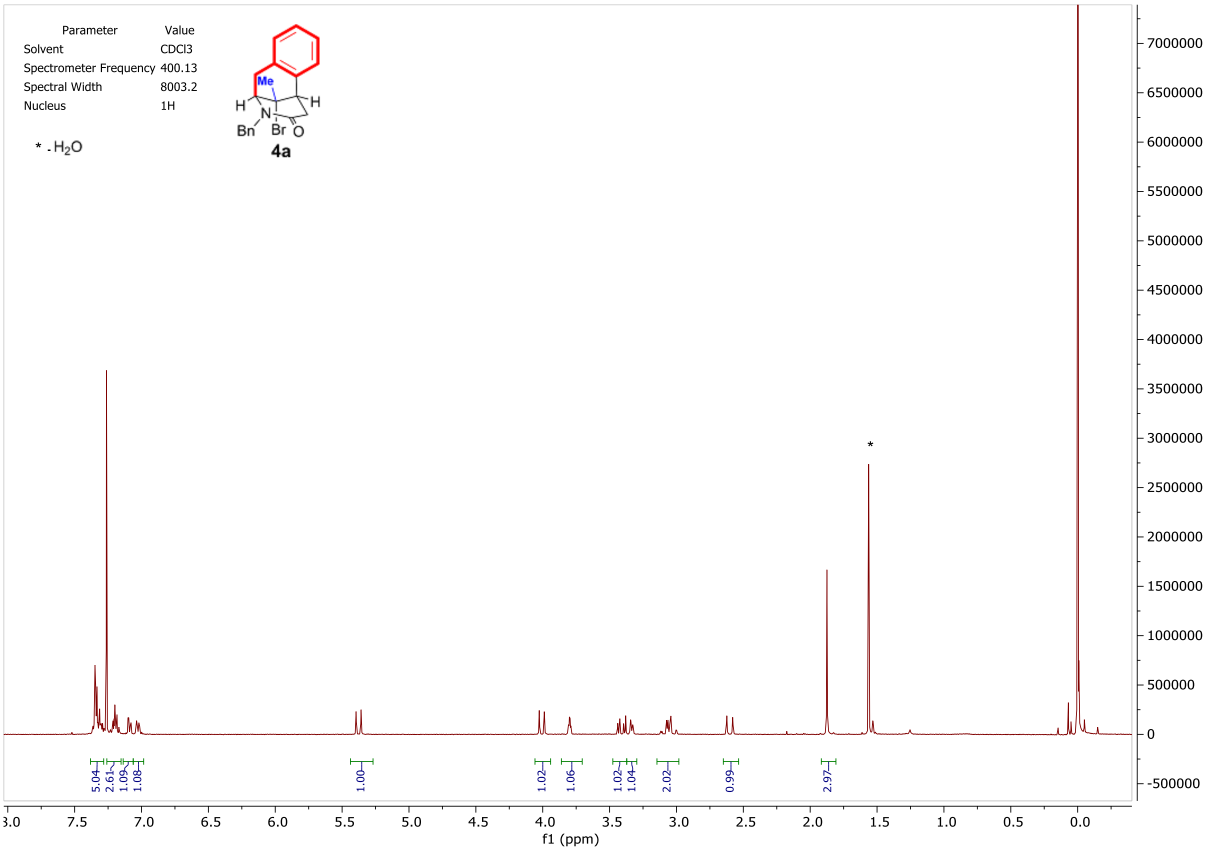
Parameter Value
Solvent CDCl3
Spectrometer Frequency 400.13
Spectral Width 8003.2
Nucleus 1H
* - H_2O
Me
H
H
N
Bn
Br
O
4a
*
5.04
2.61
1.09
1.08
1.00
1.02
1.06
1.02
1.04
2.02
0.99
2.97
f1 (ppm)

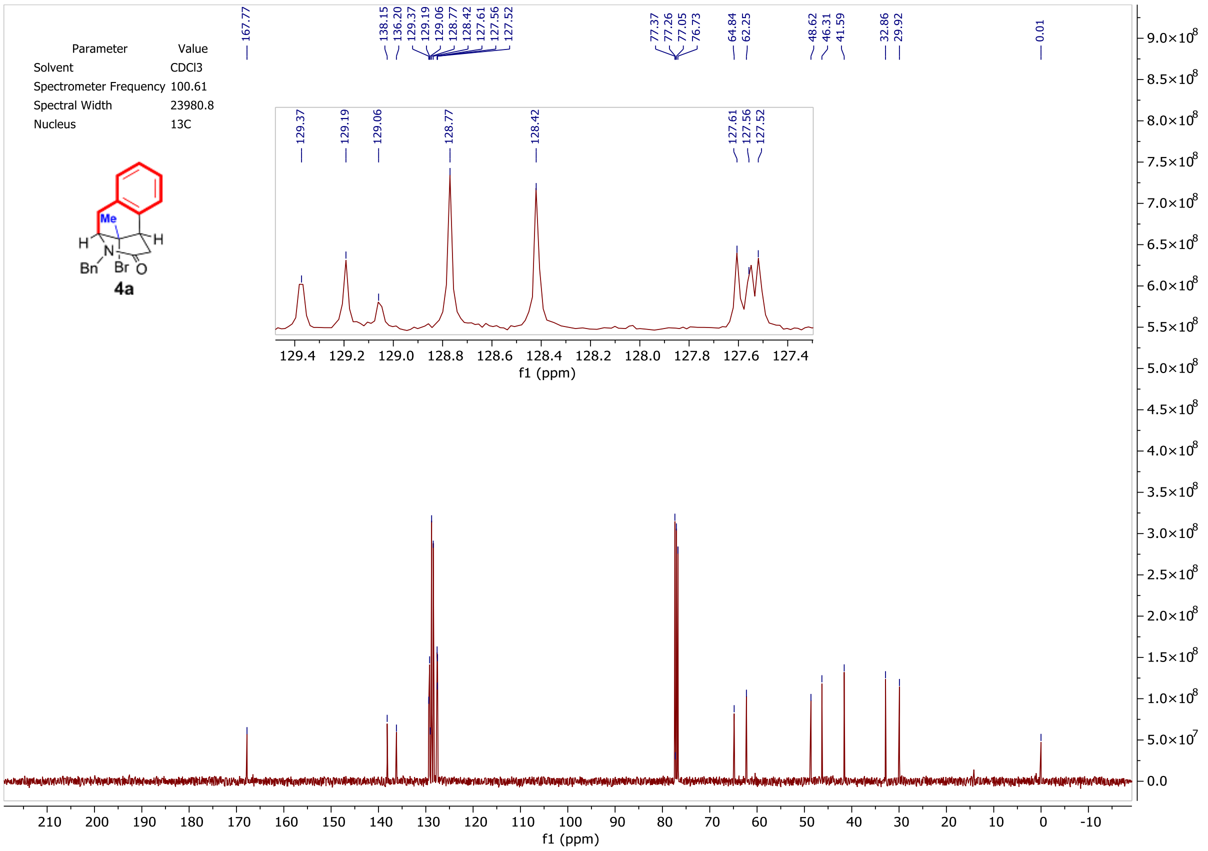
Parameter Value
Solvent CDCl3
Spectrometer Frequency 100.61
Spectral Width 23980.8
Nucleus 13C
Me
H
H
N
Bn
Br
O
4a
167.77
138.15
136.20
129.37
129.19
129.06
128.77
128.42
127.61
127.56
127.52
77.37
77.26
77.05
76.73
64.84
62.25
48.62
46.31
41.59
32.86
29.92
0.01
f1 (ppm)

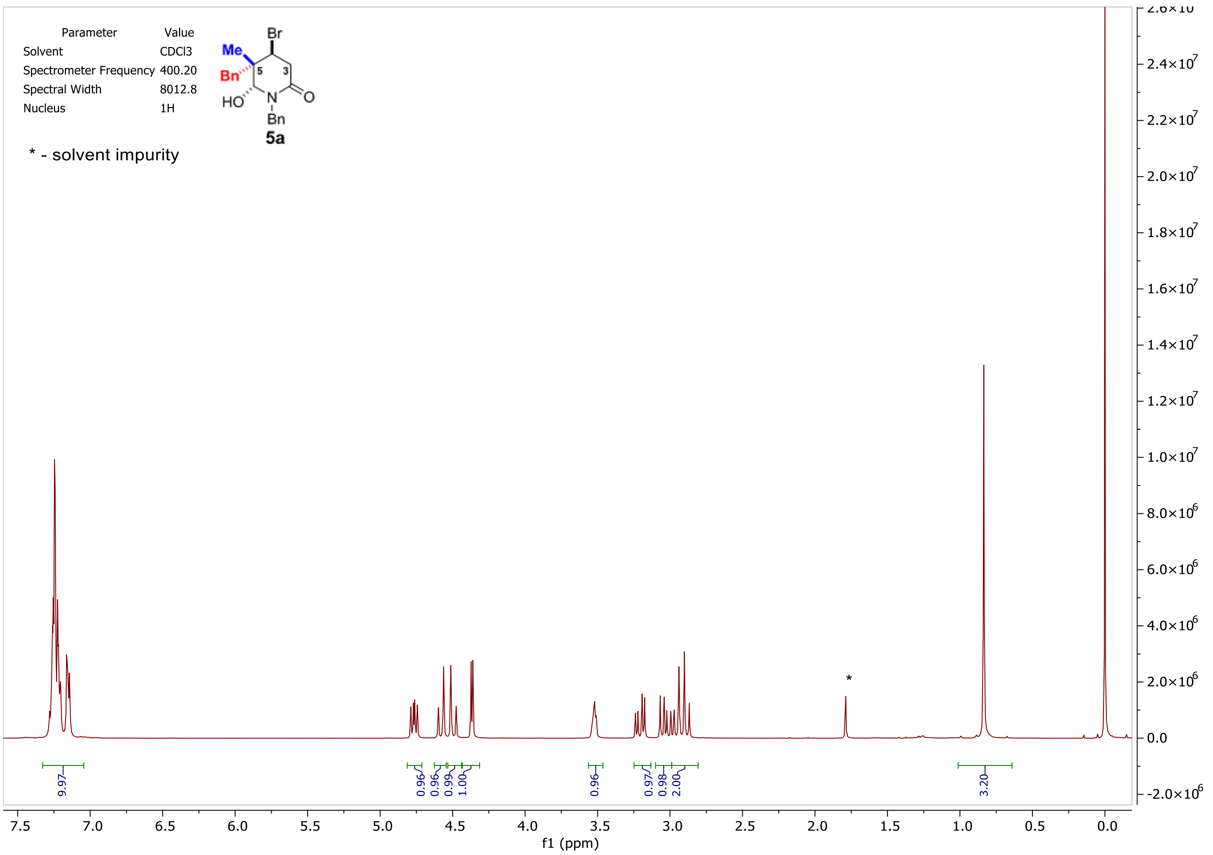
Parameter Value
Solvent CDCl3
Spectrometer Frequency 400.20
Spectral Width 8012.8
Nucleus 1H
Me
Br
Bn
HO
N
O
Bn
5a
* - solvent impurity
9.97
0.96
0.96
0.99
1.00
0.96
0.97
0.98
2.00
3.20
f1 (ppm)

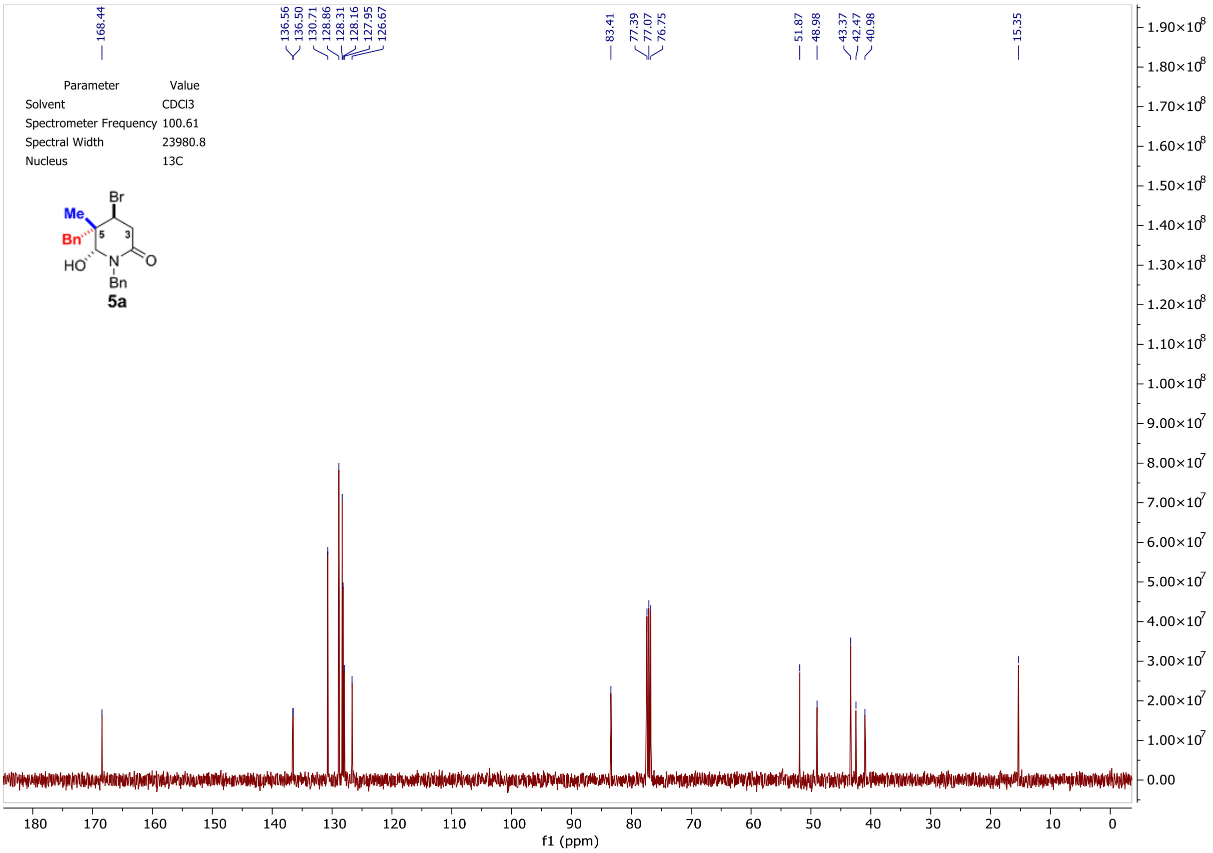
168.44
136.56
136.50
130.71
128.86
128.31
128.16
127.95
126.67
83.41
77.39
77.07
76.75
51.87
48.98
43.37
42.47
40.98
15.35
Parameter Value
Solvent CDCl3
Spectrometer Frequency 100.61
Spectral Width 23980.8
Nucleus 13C
Me
Br
Bn
HO
N
O
Bn
5a
f1 (ppm)

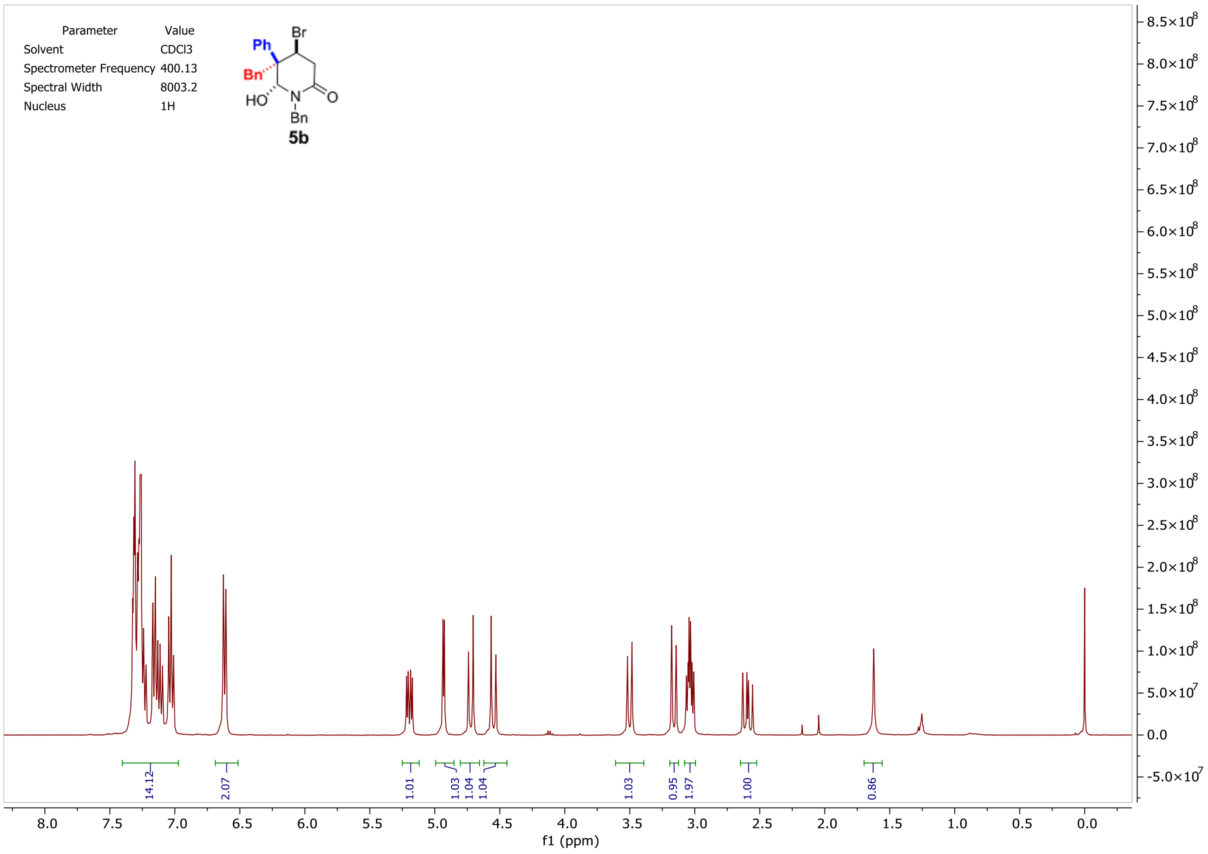

Parameter Value
Solvent CDCl3
Spectrometer Frequency 400.13
Spectral Width 8003.2
Nucleus 1H
Br
Ph
Bn
HO
N
O
Bn
5b
14.12
2.07
1.01
1.03
1.04
1.04
1.03
0.95
1.97
1.00
0.86
f1 (ppm)

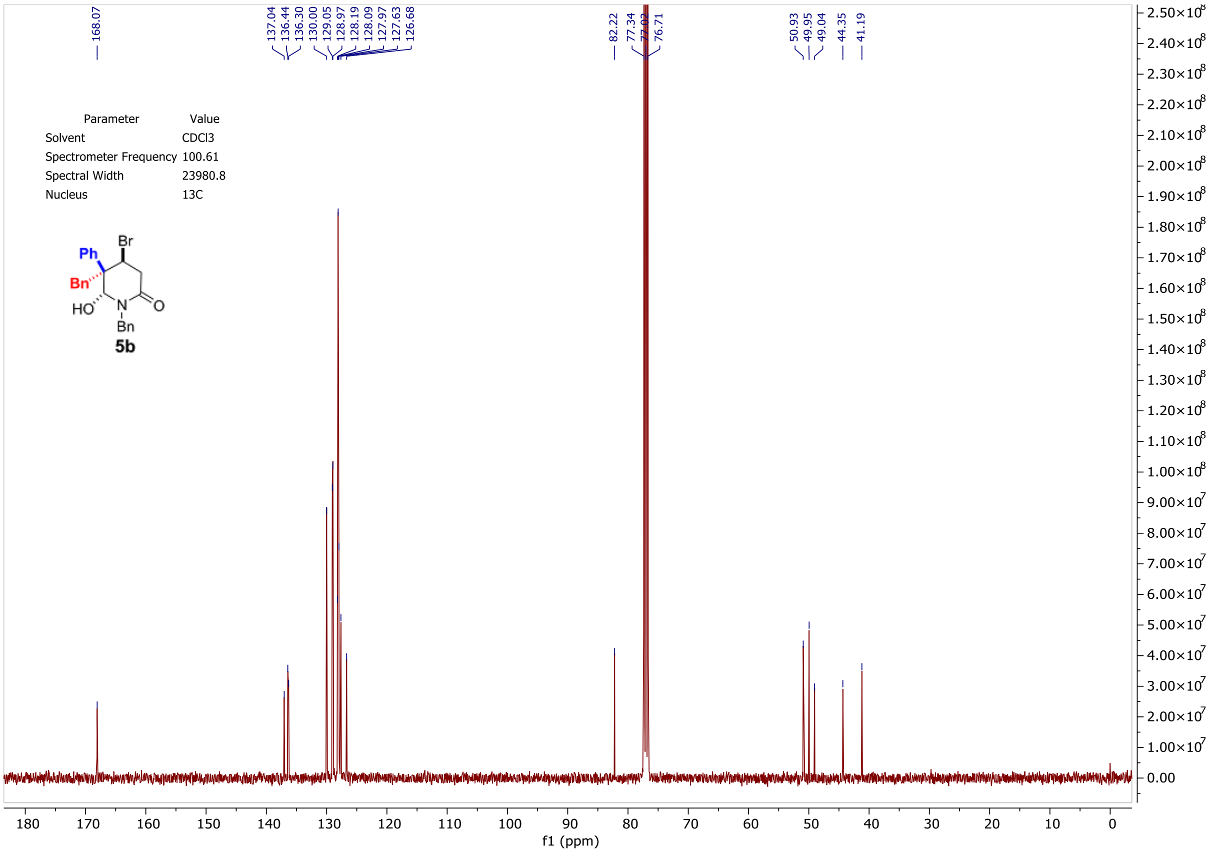

168.07
137.04
136.44
136.30
130.00
129.05
128.97
128.19
128.09
127.97
127.63
126.68
82.22
77.34
76.71
50.93
49.95
49.04
44.35
41.19
Parameter Value
Solvent CDCl3
Spectrometer Frequency 100.61
Spectral Width 23980.8
Nucleus 13C
Br
Ph
Bn
HO
N
O
Bn
5b
f1 (ppm)

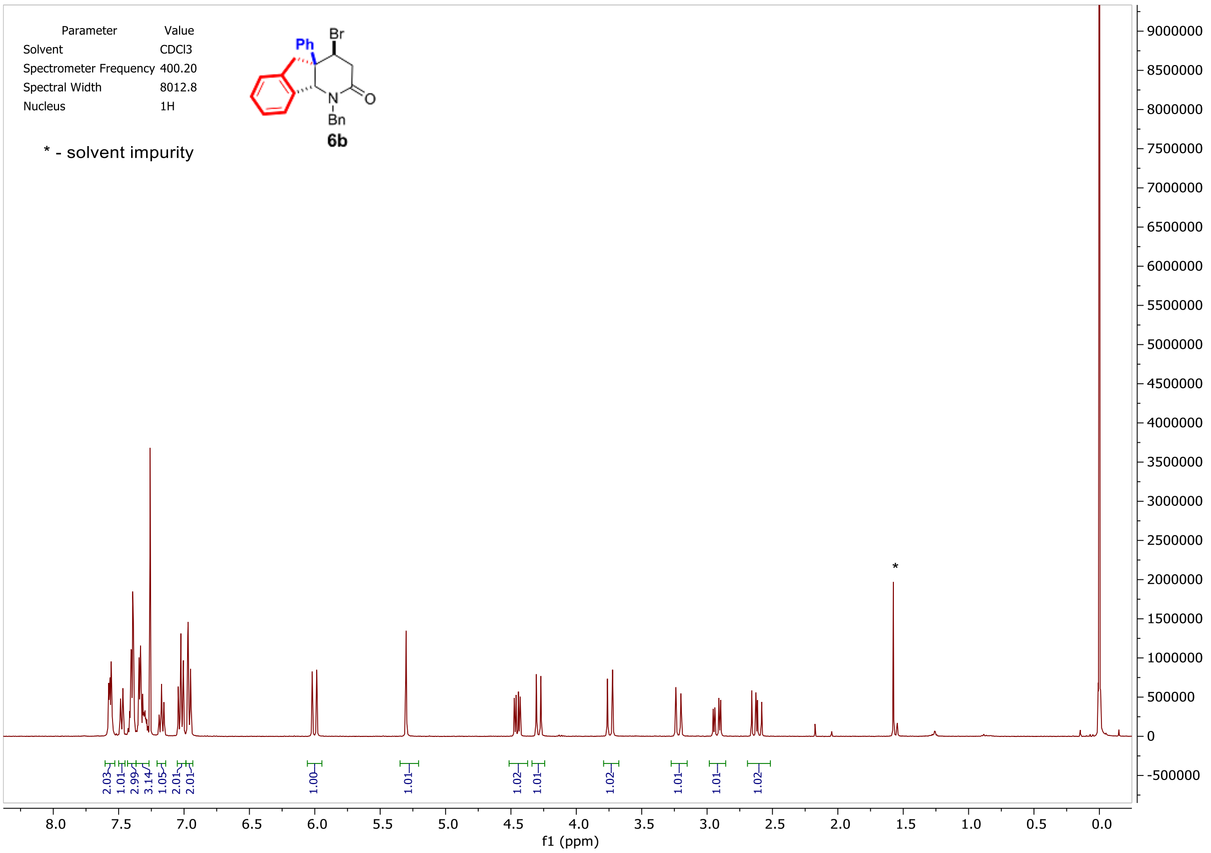
Parameter Value
Solvent CDCl3
Spectrometer Frequency 400.20
Spectral Width 8012.8
Nucleus 1H
* - solvent impurity
Ph
Br
N
O
Bn
6b
*
2.03 1.01 2.99 3.14 1.05 2.01 2.01 1.00 1.01 1.02 1.01 1.02 1.01 1.01 1.02
f1 (ppm)

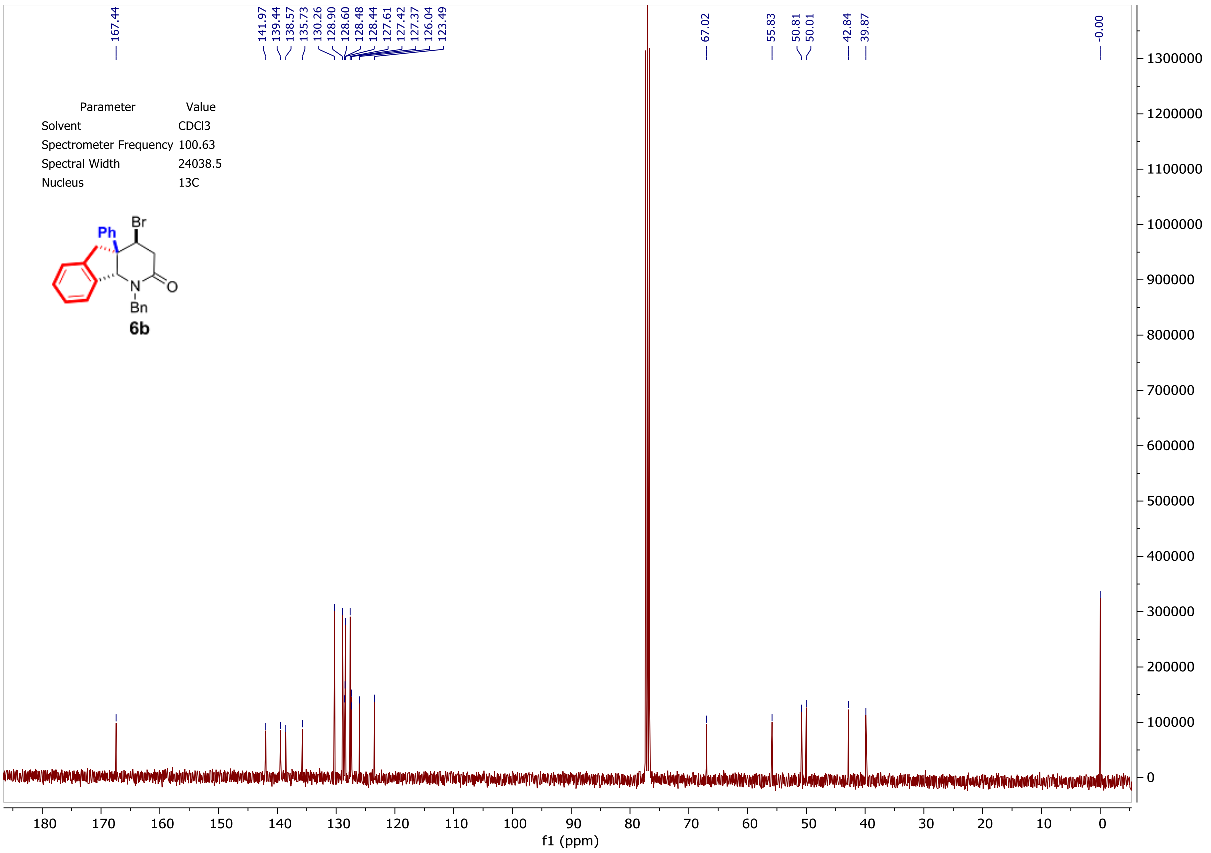
167.44
141.97 139.44 138.57 135.73 130.26 128.90 128.60 128.48 128.44 127.61 127.42 127.37 126.04 123.49
67.02
55.83 50.81 50.01
42.84 39.87
-0.00
Parameter Value
Solvent CDCl3
Spectrometer Frequency 100.63
Spectral Width 24038.5
Nucleus 13C
Ph
Br
N
O
Bn
6b
f1 (ppm)

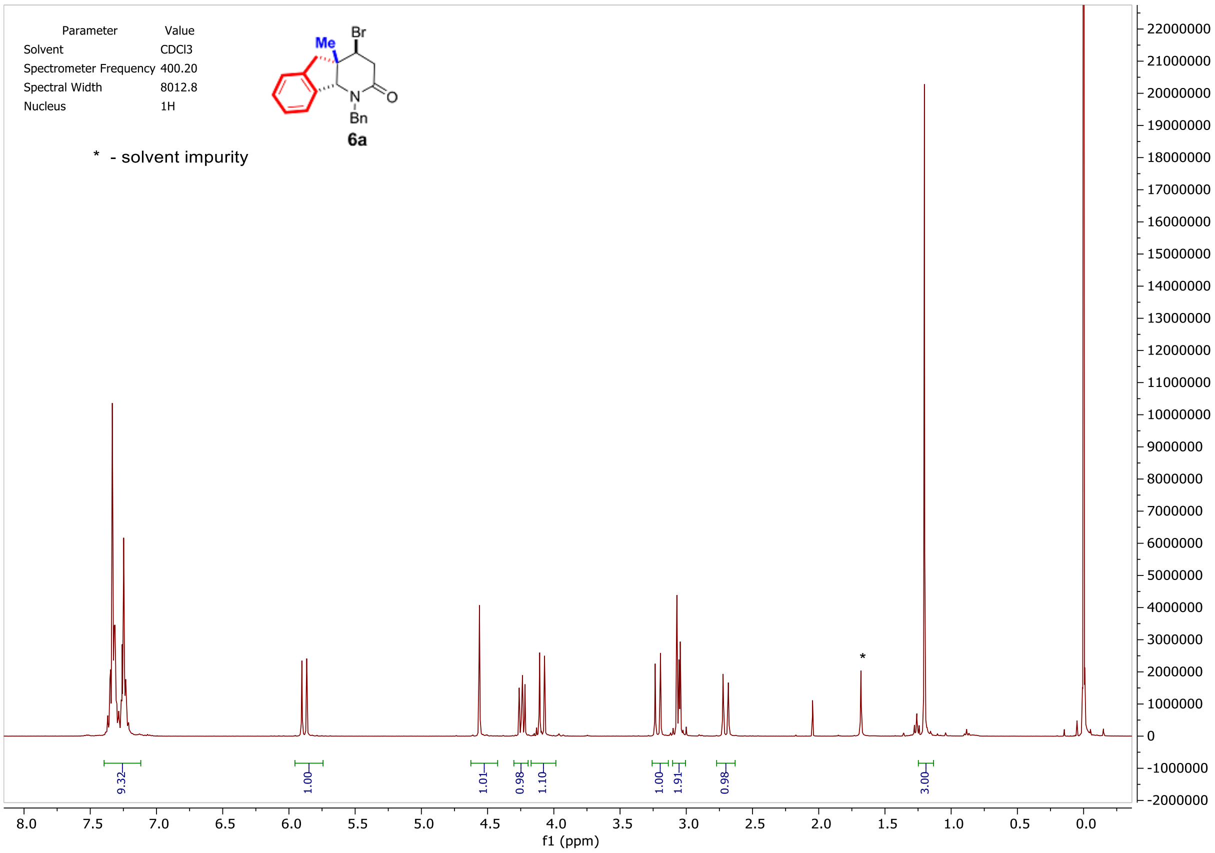
Parameter Value
Solvent CDCl3
Spectrometer Frequency 400.20
Spectral Width 8012.8
Nucleus 1H
Me
Br
N
O
Bn
6a
* - solvent impurity
9.32
1.00
1.01
0.98
1.10
1.00
1.91
0.98
3.00
f1 (ppm)

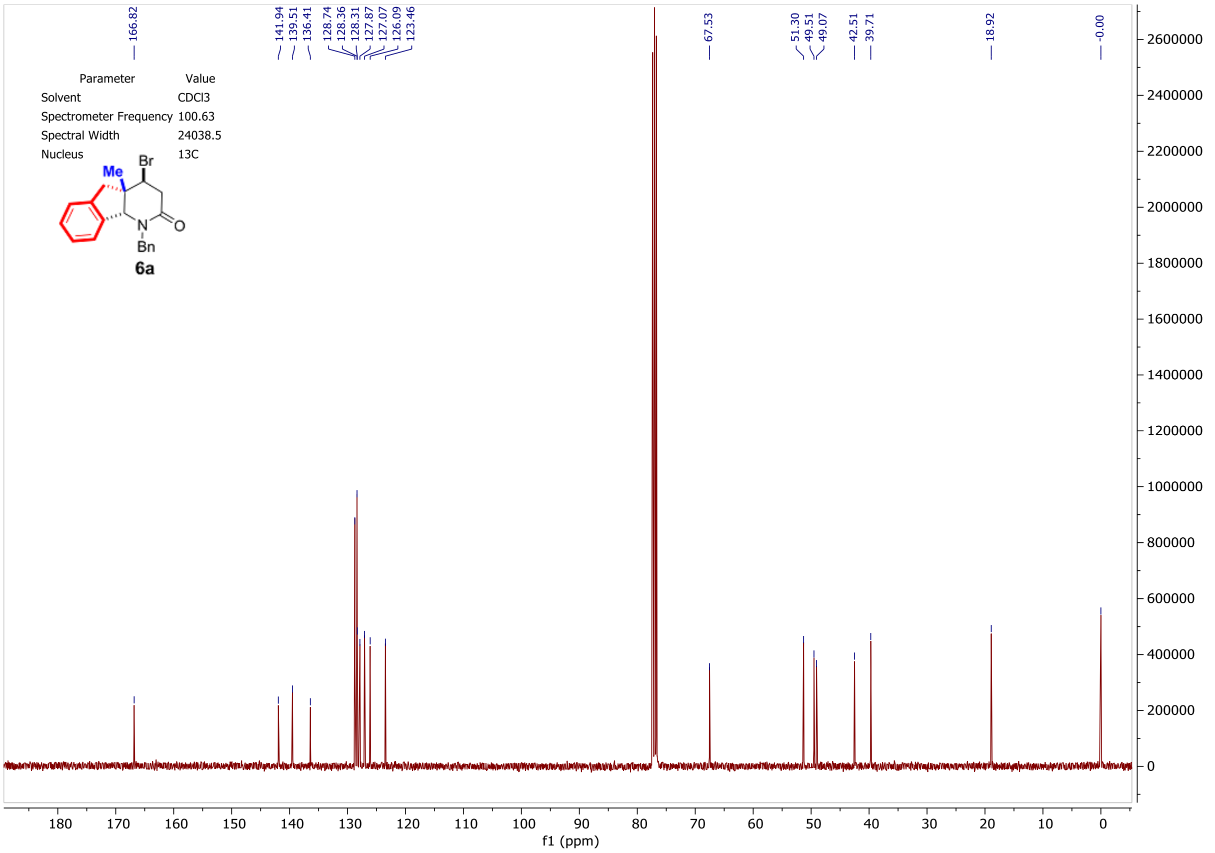
166.82
141.94
139.51
136.41
128.74
128.36
128.31
127.87
127.07
126.09
123.46
67.53
51.30
49.51
49.07
42.51
39.71
18.92
-0.00
Parameter Value
Solvent CDCl3
Spectrometer Frequency 100.63
Spectral Width 24038.5
Nucleus 13C
Me
Br
N
O
Bn
6a
f1 (ppm)

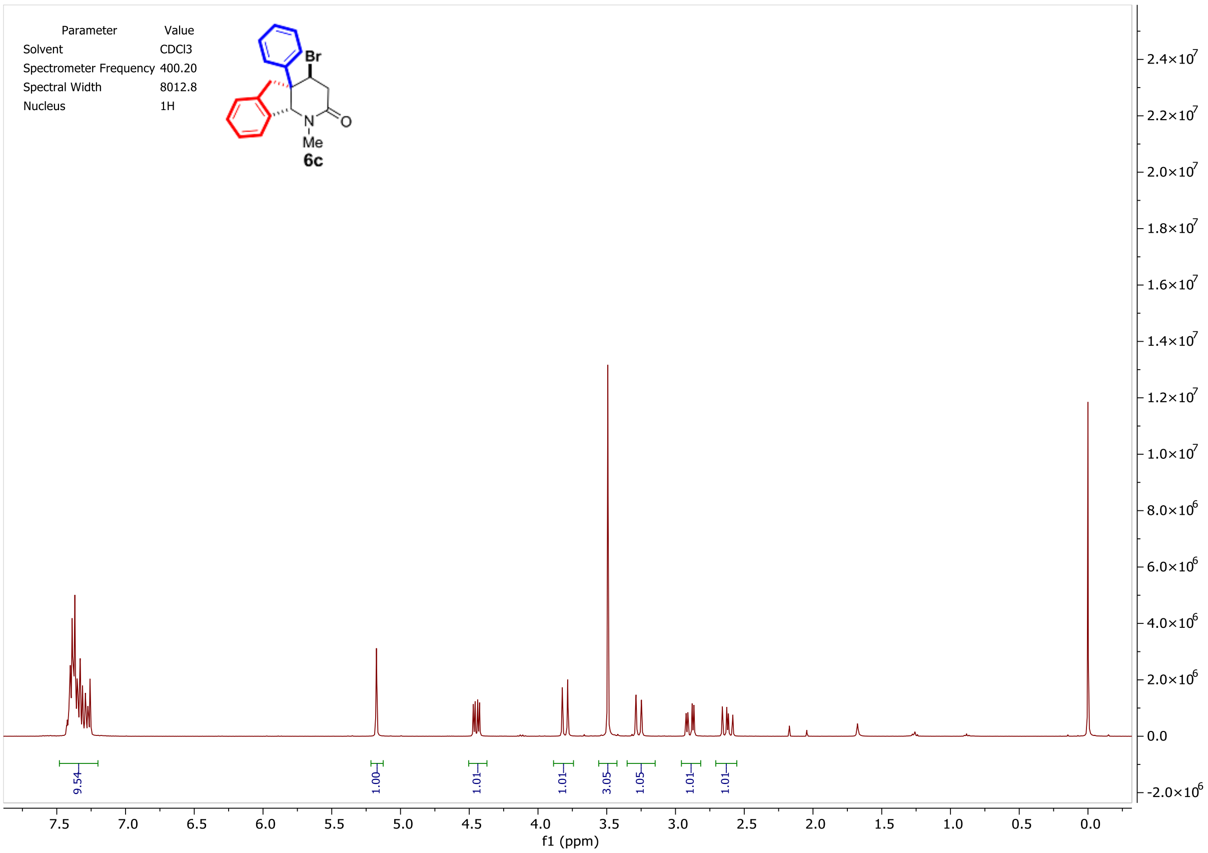
Parameter Value
Solvent CDCl3
Spectrometer Frequency 400.20
Spectral Width 8012.8
Nucleus 1H
Br
N
Me
O
6c
9.54
1.00
1.01
1.01
3.05
1.05
1.01
1.01
f1 (ppm)

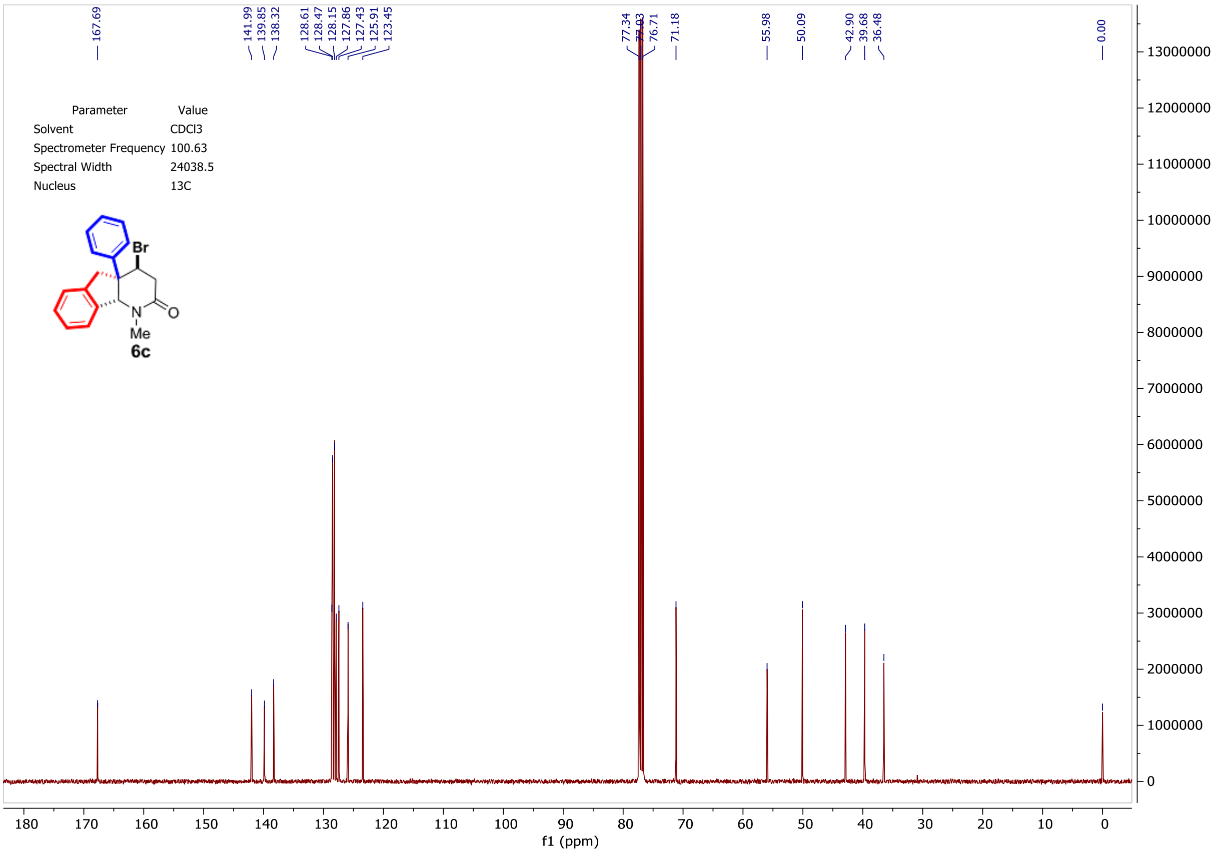
167.69
141.99
139.85
138.32
128.61
128.47
128.15
127.86
127.43
125.91
123.45
77.34
77.03
76.71
71.18
55.98
50.09
42.90
39.68
36.48
0.00
Parameter Value
Solvent CDCl3
Spectrometer Frequency 100.63
Spectral Width 24038.5
Nucleus 13C
Br
N
Me
O
6c
f1 (ppm)

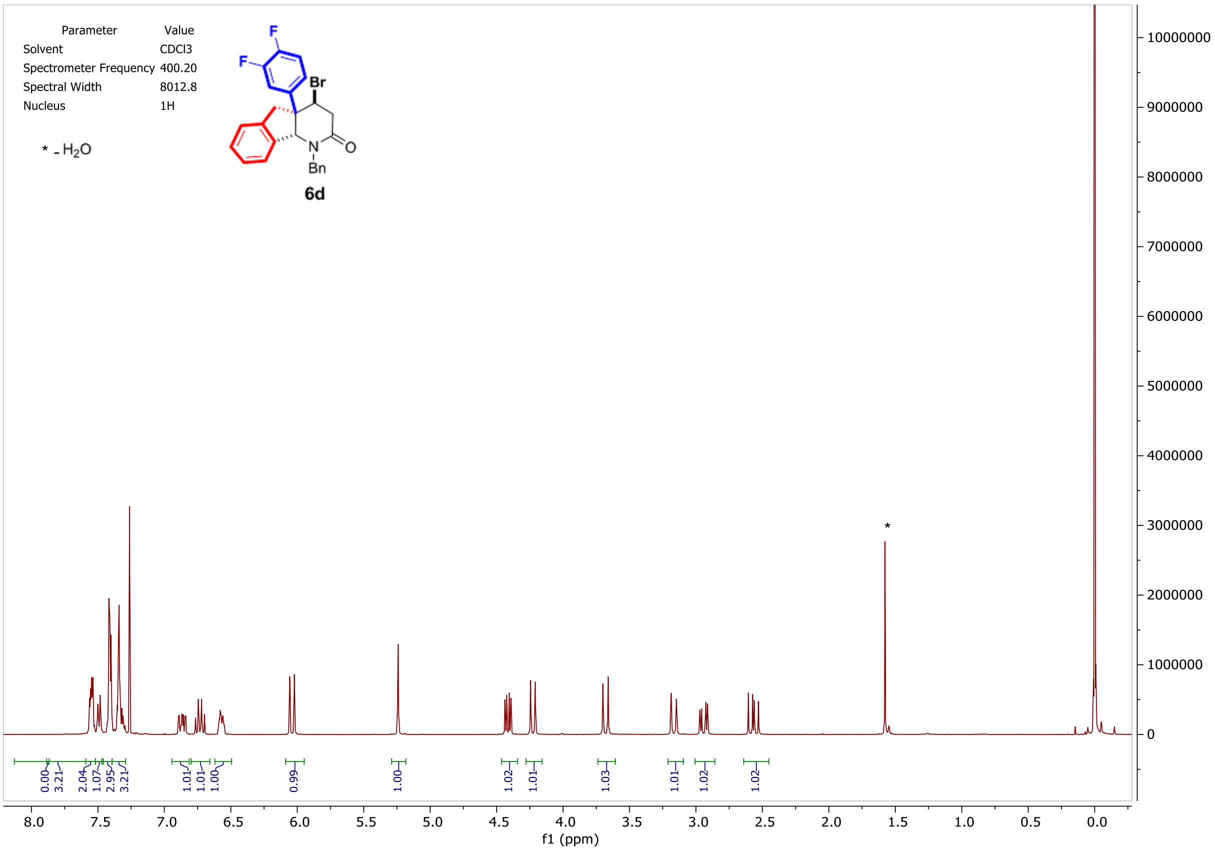
Parameter Value
Solvent CDCl3
Spectrometer Frequency 400.20
Spectral Width 8012.8
Nucleus 1H
* - H2O
F
F
Br
N
O
Bn
6d
*
0.00
3.21
2.04
1.07
2.95
3.21
1.01
1.01
1.00
0.99
1.00
1.02
1.01
1.03
1.01
1.02
1.02
f1 (ppm)

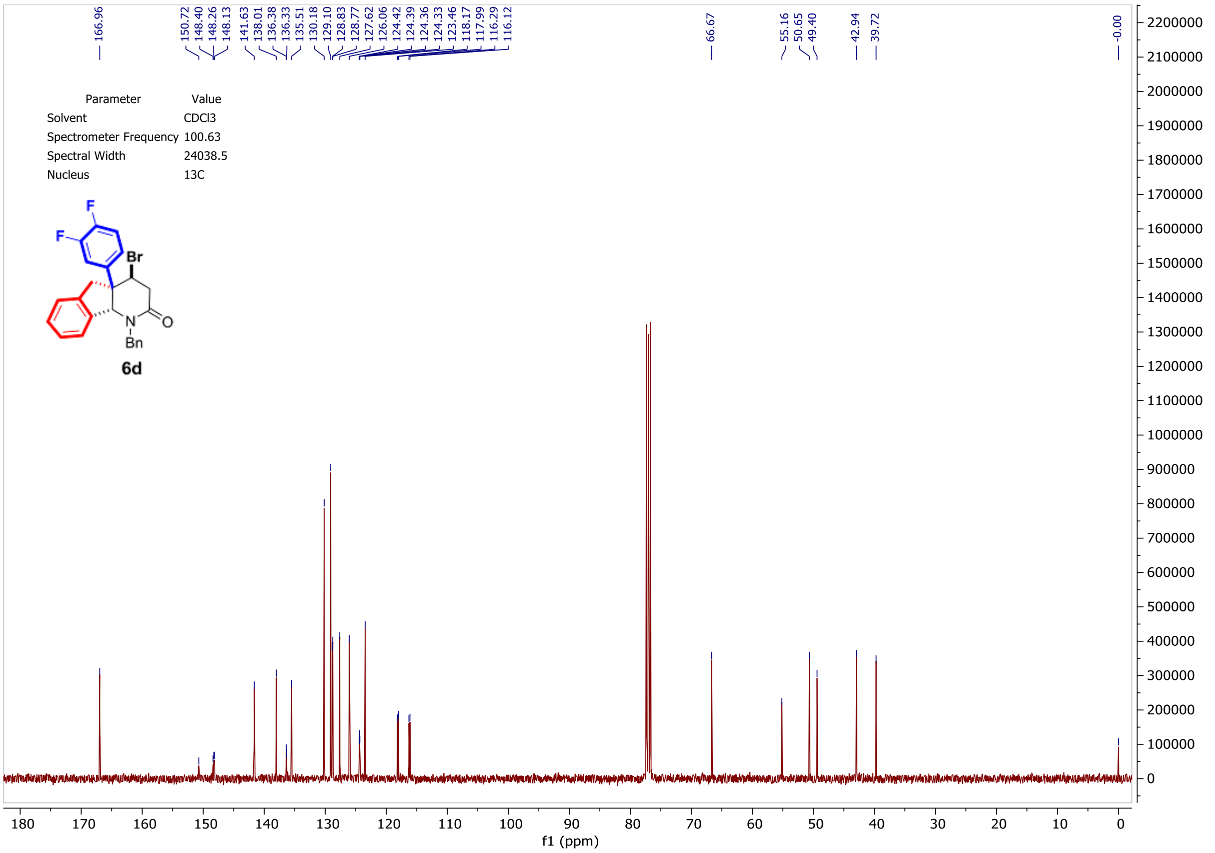
166.96
150.72
148.40
148.26
148.13
141.63
138.01
136.38
136.33
135.51
130.18
129.10
128.83
128.77
127.62
126.06
124.42
124.39
124.36
124.33
123.46
118.17
117.99
116.29
116.12
66.67
55.16
50.65
49.40
42.94
39.72
-0.00
Parameter Value
Solvent CDCl3
Spectrometer Frequency 100.63
Spectral Width 24038.5
Nucleus 13C
F
F
Br
N
O
Bn
6d
f1 (ppm)

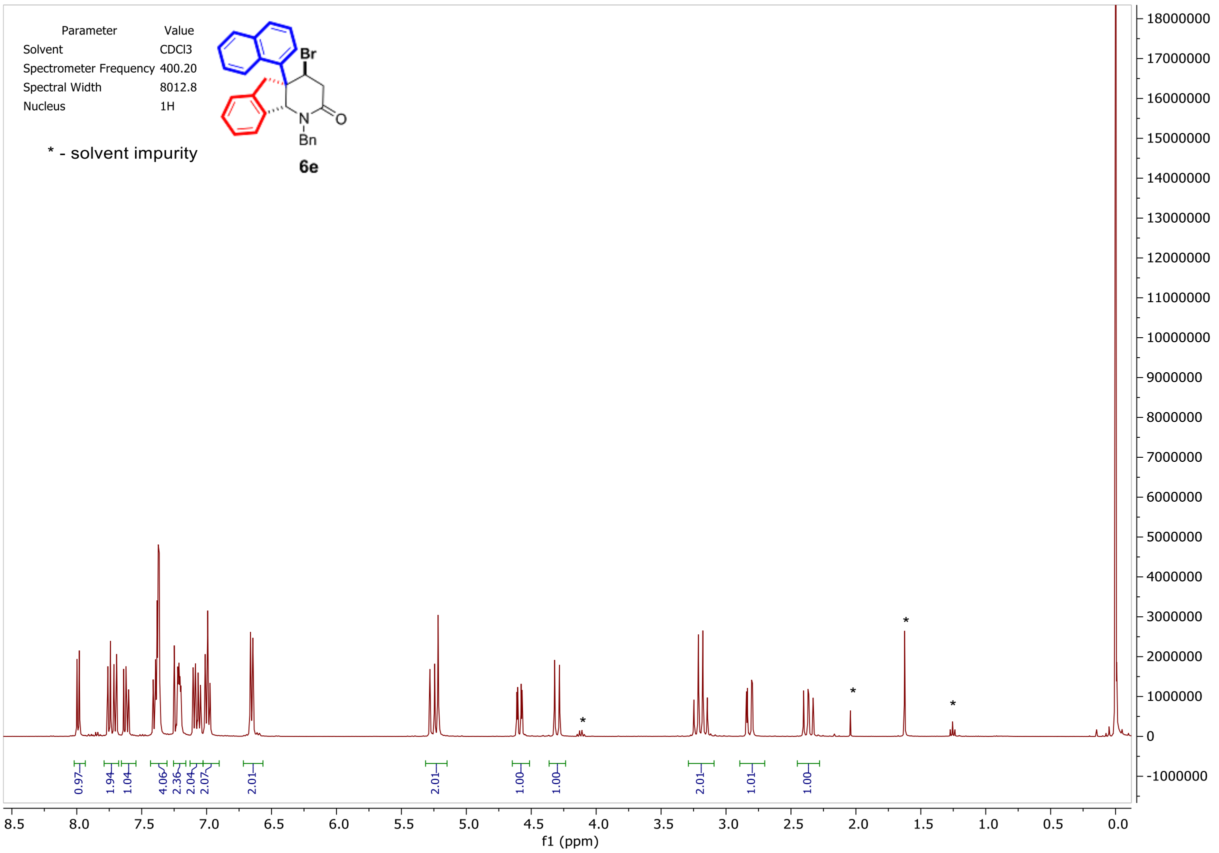
Parameter Value
Solvent CDCl3
Spectrometer Frequency 400.20
Spectral Width 8012.8
Nucleus 1H
* - solvent impurity
Br
N
O
Bn
6e
0.97
1.94
1.04
4.06
2.36
2.04
2.07
2.01
2.01
1.00
1.00
2.01
1.01
1.00
f1 (ppm)

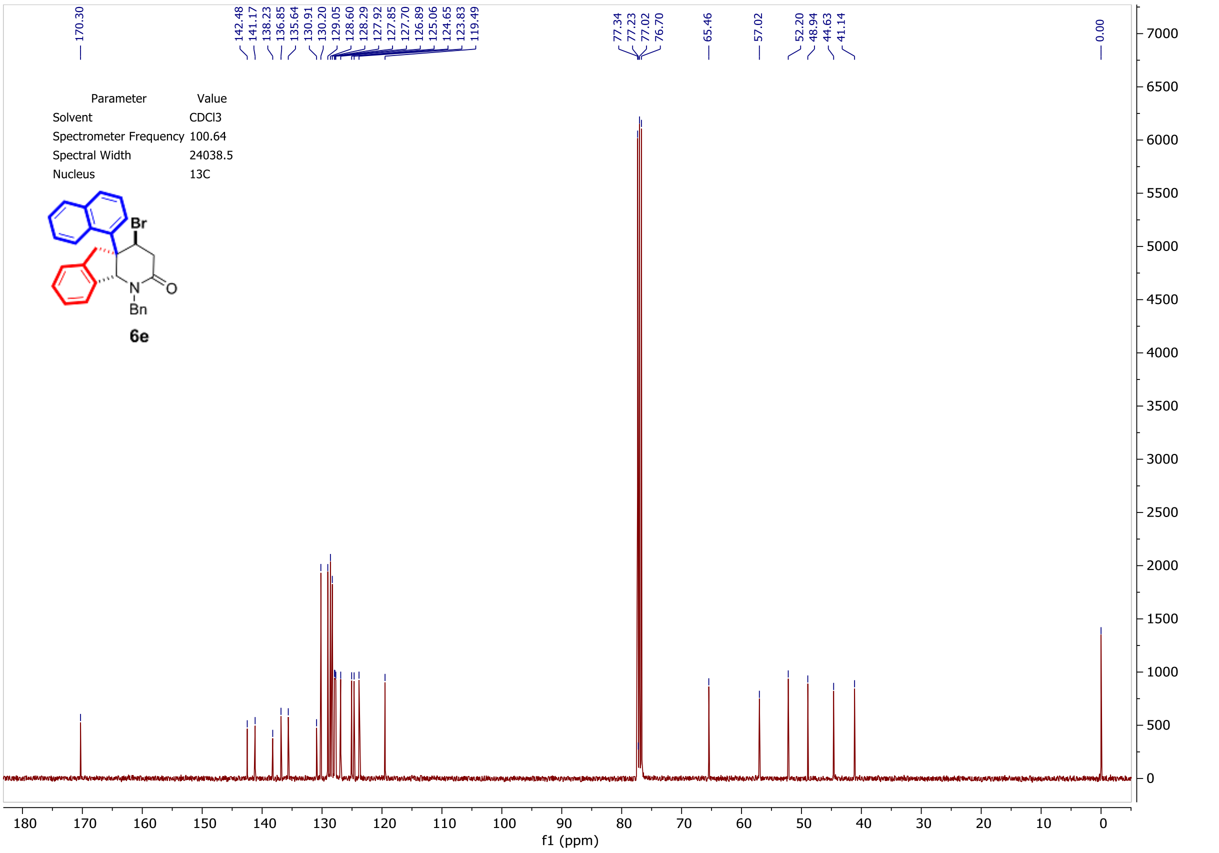
170.30
142.48
141.17
138.23
136.85
135.64
130.91
130.20
129.05
128.60
128.29
127.92
127.85
127.70
126.89
125.06
124.65
123.83
119.49
77.34
77.23
77.02
76.70
65.46
57.02
52.20
48.94
44.63
41.14
0.00
Parameter Value
Solvent CDCl3
Spectrometer Frequency 100.64
Spectral Width 24038.5
Nucleus 13C
Br
N
O
Bn
6e
f1 (ppm)

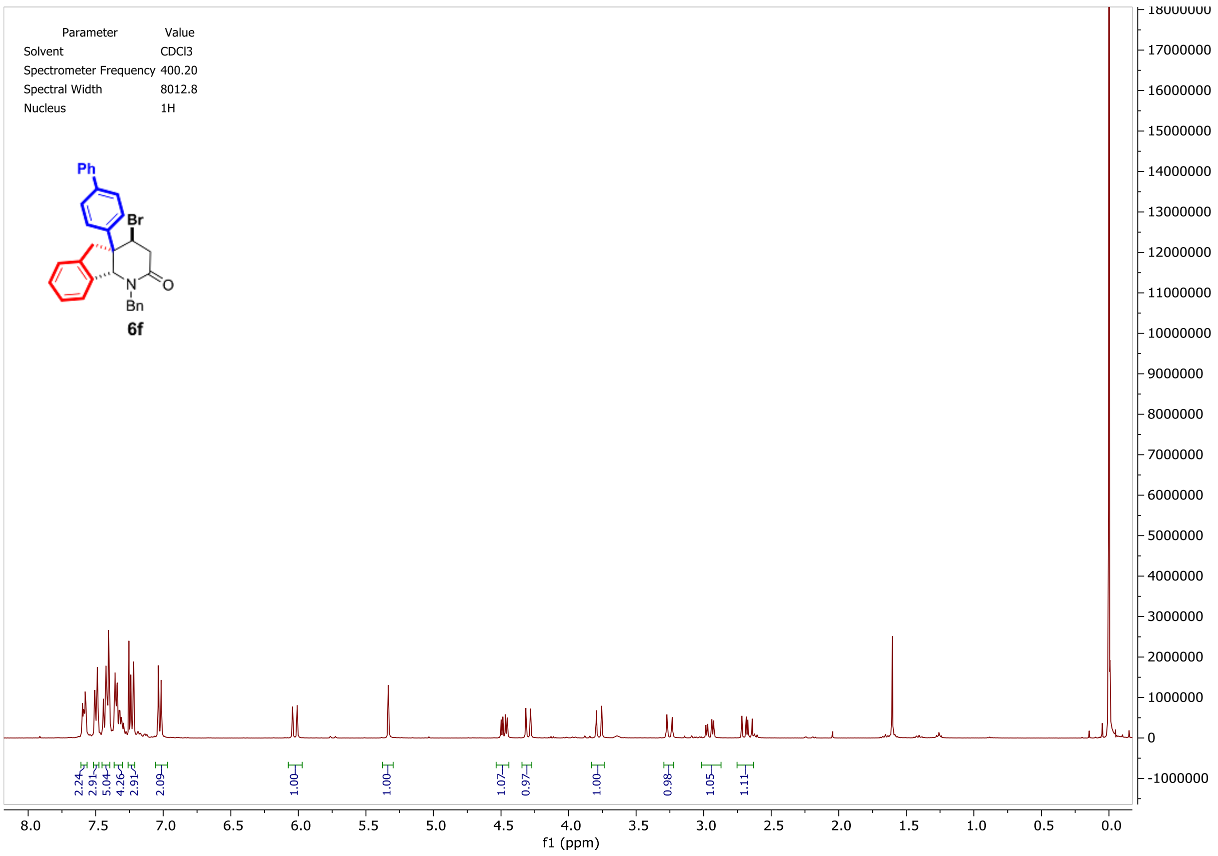
Parameter Value
Solvent CDCl3
Spectrometer Frequency 400.20
Spectral Width 8012.8
Nucleus 1H
Ph
Br
N
O
Bn
6f
2.24
2.91
5.04
4.26
2.91
2.09
1.00
1.00
1.07
0.97
1.00
0.98
1.05
1.11
f1 (ppm)

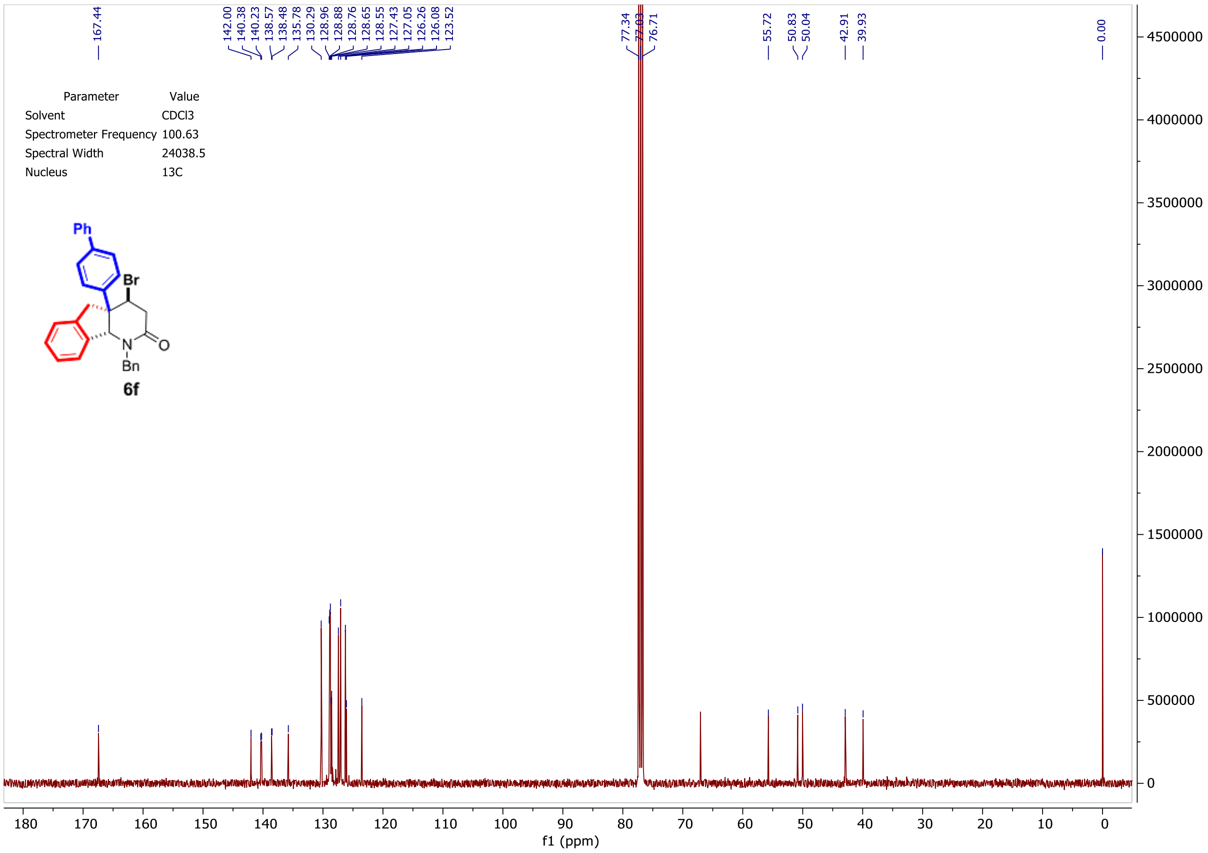
167.44
142.00
140.38
140.23
138.57
138.48
135.78
130.29
128.96
128.88
128.76
128.65
128.55
127.43
127.05
126.26
126.08
123.52
77.34
77.03
76.71
55.72
50.83
50.04
42.91
39.93
0.00
Parameter Value
Solvent CDCl3
Spectrometer Frequency 100.63
Spectral Width 24038.5
Nucleus 13C
Ph
Br
N
O
Bn
6f
f1 (ppm)

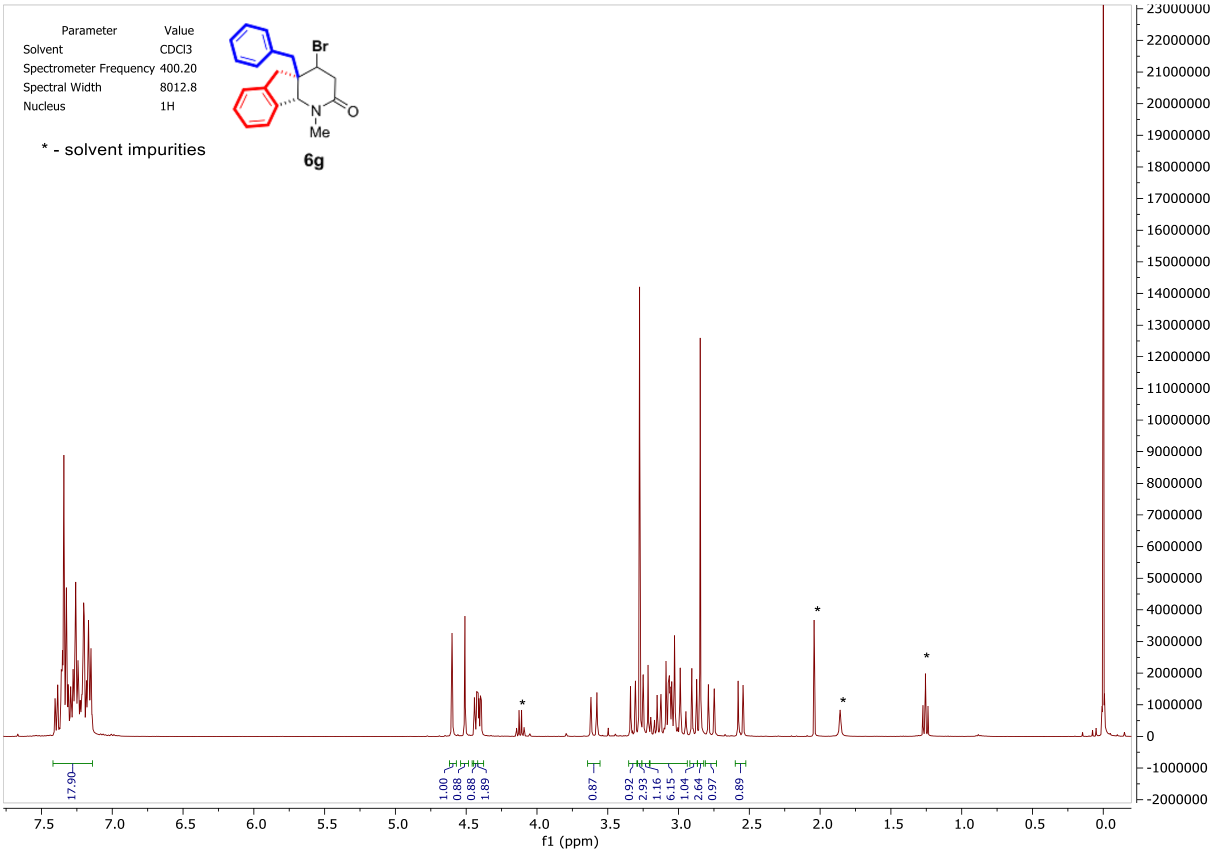
Parameter Value
Solvent CDCl3
Spectrometer Frequency 400.20
Spectral Width 8012.8
Nucleus 1H
* - solvent impurities
Br
N
Me
O
6g
17.90
1.00
0.88
0.88
1.89
0.87
0.92
2.93
1.16
6.15
1.04
2.64
0.97
0.89
f1 (ppm)

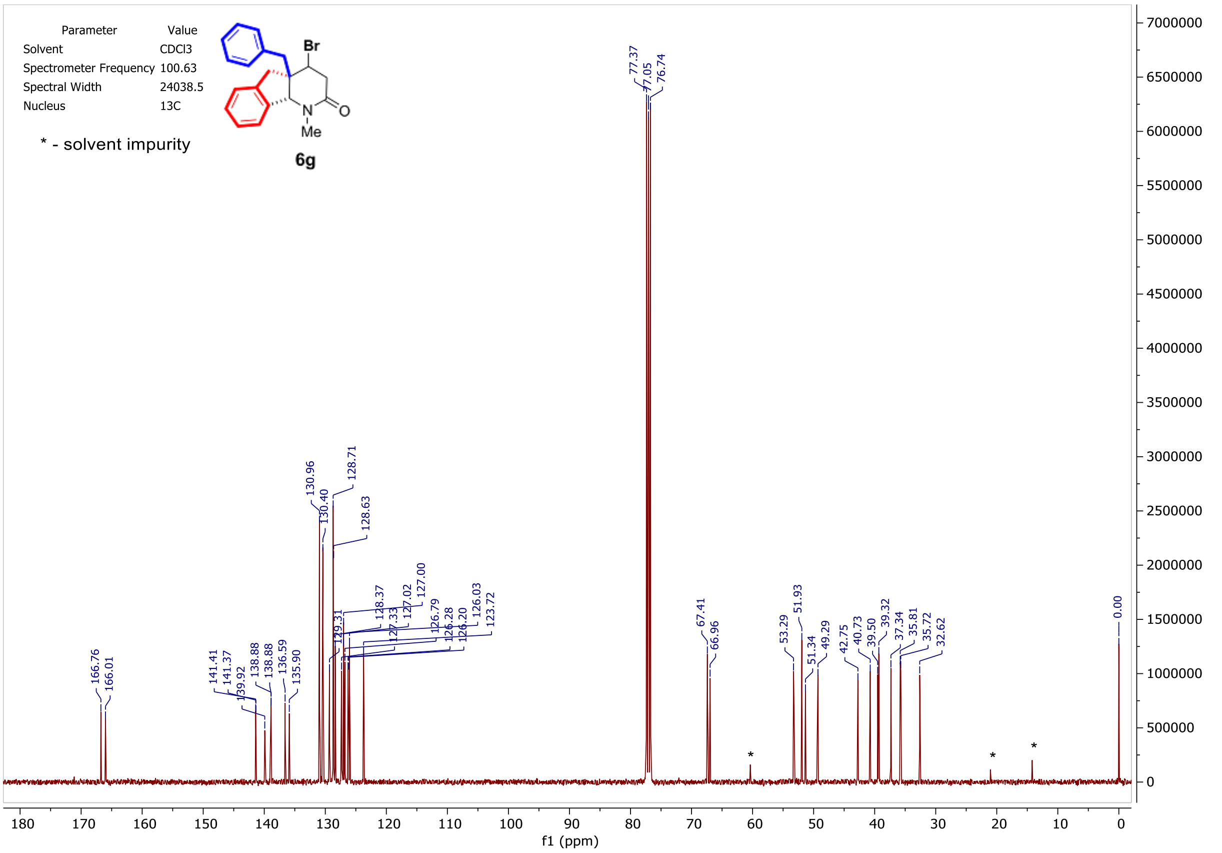
Parameter Value
Solvent CDCl3
Spectrometer Frequency 100.63
Spectral Width 24038.5
Nucleus 13C
* - solvent impurity
Br
N
Me
O
6g
166.76
166.01
141.41
141.37
139.92
138.88
138.88
136.59
135.90
130.96
130.40
129.31
128.71
128.63
128.37
127.33
127.02
127.00
126.79
126.28
126.20
126.03
123.72
77.37
77.05
76.74
67.41
66.96
53.29
51.93
51.34
49.29
42.75
40.73
39.50
39.32
37.34
35.81
35.72
32.62
0.00
f1 (ppm)

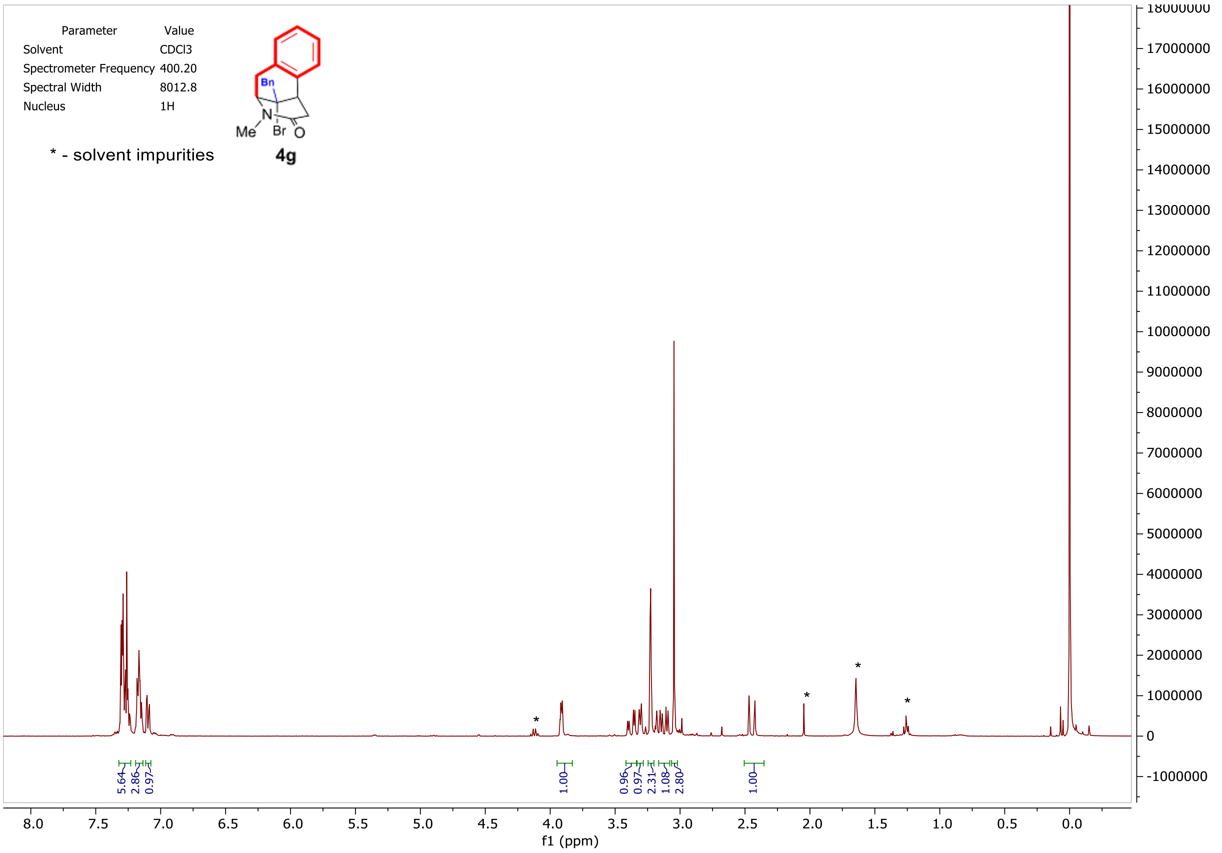

Parameter Value
Solvent CDCl3
Spectrometer Frequency 400.20
Spectral Width 8012.8
Nucleus 1H
* - solvent impurities
4g
f1 (ppm)

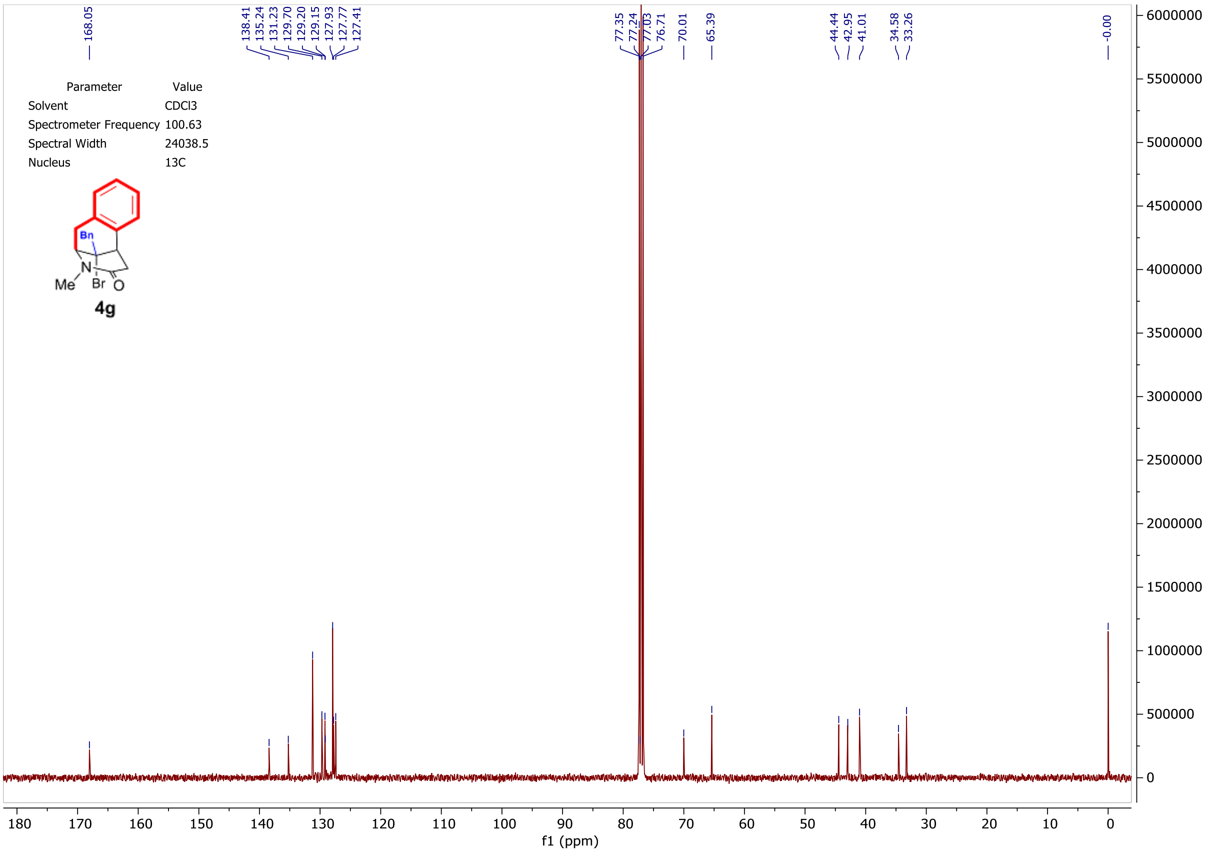

Parameter Value
Solvent CDCl3
Spectrometer Frequency 100.63
Spectral Width 24038.5
Nucleus 13C
4g
168.05
138.41
135.24
131.23
129.70
129.20
129.15
127.93
127.77
127.41
77.35
77.24
77.03
76.71
70.01
65.39
44.44
42.95
41.01
34.58
33.26
-0.00
f1 (ppm)

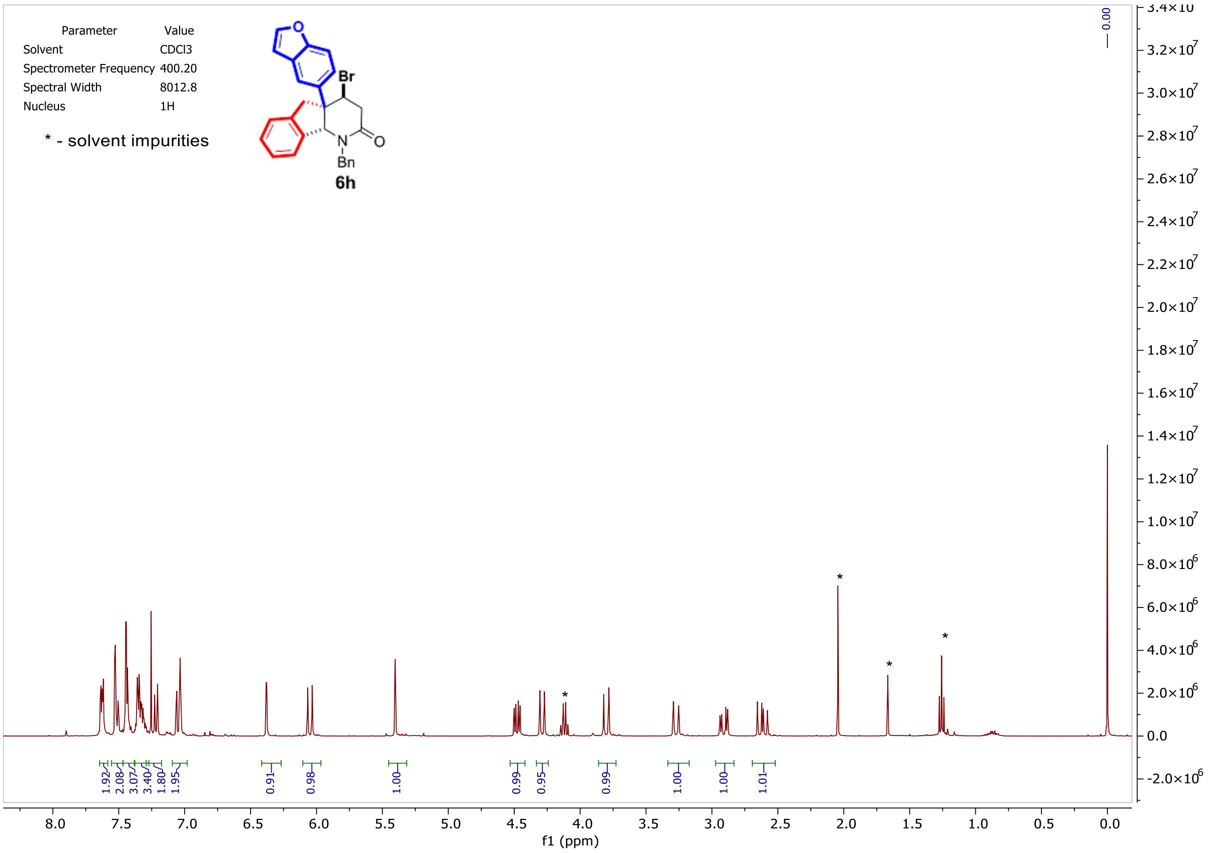
Parameter Value
Solvent CDCl3
Spectrometer Frequency 400.20
Spectral Width 8012.8
Nucleus 1H
* - solvent impurities
6h
f1 (ppm)

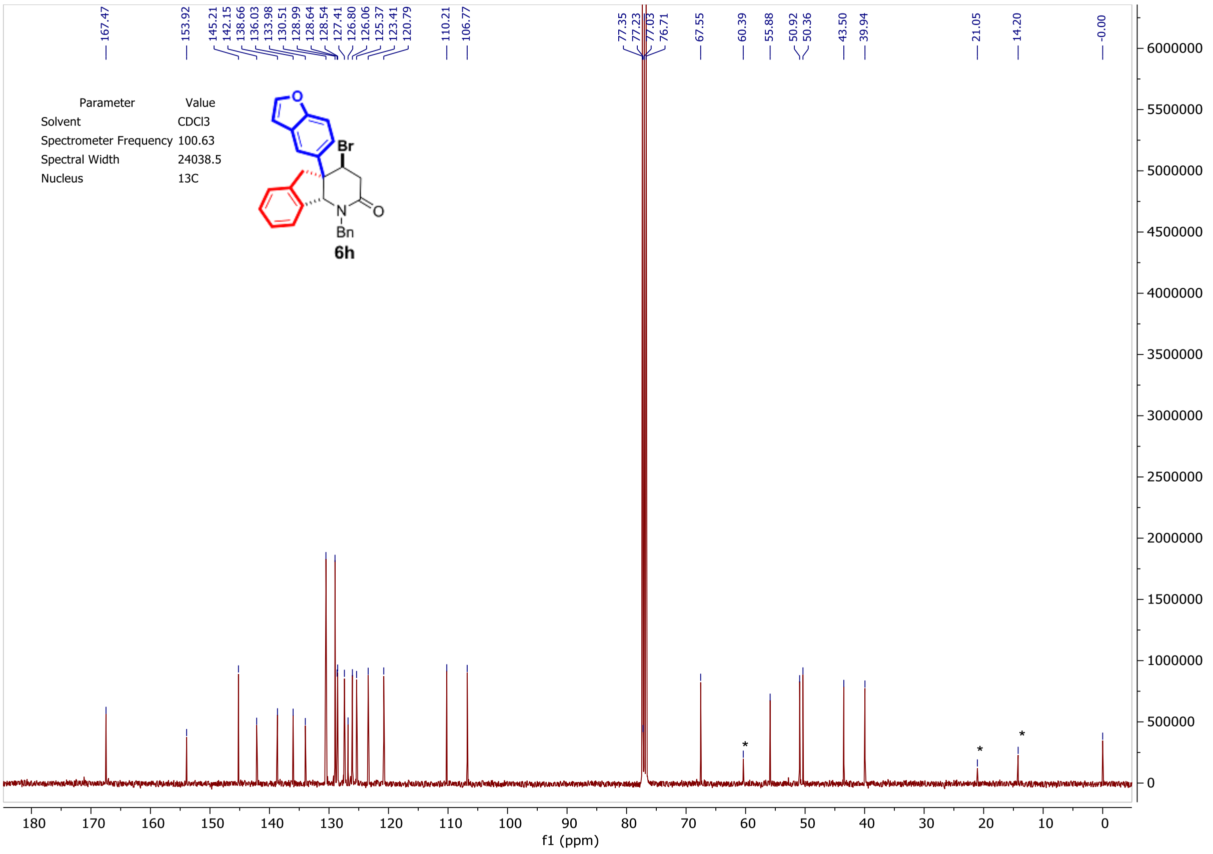
Parameter Value
Solvent CDCl3
Spectrometer Frequency 100.63
Spectral Width 24038.5
Nucleus 13C
6h
f1 (ppm)

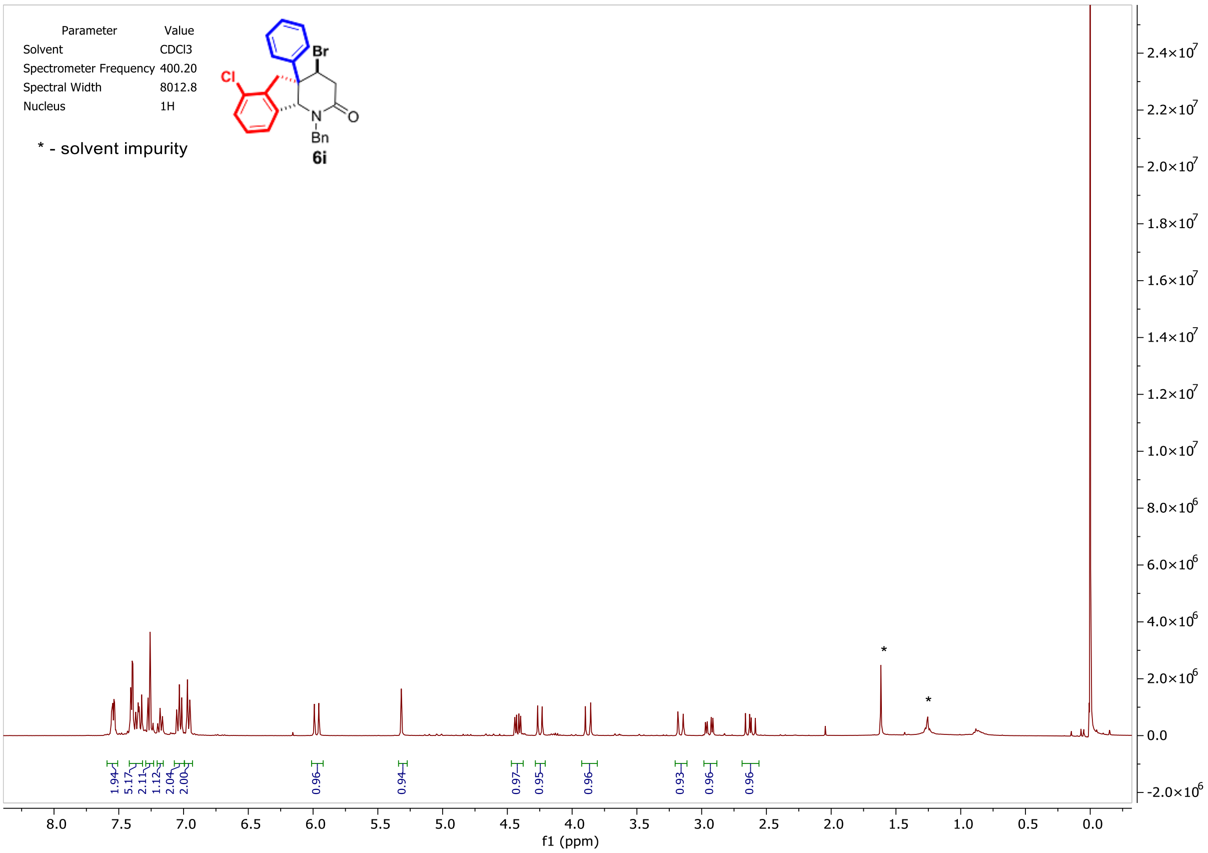
Parameter Value
Solvent CDCl3
Spectrometer Frequency 400.20
Spectral Width 8012.8
Nucleus 1H
* - solvent impurity
Br
Cl
N
O
Bn
6i
1.94
5.17
2.11
1.12
2.04
2.00
0.96
0.94
0.97
0.95
0.96
0.93
0.96
0.96
f1 (ppm)

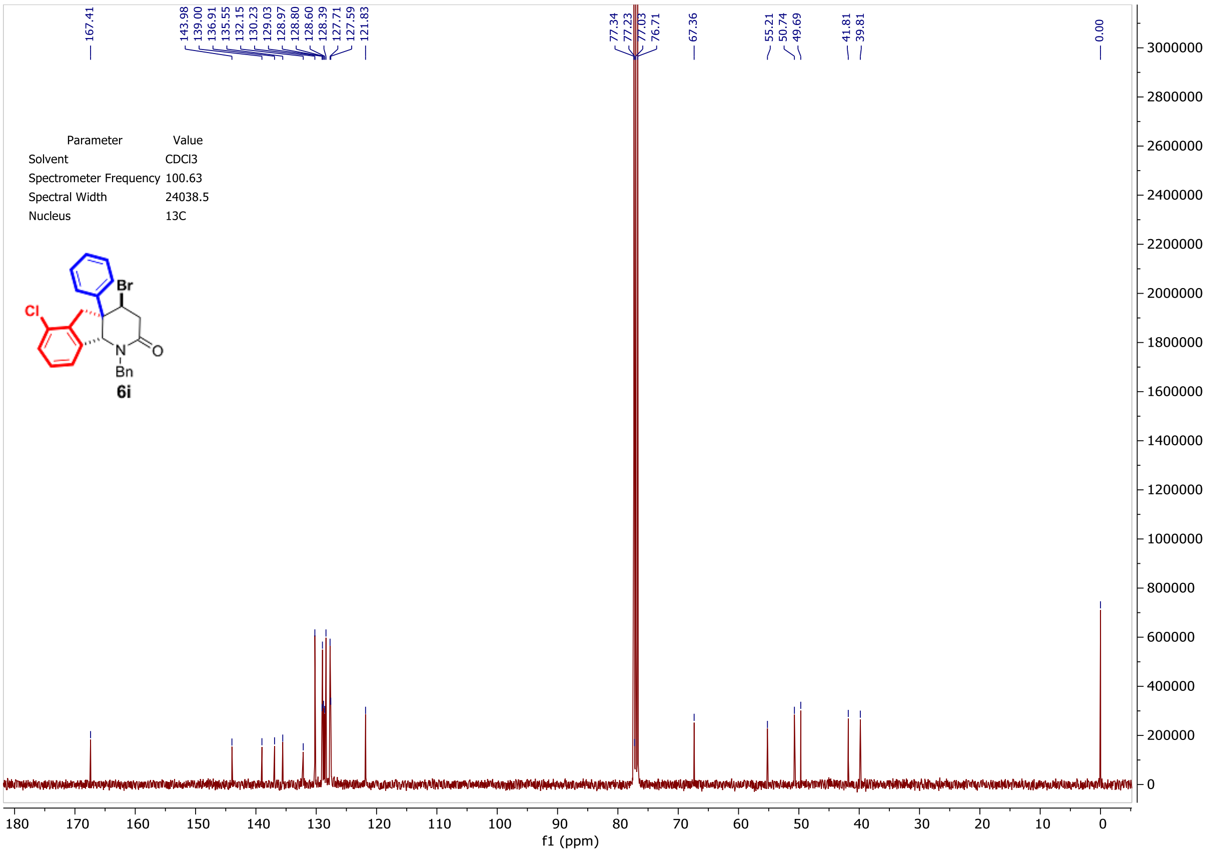
167.41
143.98
139.00
136.91
135.55
132.15
130.23
129.03
128.97
128.80
128.60
128.39
127.71
127.59
121.83
77.34
77.23
77.03
76.71
67.36
55.21
50.74
49.69
41.81
39.81
0.00
Parameter Value
Solvent CDCl3
Spectrometer Frequency 100.63
Spectral Width 24038.5
Nucleus 13C
Br
Cl
N
O
Bn
6i
f1 (ppm)

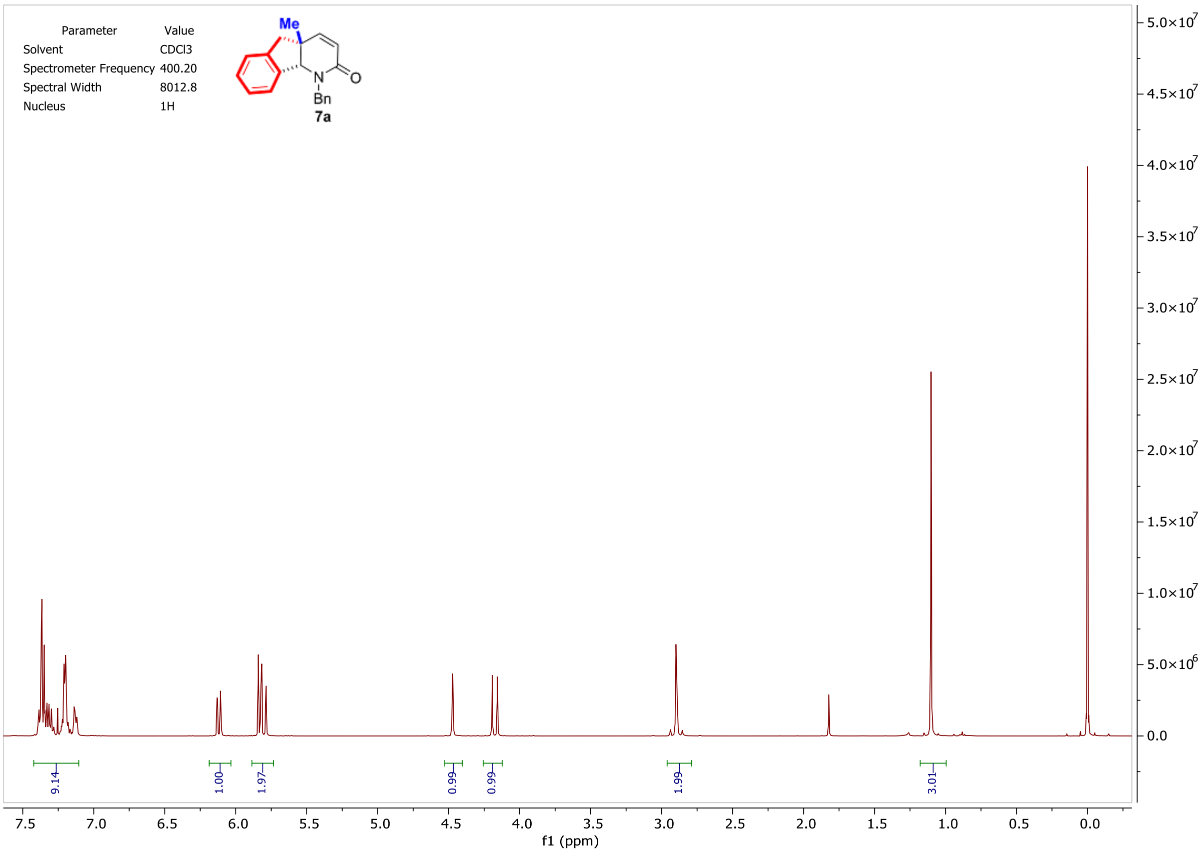

Parameter Value
Solvent CDCl3
Spectrometer Frequency 400.20
Spectral Width 8012.8
Nucleus 1H
Me
N
O
Bn
7a
9.14
1.00
1.97
0.99
0.99
1.99
3.01
f1 (ppm)

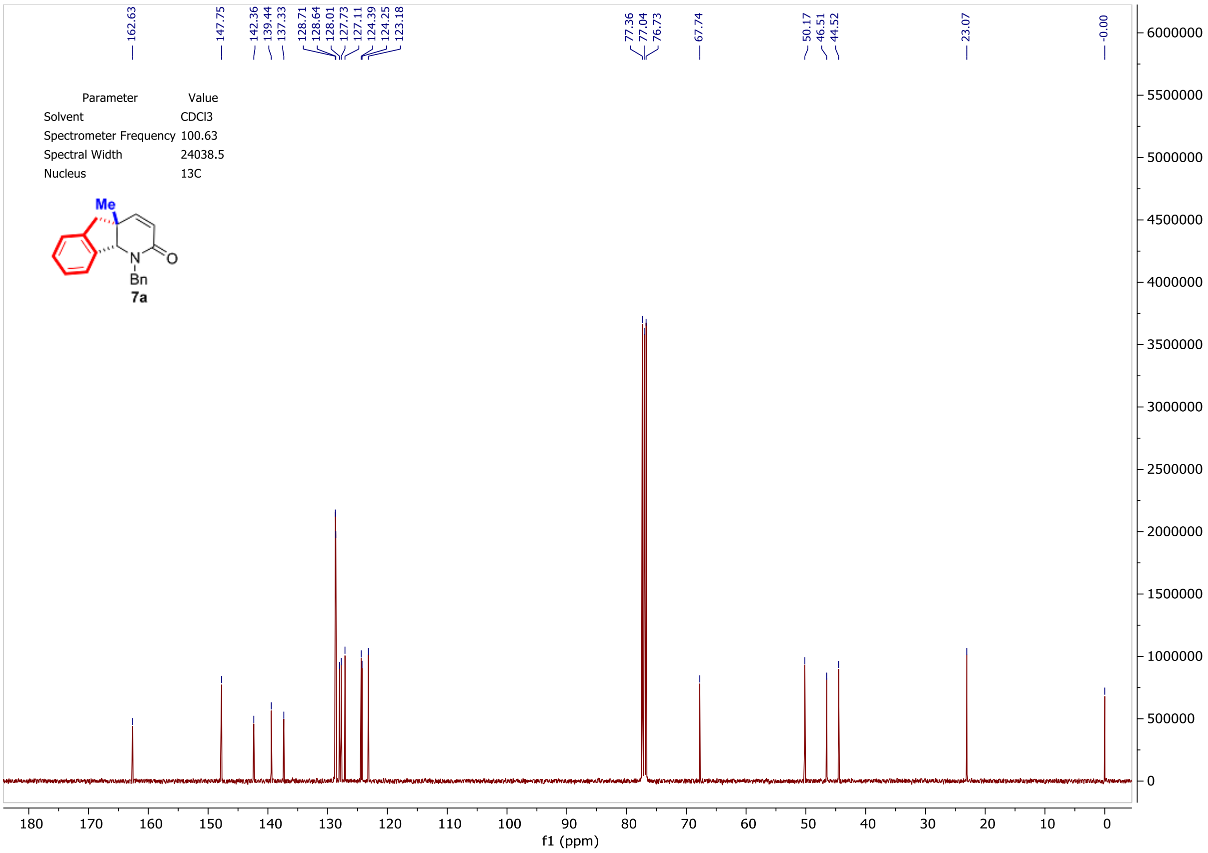

162.63
147.75
142.36
139.44
137.33
128.71
128.64
128.01
127.73
127.11
124.39
124.25
123.18
77.36
77.04
76.73
67.74
50.17
46.51
44.52
23.07
-0.00
Parameter Value
Solvent CDCl3
Spectrometer Frequency 100.63
Spectral Width 24038.5
Nucleus 13C
Me
N
O
Bn
7a
f1 (ppm)

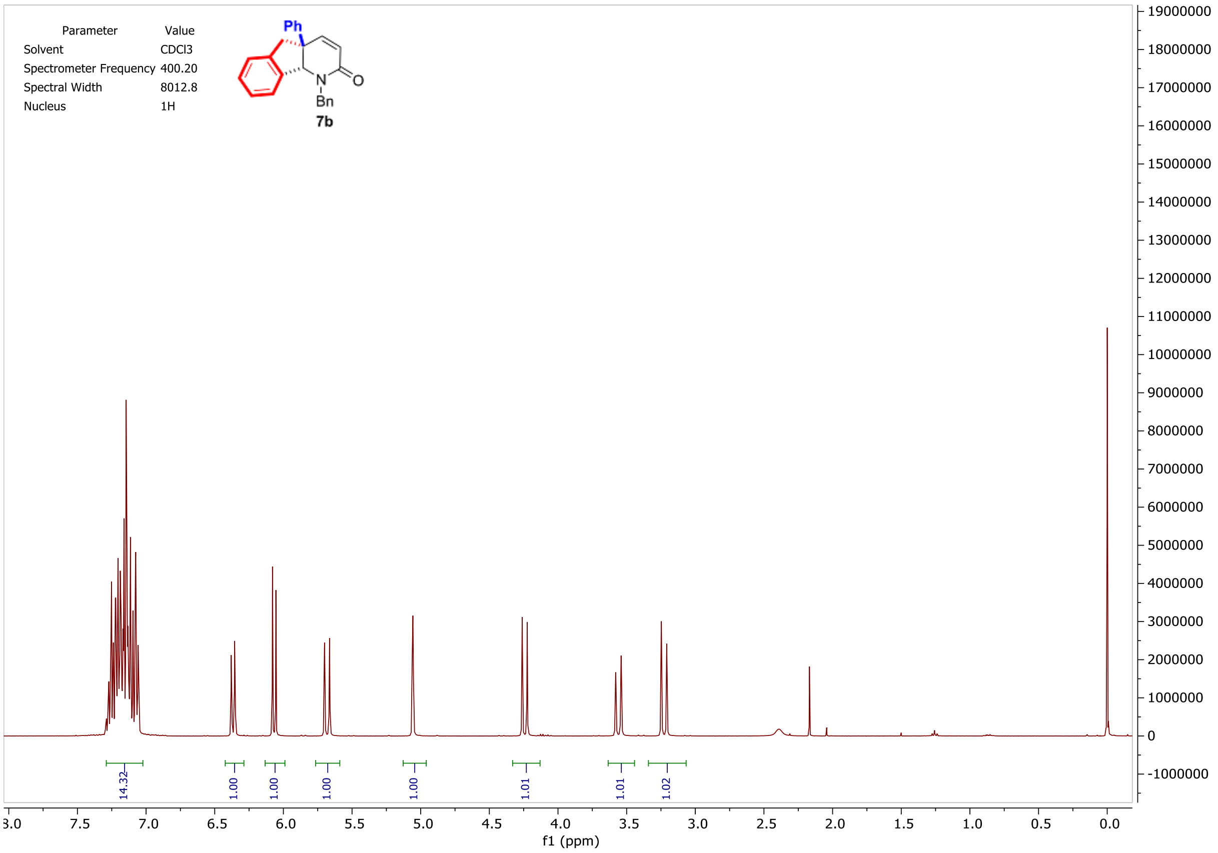

Parameter Value
Solvent CDCl3
Spectrometer Frequency 400.20
Spectral Width 8012.8
Nucleus 1H
Ph
N
O
Bn
7b
f1 (ppm)

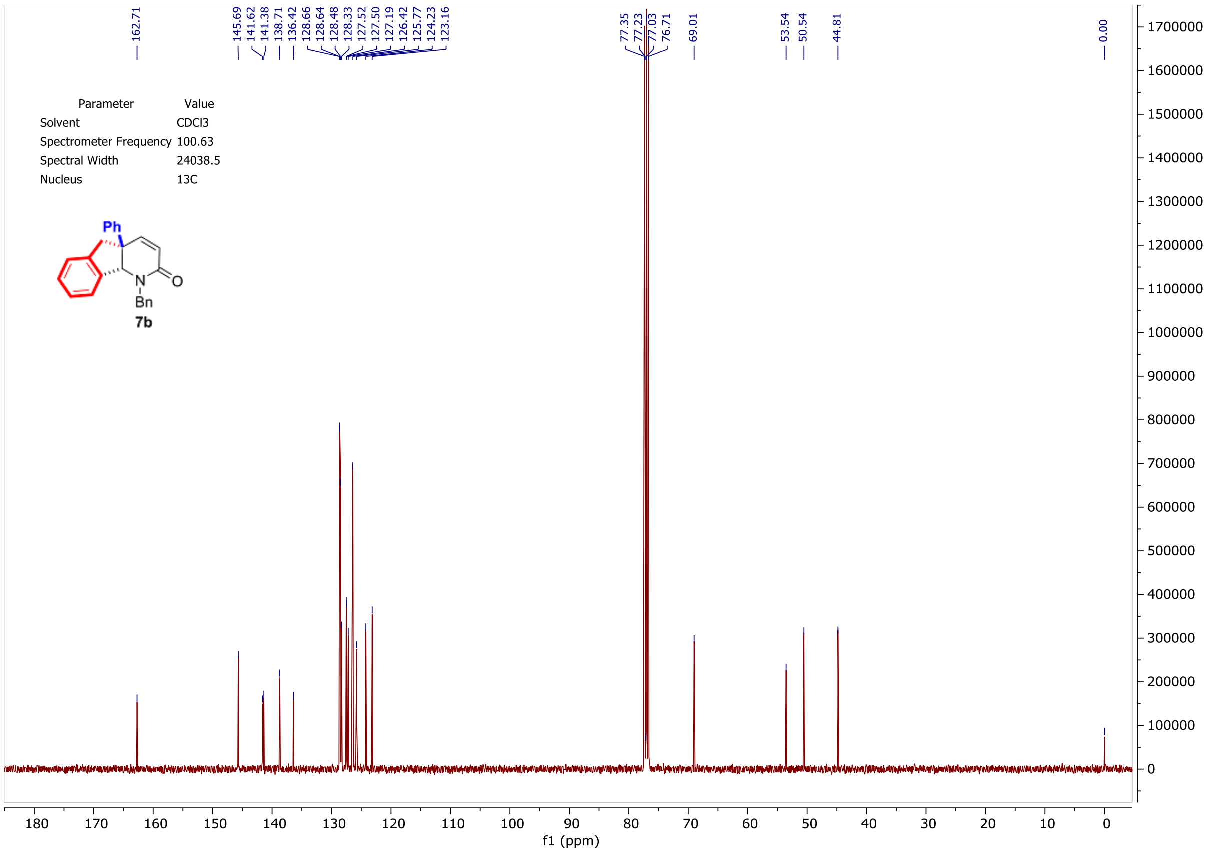

Parameter Value
Solvent CDCl3
Spectrometer Frequency 100.63
Spectral Width 24038.5
Nucleus 13C
Ph
N
O
Bn
7b
f1 (ppm)

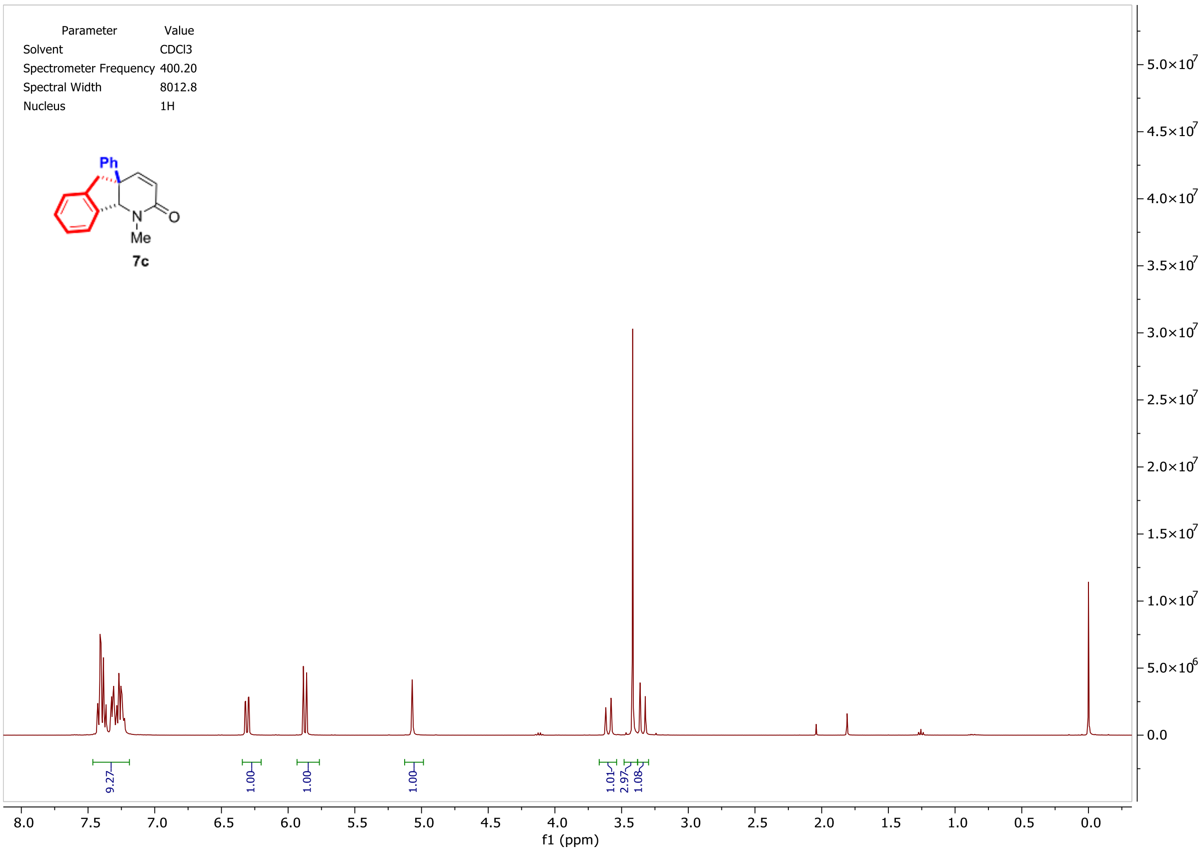
Parameter Value
Solvent CDCl3
Spectrometer Frequency 400.20
Spectral Width 8012.8
Nucleus 1H
Ph
N
O
Me
7c
9.27
1.00
1.00
1.00
1.01
2.97
1.08
f1 (ppm)

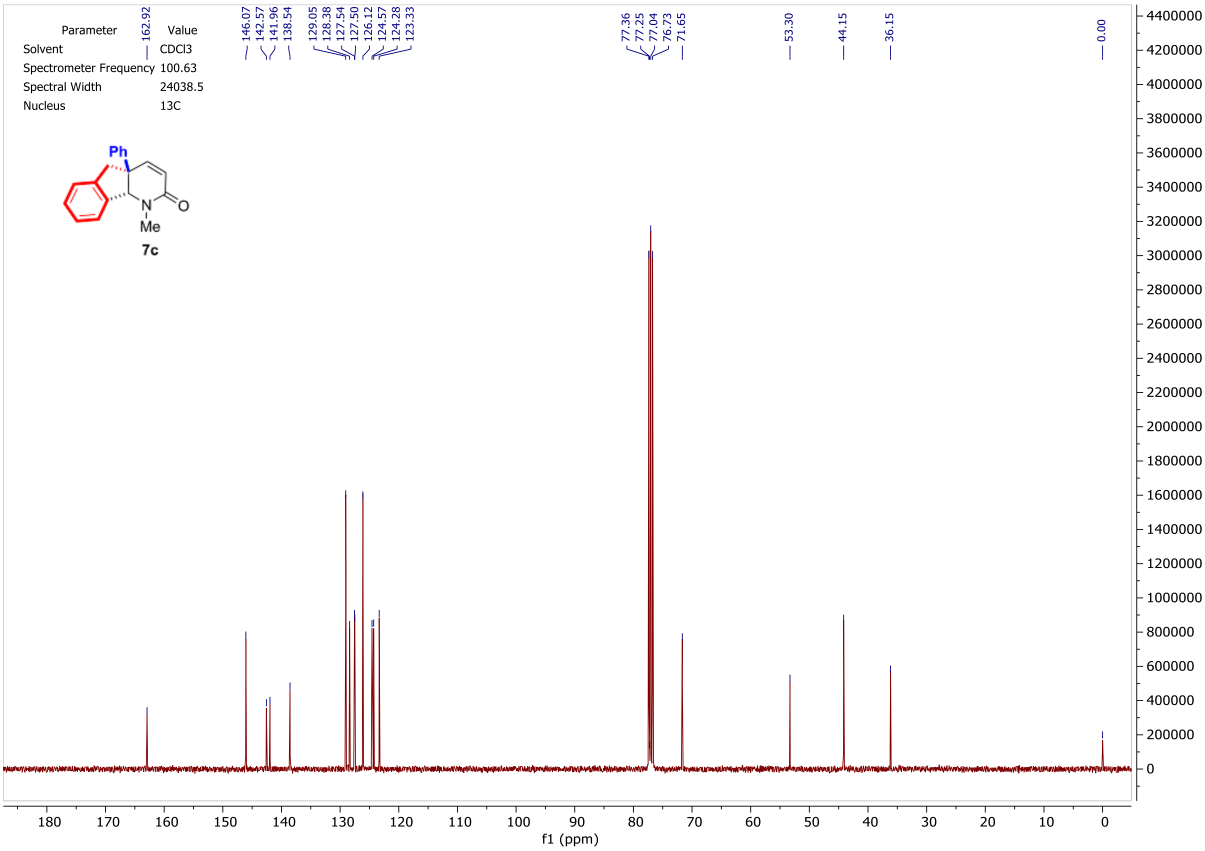
Parameter Value
Solvent CDCl3
Spectrometer Frequency 100.63
Spectral Width 24038.5
Nucleus 13C
Ph
N
O
Me
7c
162.92
146.07
142.57
141.96
138.54
129.05
128.38
127.54
127.50
126.12
124.57
124.28
123.33
77.36
77.25
77.04
76.73
71.65
53.30
44.15
36.15
0.00
f1 (ppm)

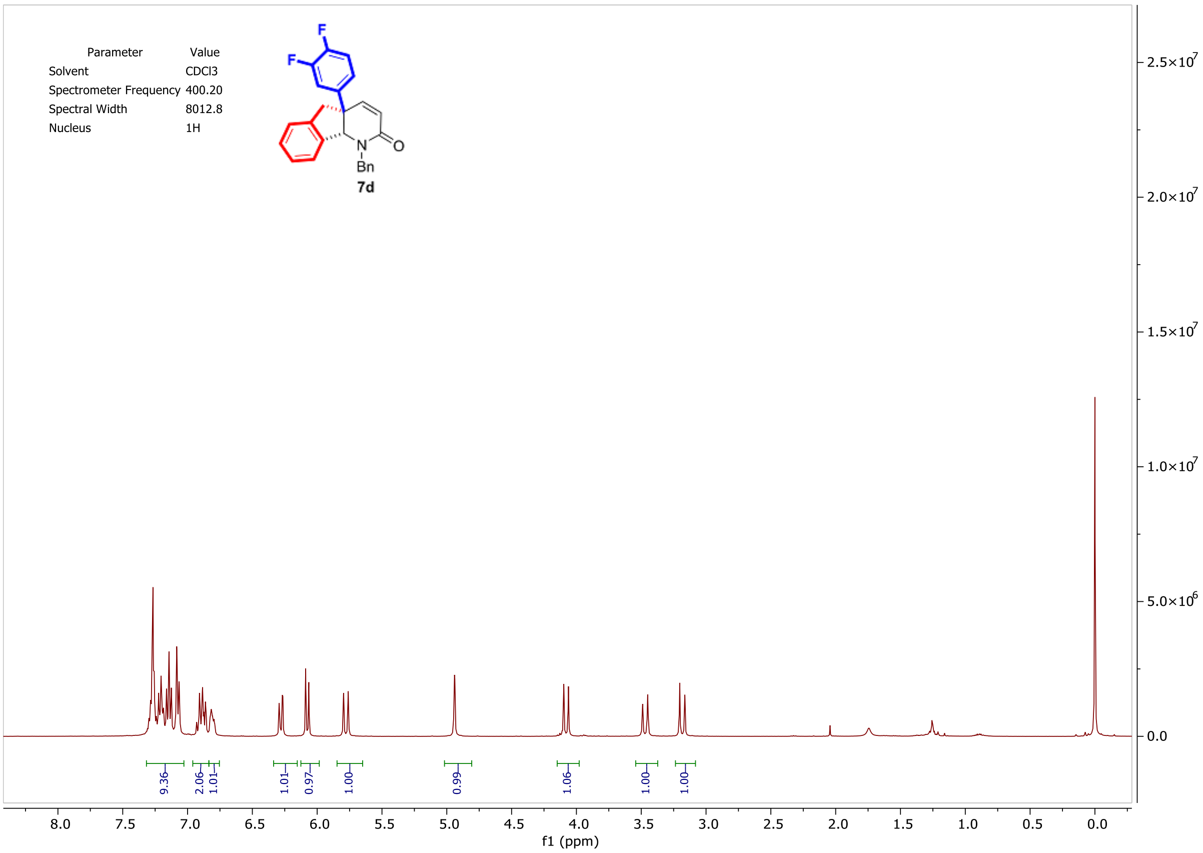
Parameter Value
Solvent CDCl3
Spectrometer Frequency 400.20
Spectral Width 8012.8
Nucleus 1H
F
F
N
O
Bn
7d
9.36
2.06
1.01
1.01
0.97
1.00
0.99
1.06
1.00
1.00
f1 (ppm)

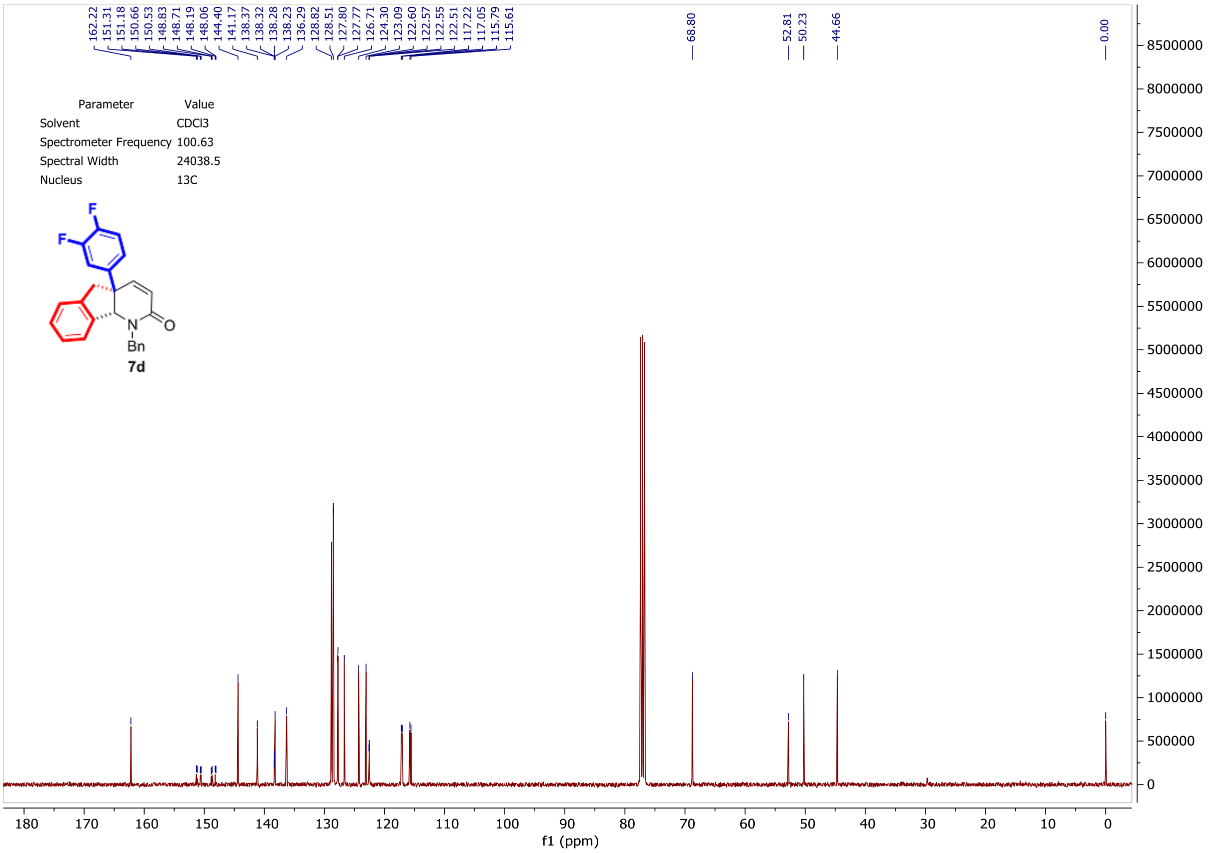
162.22
151.31
151.18
150.66
150.53
148.83
148.71
148.19
148.06
144.40
141.17
138.37
138.32
138.28
138.23
136.29
128.82
128.51
127.80
127.77
126.71
124.30
123.09
122.60
122.57
122.55
122.51
117.22
117.05
115.79
115.61
68.80
52.81
50.23
44.66
0.00
Parameter Value
Solvent CDCl3
Spectrometer Frequency 100.63
Spectral Width 24038.5
Nucleus 13C
F
F
N
O
Bn
7d
f1 (ppm)

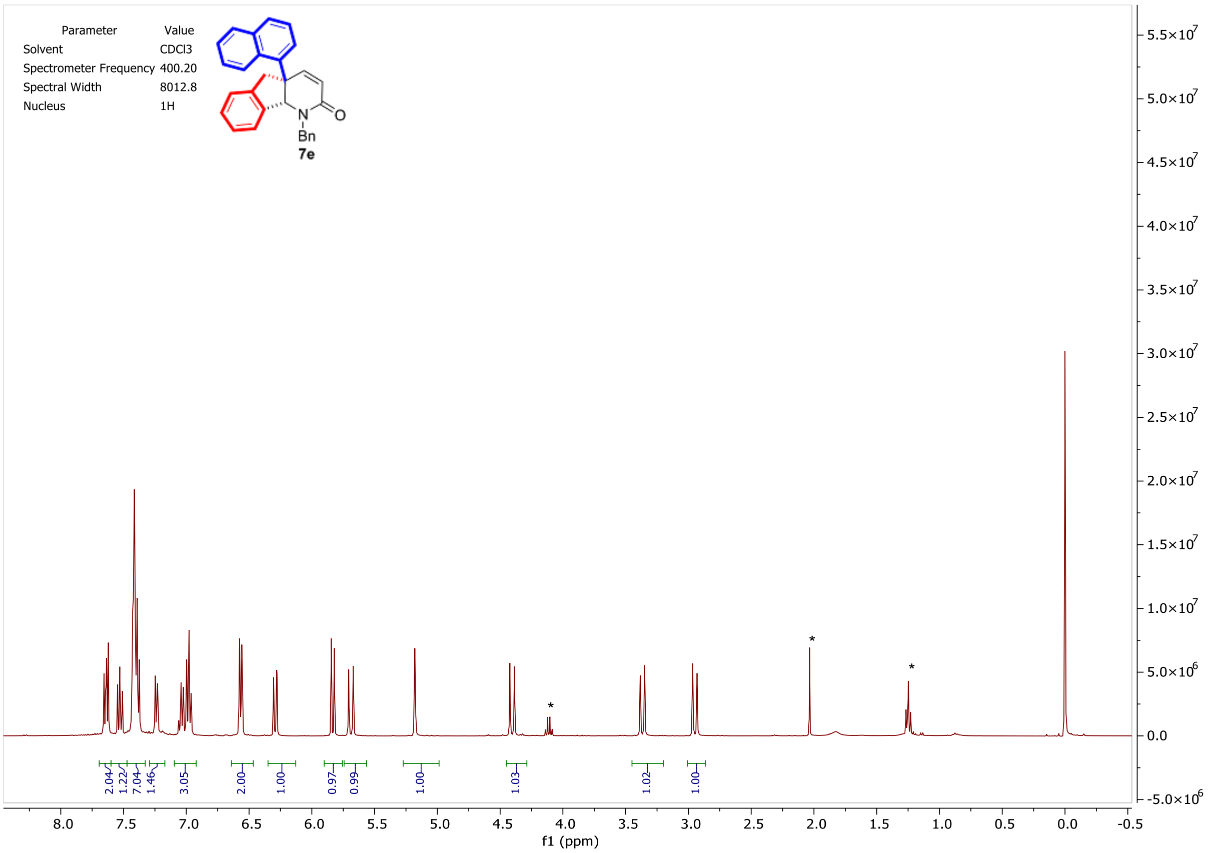

Parameter Value
Solvent CDCl3
Spectrometer Frequency 400.20
Spectral Width 8012.8
Nucleus 1H
N
O
Bn
7e
f1 (ppm)

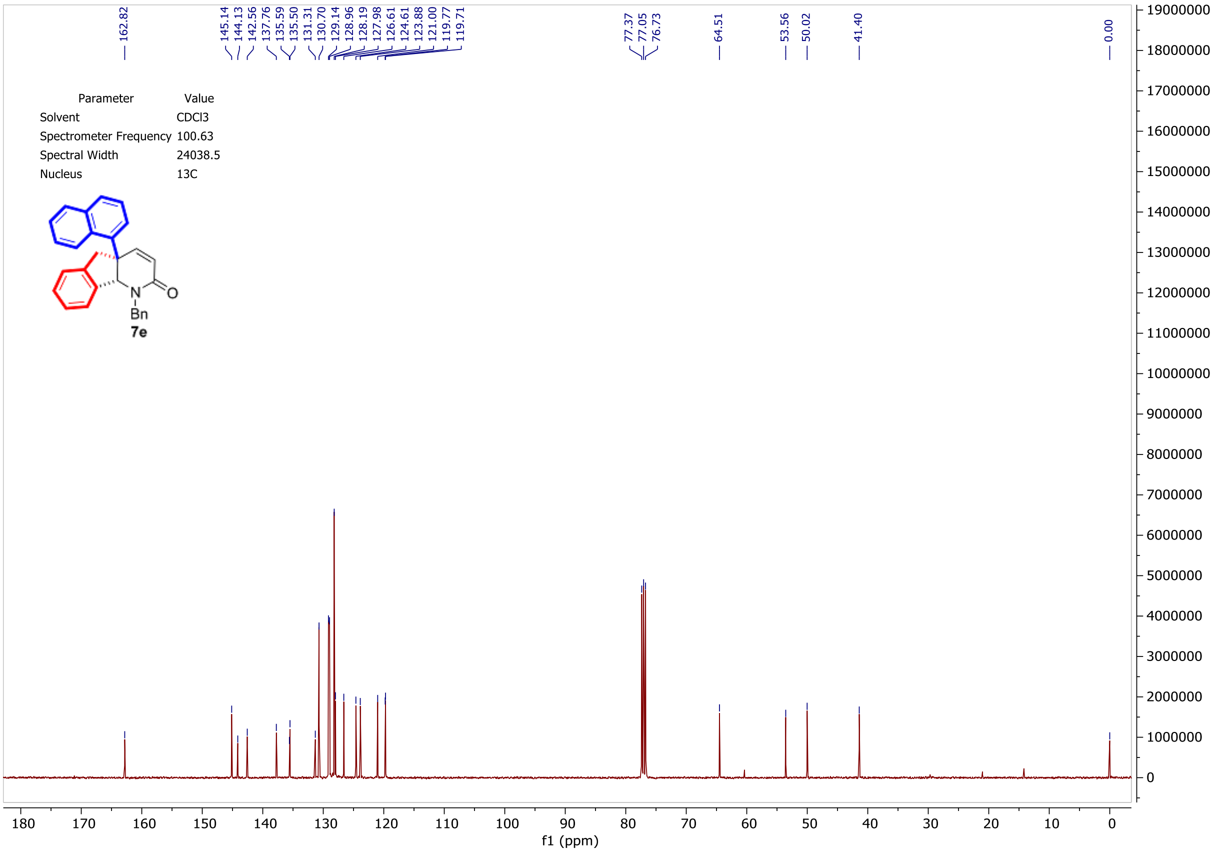

162.82
145.14 144.13 142.56 137.76 135.59 135.50 131.31 130.70 129.14 128.96 128.19 127.98 126.61 124.61 123.88 121.00 119.77 119.71
77.37 77.05 76.73
64.51
53.56
50.02
41.40
0.00
Parameter Value
Solvent CDCl3
Spectrometer Frequency 100.63
Spectral Width 24038.5
Nucleus 13C
N
O
Bn
7e
f1 (ppm)

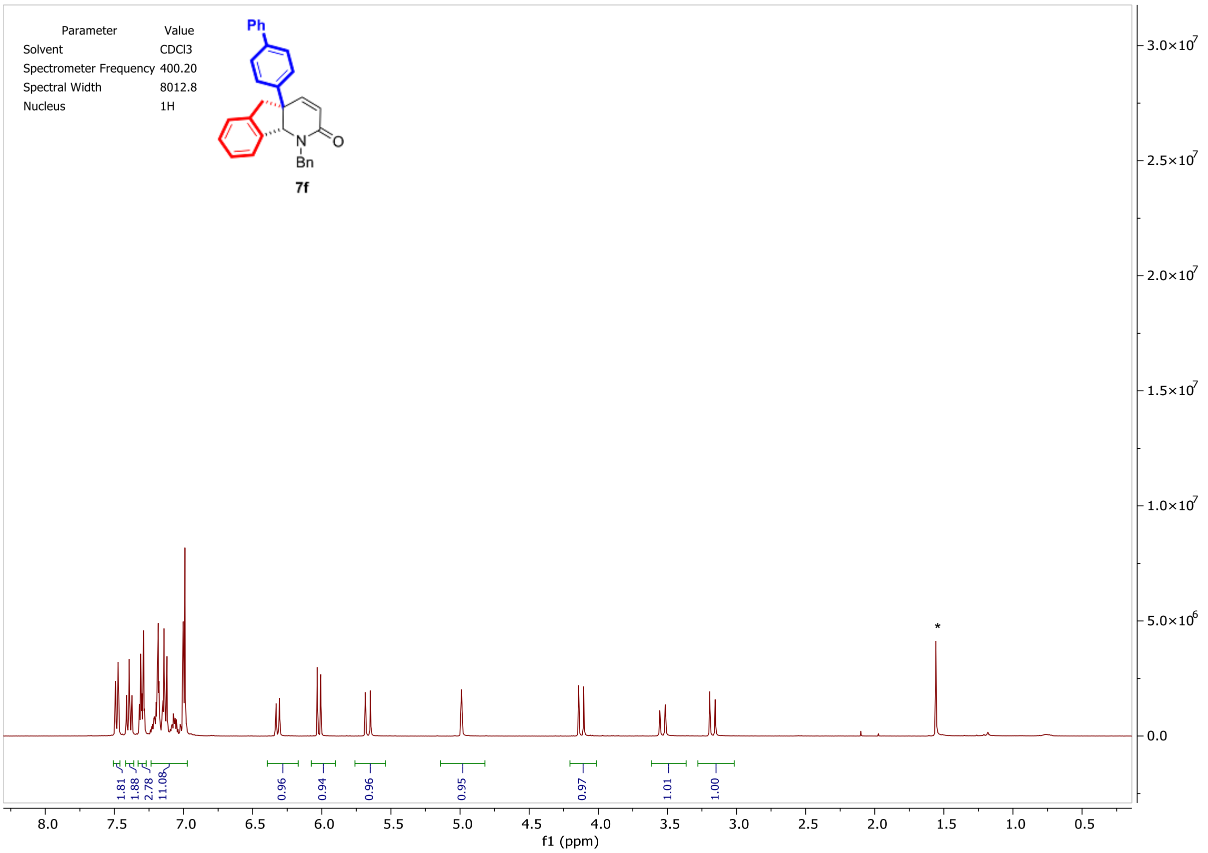
Parameter Value
Solvent CDCl3
Spectrometer Frequency 400.20
Spectral Width 8012.8
Nucleus 1H
Ph
N
O
Bn
7f
1.81
1.88
2.78
11.08
0.96
0.94
0.96
0.95
0.97
1.01
1.00
f1 (ppm)

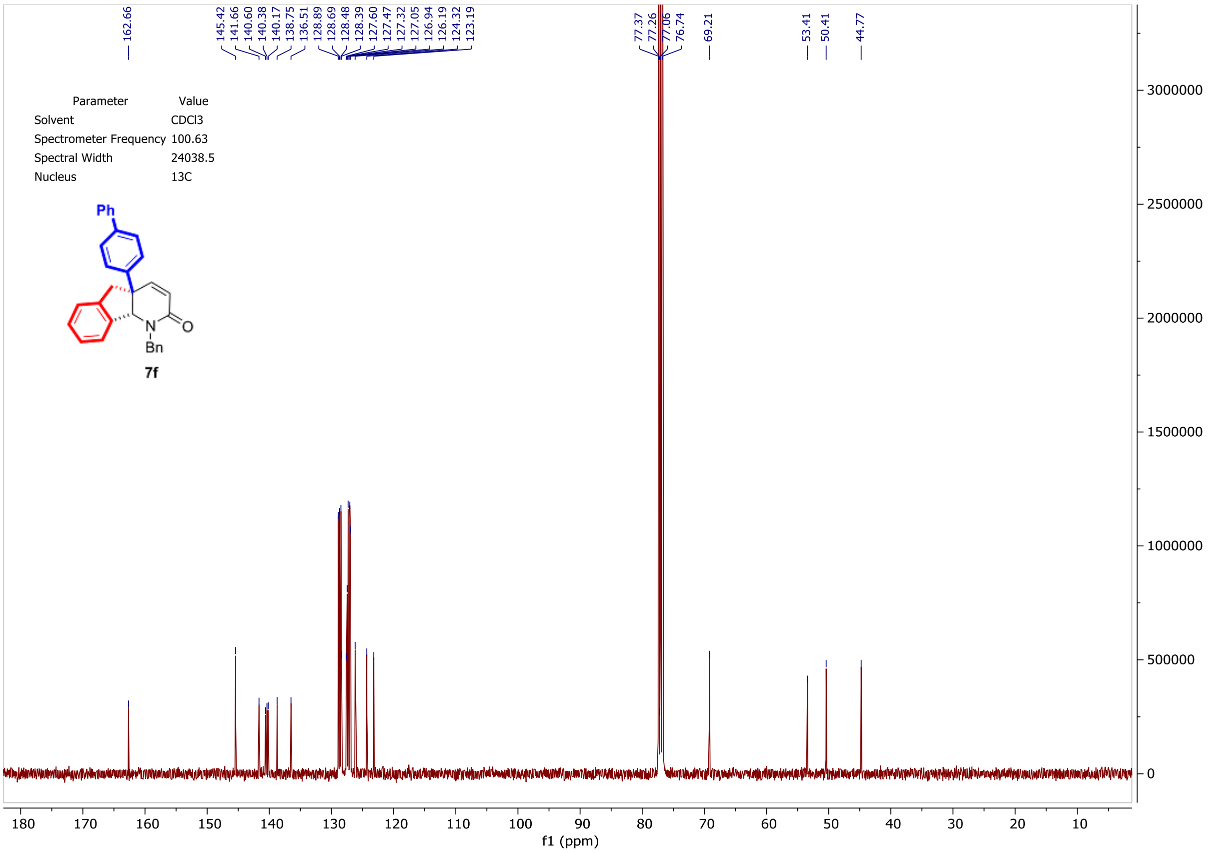
162.66
145.42
141.66
140.60
140.38
140.17
138.75
136.51
128.89
128.69
128.48
128.39
127.60
127.47
127.32
127.05
126.94
126.19
124.32
123.19
77.37
77.26
77.06
76.74
69.21
53.41
50.41
44.77
Parameter Value
Solvent CDCl3
Spectrometer Frequency 100.63
Spectral Width 24038.5
Nucleus 13C
Ph
N
O
Bn
7f
f1 (ppm)

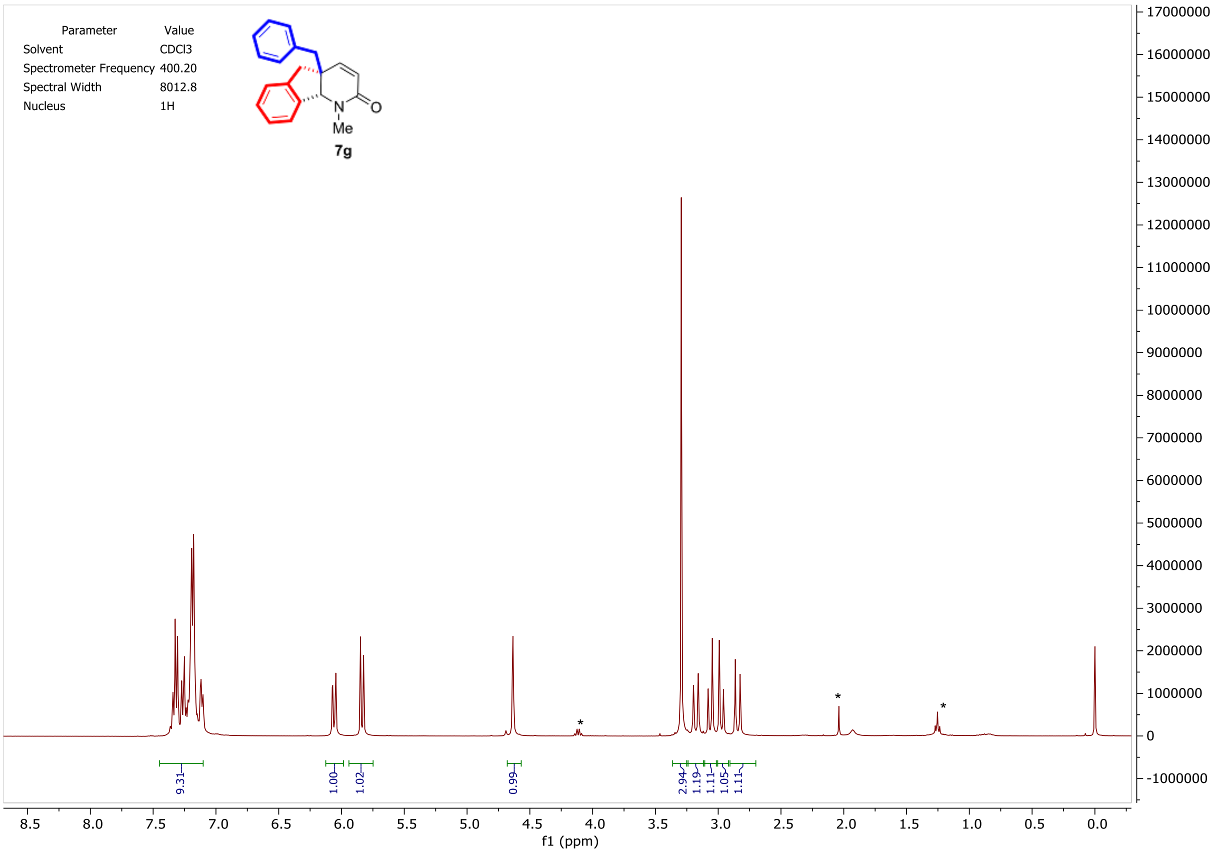
Parameter Value
Solvent CDCl3
Spectrometer Frequency 400.20
Spectral Width 8012.8
Nucleus 1H
N
O
Me
7g
9.31
1.00
1.02
0.99
2.94
1.19
1.11
1.11
1.05
1.11
f1 (ppm)

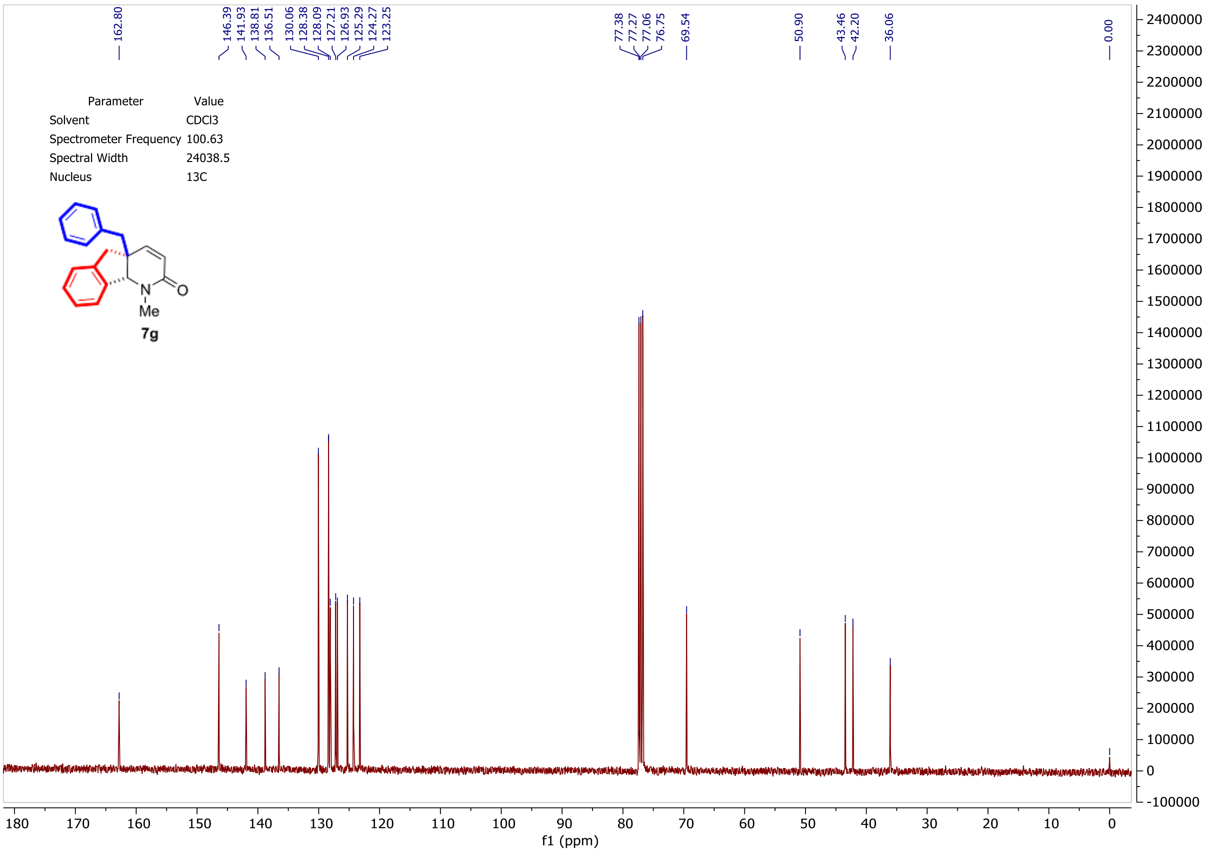
162.80
146.39
141.93
138.81
136.51
130.06
128.38
128.09
127.21
126.93
125.29
124.27
123.25
77.38
77.27
77.06
76.75
69.54
50.90
43.46
42.20
36.06
0.00
Parameter Value
Solvent CDCl3
Spectrometer Frequency 100.63
Spectral Width 24038.5
Nucleus 13C
N
O
Me
7g
f1 (ppm)

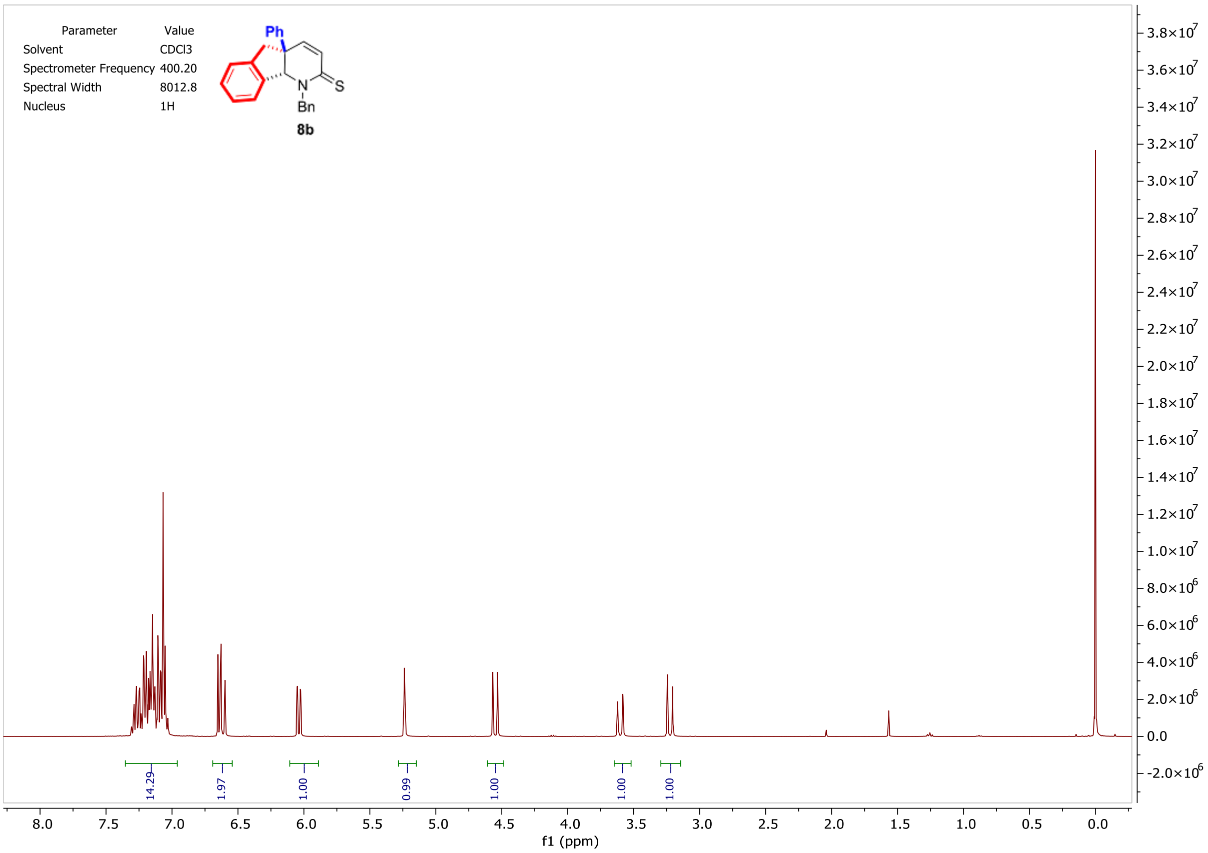
Parameter Value
Solvent CDCl3
Spectrometer Frequency 400.20
Spectral Width 8012.8
Nucleus 1H
Ph
N
S
Bn
8b
14.29
1.97
1.00
0.99
1.00
1.00
1.00
f1 (ppm)

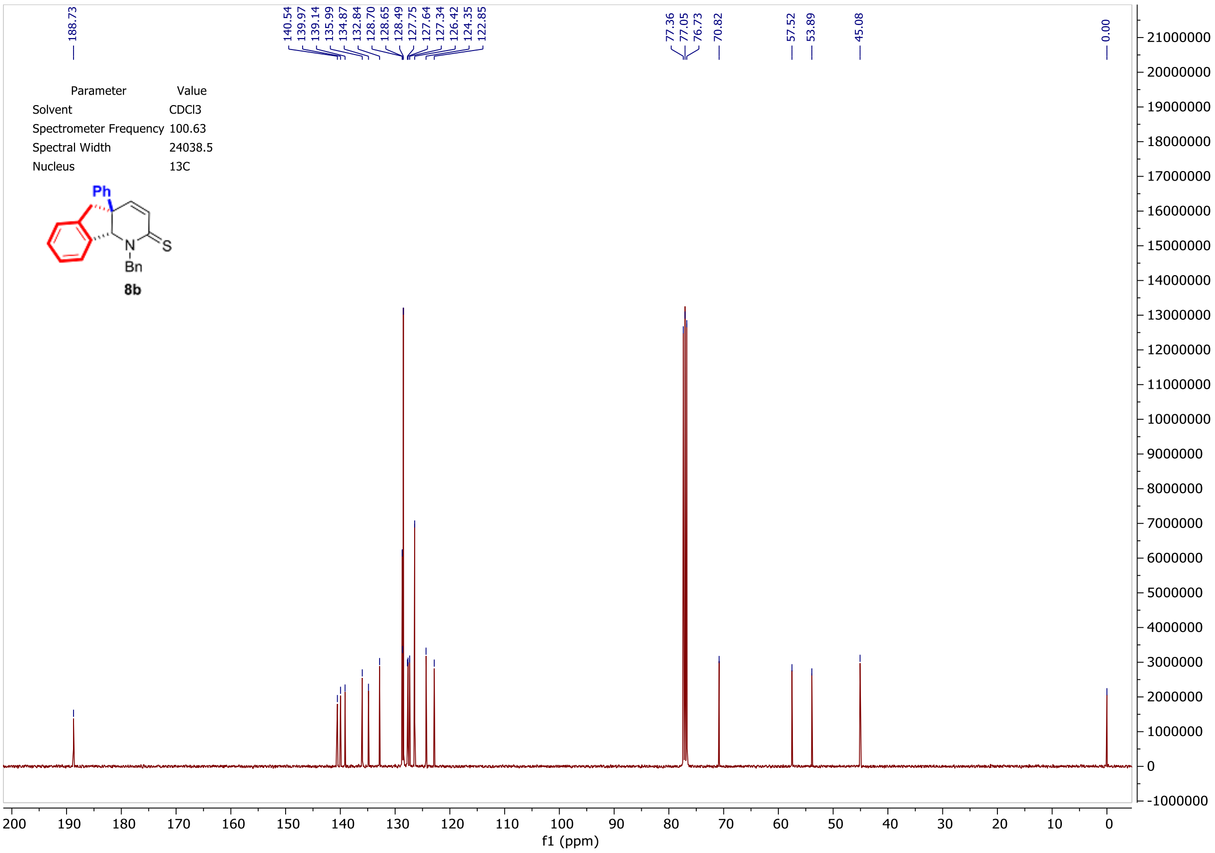
188.73
140.54
139.97
139.14
135.99
134.87
132.84
128.70
128.65
128.49
127.75
127.64
127.34
126.42
124.35
122.85
77.36
77.05
76.73
70.82
57.52
53.89
45.08
0.00
Parameter Value
Solvent CDCl3
Spectrometer Frequency 100.63
Spectral Width 24038.5
Nucleus 13C
Ph
N
S
Bn
8b
f1 (ppm)

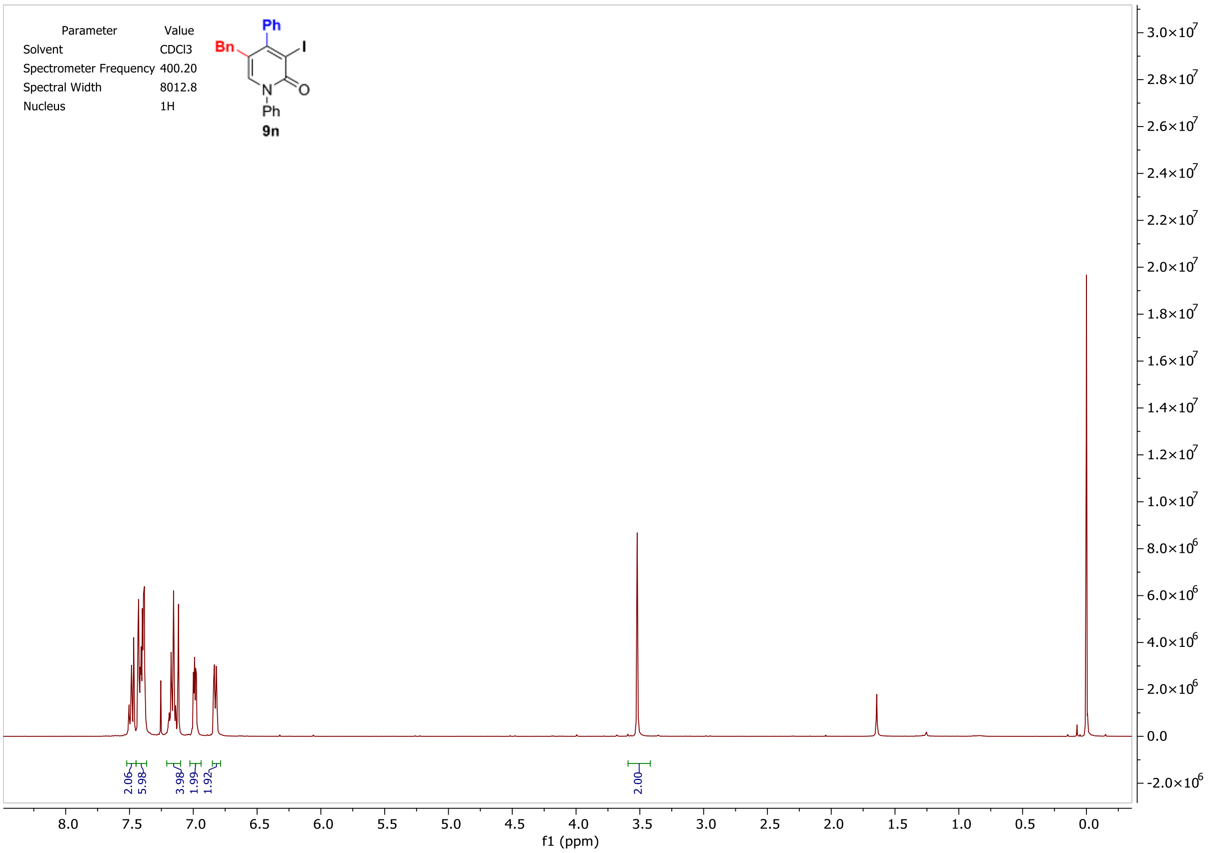
Parameter Value
Solvent CDCl3
Spectrometer Frequency 400.20
Spectral Width 8012.8
Nucleus 1H
Ph
Bn
I
N
O
Ph
9n
2.06
5.98
3.98
1.99
1.92
2.00
f1 (ppm)

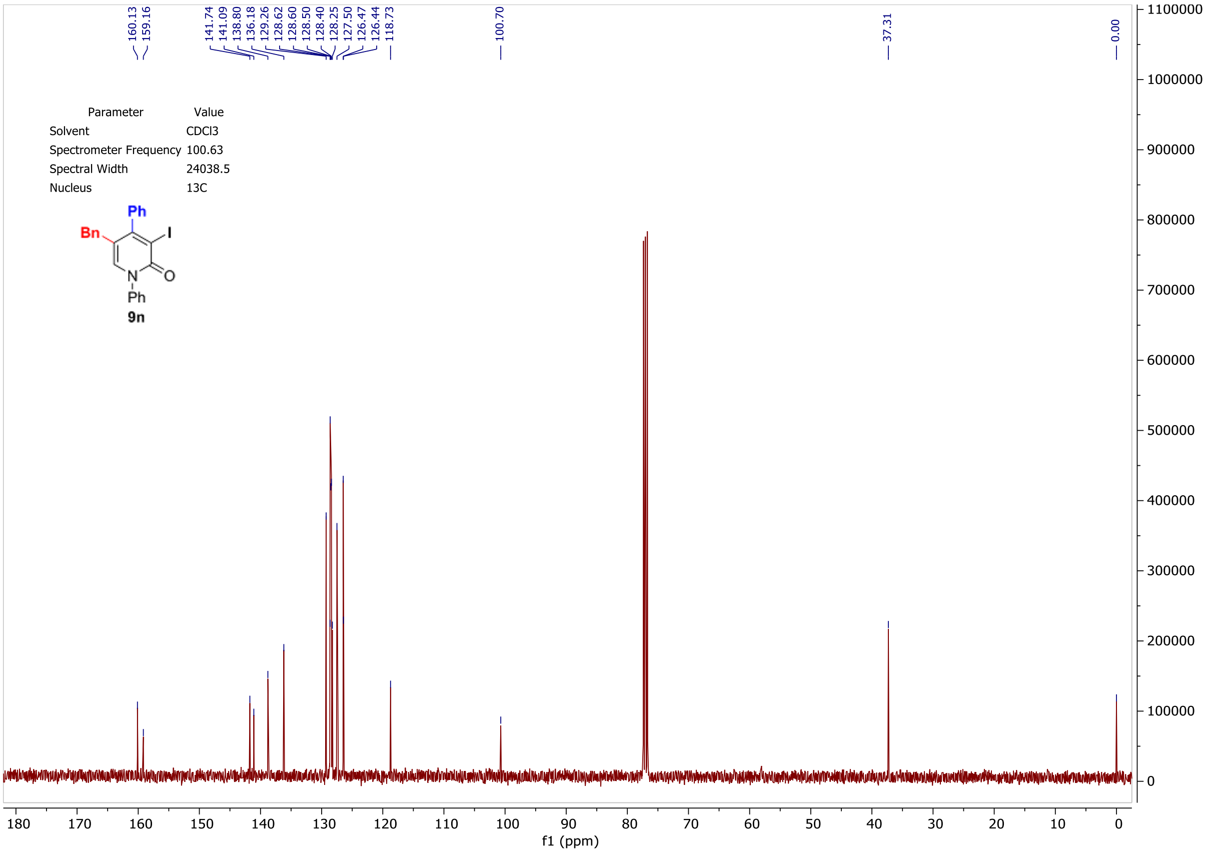
160.13
159.16
141.74
141.09
138.80
136.18
129.26
128.62
128.60
128.50
128.40
128.25
127.50
126.47
126.44
118.73
100.70
37.31
0.00
Parameter Value
Solvent CDCl3
Spectrometer Frequency 100.63
Spectral Width 24038.5
Nucleus 13C
Ph
Bn
I
N
O
Ph
9n
f1 (ppm)

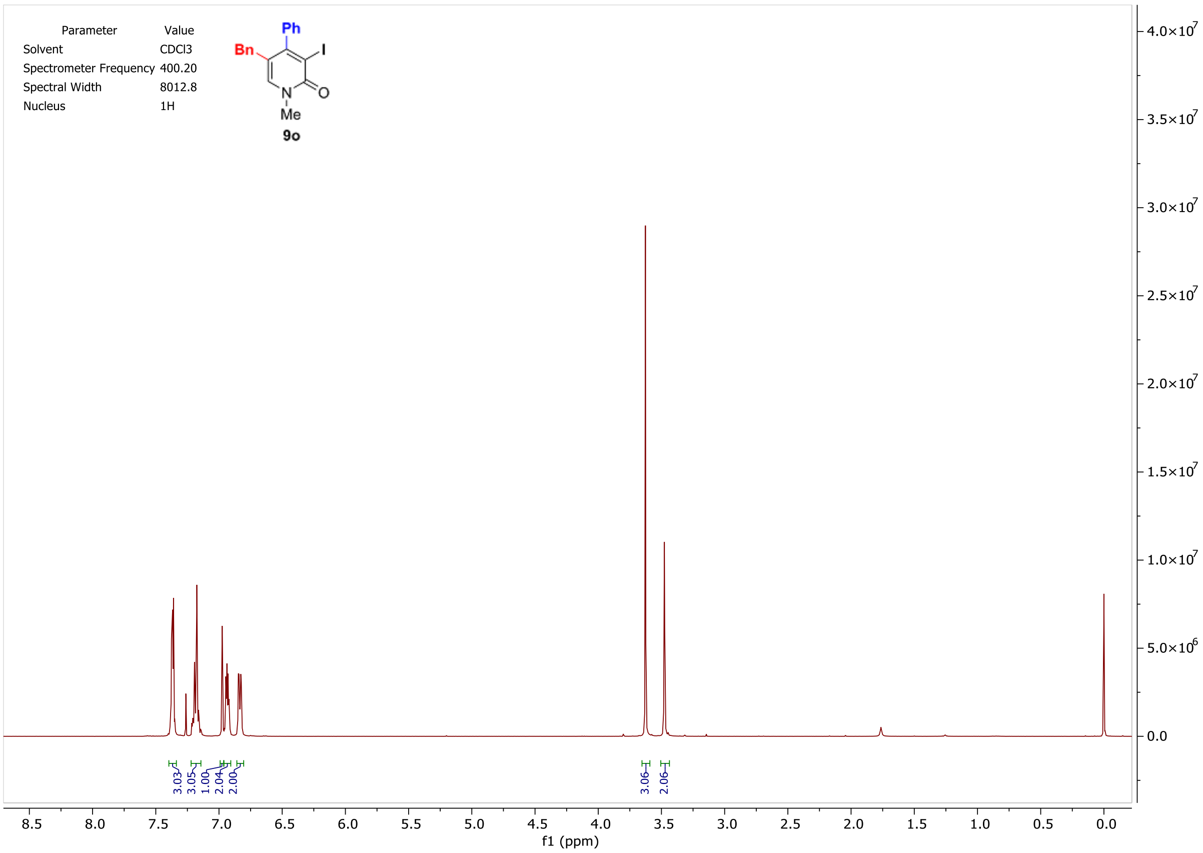

Parameter Value
Solvent CDCl3
Spectrometer Frequency 400.20
Spectral Width 8012.8
Nucleus 1H
Ph
Bn
I
N
O
Me
9o
3.03
3.05
1.00
2.04
2.00
3.06
2.06
f1 (ppm)

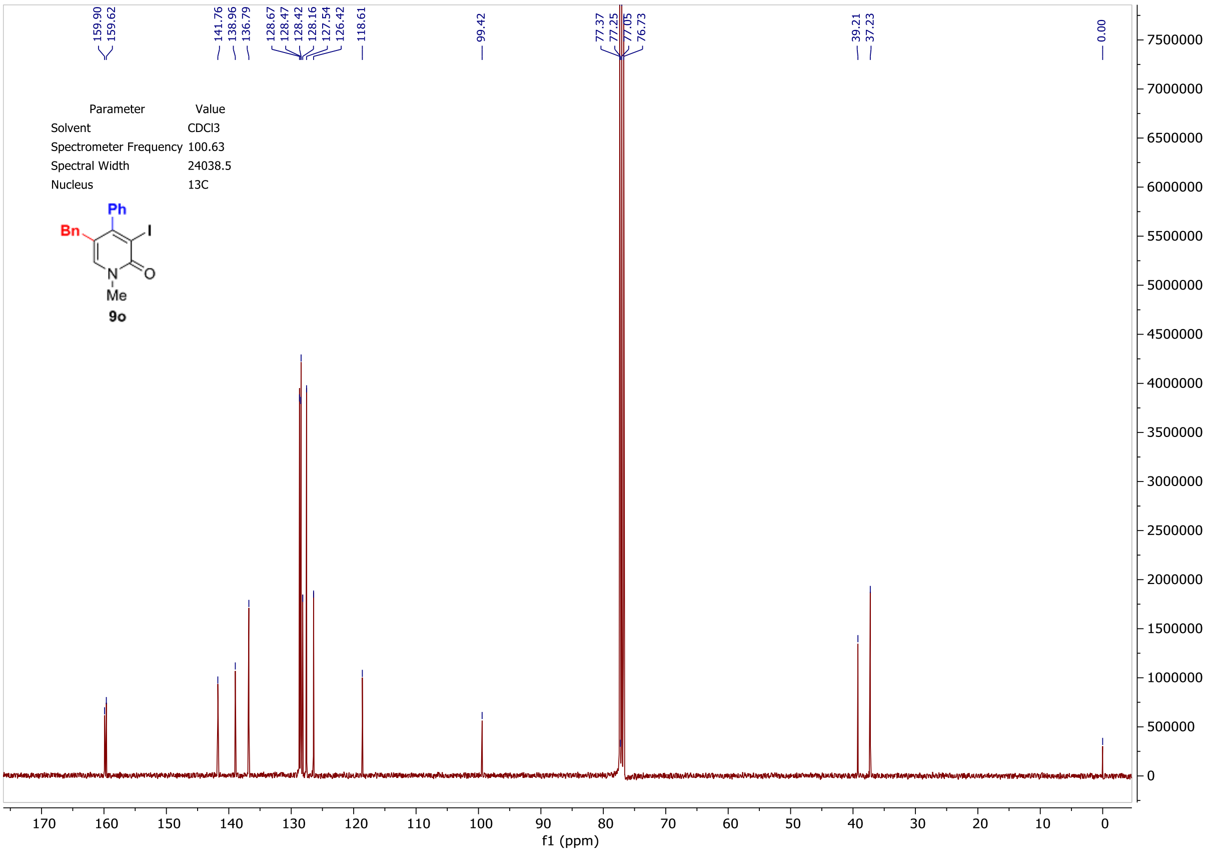

159.90
159.62
141.76
138.96
136.79
128.67
128.47
128.42
128.16
127.54
126.42
118.61
99.42
77.37
77.25
77.05
76.73
39.21
37.23
0.00
Parameter Value
Solvent CDCl3
Spectrometer Frequency 100.63
Spectral Width 24038.5
Nucleus 13C
Ph
Bn
I
N
O
Me
9o
f1 (ppm)

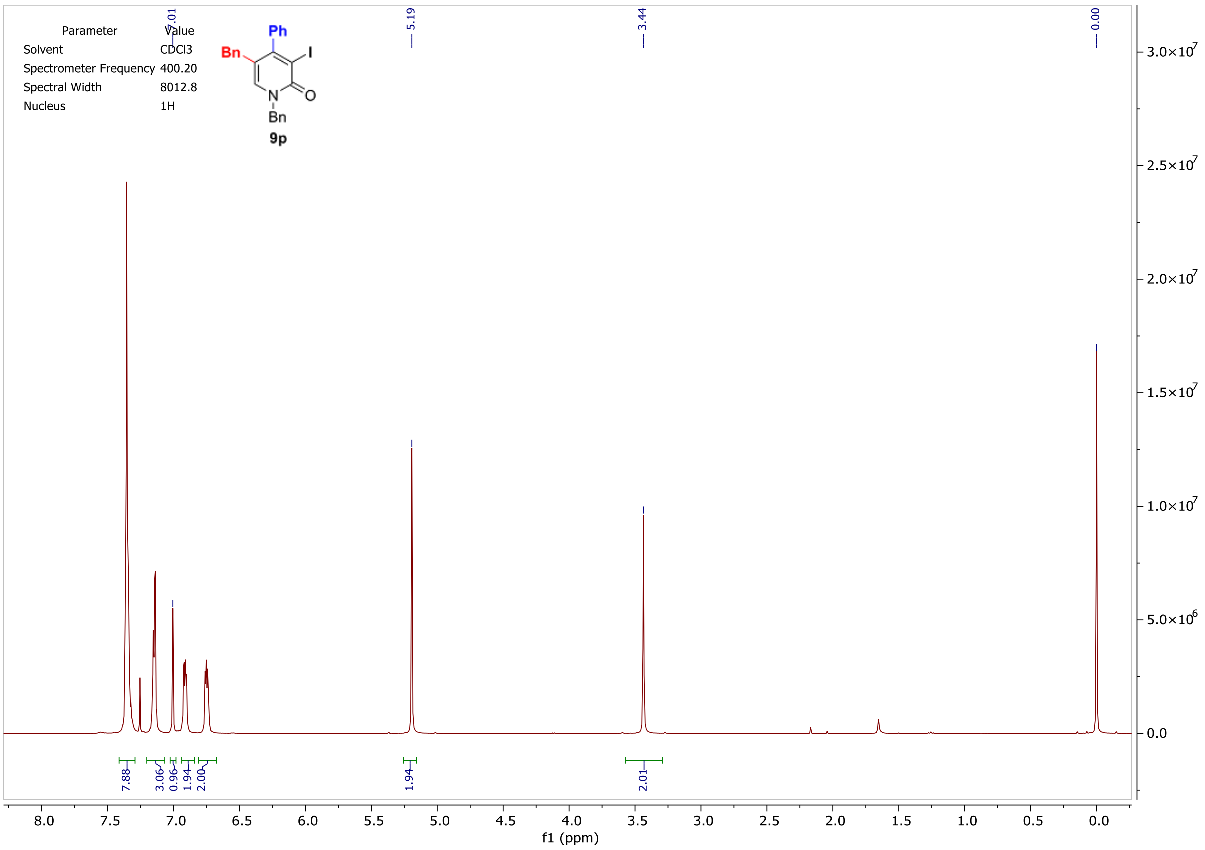
Parameter Value
Solvent CDCl3
Spectrometer Frequency 400.20
Spectral Width 8012.8
Nucleus 1H
Ph
Bn
I
N
O
Bn
9p
7.01
5.19
3.44
0.00
7.88
3.06
0.96
1.94
2.00
1.94
2.01
f1 (ppm)

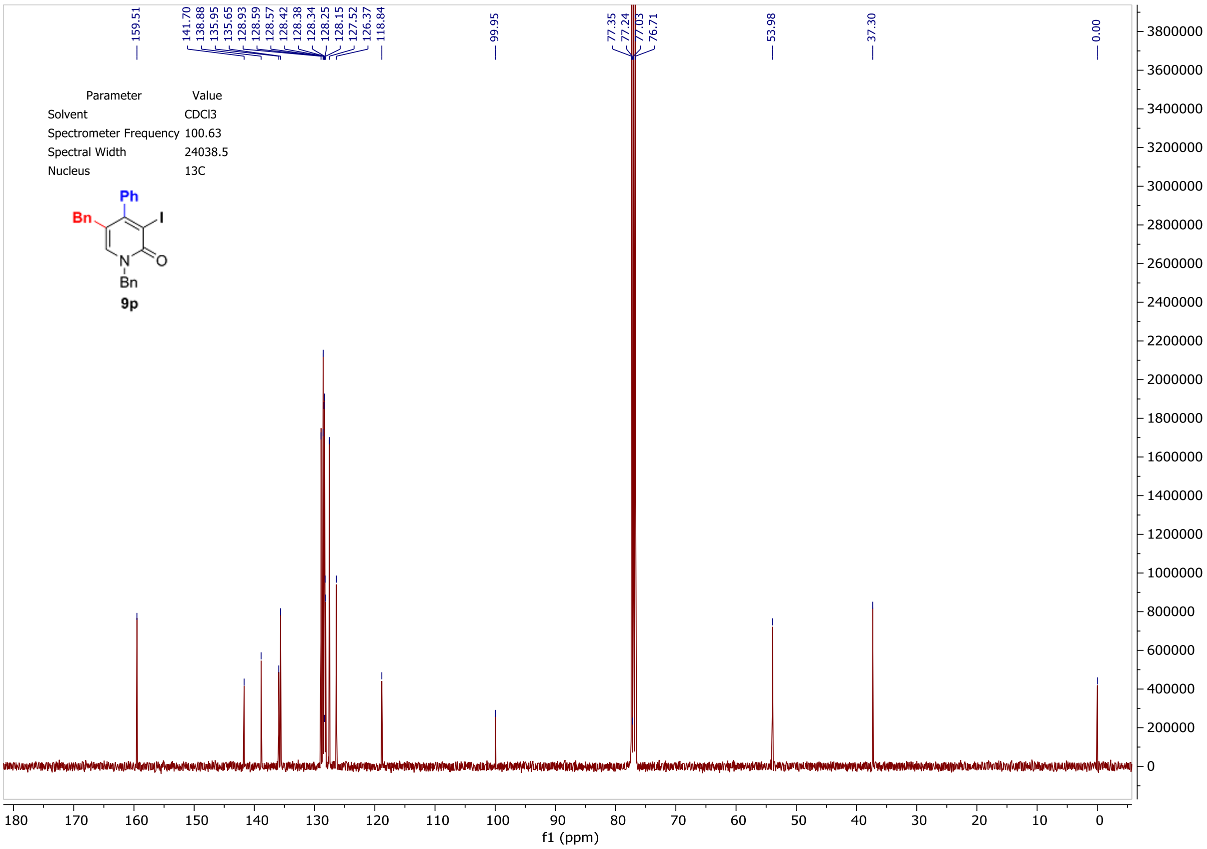
159.51
141.70
138.88
135.95
135.65
128.93
128.59
128.57
128.42
128.38
128.34
128.25
128.15
127.52
126.37
118.84
99.95
77.35
77.24
77.03
76.71
53.98
37.30
0.00
Parameter Value
Solvent CDCl3
Spectrometer Frequency 100.63
Spectral Width 24038.5
Nucleus 13C
Ph
Bn
I
N
O
Bn
9p
f1 (ppm)

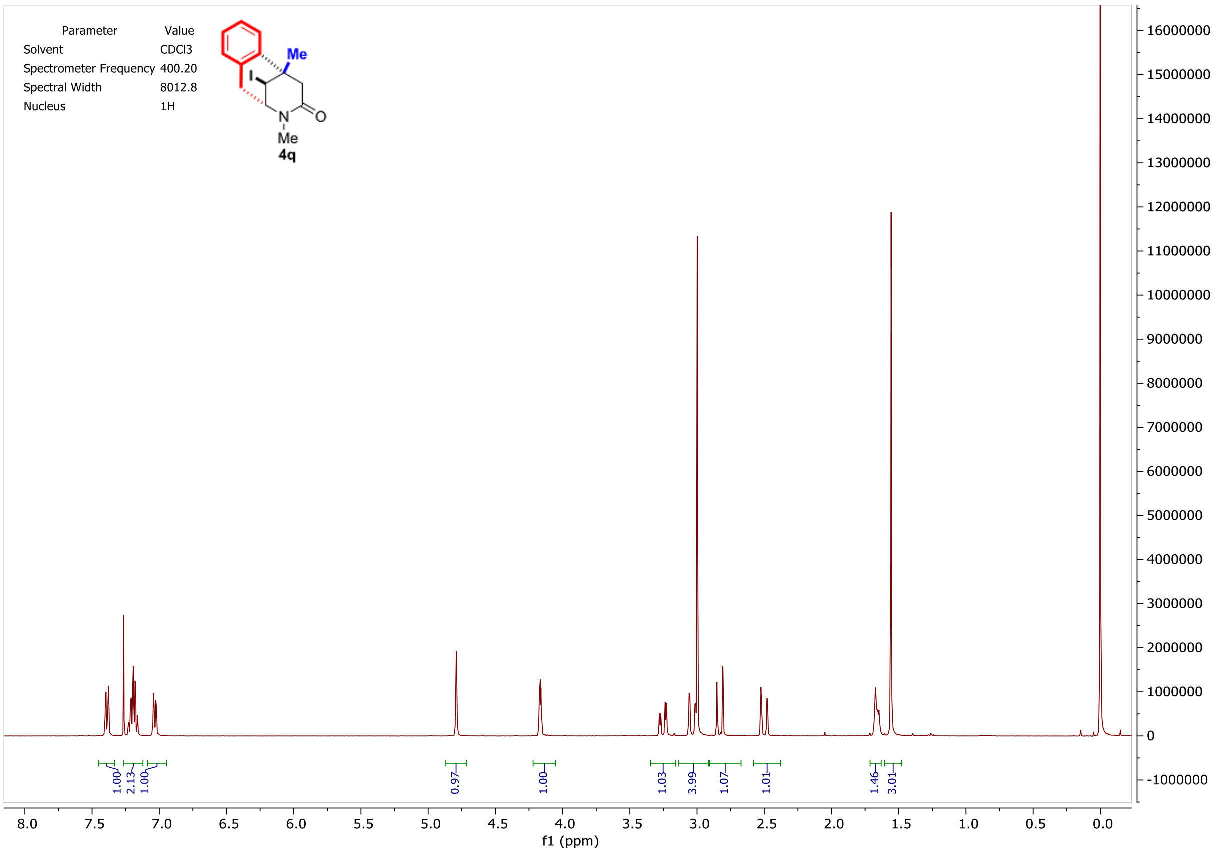

Parameter Value
Solvent CDCl3
Spectrometer Frequency 400.20
Spectral Width 8012.8
Nucleus 1H
Me
I
N
O
Me
4q
1.00
2.13
1.00
0.97
1.00
1.03
3.99
1.07
1.01
1.46
3.01
8.0 7.5 7.0 6.5 6.0 5.5 5.0 4.5 4.0 3.5 3.0 2.5 2.0 1.5 1.0 0.5 0.0
f1 (ppm)
16000000
15000000
14000000
13000000
12000000
11000000
10000000
9000000
8000000
7000000
6000000
5000000
4000000
3000000
2000000
1000000
0
-1000000

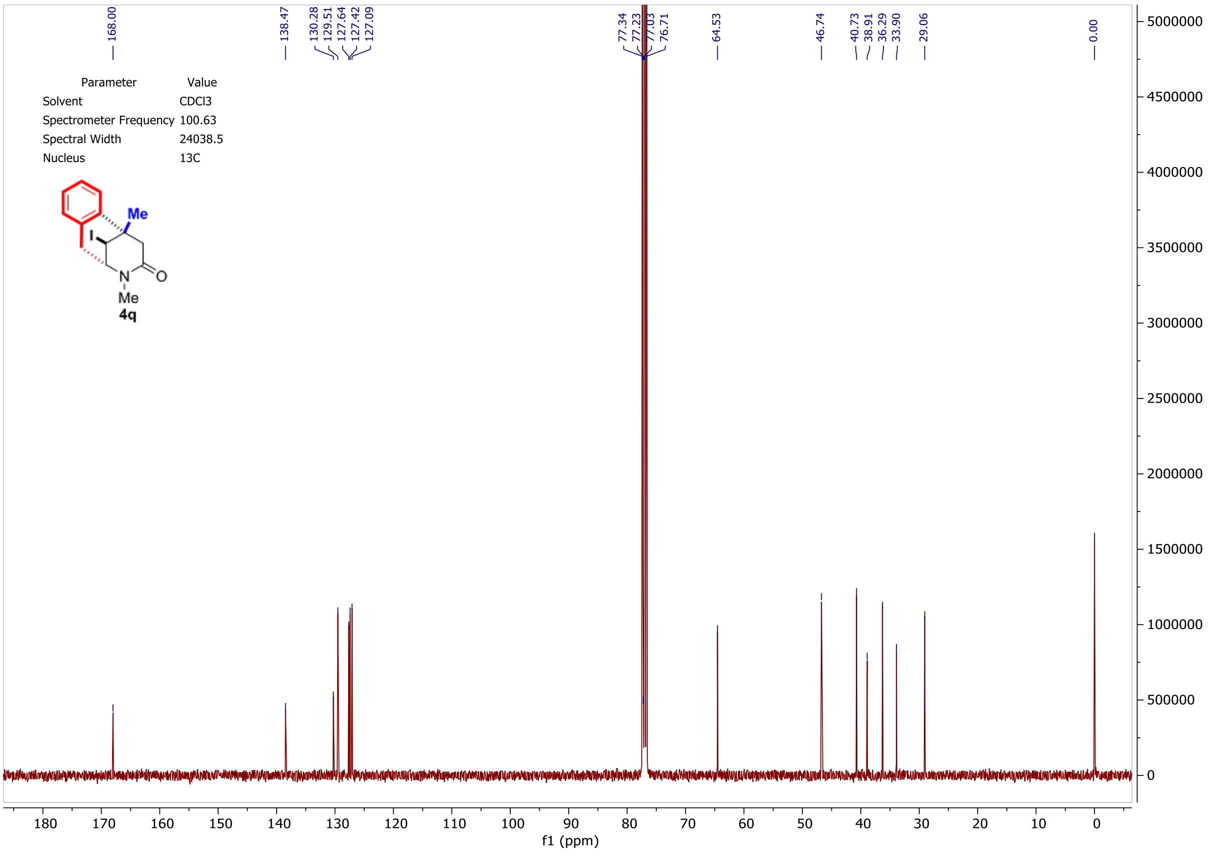

168.00
138.47
130.28
129.51
127.64
127.42
127.09
77.34
77.23
77.03
76.71
64.53
46.74
40.73
38.91
36.29
33.90
29.06
0.00
Parameter Value
Solvent CDCl3
Spectrometer Frequency 100.63
Spectral Width 24038.5
Nucleus 13C
Me
I
N
O
Me
4q
180 170 160 150 140 130 120 110 100 90 80 70 60 50 40 30 20 10 0
f1 (ppm)
5000000
4500000
4000000
3500000
3000000
2500000
2000000
1500000
1000000
500000
0

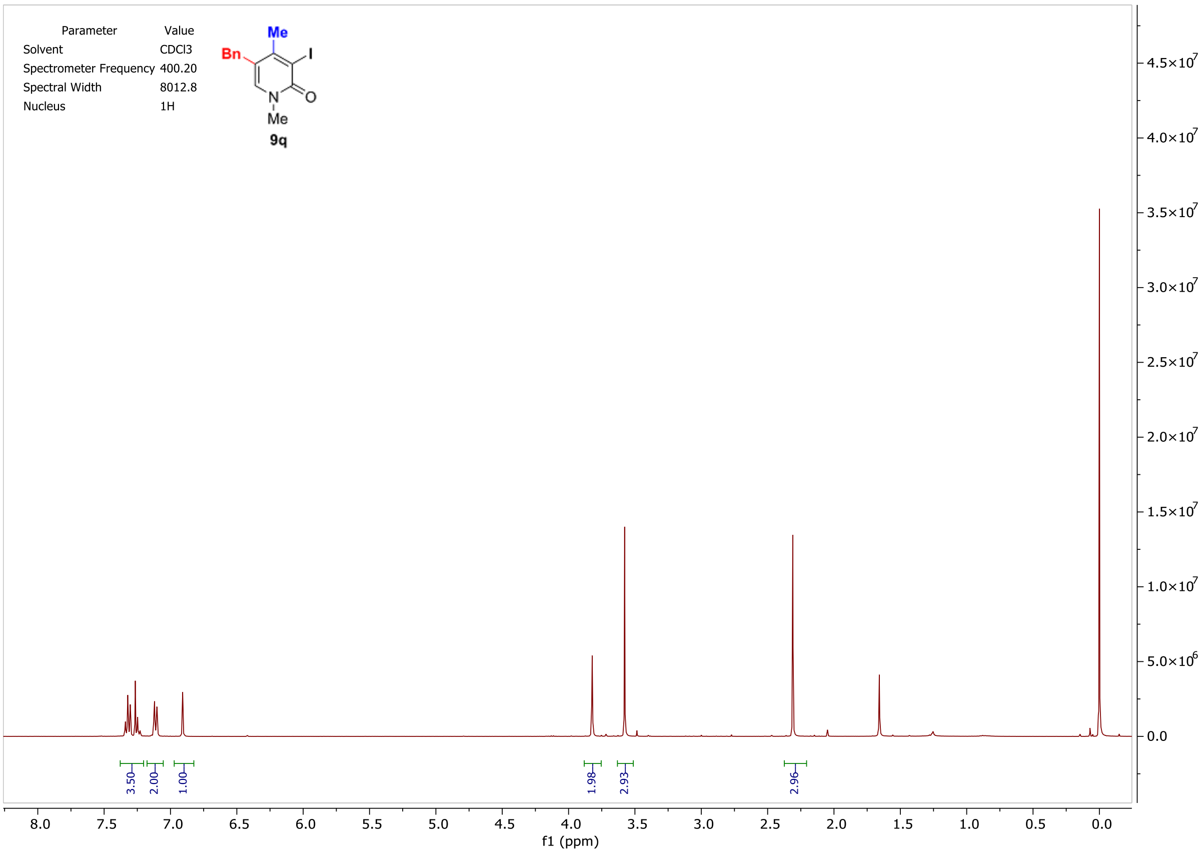
Parameter Value
Solvent CDCl3
Spectrometer Frequency 400.20
Spectral Width 8012.8
Nucleus 1H
Me
Bn
I
N
O
Me
9q
3.50
2.00
1.00
1.98
2.93
2.96
f1 (ppm)

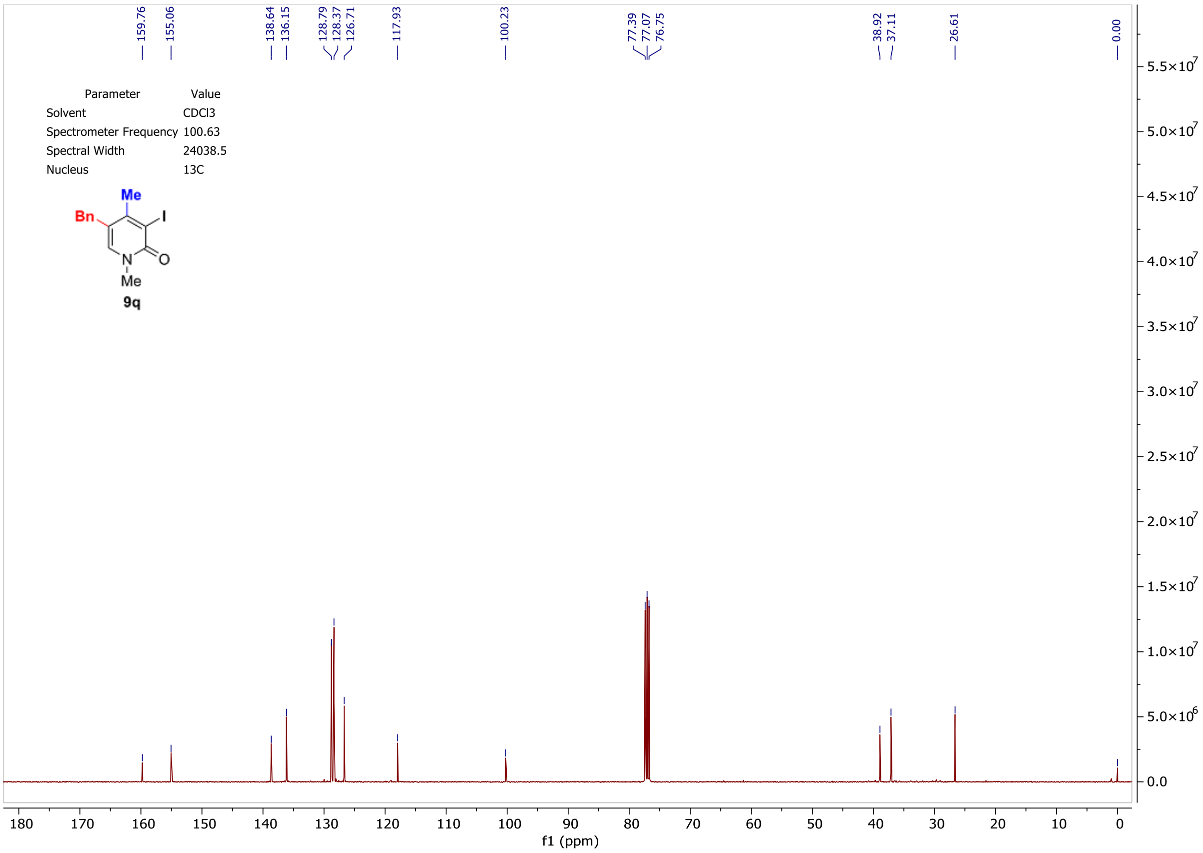
159.76
155.06
138.64
136.15
128.79
128.37
126.71
117.93
100.23
77.39
77.07
76.75
38.92
37.11
26.61
0.00
Parameter Value
Solvent CDCl3
Spectrometer Frequency 100.63
Spectral Width 24038.5
Nucleus 13C
Me
Bn
I
N
O
Me
9q
f1 (ppm)

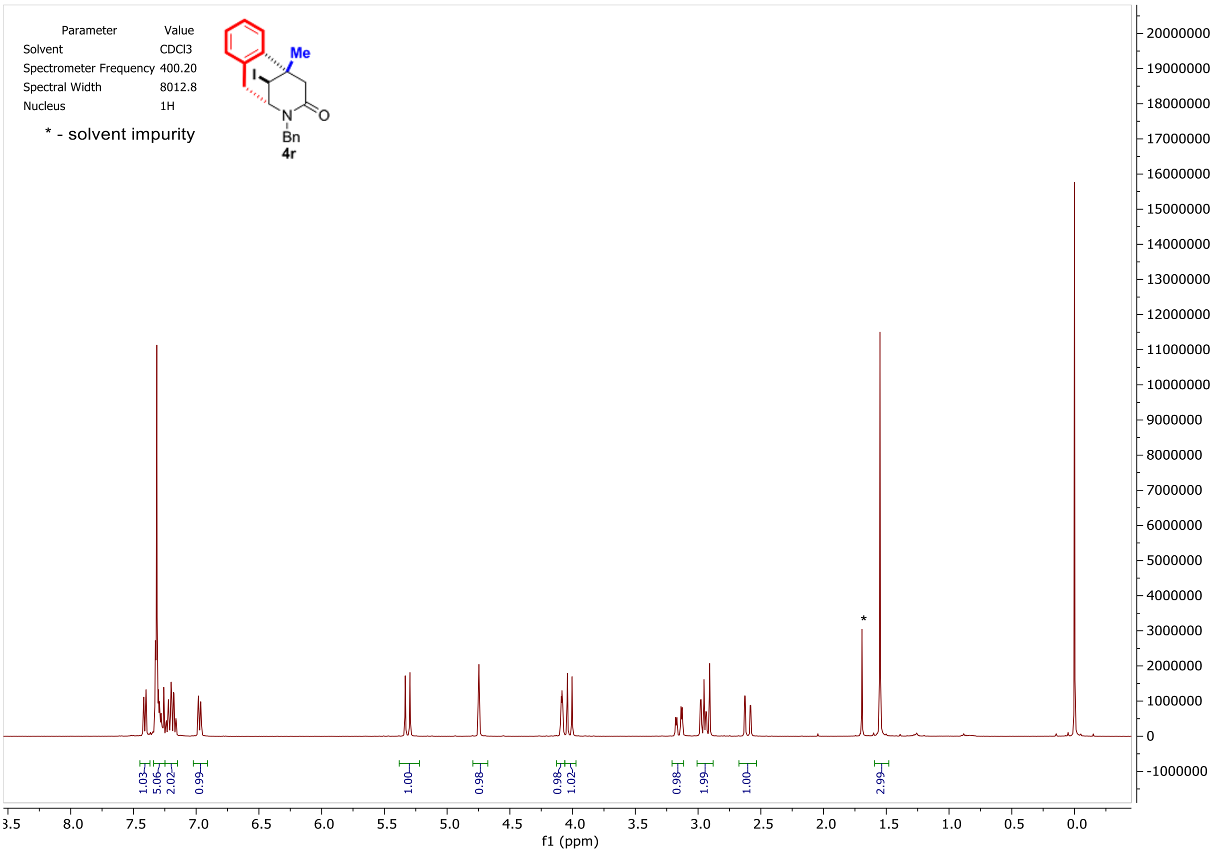
Parameter Value
Solvent CDCl3
Spectrometer Frequency 400.20
Spectral Width 8012.8
Nucleus 1H
* - solvent impurity
Me
I
N
O
Bn
4r
1.03
5.06
2.02
0.99
1.00
0.98
0.98
1.02
0.98
1.99
1.00
2.99
f1 (ppm)

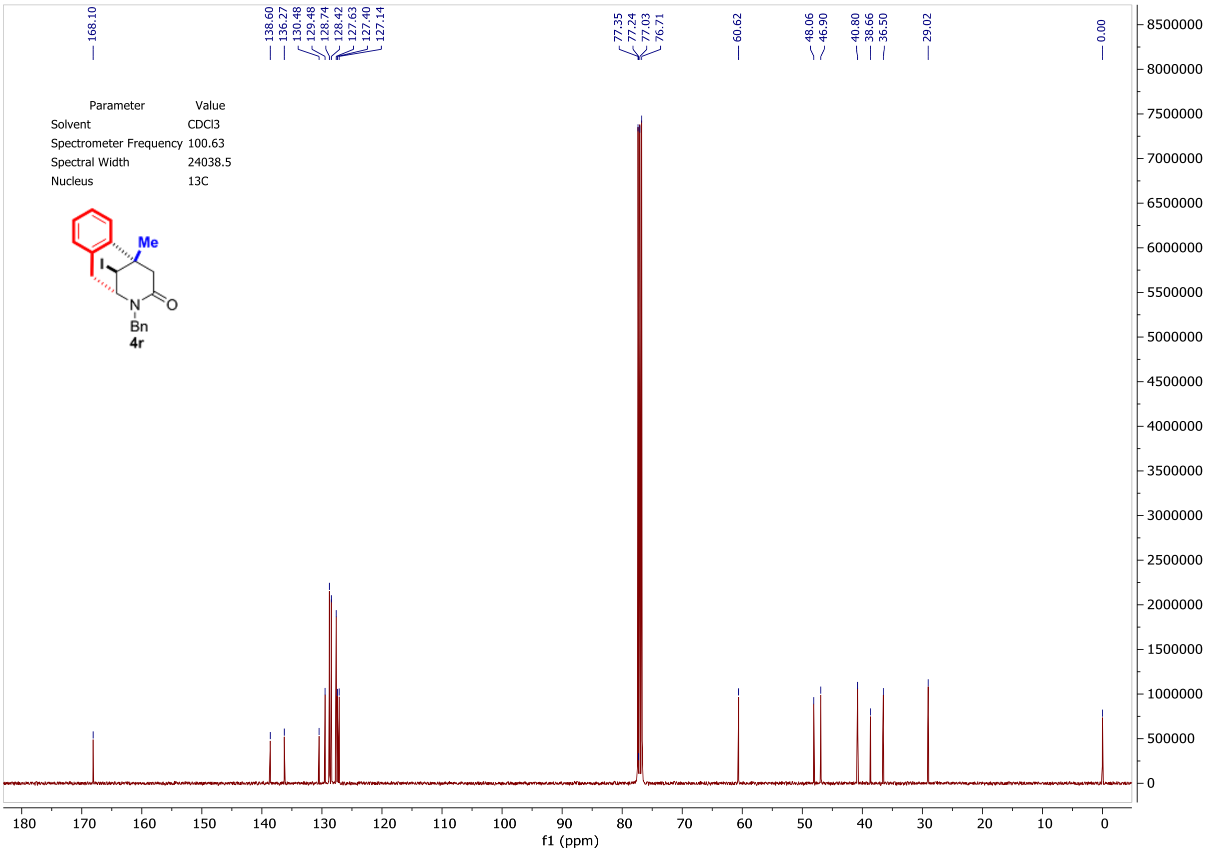
168.10
138.60
136.27
130.48
129.48
128.74
128.42
127.63
127.40
127.14
77.35
77.24
77.03
76.71
60.62
48.06
46.90
40.80
38.66
36.50
29.02
0.00
Parameter Value
Solvent CDCl3
Spectrometer Frequency 100.63
Spectral Width 24038.5
Nucleus 13C
Me
I
N
O
Bn
4r
f1 (ppm)

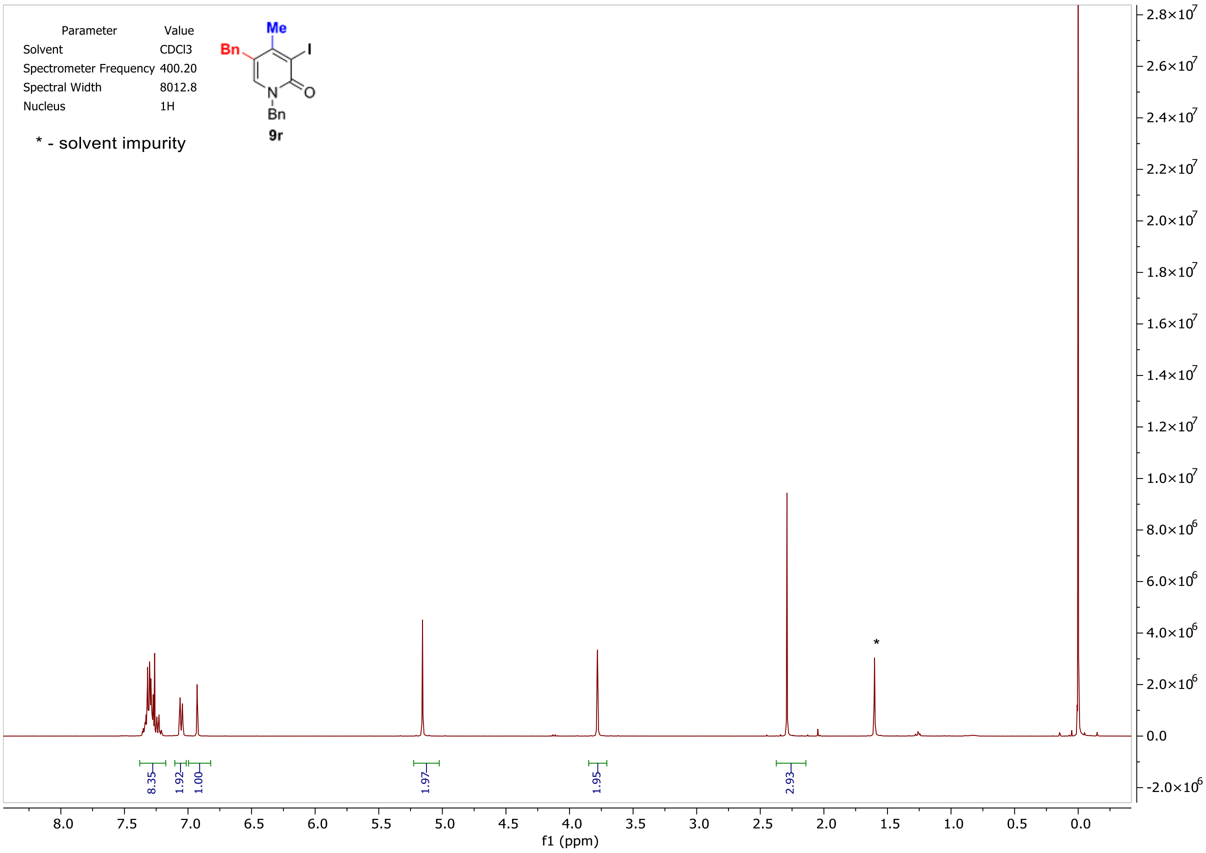
Parameter Value
Solvent CDCl3
Spectrometer Frequency 400.20
Spectral Width 8012.8
Nucleus 1H
* - solvent impurity
Me
Bn
I
N
O
Bn
9r
8.35
1.92
1.00
1.97
1.95
2.93
f1 (ppm)

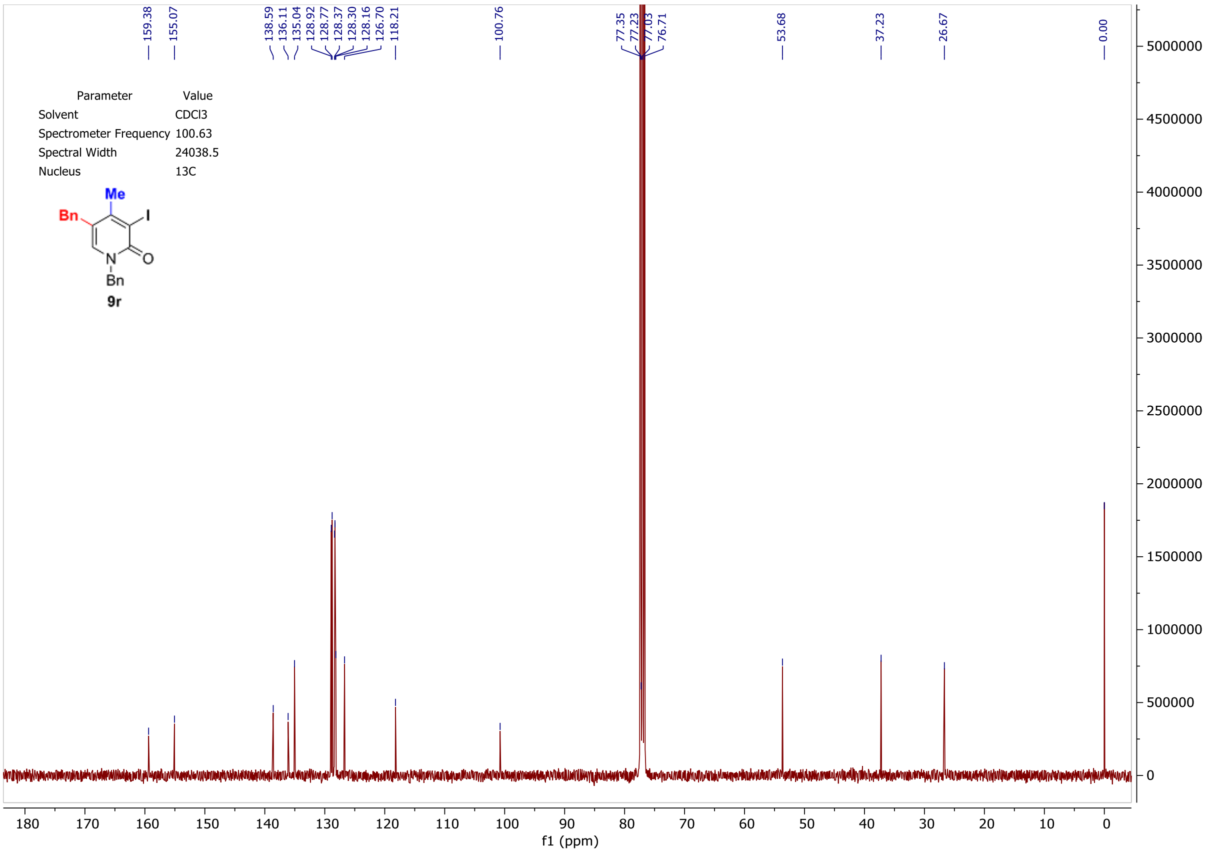
159.38
155.07
138.59
136.11
135.04
128.92
128.77
128.37
128.30
128.16
126.70
118.21
100.76
77.35
77.23
77.03
76.71
53.68
37.23
26.67
0.00
Parameter Value
Solvent CDCl3
Spectrometer Frequency 100.63
Spectral Width 24038.5
Nucleus 13C
Me
Bn
I
N
O
Bn
9r
f1 (ppm)

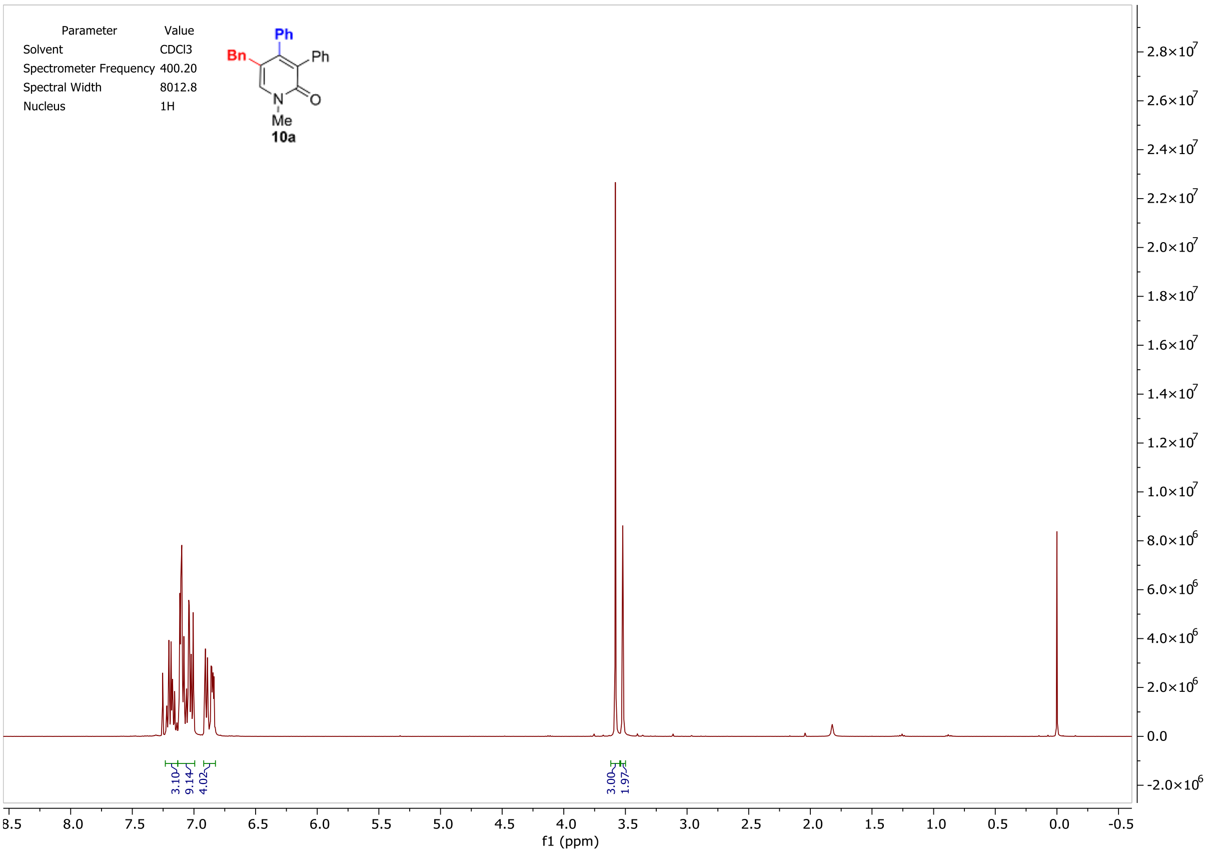

Parameter Value
Solvent CDCl3
Spectrometer Frequency 400.20
Spectral Width 8012.8
Nucleus 1H
Ph
Bn
Ph
N
O
Me
10a
3.10
9.14
4.02
3.00
1.97
f1 (ppm)

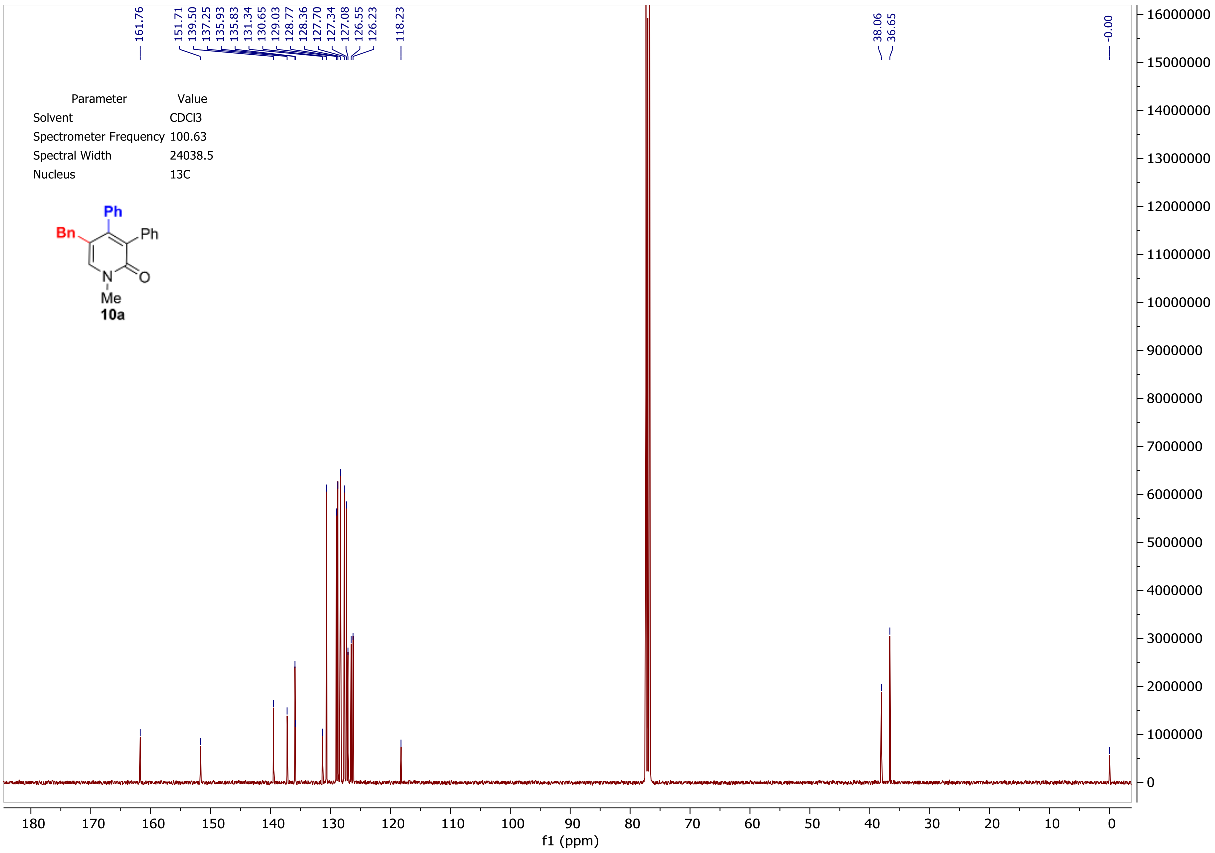

161.76
151.71
139.50
137.25
135.93
135.83
131.34
130.65
129.03
128.77
128.36
127.70
127.34
127.08
126.55
126.23
118.23
38.06
36.65
-0.00
Parameter Value
Solvent CDCl3
Spectrometer Frequency 100.63
Spectral Width 24038.5
Nucleus 13C
Ph
Bn
Ph
N
O
Me
10a
f1 (ppm)

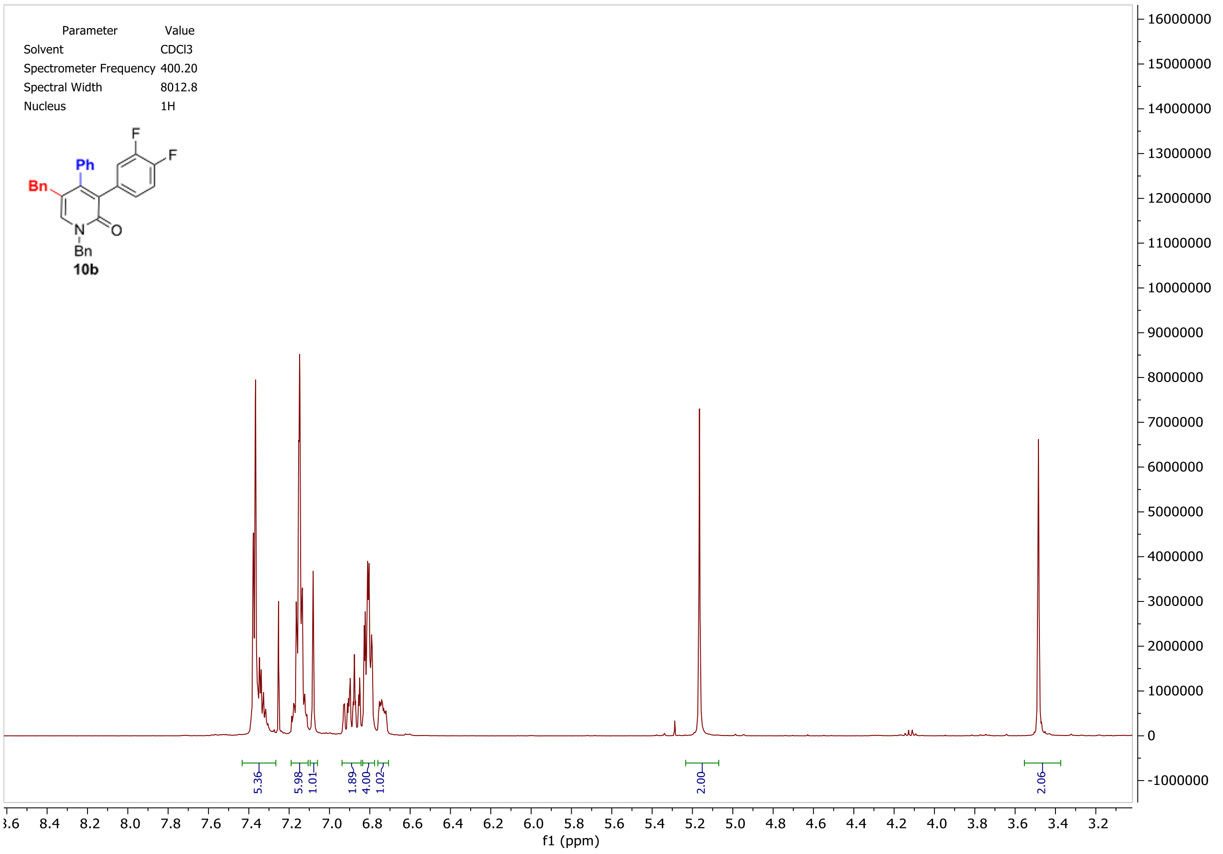
Parameter Value
Solvent CDCl3
Spectrometer Frequency 400.20
Spectral Width 8012.8
Nucleus 1H
Ph
Bn
F
F
N
O
Bn
10b
5.36
5.98
1.01
1.89
4.00
1.02
2.00
2.06
f1 (ppm)

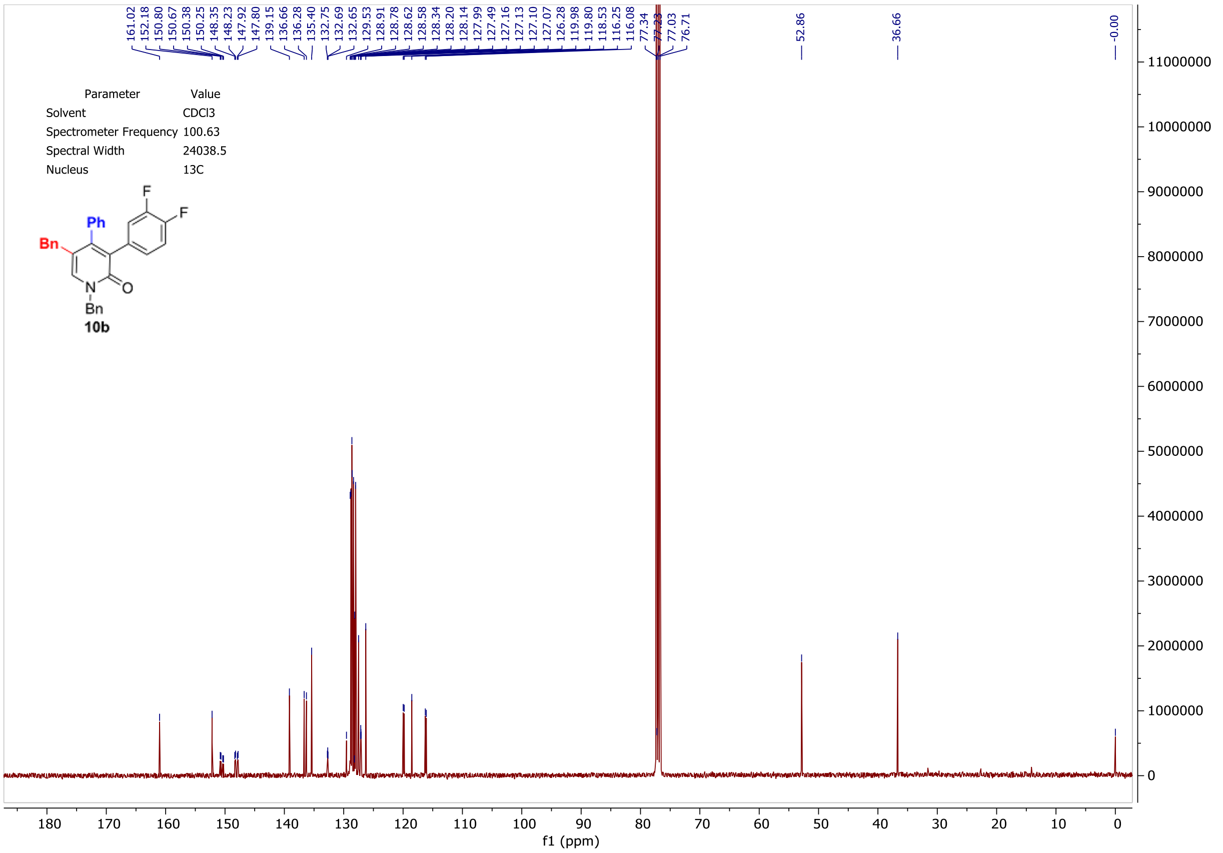
161.02
152.18
150.80
150.67
150.38
150.25
148.35
148.23
147.92
147.80
139.15
136.66
136.28
135.40
132.75
132.69
132.65
129.53
128.91
128.78
128.62
128.58
128.34
128.20
128.14
127.99
127.49
127.16
127.13
127.10
127.07
126.28
119.98
119.80
118.53
116.25
116.08
77.34
77.03
76.71
52.86
36.66
-0.00
Parameter Value
Solvent CDCl3
Spectrometer Frequency 100.63
Spectral Width 24038.5
Nucleus 13C
Ph
Bn
F
F
N
O
Bn
10b
f1 (ppm)

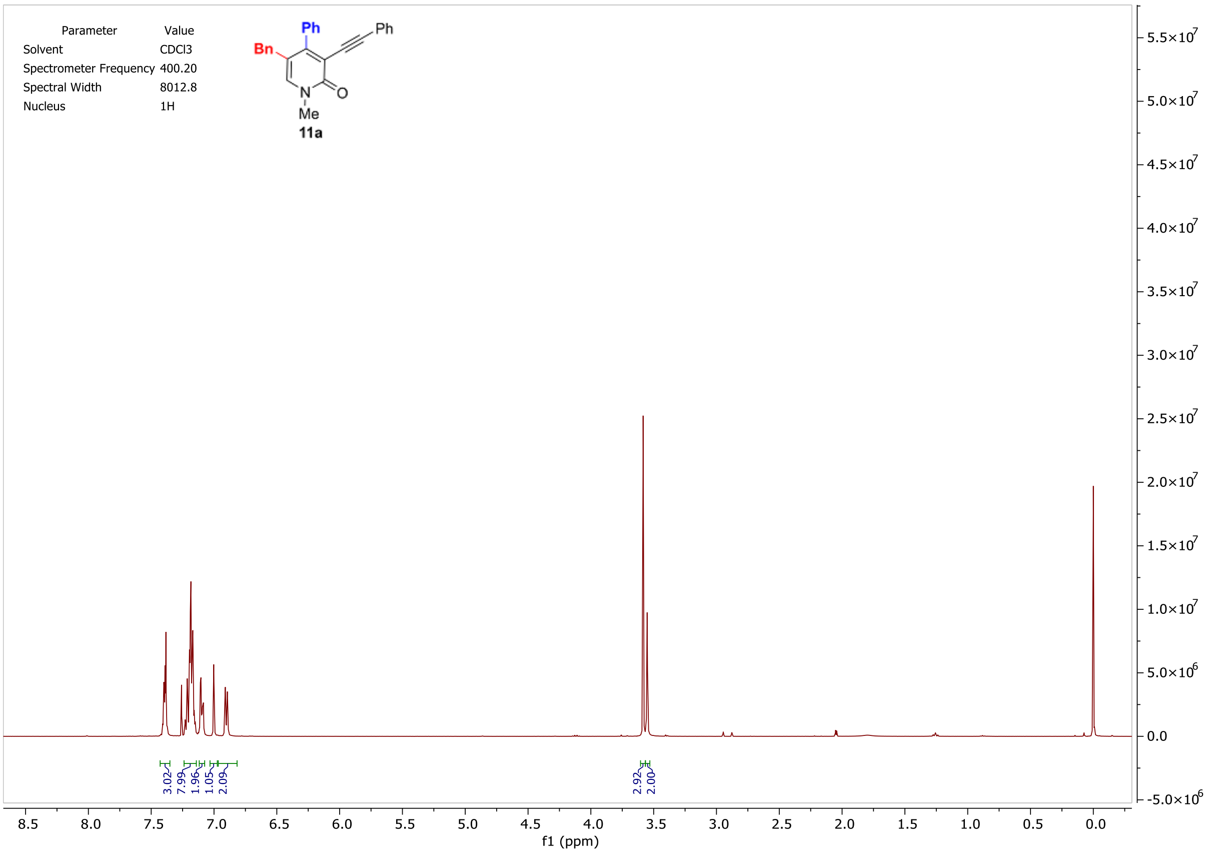

Parameter Value
Solvent CDCl3
Spectrometer Frequency 400.20
Spectral Width 8012.8
Nucleus 1H
Ph
Ph
Bn
N
O
Me
11a
3.02
7.99
1.96
1.05
2.09
2.92
2.00
f1 (ppm)

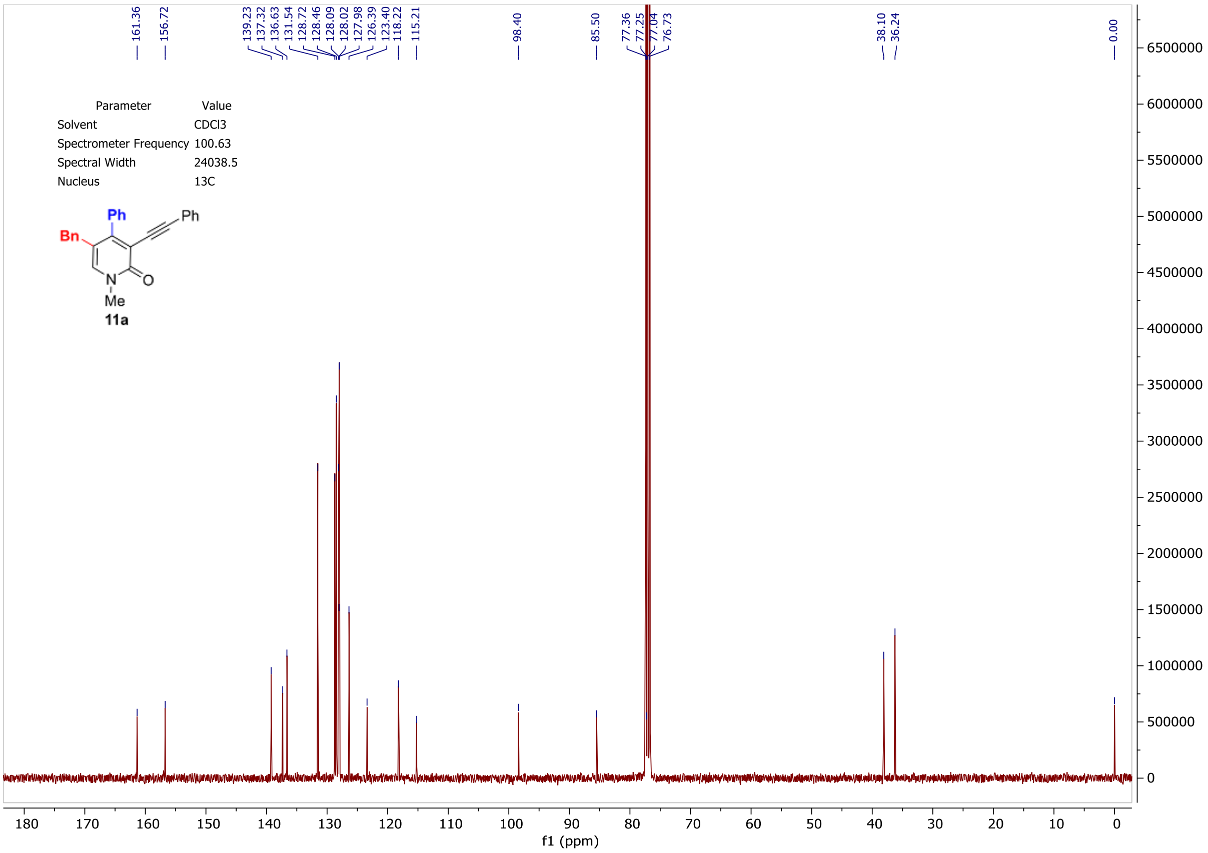

161.36
156.72
139.23
137.32
136.63
131.54
128.72
128.46
128.09
128.02
127.98
126.39
123.40
118.22
115.21
98.40
85.50
77.36
77.25
77.04
76.73
38.10
36.24
0.00
Parameter Value
Solvent CDCl3
Spectrometer Frequency 100.63
Spectral Width 24038.5
Nucleus 13C
Ph
Ph
Bn
N
O
Me
11a
f1 (ppm)

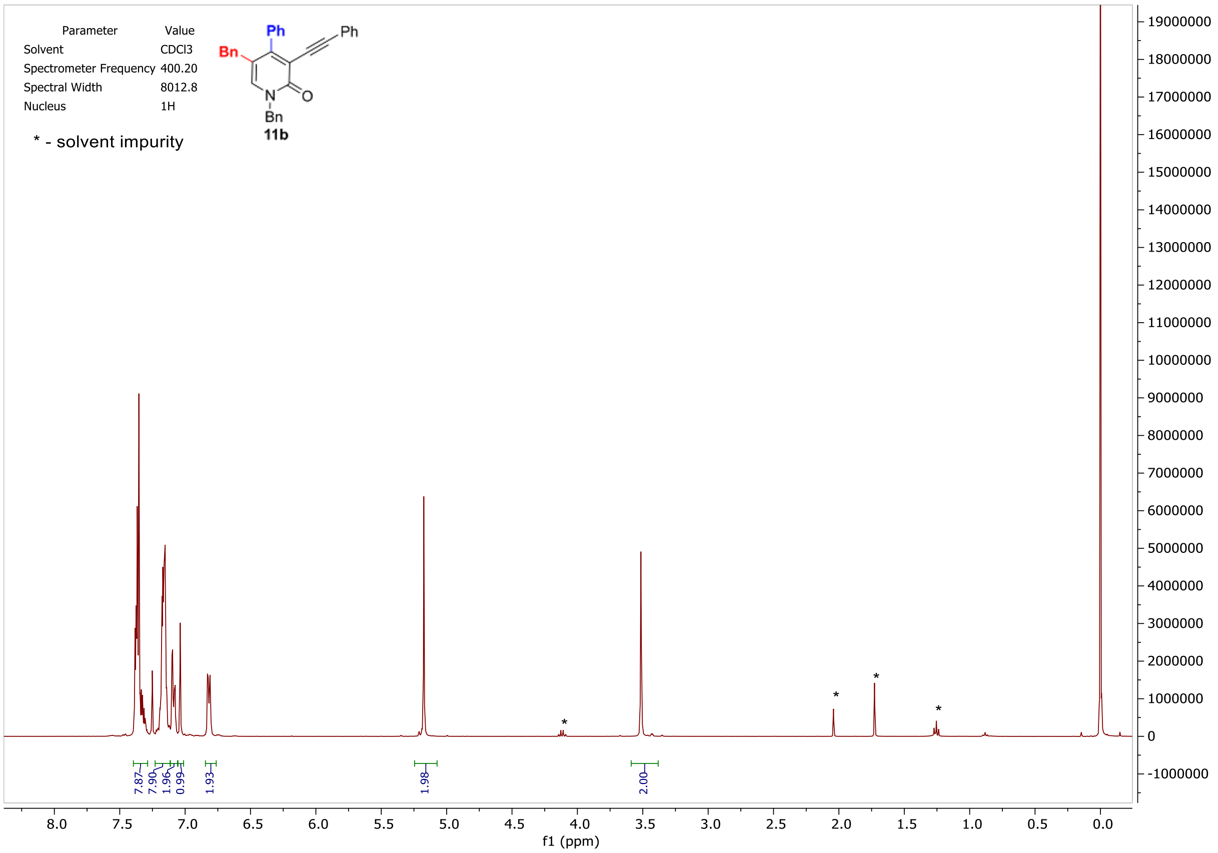
Parameter Value
Solvent CDCl3
Spectrometer Frequency 400.20
Spectral Width 8012.8
Nucleus 1H
* - solvent impurity
Ph
Bn
Ph
N
O
Bn
11b
7.87
7.90
1.96
0.99
1.93
1.98
2.00
f1 (ppm)

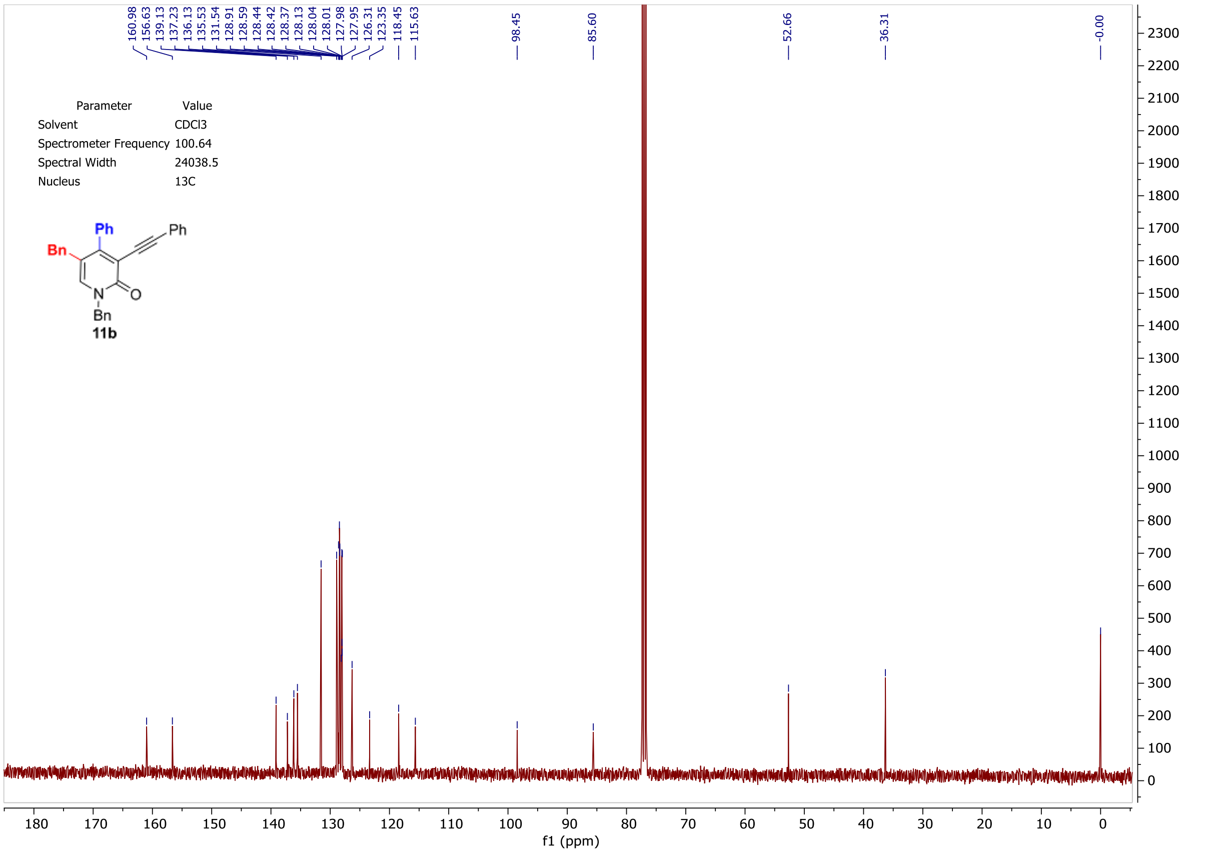
160.98
156.63
139.13
137.23
136.13
135.53
131.54
128.91
128.59
128.44
128.42
128.37
128.13
128.04
128.01
127.98
127.95
126.31
123.35
118.45
115.63
98.45
85.60
52.66
36.31
-0.00
Parameter Value
Solvent CDCl3
Spectrometer Frequency 100.64
Spectral Width 24038.5
Nucleus 13C
Ph
Bn
Ph
N
O
Bn
11b
f1 (ppm)

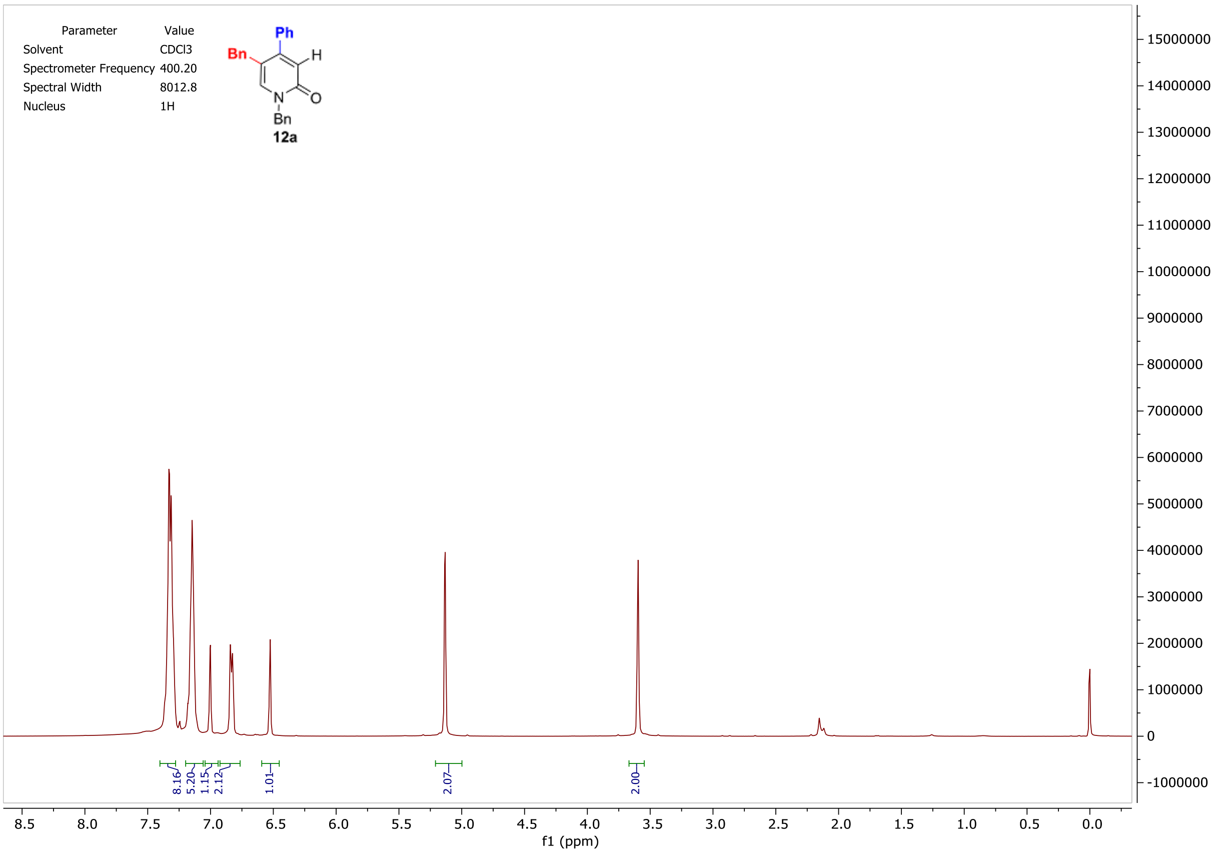
Parameter Value
Solvent CDCl3
Spectrometer Frequency 400.20
Spectral Width 8012.8
Nucleus 1H
Ph
Bn
H
N
O
Bn
12a
8.16
5.20
1.15
2.12
1.01
2.07
2.00
f1 (ppm)

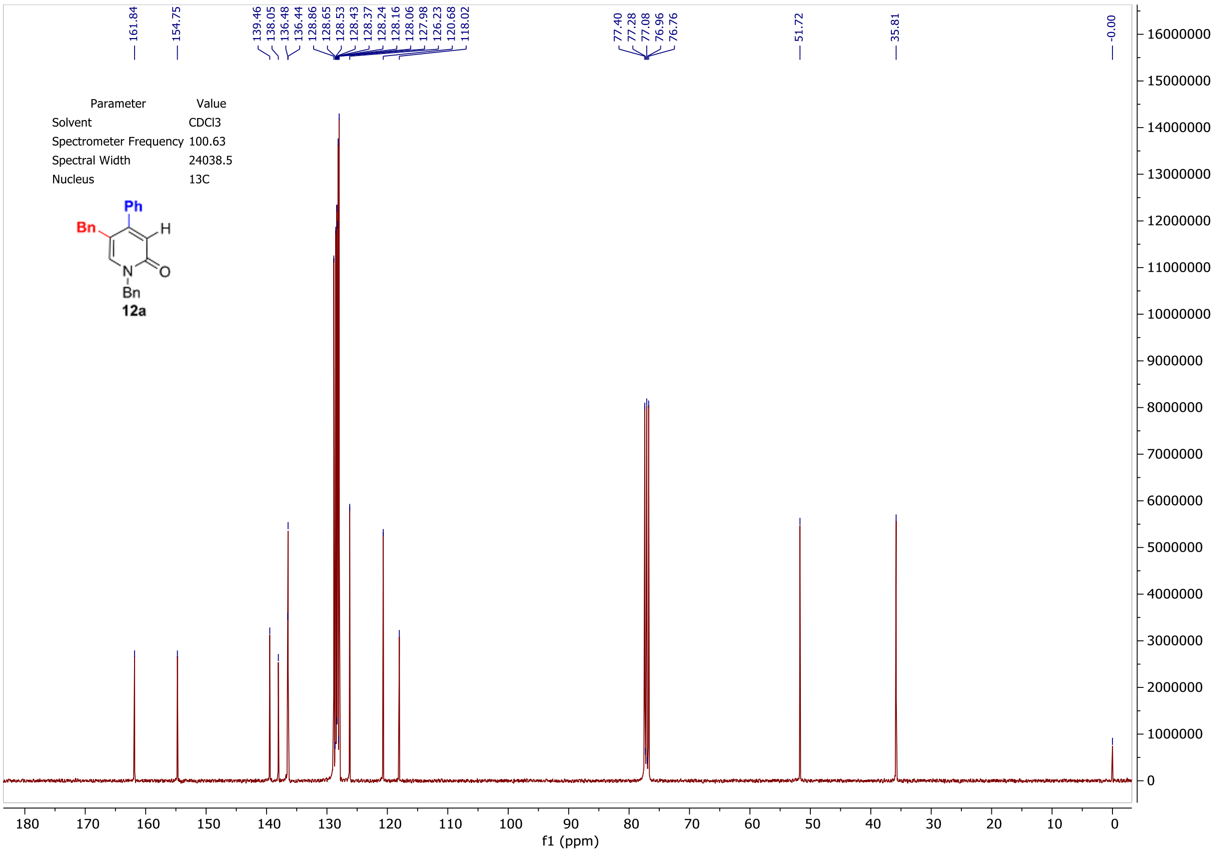
161.84
154.75
139.46
138.05
136.48
136.44
128.86
128.65
128.53
128.43
128.37
128.24
128.16
128.06
127.98
126.23
120.68
118.02
77.40
77.28
77.08
76.96
76.76
51.72
35.81
-0.00
Parameter Value
Solvent CDCl3
Spectrometer Frequency 100.63
Spectral Width 24038.5
Nucleus 13C
Ph
Bn
H
N
O
Bn
12a
f1 (ppm)

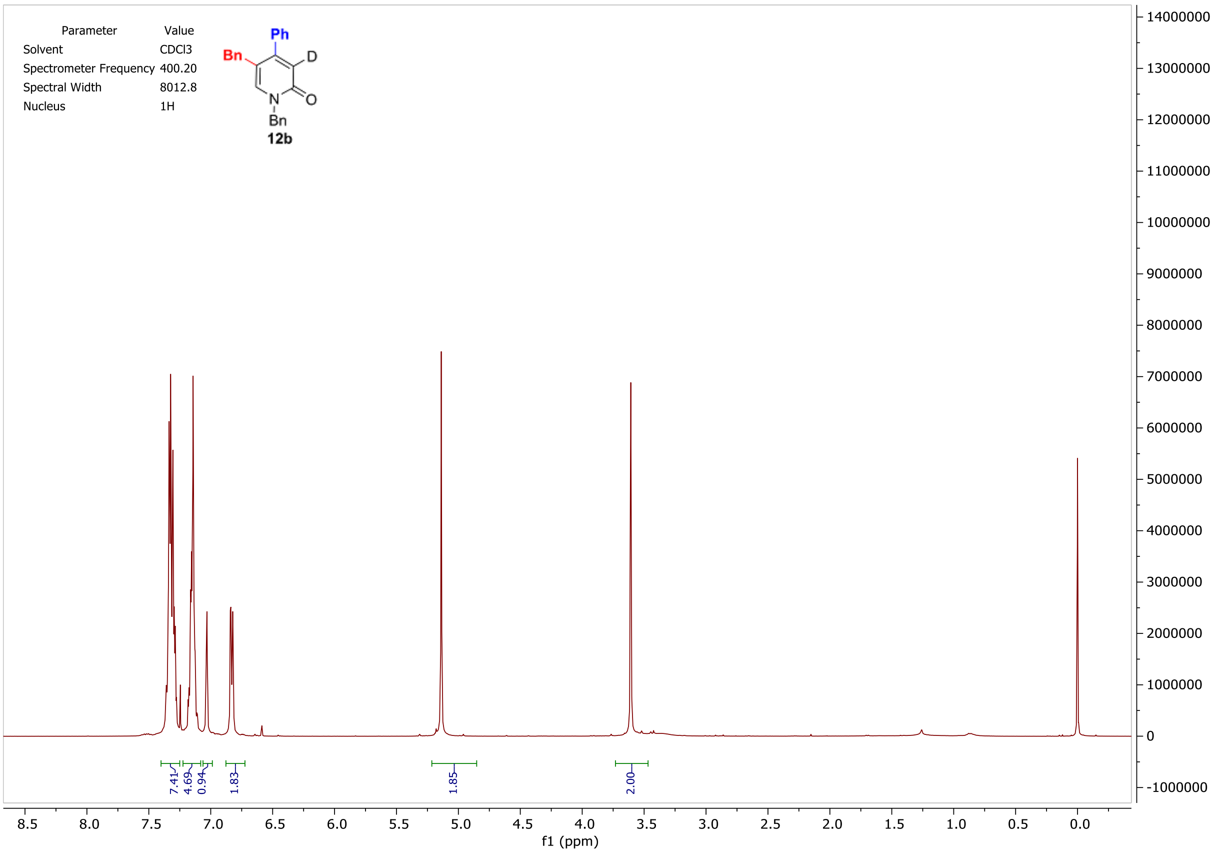
Parameter Value
Solvent CDCl3
Spectrometer Frequency 400.20
Spectral Width 8012.8
Nucleus 1H
12b
7.41
4.69
0.94
1.83
1.85
2.00
f1 (ppm)

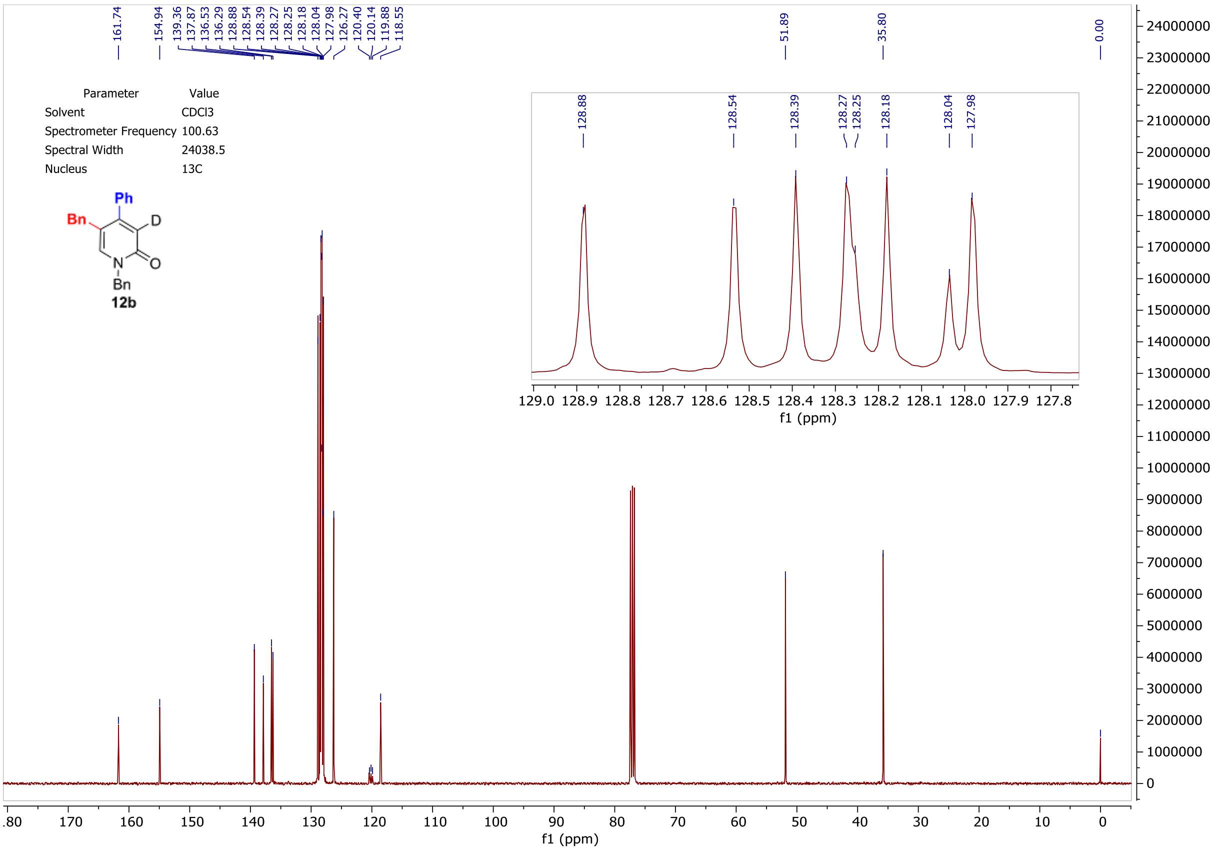
161.74
154.94
139.36
137.87
136.53
136.29
128.88
128.54
128.39
128.27
128.25
128.18
128.04
127.98
126.27
120.40
120.14
119.88
118.55
51.89
35.80
0.00
Parameter Value
Solvent CDCl3
Spectrometer Frequency 100.63
Spectral Width 24038.5
Nucleus 13C
12b
f1 (ppm)

14. Selected 2D NMR spectra of **5a**, **5b**, **6a**, **6c**, **6d**, **9o**, **9p**, **9q**, **9r** and **11b**

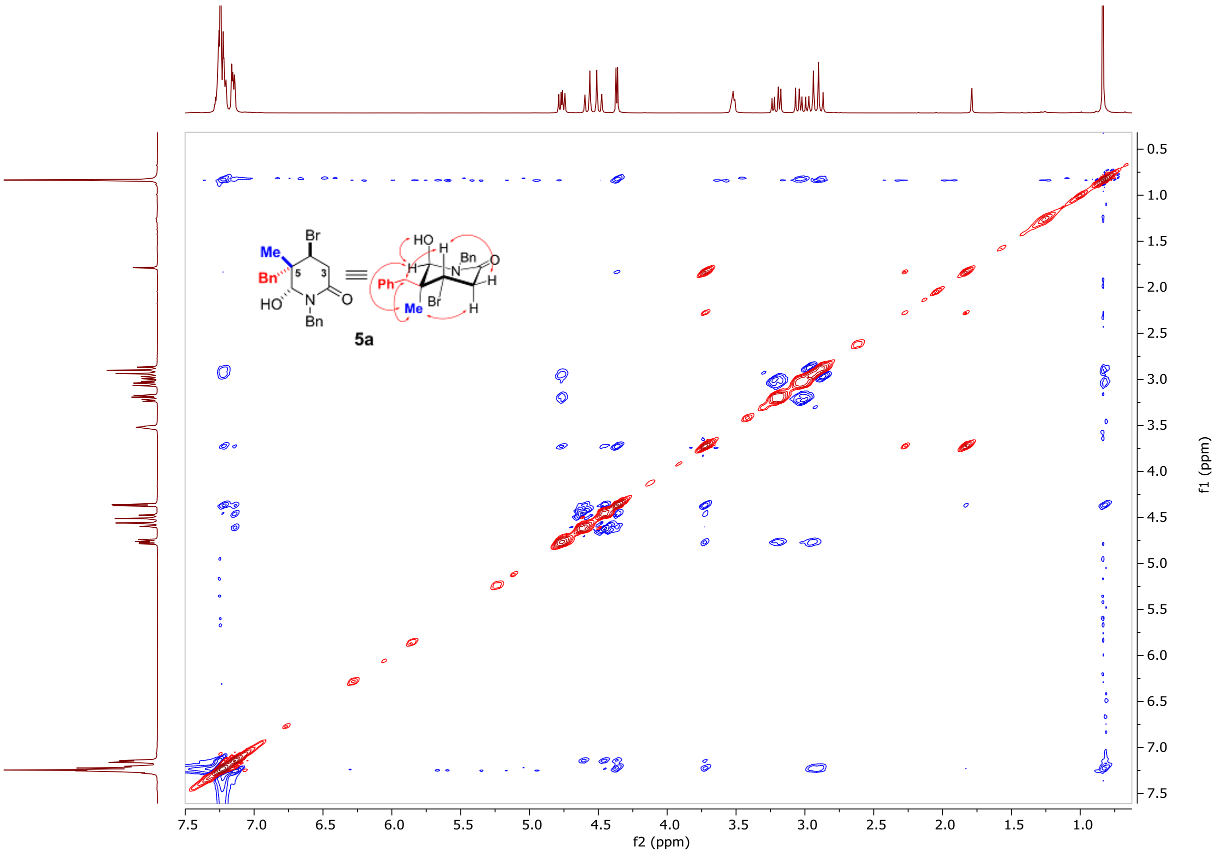

Figure S4. ^{1}H,^{1}H NOESY NMR spectrum of pure **5a** taken in $CDCl_3$

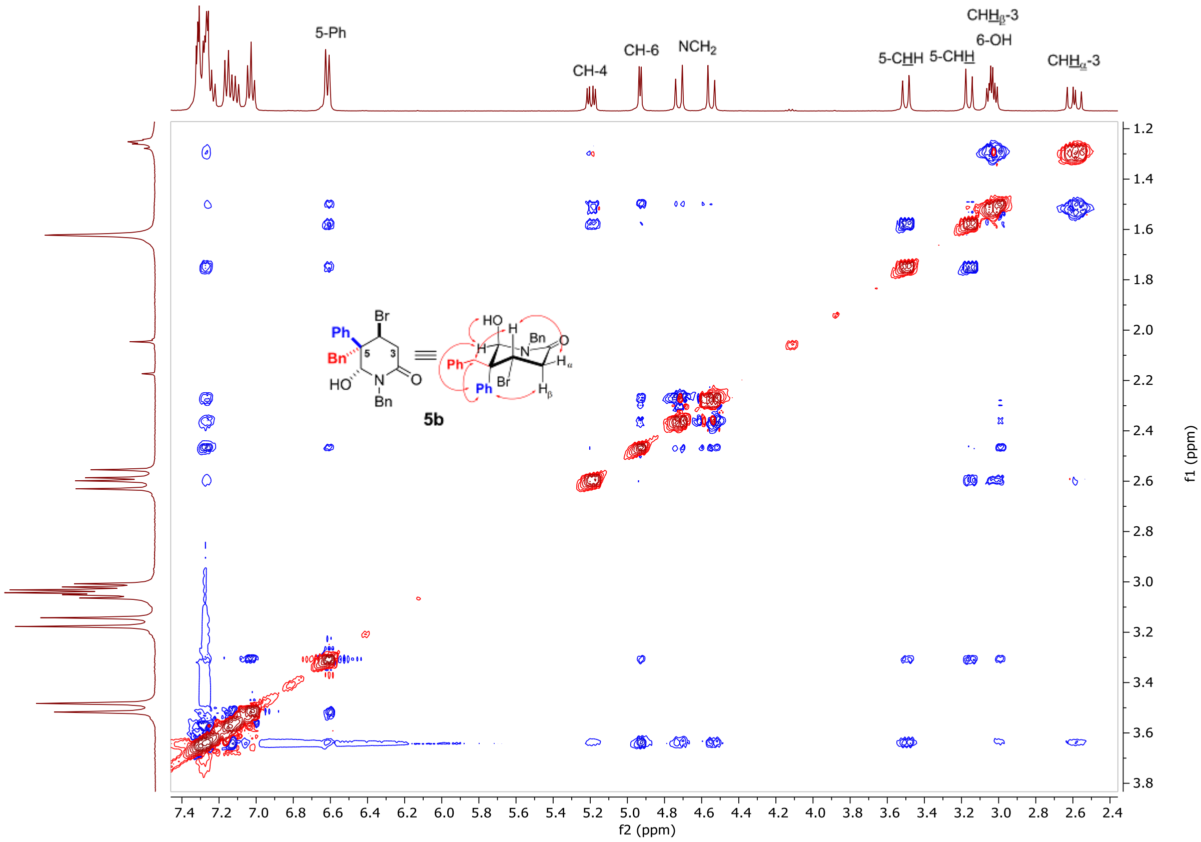

Figure S5. ^{1}H,^{1}H NOESY NMR spectrum of pure **5b** taken in $CDCl_3$

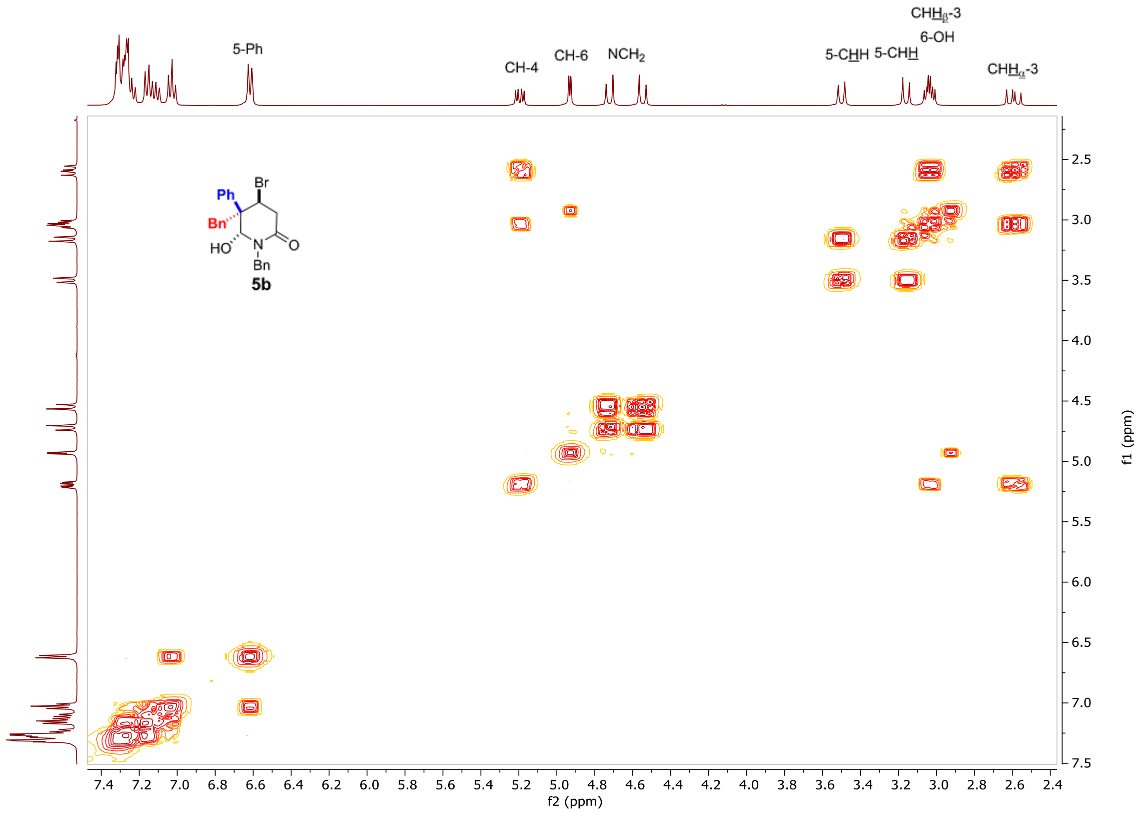

Figure S6. ^{1}H,^{1}H COSY NMR spectrum of pure **5b** taken in $CDCl_3$

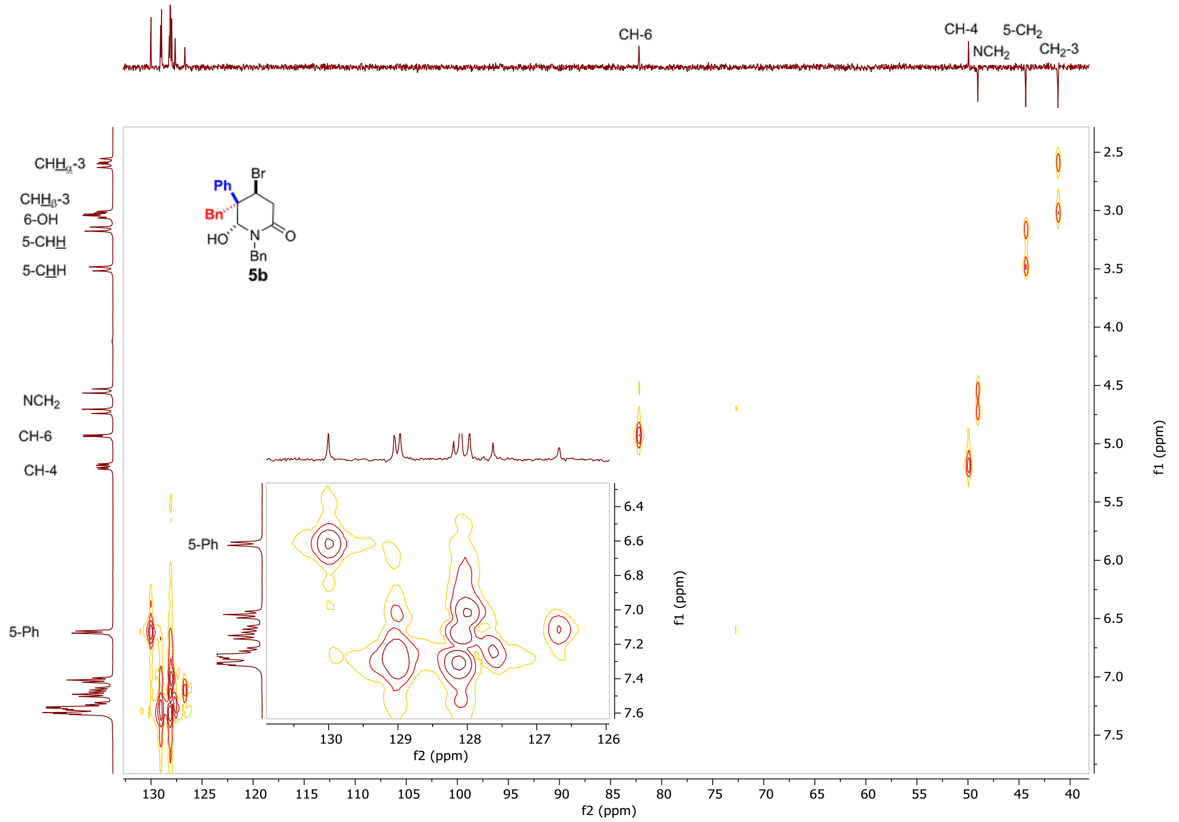

Figure S7. ^{13}C,^{1}H COSY NMR spectrum of pure **5b** taken in $CDCl_3$ with ^{13}C-DEPT-135 as horizontal trace

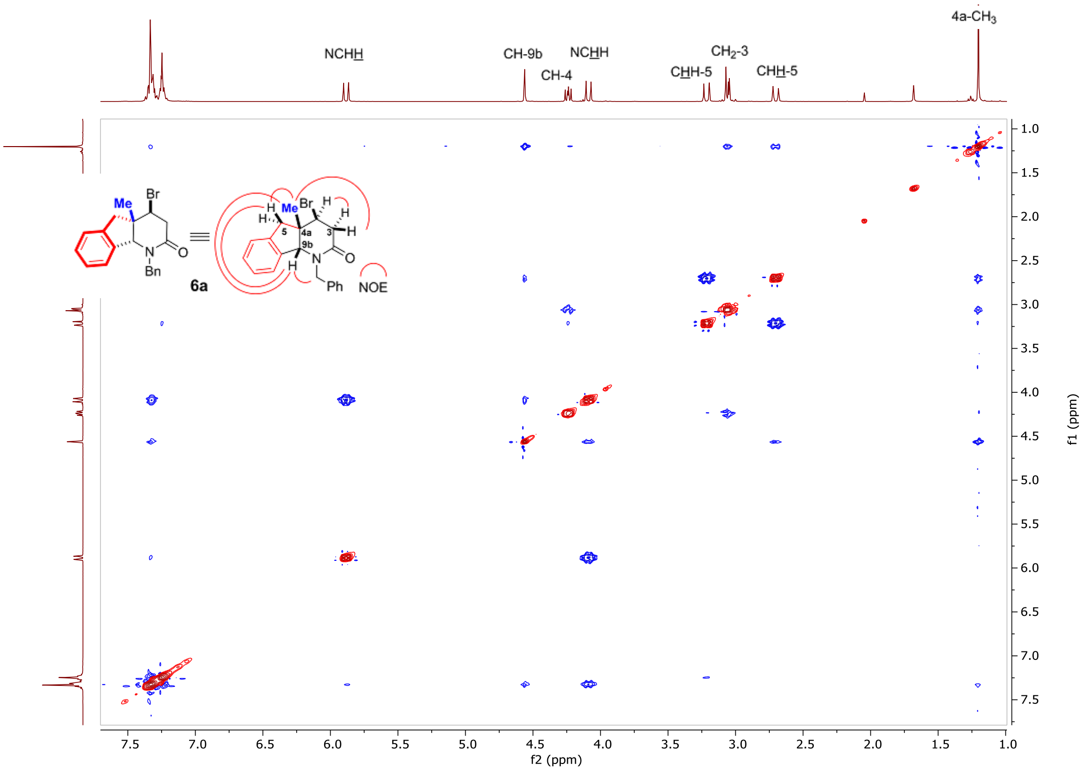

Figure S8. $^{1}H,^{1}H$ NOESY NMR spectrum of pure **6a** taken in $CDCl_3$

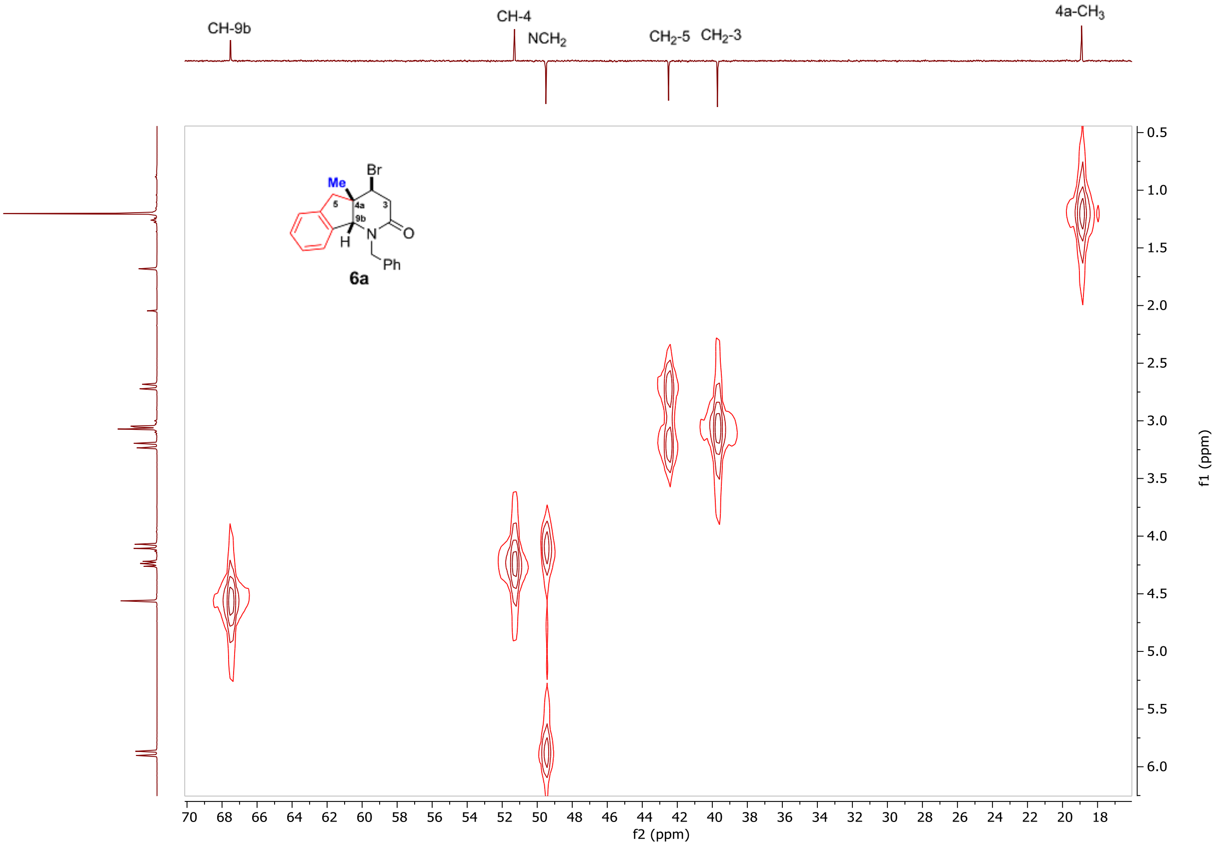

Figure S9. $^{13}C,^{1}H$ COSY NMR spectrum of pure **6a** taken in $CDCl_3$ with ^{13}C-DEPT-135 as horizontal trace

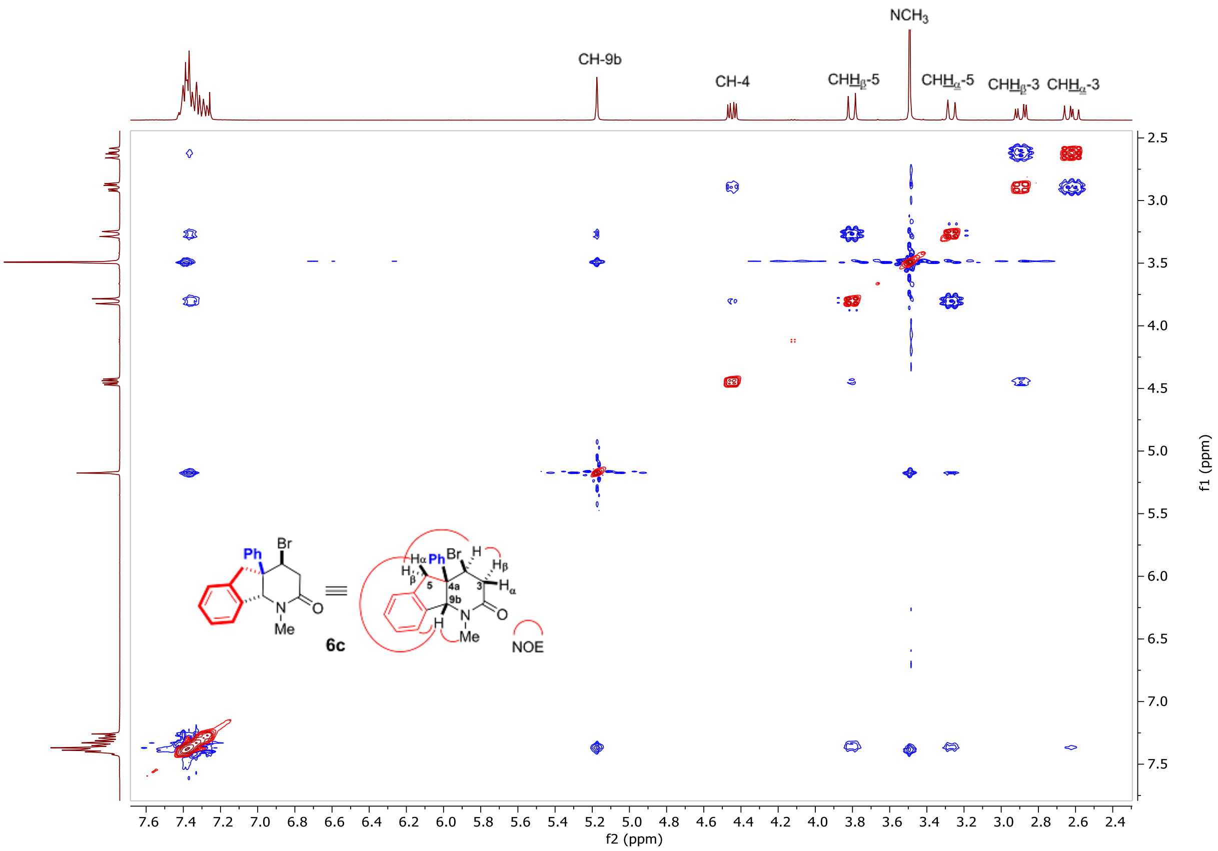

Figure S10. $^{1}H,^{1}H$ NOESY NMR spectrum of pure **6c** taken in $CDCl_3$

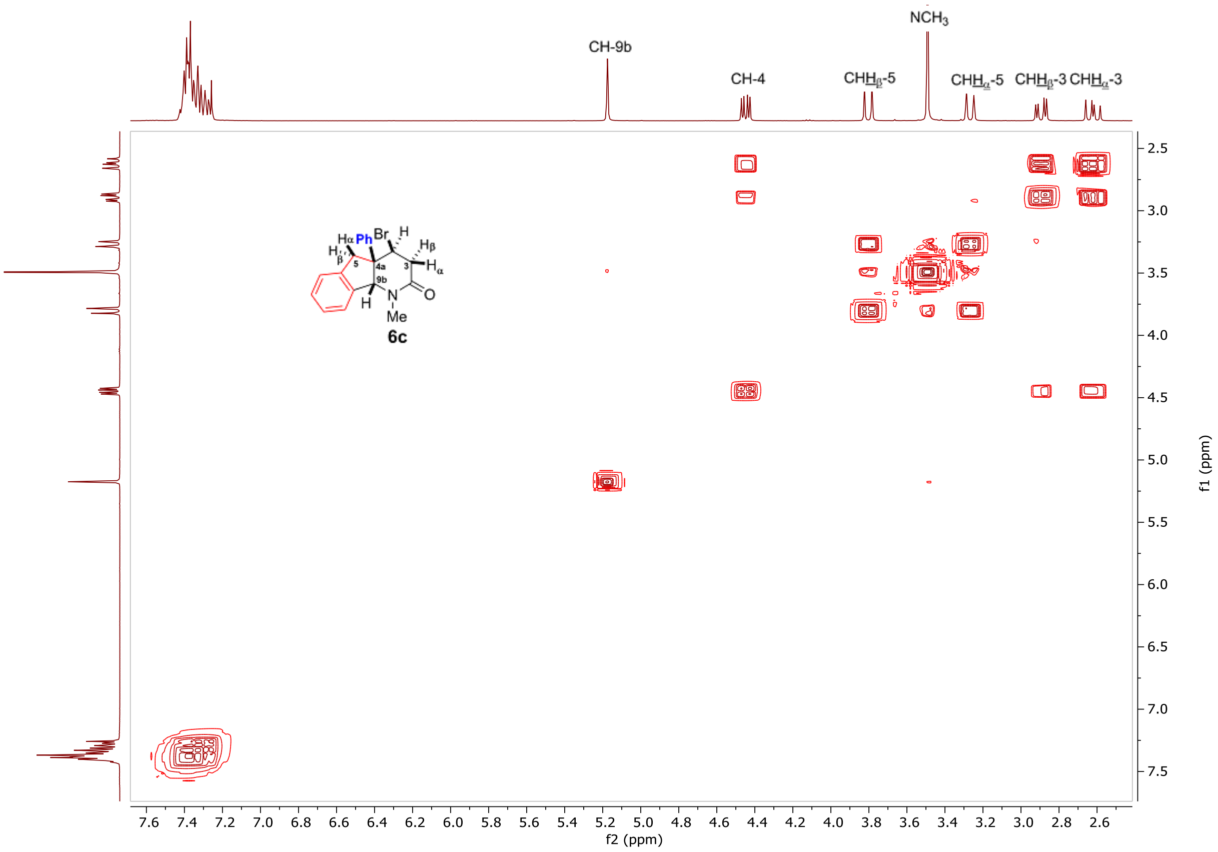

Figure S11. $^{1}H,^{1}H$ COSY NMR spectrum of pure **6c** taken in $CDCl_3$

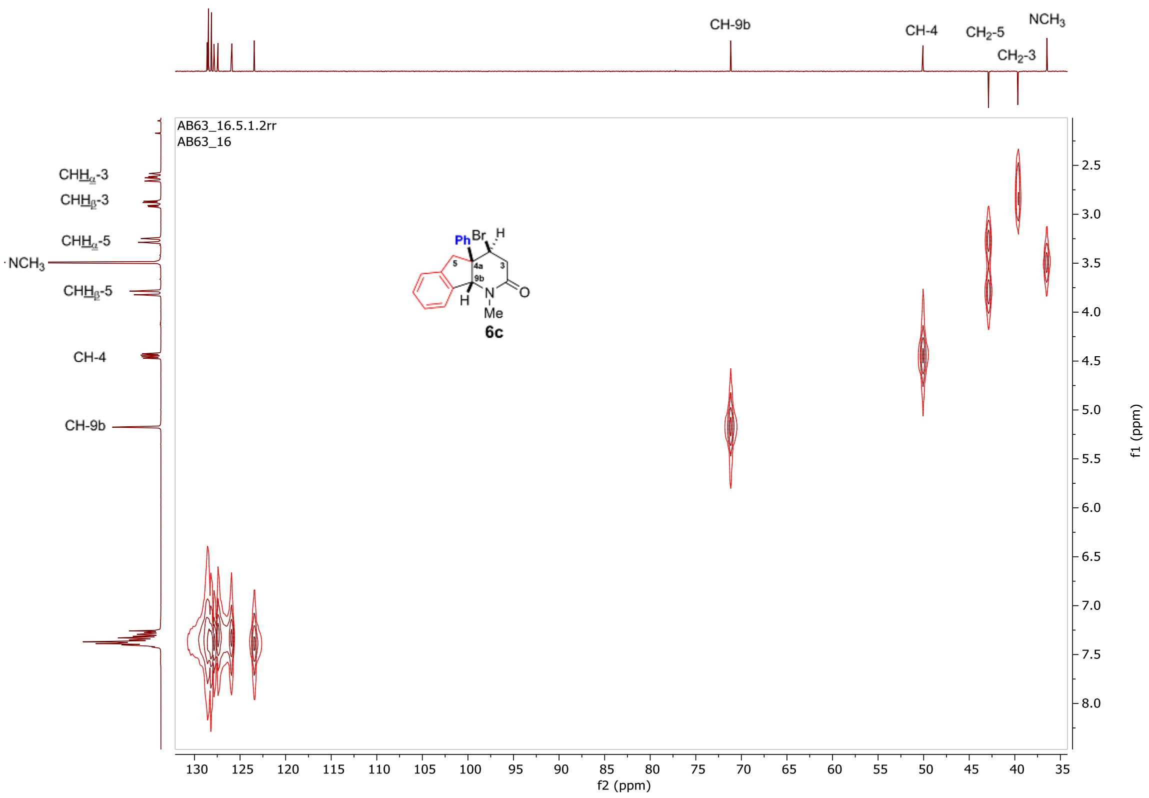

Figure S12. ^{13}C,^{1}H COSY NMR spectrum of pure **6c** taken in $CDCl_3$ with ^{13}C-DEPT-135 as horizontal trace

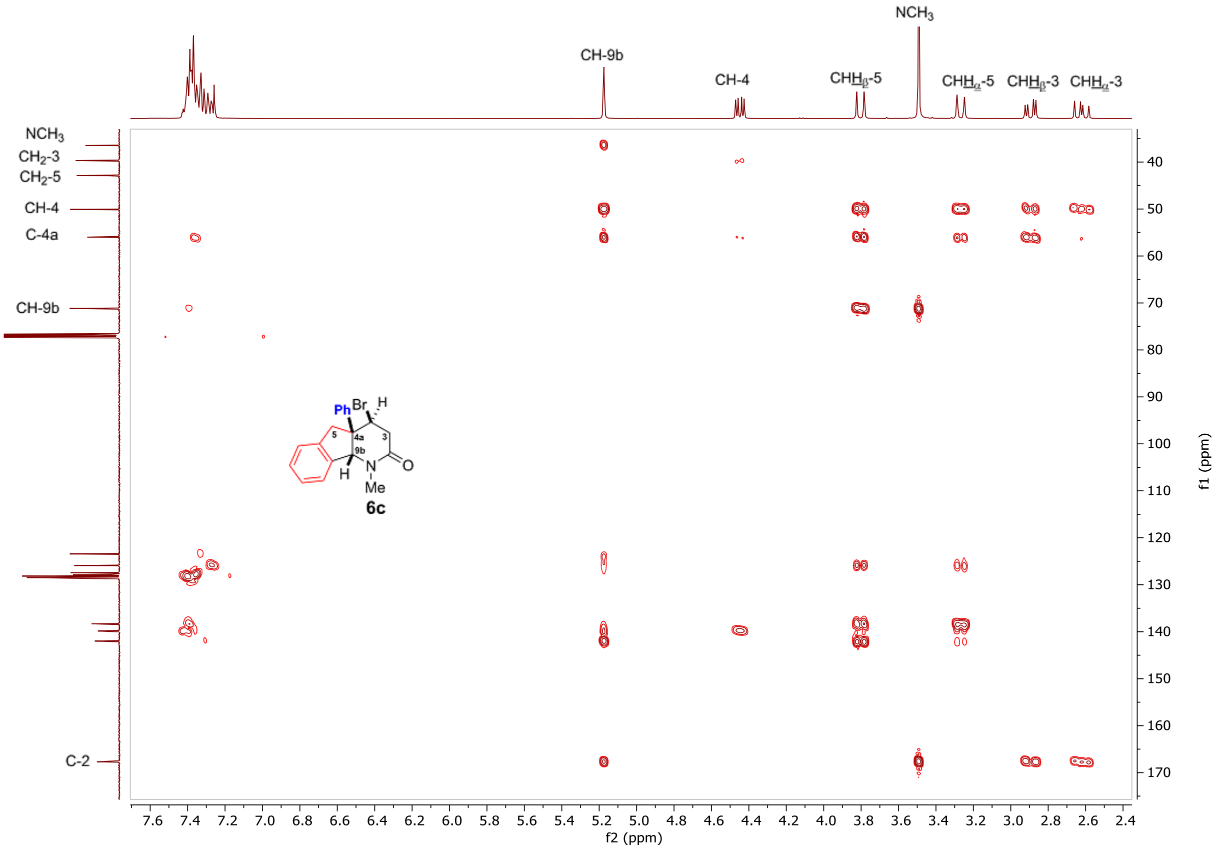

Figure S13. ^{1}H,^{13}C HMBC NMR spectrum of pure **6c** taken in $CDCl_3$

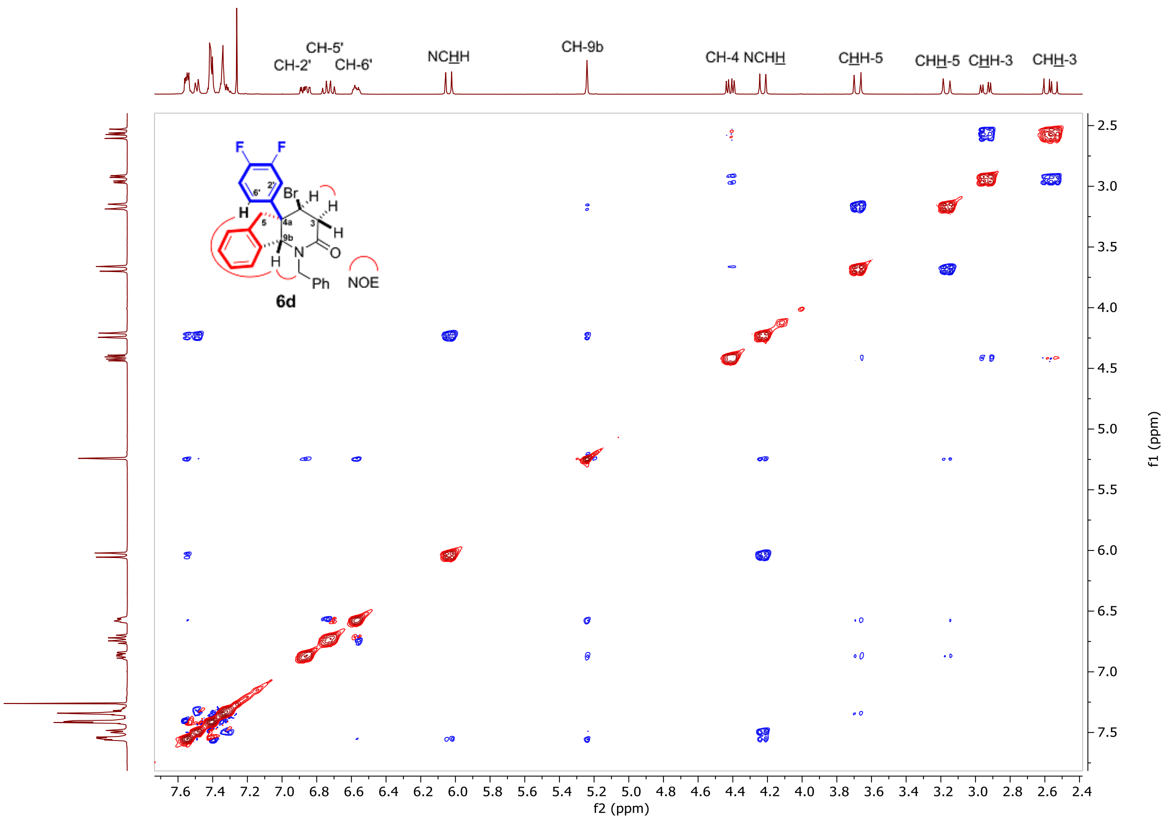

Figure S14. $^{1}H,^{1}H$ NOESY NMR spectrum of pure **6d** taken in $CDCl_3$

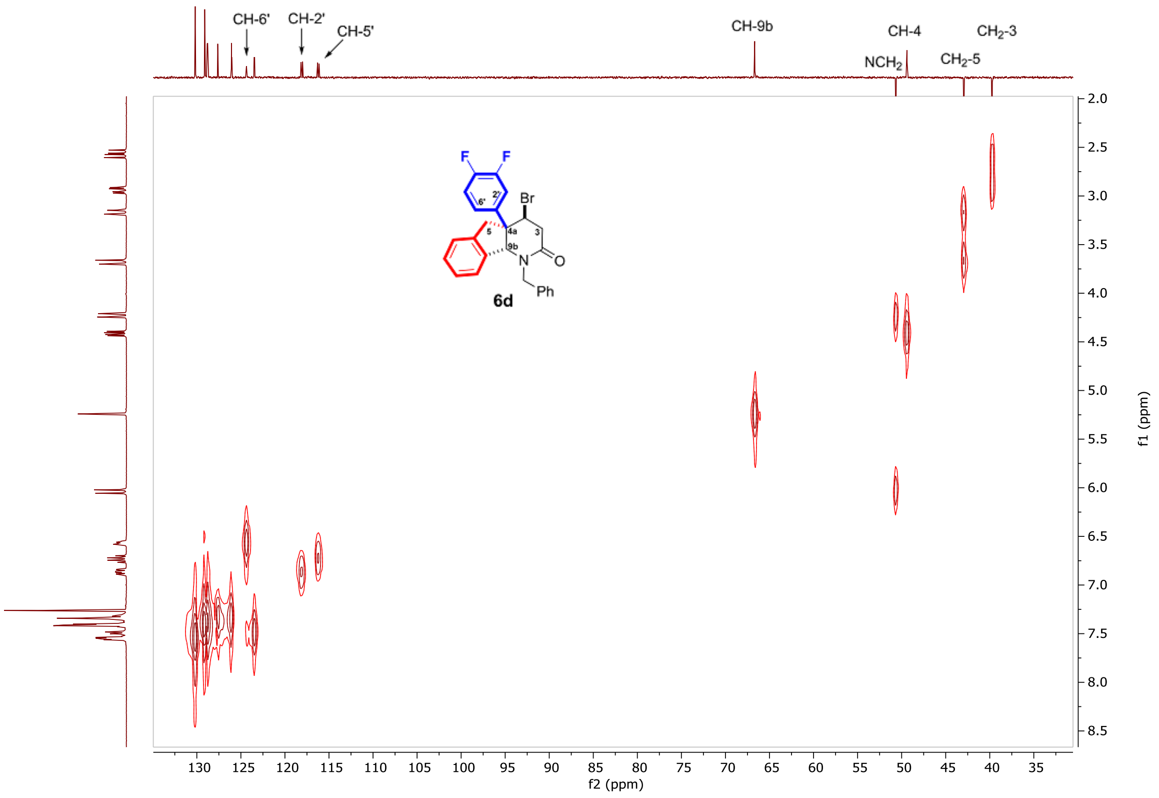

Figure S15. $^{13}C,^{1}H$ COSY NMR spectrum of pure **6d** taken in $CDCl_3$ with ^{13}C-DEPT-135 as horizontal trace

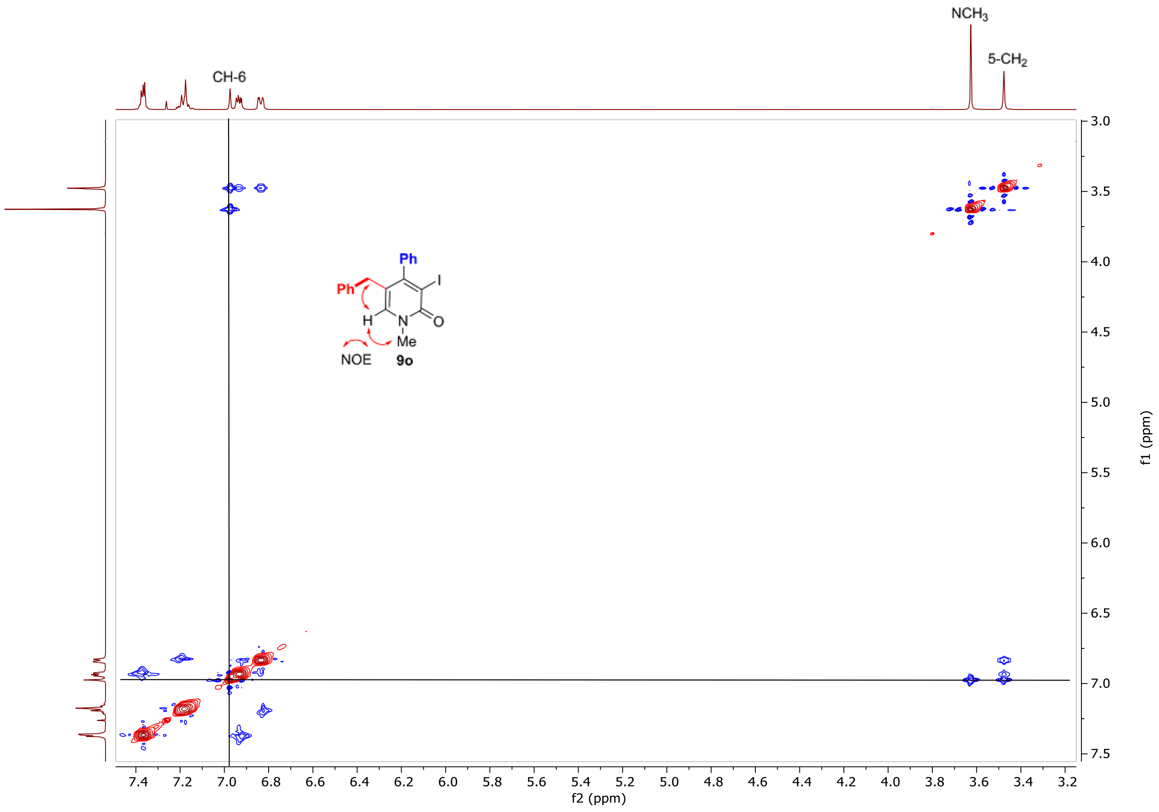

Figure S16. ^{1}H,^{1}H NOESY NMR spectrum of pure **9o** taken in $CDCl_3$

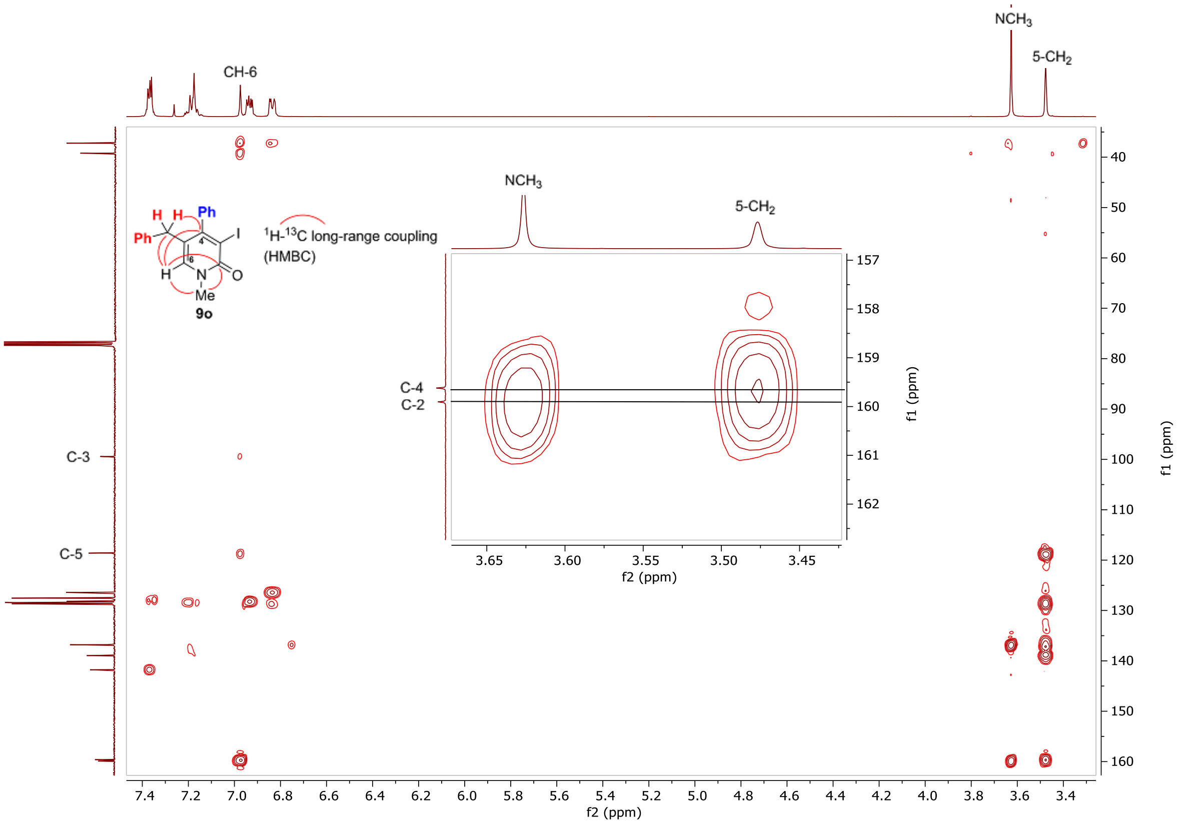

Figure S17. ^{1}H,^{13}C HMBC NMR spectrum of pure **9o** taken in $CDCl_3$

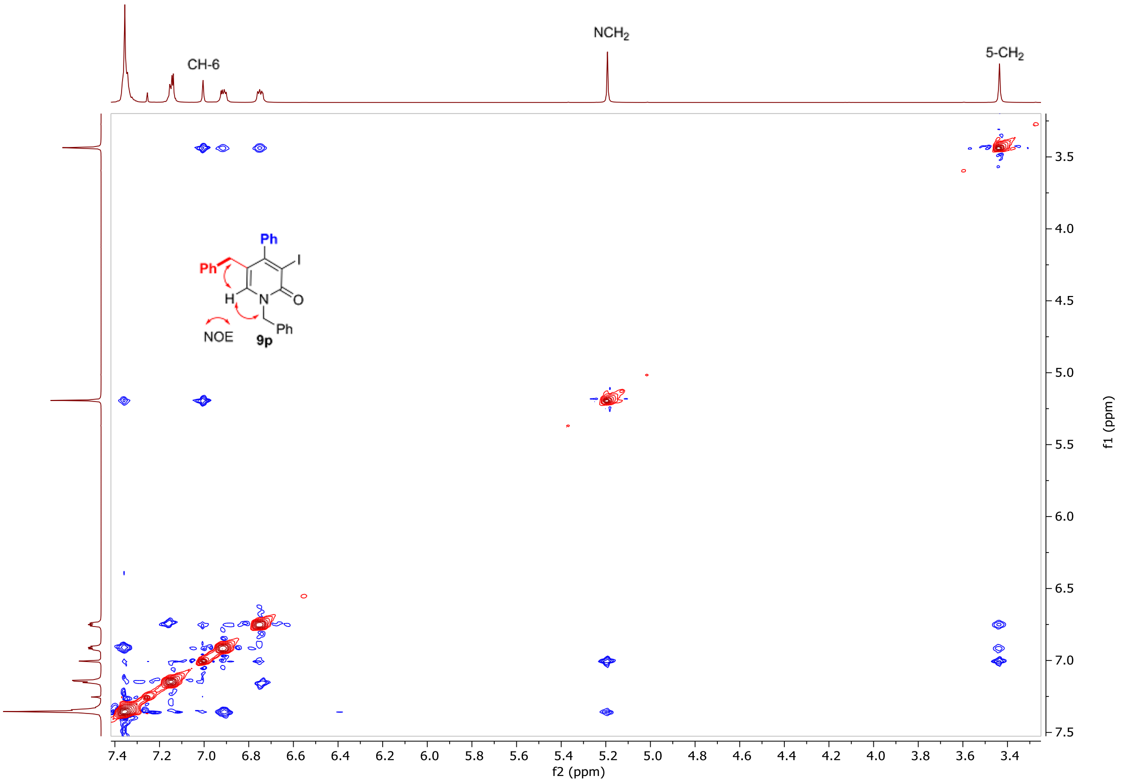

Figure S18. $^{1}H,^{1}H$ NOESY NMR spectrum of pure **9p** taken in $CDCl_3$

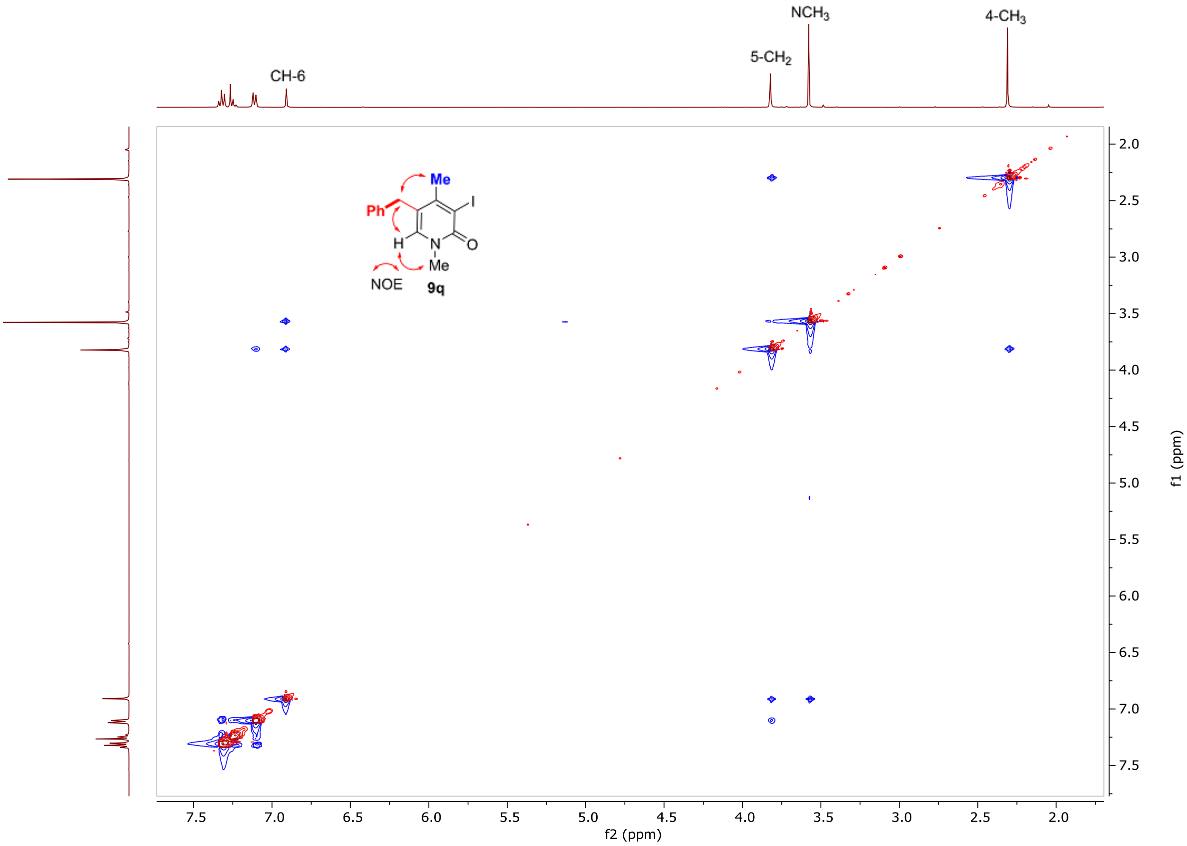

Figure S19. $^{1}H,^{1}H$ NOESY NMR spectrum of pure **9q** taken in $CDCl_3$

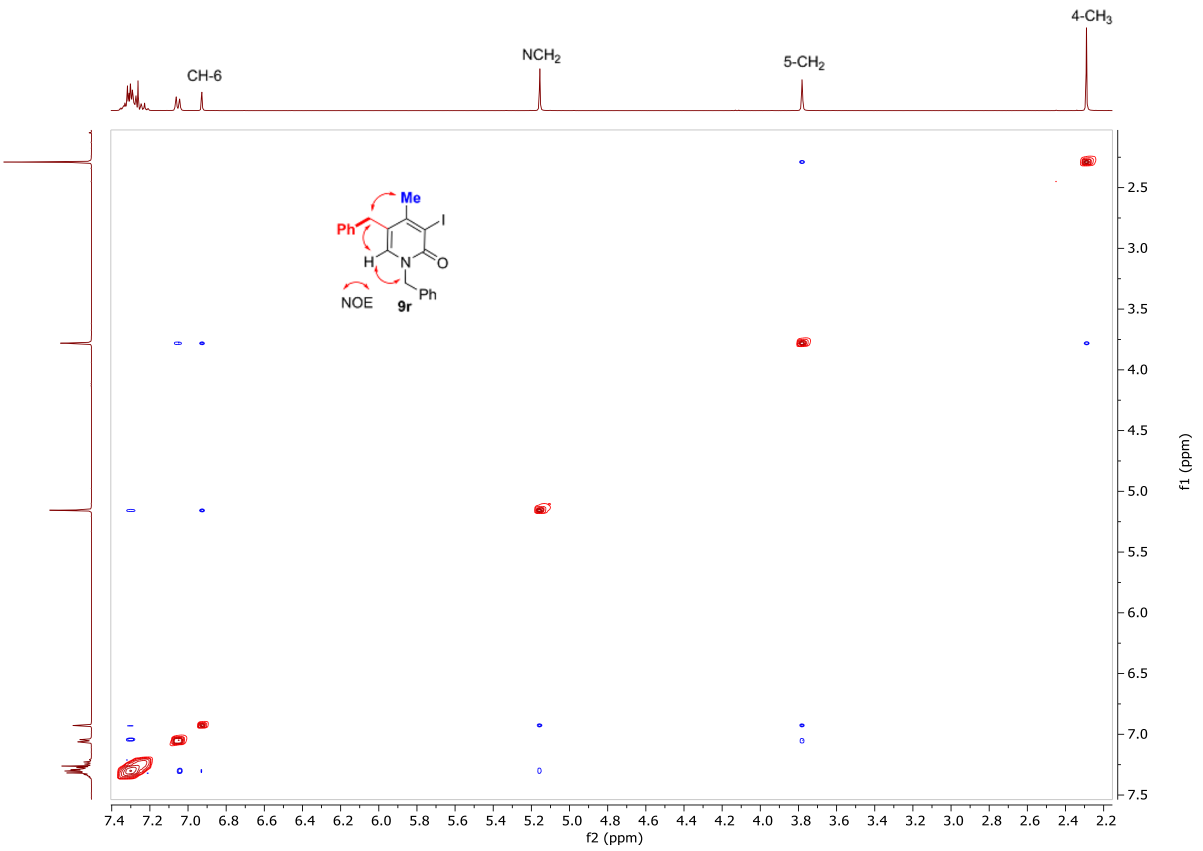

Figure S20. ^{1}H,^{1}H NOESY NMR spectrum of pure **9r** taken in $CDCl_3$

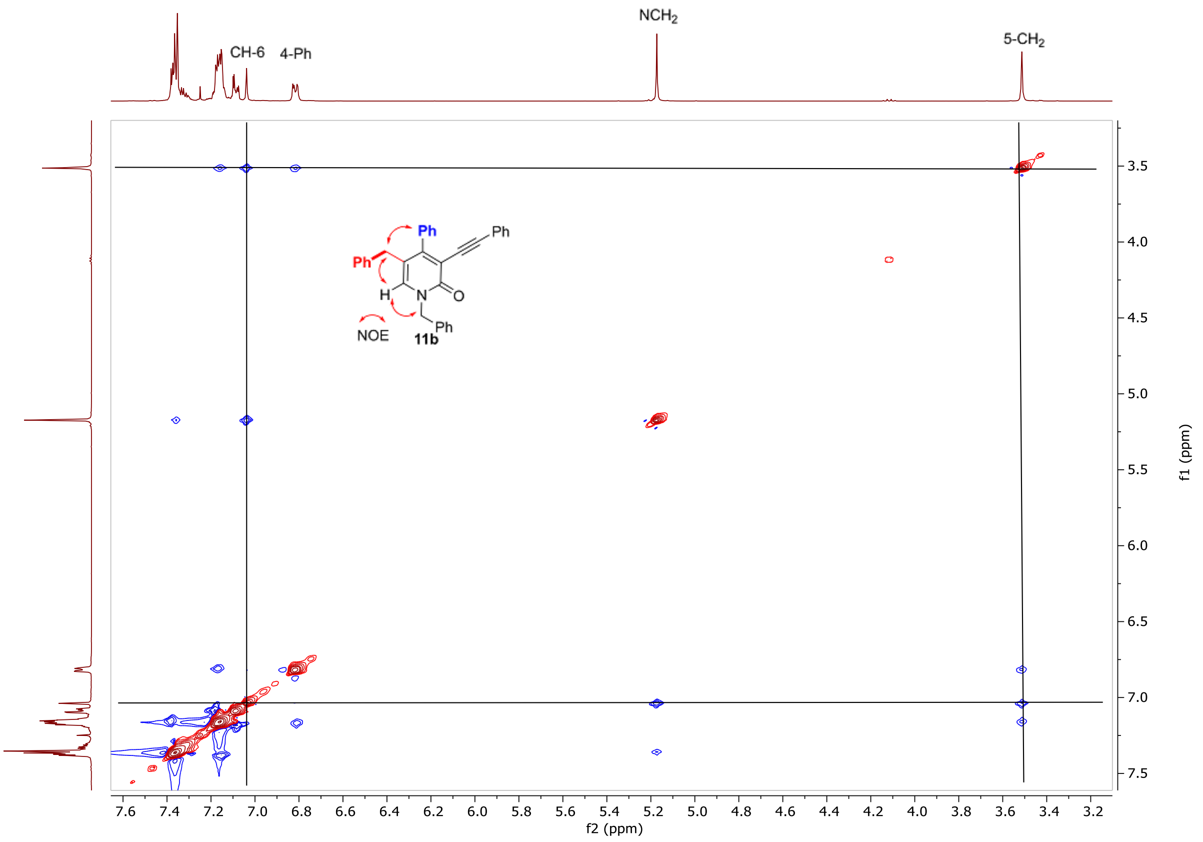

Figure S21. ^{1}H,^{1}H NOESY NMR spectrum of pure **11b** taken in $CDCl_3$

15. References

[1] Rosenau, C, P.; Jelier, B. J.; Gossert, A. D.; Togni, A. Exposing the Origins of Irreproducibility in Fluorine NMR Spectroscopy. *Angew. Chem. Int. Ed.* **2018**, *57*, 9528–9533.

[2] Sośnicki, J. G.; Idzik, T.; Borzyszkowska, A.; Wróblewski, E.; Maciejewska, G.; Struk, Ł. Addition of novel benzylmagnesium “ate” complexes of BnR_2MgLi type to 2-(thio)pyridones and related compounds. *Tetrahedron* **2017**, *73*, 481–493.

[3] Perużyńska, M.; Borzyszkowska-Ledwig, A.; Sośnicki, J. G.; Struk, Ł.; Idzik, T. J.; Maciejewska, G.; Skalski, Ł.; Piotrowska, K.; Łukasik, P.; Droździk, M.; Kurzawski, M. Synthesis and anticancer activity of mitotic-specific 3,4-dihydropyridine-2(1*H*)-thiones. *Int. J. Mol. Sci.* **2021**, *22*, 1–36, 2462.

[4] Sośnicki, J. G.; Idzik, T. J.; Borzyszkowska, A.; Maciejewska, G.; Struk, Ł. Synthesis of Polycyclic δ-Lactams with Bridged Benzomorphan Skeleton: Selectivity and Diversity Driven by Substituents, *J. Org. Chem.* **2018**, *83*, 1745−1760.

[5] Bowman, W.R.; Bridge, C.F. Regioselective Synthesis of *N*-Alkyl Pyridones *Synth. Commun.* **1999**, *29*, 4051–4060.

[6] Liu, C.; Ni, Q.; Bao, F.; Qiu, J. A simple and efficient protocol for a palladium-catalyzed ligand-free Suzuki reaction at room temperature in aqueous DMF. *Green Chem.* **2011**, 13, 1260–1266.

[7] Struk, Ł.; Sośnicki, J. G. Noncryogenic Synthesis of Functionalized 2-Methoxypyridines by Halogen-Magnesium Exchange Using Lithium Dibutyl(isopropyl)magnesate(1-) and Lithium Chloride. *Synthesis* **2012**, 44, 735–746.

[8] Zheng, B.; Yao, Y.; Liu, Z.; Deng, L.; Anglin, J. L.; Jiang, H.; Venkataram Prasad B. V.; Song, Y. Crystallographic Investigation and Selective Inhibition of Mutant Isocitrate Dehydrogenase. *ACS Med. Chem. Lett.* **2013**, 4, 6, 542–546.

[9] de la Hoz, A.; Prieto, M. P.; Rajzmann, M.; de Cózar, A.; Díaz-Ortiz, A.; Moreno, A.; Cossío, F. P. Selectivity under microwave irradiation. Benzylation of 2-pyridone: an experimental and theoretical study. *Tetrahedron* **2008**, 64, 8169–8176.

[10] Sośnicki, J. G.; Struk, Ł.; Idzik, T.; Maciejewska, G. Scope and limitations of the synthesis of functionalized quinolizidinones and related compounds by a simple precursor approach via addition of lithium allylmagnesates to 2-pyridones and RCM as key steps. *Tetrahedron* **2014**, *70*, 8624–8635.

[11] Hartmann, R. W.; Reichert, M. New Nonsteroidal Steroid 5a-Reductase Inhibitors. Syntheses and Structure-Activity Studies on Carboxamide Phenylalkyl-Substituted Pyridones and Piperidones. *Archiv der Pharmazie*, **2000**, 333, 145–153.

[12] Gupton, J. T.; Polaski, C. M. Reaction of [3-(Dimethylamino)-2-Phenyl-Prop-2-en-1-Ylidene]Dimethylammonium Perchlorate with Grignard and Lithium Reagents. *Synth. Commun*. **1981**, 11, 561–570.

[13] Jutz, C.; Kirchlechner, R.; Siedel, H. J. Über die umsetzung von phenalen mit vinylogen farmamidinium-salzen: Synthese substituierter pyrene und des azuleno[5,6,7-cd]phenalens. *Chem. Ber*. **1969**, 102, 2301–2318.

[14] Jones, B. T.; García-Cárceles, J.; Caiger, L.; Hazelden, I. R.; Lewis, R.J.; Langer, T.; Bower, J. F. Complex Polyheterocycles and the Stereochemical Reassignment of Pileamartine A via Aza-Heck Triggered Aryl C−H Functionalization Cascades. *J. Am. Chem. Soc.* **2021**, 143, 15593−15598.

[15] Thesing, J.; Müller, A. Über Eine Neue Methode zur Darstellung von α-Pyridonen und Die Synthese des Nicotellins. *Chem. Ber*. **1957**, 90, 711–720.

[16] Yamamoto, K.; Yamazaki, S.; Murata, I. Ring Contraction Reactions of Dihydro- and Tetrahydrothiazepines to Isothiazolone Derivatives under Pummerer Conditions. *J. Org. Chem*.**1987**, 52, 5239–5243.

[17] Krasovskiy, A.; Knochel, P.; Convenient Titration Method for Organometallic Zinc, Magnesium, and Lanthanide Reagents. *Synthesis* **2006**, *5*, 0890–0891.
